# The Impact of Dysphagia in Myositis: A Systematic Review and Meta-Analysis

**DOI:** 10.3390/jcm9072150

**Published:** 2020-07-08

**Authors:** Bendix Labeit, Marc Pawlitzki, Tobias Ruck, Paul Muhle, Inga Claus, Sonja Suntrup-Krueger, Tobias Warnecke, Sven G. Meuth, Heinz Wiendl, Rainer Dziewas

**Affiliations:** 1 Department of Neurology with Institute of Translational Neurology, University Hospital Muenster, 48149 Muenster, Germany; Marc.Pawlitzki@ukmuenster.de (M.P.); Tobias.Ruck@ukmuenster.de (T.R.); Paul.Muhle@ukmuenster.de (P.M.); Inga.Claus@ukmuenster.de (I.C.); Sonja.Suntrup-Krueger@ukmuenster.de (S.S.-K.); Tobias.Warnecke@ukmuenster.de (T.W.); sven.meuth@ukmuenster.de (S.G.M.); heinz.wiendl@ukmuenster.de (H.W.); Rainer.Dziewas@ukmuenster.de (R.D.); 2 Institute for Biomagnetism and Biosignalanalysis, University of Muenster, 48149 Muenster, Germany

**Keywords:** myositis, inflammatory idiopathic myopathy, dysphagia, aspiration, pneumonia

## Abstract

(1) Background: Dysphagia is a clinical hallmark and part of the current American College of Rheumatology/European League Against Rheumatism (ACR/EULAR) diagnostic criteria for idiopathic inflammatory myopathy (IIM). However, the data on dysphagia in IIM are heterogenous and partly conflicting. The aim of this study was to conduct a systematic review on epidemiology, pathophysiology, outcome and therapy and a meta-analysis on the prevalence of dysphagia in IIM. (2) Methods: Medline was systematically searched for all relevant articles. A random effect model was chosen to estimate the pooled prevalence of dysphagia in the overall cohort of patients with IIM and in different subgroups. (3) Results: 234 studies were included in the review and 116 (10,382 subjects) in the meta-analysis. Dysphagia can occur as initial or sole symptom. The overall pooled prevalence estimate in IIM was 36% and with 56% particularly high in inclusion body myositis. The prevalence estimate was significantly higher in patients with cancer-associated myositis and with NXP2 autoantibodies. Dysphagia is caused by inflammatory involvement of the swallowing muscles, which can lead to reduced pharyngeal contractility, cricopharyngeal dysfunction, reduced laryngeal elevation and hypomotility of the esophagus. Swallowing disorders not only impair the quality of life but can lead to serious complications such as aspiration pneumonia, thus increasing mortality. Beneficial treatment approaches reported include immunomodulatory therapy, the treatment of associated malignant diseases or interventional procedures targeting the cricopharyngeal muscle such as myotomy, dilatation or botulinum toxin injections. (4) Conclusion: Dysphagia should be included as a therapeutic target, especially in the outlined high-risk groups.

## 1. Introduction

Idiopathic inflammatory myopathies (IIM) are a heterogeneous group of autoimmune diseases in which inflammation of the striated skeletal muscles leads to myalgia and weakness. As distinct subgroups, they include dermatomyositis (DM), inclusion body myositis (IBM) and polymyositis (PM), defined by clinical, serological and histological criteria. In DM, both muscles and skin tissues are affected. IBM owes its name to the histological findings of protein aggregates in muscle cells. In PM there is no skin involvement and an inflammation of the muscle tissue occurs without evidence of inclusion bodies in muscle biopsy. Besides these major groups, there are also overlap syndromes in which symptoms of other rheumatological diseases occur in combination with muscle impairment. In recent years, the role of autoantibodies has been increasingly recognized in both research and diagnostics. Specific autoantibodies are hypothesized to be involved in the pathophysiology of inflammation and thus are associated with distinct disease entities, e.g., the Jo-1 antibody is highly specific for the antisynthetase syndrome.

Swallowing is a complex neuromuscular process that requires the precise motor coordination of the oropharynx, larynx and esophagus [1,2]. While smooth muscles are located in the lower and middle part of the esophagus, the upper part and the oropharynx consist of striated skeletal muscle tissue [2], which is typically affected by inflammation in IIM. It is therefore not surprising that myositis can cause dysphagia via inflammatory involvement of the swallowing muscles. In fact, dysphagia is part of the current American College of Rheumatology/European League Against Rheumatism (ACR/EULAR) diagnostic criteria as an item indicating IIM in patients with symptoms of myalgia [3]. Instrumental assessments, e.g., flexible endoscopic evaluation of swallowing (FEES) or videofluoroscopy (VFSS) are considered the diagnostic gold standard [4,5].

The study data available on dysphagia in IIM are heterogeneous with partly conflicting results, e.g., the reported prevalence rates range from 0% [6] to 100% [7]. Similarly, heterogeneous study results can be found in the instrumental characterization of dysphagia, its consequences or therapeutic implications. The aim of this systematic review was therefore to summarize and analyze the existing evidence on epidemiology, pathophysiology, outcome and therapeutic effects and to estimate pooled prevalence rates in a meta-analysis.

## 2. Methods

### 2.1. Review

#### 2.1.1. Inclusion and Exclusion Criteria in the Review

Studies had to meet the following inclusion criteria:

1. Cohort: the article had to report on dysphagia in at least one subject with IIM. If the cohort included less than five subjects, it had to be stated that diagnostic criteria of definitive or probable IIM according to either Bohan and Peter [8,9], Griggs [10], Needham and Mastaglia [11], the European Neuromuscular Center [12] or the ACR/EULAR criteria [3] were met. If this was not the case, articles were only included if based on the information provided the current ACR/EULAR criteria [3] for definitive or probable IIM were met or if the diagnosis was confirmed by muscle biopsy.
2. Topic: the articles had to report on at least one of the following topics:
  - Epidemiology or prevalence of dysphagia in a population with a minimum of five subjects;
  - Pathophysiology of dysphagia;
  - Outcome of a patient cohort with dysphagia;
  - Therapeutic effects on dysphagia or swallowing.

Articles were excluded if:

1. Patients had other diseases associated with dysphagia, e.g., myasthenia gravis. However, this exclusion criterion was not applied to diseases associated with IIM such as rheumatological diseases in case of overlap syndromes;
2. They exclusively reported on gastroesophageal reflux as manifestation of dysphagia;
3. Dysphagia was reported exclusively as manifestation of structures distal to the esophagus;
4. Conflicting results were reported within the article (e.g., differing prevalence rates).

#### 2.1.2. Search Strategy

To identify studies, MEDLINE was searched for all relevant articles on dysphagia and myositis from inception to January 2020 (last update in January 2020). The following PubMed search algorithm was used:

(“deglutition disorders”(MeSH Terms) OR (“deglutition”(All Fields) AND “disorders”(All Fields)) OR “deglutition disorders”(All Fields) OR “dysphagia”(All Fields)) AND ((“myositis”(MeSH Terms) OR “myositis”(All Fields)) OR (“polymyositis”(MeSH Terms) OR “polymyositis”(All Fields)) OR (“dermatomyositis”(MeSH Terms) OR “dermatomyositis”(All Fields)) OR (“myositis, inclusion body”(MeSH Terms) OR (“myositis”(All Fields) AND “inclusion”(All Fields) AND “body”(All Fields)) OR “inclusion body myositis”(All Fields) OR (“inclusion”(All Fields) AND “body”(All Fields) AND “myositis”(All Fields))) OR (“Antisynthetase syndrome”(Supplementary Concept) OR “Antisynthetase syndrome”(All Fields] OR “antisynthetase syndrome”(All Fields)))”.

Furthermore, reference lists of published articles were screened for additional studies.

### 2.2. Meta-Analysis

#### 2.2.1. Inclusion and Exclusion Criteria in the Meta-Analysis

All studies that reported the prevalence of dysphagia in a cohort of a minimum of five subjects were included in the meta-analysis (Document S1). Only studies that reported directly on a cohort were included (no survey data with estimates of prevalence among physicians). If both instrumental and clinical results were available, the results of the instrumental diagnostics were used. If studies at the same institution had recruited subjects during overlapping periods, only the study with lowest bias risk (Section 2.2.2) was included, or, in case of equal bias risk, the study with the larger sample was included. An equivalent procedure was applied to overlapping cohorts of registry studies or precursor cohorts of a registry. If studies at the same institution did not report an overlapping recruitment period, studies were excluded only if one of the studies stated that all available patients at the institution were included. If studies reported on an identical patient cohort with the same bias risk and sample size, the study that allowed for more subgroup analyses was included.

In addition to the total cohort of IIM, pooled prevalence for dysphagia was estimated in the PM, DM and IBM subgroup and in the subgroup of studies with low bias risk regarding study cohort and dysphagia assessment (Section 2.2.2). Also, the pooled prevalence was estimated for cancer associated myositis, and non-cancer associated myositis in all studies that compared these two groups. All studies on myositis associated/specific antibodies were reviewed to determine whether dysphagia was reported to be associated with (or with the absence of) a specific antibody. If two or more studies compared the prevalence in a population with one of these reported antibodies to a population without the respective antibody, pooled prevalence was again estimated in both of these groups. Studies in the subgroup analysis were only included, if the sample size of the subgroup contained a minimum of five subjects.

#### 2.2.2. Bias Risk in Individual Studies

In all studies included in the meta-analysis, the bias risk was assessed according to the two domains relevant for observational studies, “study participation” and “outcome measurement” of the “Quality in Prognosis Studies Tool” [13]. The domains were adapted to the topic of dysphagia, e.g., in the outcome measurement it was evaluated if studies relied on an instrumental gold-standard assessment including the pharyngeal phase of swallowing. The aim was to evaluate if the presence or absence of oropharyngoesophageal involvement had been assessed by an objective procedure and that dysphagia had not been determined by clinical examination or the presence of symptoms alone. The following criteria were evaluated:

Study participation criteria: (1) Study population represents the total population of IIM or one of its subgroups (DM, PM, IBM, JDM etc.) without additional clinical, demographic or diagnostic criteria, e.g., not only subjects with specific diagnostic procedure or additional clinical hallmark. Excluded from this were clinical criteria, which exclusively represented the contraindications of the instrumental diagnostics used. (2) Adequate description of recruitment: Either a defined period of time at a particular institution/region had to be specified, or it had to be evident that all available patients of an institution/region were included. (3) Adequate description of inclusion and exclusion criteria.

Outcome measures: (1) A clear definition of dysphagia or swallowing pathologies assessed is provided. (2) Dysphagia was assessed with an instrumental gold-standard procedure (flexible endoscopy of swallowing, VFSS, real-time MRI, scintigraphy) that includes the visualization of the pharyngeal phase of swallowing. (3) Identical method and setting of outcome measurement was applied for all study participants.

All points in this list had to have been fulfilled for a study to be classified as “low bias risk”. If there was no indication of bias risk, the study was classified as “low bias risk”, otherwise the study was classified as “significant bias risk”.

#### 2.2.3. Statistical Analysis

A random effect model (restricted maximum likelihood) was chosen to estimate the pooled prevalence rates. The effect size and standard deviation was calculated with Microsoft Excel 16 using the following approach [14]: If no patient had dysphagia in a study population (0 events), a “continuity correction” of 0.5 was added to the event column as well as to the sample size column to enable inverse variance weighting [15]. The further analysis was calculated with the software JASP 0.11.1. The pooled prevalence, the 95% confidence interval (CI), I^2^ as a measure for heterogeneity and a funnel plot with the Egger’s test as a measure for publication bias were calculated for each analyzed group. In the comparison of subjects with a parameter to subjects without the respective parameter (Section 2.2.1), prevalence rates were considered to be significantly different when the 95% CI did not overlap.

## 3. Results

**Figure 1:**
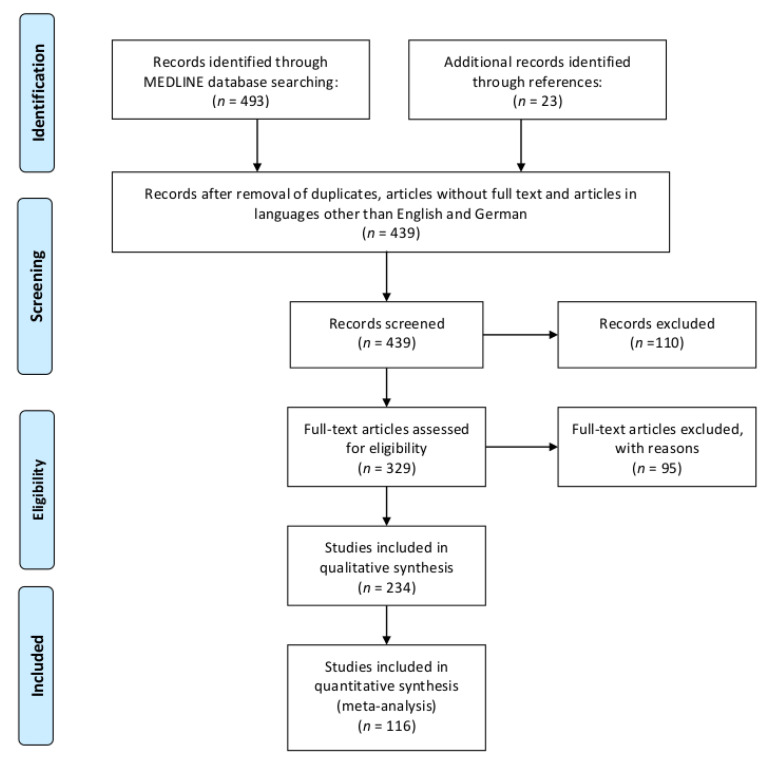
Preferred Reporting Items for Systematic Reviews and Meta-Analyses (PRISMA) flow diagram of the reviewed literature.

Figure 1 illustrates the Preferred Reporting Items for Systematic Reviews and Meta-Analyses (PRISMA) flow diagram of the reviewed literature [16].

### 3.1. Review

A total of 139 articles reported on epidemiology and prevalence (Table S1), 101 articles on pathophysiology (Table S2), 34 articles on the outcome (Table S3) and 93 articles on therapeutic effects (Table S4).

#### 3.1.1. Epidemiology

##### 3.1.1.1. Dysphagia and Disease Course

In principle, IIM is a chronically progressive disease, but sometimes there are also relapsing–remitting episodes. The situation is similar with dysphagia in IIM. Besides relapsing–remitting episodes of dysphagia, several authors report that the prevalence of dysphagia increases as the disease progresses [17,18,19,20,21,22,23,24,25,26]. Nevertheless, dysphagia can also be the initial [17,18,19,20,21,23,24,25,26,27,28,29,30,31,32] or even the only symptom [18,29,31,33]. Therefore, dysphagia should not be considered a late symptom in IIM. Indeed, IIM might be the underlying disease in patients with unclear dysphagia, even if other investigations, such as laboratory results and electrophysiology, do not refer to IIM [32].

##### 3.1.1.2. Factors Associated with Dysphagia

Several factors are reported to be associated with dysphagia. Among the subgroups, differences in prevalence are found: Higher prevalence is reported in DM compared to PM [34,35,36,37] but also vice versa [38], in IBM compared to other forms of IIM [39] and in overlap syndromes compared to other forms of IIM [39]. In addition, an increased risk of dysphagia is reported in patients with associated malignancy [37,40,41,42,43,44,45]. A number of antibodies are also linked to an increased risk of dysphagia: NXP2 [46,47,48,49], FHL-1 [50], SAE [47,51], HMGCR [47,52], NT5c1A [53], SRP [47,54,55], TIF1y [44,47], OJ [56] and myositis-specific or -associated autoantibodies in general [47]. ANA and MDA5 antibodies are reported to be associated with a reduced risk of dysphagia [47,57].

#### 3.1.2. Pathophysiology

##### Inflammation of Swallowing Muscles

IIM can result in impairment of the oral [29,58,59,60,61,62,63,64], pharyngeal [7,23,24,27,29,31,32,33,44,58,59,60,61,62,63,64,65,66,67,68,69,70,71,72,73,74,75,76,77,78,79,80,81,82,83,84,85,86,87,88,89,90,91,92,93,94,95,96,97,98,99,100,101,102,103,104,105,106,107,108,109,110,111,112,113,114,115,116] and esophageal [24,29,38,59,62,73,81,85,89,90,93,95,98,99,101,103,104,105,112,114,115,116,117,118,119,120,121,122,123,124,125,126,127,128,129,130,131] phases of swallowing and pharyngeal dysfunction is associated with aspiration [27,29,31,32,33,58,60,61,70,76,77,78,79,81,83,84,86,87,88,89,91,93,94,96,97,98,102,105,106,108,111,113]. Results from studies and case-reports with biopsies suggest that inflammatory involvement occurs in the affected swallowing muscles [29,31,32,86,90,91,94,95,96,97,100,101,103,104,108,126,132,133], similarly to the well-known inflammatory reactions in the peripheral skeletal muscles in IIM. Interestingly, such changes also seem to occur in smooth muscle tissue of the esophagus [104,119,126]. Besides muscle biopsy, signs for inflammation can be detected by characteristic MRI findings, e.g., edema in the oropharynx [74,134,135,136]. However, presumably due to the small volume of the respective muscles, MRI findings are inconclusive and, if normal, cannot rule out myositis as cause of dysphagia [33]. The study data is conflicting on whether dysphagia is related to the clinical impairment of the peripheral skeletal muscles. Some studies report a correlation of peripheral symptoms with dysphagia [17,39,127], while other studies report the opposite [137,138].

##### Dysphagia Pathology

**Figure 2:**
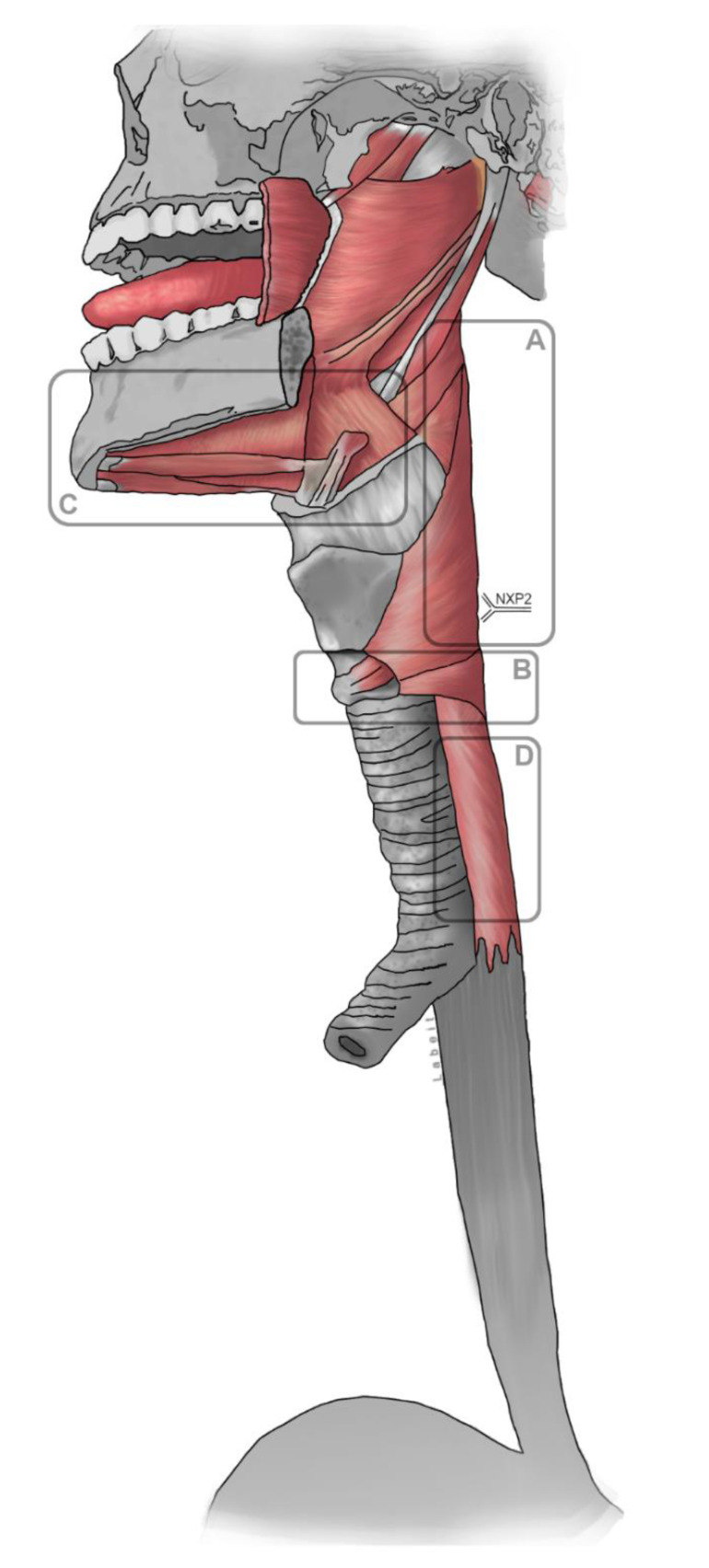
Skeletal swallowing muscles and the associated dysphagia mechanisms: A: Reduced pharyngeal contractility; B: Cricopharyngeal dysfunction; C: Reduced laryngeal elevation; D: Esophageal hypomotility.

In general, four patterns of swallowing impairment can be distinguished depending on the muscle groups affected (illustrated in Figure 2): Reduced pharyngeal contractility, cricopharyngeal dysfunction, reduced laryngeal elevation and esophageal hypomotility. In case of unclear dysphagia, knowledge of these mechanisms and the corresponding findings in instrumental dysphagia assessment can be helpful in the differential diagnosis [33].

Reduced pharyngeal contractility can result in insufficient pharyngeal bolus clearance [139]. Consequently, myositis patients often show pharyngeal residue after swallowing [7,27,29,31,33,44,58,59,60,61,65,66,67,69,71,73,74,75,76,77,78,79,80,83,84,86,87,91,93,96,100,101,103,104,106,111,112,115]. These can impact on swallowing safety and ultimately cause aspiration, which is a frequently reported finding [33,78,81,86]. Further findings indicating reduced pharyngeal contractility are absent or inadequate peristalsis or bolus propulsion [27,31,69,95,106,111], inadequate pharyngeal contraction [29,61,75,87,100,116,126], nasal regurgitation due to velopharyngeal insufficiency [31,44,62,74,79,80,97,98,104,109,110,111,116], piecemeal deglutition when swallowing larger boluses [82,140] or reduced pharyngeal pressure in manometry [7,29,32].

Numerous authors reported a dysfunction of the upper esophageal sphincter (UES) due to cricopharyngeal impairment [7,23,24,27,29,31,32,38,58,61,63,66,68,71,72,77,78,80,82,86,87,89,90,91,92,93,94,96,97,100,101,102,106,108]. Both, hypercontractility, e.g., a relaxation deficit of the UES [7,23,24,31,58,61,63,77,78,80,82,86,87,89,93,94,96,97,100,102,106,108], and hypocontractility [24,38,82,85,90,98,114] have been described. This may be explained by the fact that muscle physiology is affected in a different way during the acute inflammatory phase compared to the chronic phase when fibrosis occurs [82]. Cricopharyngeal hypercontractility often leads to an opening or relaxation disorder of the UES, resulting in pronounced residue or pooling of saliva in the piriform sinus [7,27,31,33,65,66,67,69,73,74,76,77,78,80,86,87,101,103,106,111,112,115], which is located directly above the UES. Typical findings in VFSS are a prominent cricopharyngeus muscle, also referred to as cricopharyngeal bar [23,32,61,68,86,91,93,97,99,106] and muscle propulsions or posterior indentations between C3 and C7 [7,71,96].

Another common finding in myositis is reduced laryngeal elevation [29,58,63,66,76,77,83,107,111]. This is probably caused by impaired contractility of the suprahyoid and longitudinal pharyngeal muscles [141]. Laryngeal elevation is a prerequisite for the UES to open [142] and reduced laryngeal elevation can lead to functional UES impairment. Therefore, the findings in dysphagia diagnostics can be similar to findings with primary UES disorder. Some studies suggest that the typical myositis-associated finding of residue in the piriform sinus may be primarily caused by reduced pharyngeal contractility of suprahyoid muscles rather than an actual dysfunction of the cricopharyngeus muscle itself [58,77]. In this context, one could speak of a pseudocricopharyngeal dysfunction due to reduced laryngeal elevation.

Various authors reported reduced or absent esophageal motility sometimes extending to the lower esophageal sphincter [29,38,114]. Most studies used manometry [24,29,38,59,85,90,98,105,114,117,118,119,122,129,130] some also VFSS, barium swallow or scintigraphy [62,73,81,89,99,104,120,121,124,125,127] to detect esophageal impairment.

#### 3.1.3. Outcome

Dysphagia in patients with IIM not only affects quality of life [71], but is also associated with severe complications such as weight loss [30,138] or aspiration pneumonia [24,29,30,32,68,116,143,144,145,146]. Pneumonia after aspiration is particularly dangerous as this condition can be fatal [24,29,30,32,116,143,144,145,147]. The rates of pneumonia/aspiration pneumonia in cohorts with dysphagic patients are reported between 6% and 36% [24,30,32,68,99,116], are four times more prevalent and, thus, significantly higher in dysphagic than in non-dysphagic patients [24]. Some studies report that aspiration pneumonia is the leading cause of death [29,30,32,147]. In fact, a survey-based study among physicians on patient cases with IBM suggests that dysphagia-associated complications may even be the only cause of premature mortality [148]. It is therefore not surprising that dysphagia is associated with increased mortality [34,144,145,149]. Conversely, the survival rate is associated with dysphagia recovery [143]. Nevertheless, some studies also reported no association between dysphagia and mortality [25,35,150]. Besides mortality, dysphagia is associated with a worse functional status or general condition of the disease [24,39,110,151,152,153] and represents a negative predictive factor for further disease progression [151].

#### 3.1.4. Therapy

##### Immunomodulatory Therapy

There are several articles reporting positive therapeutic effects of immunomodulatory medication on symptoms and/or on findings of objective swallowing evaluations. These include intravenous methylprednisolone pulse therapy [19,33,70,81,87,134,154,155,156,157,158,159], methotrexate [30,76,89,124,136,154,159,160,161], long-term prednisone/prednisolone [29,30,33,59,64,70,74,76,81,83,84,87,89,101,103,111,118,122,124,134,136,143,154,158,159,161,162,163,164,165,166,167], azathioprine [29,30,33,83,84,87,155,158,163], intravenous immunoglobulin (IVIG) [24,30,44,65,69,70,73,76,78,81,85,87,114,117,118,121,136,155,161,168,169,170,171,172,173,174,175,176], subcutaneous immunoglobulin [173,177,178], hydroxychloroquine [30,118,154], tacrolimus [162,172], cyclophosphamide [83,170,172], mycophenolate mofetil [118,179], cyclosporine [123] and rituximab [155]. If dysphagia does not respond to medical therapy, it may be helpful to switch to another group of medication, e.g., from steroids to IVIG [24]. The effects on swallowing function were reported in all forms of IIM including IBM [176].

##### Therapy of Malignancy

IIM is associated with malignant diseases and can occur as paraneoplastic syndrome. Therefore, treatment of malignancy can improve muscle symptoms. This effect is also described for dysphagia [44]. Both tumor resection [180,181,182] and chemotherapy [182,183,184] can improve or relieve impaired deglutition.

##### Non-Pharmacological Interventional Therapy

Non-pharmacological interventional therapies are symptomatic strategies without a modulatory effect on the disease course, aiming to improve swallowing physiology. Preliminary data suggest that the pneumonia rate can be reduced by interventional therapy if aspiration is reduced [68]. To date, all non-pharmacological interventional procedures attempt to relieve or eliminate the symptoms of cricopharyngeal dysfunction. Three different procedures have been reported:

Injection of botulinum toxin A in the cricopharyngeus muscle: This procedure can reduce the pressure in the UES [61,86] which may result in both symptom relief [61,68] and improvement in objective swallowing diagnostics [68,72]. The effect of this treatment usually lasts for a few months, hence repetitive treatments are necessary [72]. Some authors also reported no improvement [29].

Cricopharyngeal dilatation: This procedure is usually performed endoscopically via a balloon catheter. A clinical improvement of symptoms [29,30,32,80,102] as well as improvement in objective dysphagia diagnostics [78,80] have been described. Here, too, the effect may not be permanent, so that repetitive treatments may become necessary [32,80,102].

Cricopharyngeal myotomy: This is a non-reversible intervention with a surgical sectioning of the cricopharyngeus muscle. It can lead to an improvement of symptoms [24,29,30,31,32,86,91,96,97,100,102,108,110] and an improvement in objective swallowing diagnostics [91,96,97,100]. In some cases, improvement of symptoms without corresponding improvement in VFSS were reported [29]. Other articles reported improvement in swallowing diagnostics without benefits being perceived by the patients [91].

In the absence of interventional trials with clinically meaningful endpoints, the available studies do not allow for a direct comparison between these treatment options and related treatment-specific recommendations.

##### Behavioral Therapy

In myositis patients, various behavioral swallowing therapies such as diet modifications, compensatory techniques and exercises are used [29]. Unfortunately, there is little evidence for these techniques as there are few studies investigating behavioral therapy in IIM. In individual cases, it was reported that the Mendelson maneuver (pressing the back of the tongue against the palate when swallowing) has helped to maintain oral food intake without aspiration pneumonia or weight loss [29]. In addition, a case report suggests that isometric tongue strengthening has contributed to the maintenance of posterior tongue pressure [60].

### 3.2. Meta-Analysis

**Figure 3:**
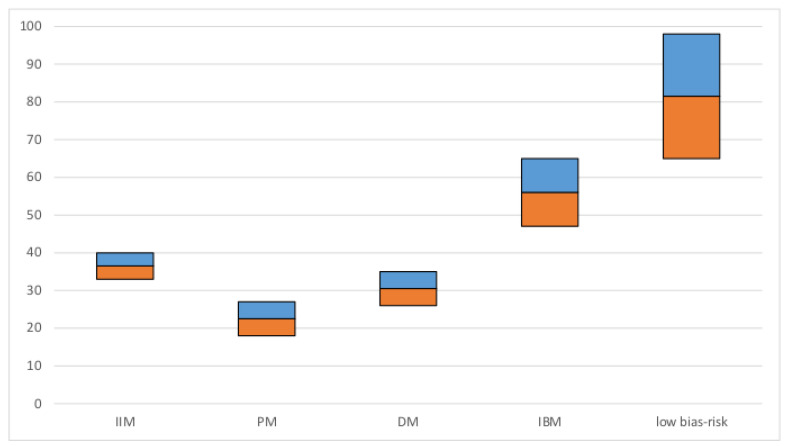
Pooled estimated prevalence of dysphagia: prevalence in % (y-axis): the blue and orange bars represent the 95% confidence interval; IIM: Idiopathic inflammatory myopathy, PM: Polymyositis, DM: Dermatomyositis, IBM: Inclusion body myositis, low bias risk studies: cohort of studies with low risk of bias.

A total of 109 studies representing 10,382 subjects were included in the meta-analysis of the total patient cohort with IIM. The overall estimate of prevalence of dysphagia was 36%. In patients with IBM, a particularly high prevalence of 56% was estimated. No significant differences in prevalence were found between PM and DM (prevalence and CIs are visualized in Figure 3).

**Figure 4:**
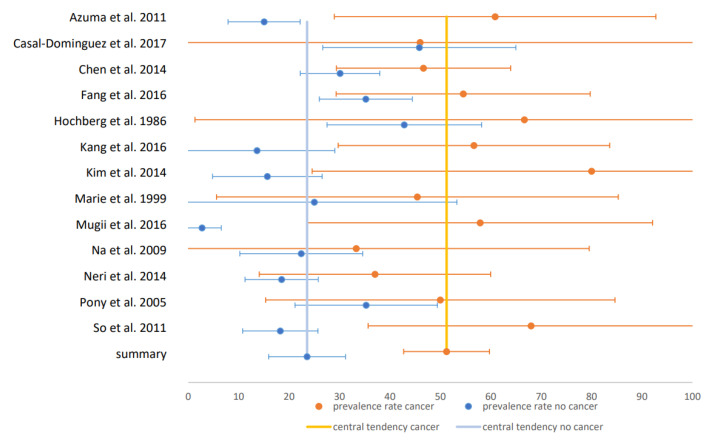
Forest plot for malignancy: Forest plot of the studies comparing prevalence in cancer and non-cancer-associated IIM: x-axis shows the prevalence in %.

**Figure 5:**
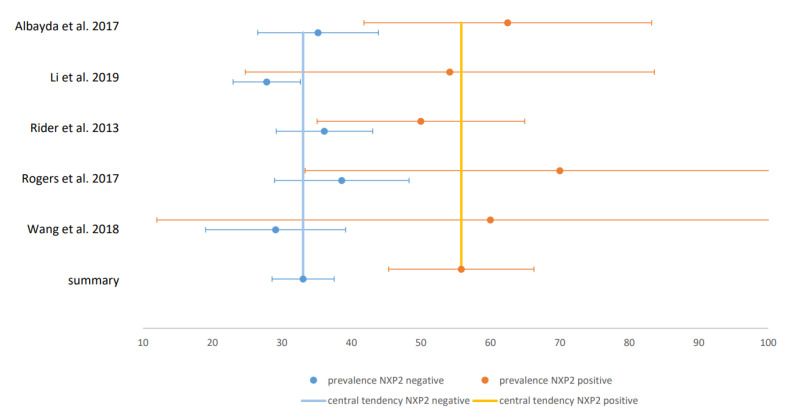
Forest plot for NXP2: Forest plot of the studies comparing prevalence in NXP2-positive and -negative IIM: x-axis shows the prevalence in %.

Only six studies were classified as “low bias risk”. In those studies, all with gold-standard instrumental assessments of dysphagia, the prevalence estimate was 82% and thus clearly higher compared to the total cohort. The estimate of dysphagia prevalence in non-cancer-associated IIM was 26% and 52% in cancer-associated IIM. In patients with NXP2-negative IIM, the estimated prevalence was 33% and 56% in patients with NXP2 antibodies. The CIs in these two comparative analyses did not overlap, so that a significant difference between patients with and without malignancy and NXP2-antibodies can be assumed. The forest plot for studies on malignancy is illustrated in Figure 4 and for studies on NXP2-antibodies in Figure 5. All other comparisons between patients with and without specific antibodies did not reveal significant differences in prevalence. Therefore, of the risk factors presented in Section 3.1.1.2, only malignancy and NXP2 antibodies could be confirmed in our meta-analysis. The estimate of the pooled prevalence, the 95% CI, the number of included studies, the number of included subjects, I^2^ as measure for heterogeneity, the *p*-value of the Egger’s test as measure for publication bias and the percentage of studies with low bias risk for all analyses are shown in Table 1. The included studies with prevalence and CI in forest and funnel plots for all analyses are shown in the Supplementary Materials S5.

## 4. Discussion

Dysphagia is a frequent complication in IIM with an estimated pooled prevalence of 36% and a peak prevalence of 56% in IBM. Due to the worse outcome associated with dysphagia and the fact that standard immunomodulatory therapy as well as interventional treatment options can improve swallowing impairment, we propose to systematically evaluate swallowing function in patients with IIM and, if present, to include dysphagia as a therapeutic target. The association with malignancy and NXP2 antibodies may have diagnostic relevance in two ways: On the one hand, dysphagia should be considered early on in patients with these risk factors and therefore initiate instrumental swallowing assessment for detailed analysis. On the other hand, in patients with proven dysphagia it might be particularly relevant to carefully look for the presence of an associated malignancy, as dysphagia was shown to be associated with malignant comorbidities [40].

The fact that specific antibodies are associated with an increased risk of swallowing impairment could be an indication that specific pathophysiologic mechanisms might be prone to the oropharynx or the esophagus. The NXP2 antibody associated with dysphagia in this study is particularly common in patients with juvenile dermatomyositis [185]. In the studies on NXP2 antibodies included in our meta-analysis, there was one study in which only juvenile IIM was investigated [49], and another study in which juvenile IIM patients were included in addition to adult patients [47]. The remaining three studies were conducted in adult patients. In addition, the antibody is associated with calcinosis and in adult patients possibly also with malignancy [185]. Thus, an association with dysphagia may also seem possible by association with malignancy which, in turn, is associated with dysphagia. A connection between dysphagia and calcinosis also seems possible, although we did not find a supporting mechanistic explanation for this connection in the literature. However, other antibodies such as TIF-1y, for which in this study no increased prevalence of dysphagia could be proven, are also associated with malignancy [185] (although individual studies associate TIF-1y to dysphagia). Furthermore, in one of the studies on NXP2 antibodies from our meta-analysis, no association with malignancy and calcinosis in adult patients was found [186]. A higher prevalence of dysphagia is also observed in malignancy with compared to malignancy without active IIM [43]. This suggests that dysphagia is not due to an unspecific general deterioration caused by the malignant disease alone. Specific paraneoplastic immune-mediated mechanisms might therefore contribute to swallowing dysfunction. Further, the reported cases of isolated dysphagia (Section 3.1.1.1) might, similarly to orbital myositis [187], represent a distinct inflammatory entity.

A higher prevalence of dysphagia of 82% was estimated in the low bias risk studies with instrumental assessment. This finding corroborates previous studies showing that refined instrumental evaluation is more sensitive for detecting dysphagia than clinical testing [188,189]. Further, this suggests that oropharyngoesophageal dysfunction may also be present in patients who subjectively experience no swallowing complaints and, therefore, do not report symptoms of dysphagia [7,27,138]. Consistent with this, silent penetration and aspiration (clinically unapparent without symptoms, e.g., coughing or dyspnea) are reported in patients with IBM [71]. The reported prevalence rates vary widely which is also reflected by the strong heterogeneity of the overall cohort. There are four main explanations for these inconsistencies: (1) IIM is not a uniform disease but instead represents a heterogeneous group of diseases with different pathophysiologic mechanisms. Thus, there are presumably real differences in prevalence between different subgroups of the disease. If this is the case, heterogeneity in a meta-analysis should decrease when individual disease groups are analyzed separately; (2) Many different definitions of dysphagia were used, e.g., oropharyngeal vs. esophageal dysphagia. The prevalence rates of the different forms of dysphagia may differ; (3) Different forms of assessment of dysphagia were used, e.g., clinical (patient chart review, swallowing examination) vs. instrumental (FEES, VFSS, manometry, scintigraphy, real-time swallowing MRI). If this is a cause of different prevalence rates, heterogeneity in a meta-analysis should decrease when studies using a uniform assessment procedure are analyzed separately; (4) Dysphagia was determined at different points in the course of the disease (Section 3.1.1.1). Indeed, the heterogeneity partly decreased in the subgroup analysis of IBM, PM and DM and disappeared in the analysis of low bias risk studies with instrumental assessment. Therefore, the heterogeneity in the overall cohort seems to be due to both the different definitions and assessments of dysphagia and real differences in the investigated patient cohorts with differing pathophysiology. The low heterogeneity in most subgroup analysis with specific antibodies may indicate that in case of uniform pathophysiology prevalence rates converge.

Both the funnel plot and the Egger’s test suggest that there was a publication bias in our overall cohort, i.e., studies with small sample sizes show higher prevalence rates than studies with large sample sizes. If the bias risk of individual studies is taken into account, an alternative conclusion emerges: Prospective studies with instrumental procedures generally had a smaller sample size, presumably due to the increased recruitment and data collection effort. However, they reported higher prevalence rates due to more sensitive and high-quality diagnostic procedures. In line with this explanatory approach, the funnel plot and the Egger’s test no longer indicate a publication bias when studies with low bias risk are analyzed separately.

There are several limitations to this study that must be considered. First, in the overall cohort of the meta-analysis, only few studies had a low bias risk. Especially in the studies with a significant bias risk, different definitions of dysphagia were used and the classification as dysphagic and non-dysphagic was often based solely on clinical evaluation or symptoms. However, due to the lack of objective swallowing diagnostics, it is not possible to say with certainty whether oropharyngoesophageal dysfunction was actually present in these studies with significant bias risk. This has certainly contributed to the considerable heterogeneity and may have contributed to the publication bias. Second, in the meta-analysis of factors associated with increased risk of dysphagia, only studies comparing the prevalence in groups with and without the respective factors were included. However, several potential factors were reported where no such comparison was possible. Third, the majority of included studies were retrospective observational studies, some with small sample size or even only individual case reports. Thus, many conclusions are based on studies with low quality and evidence levels. This applies in particular to the therapy section, where not a single prospective randomized controlled trial could be included. Fourth, although studies at the same institutions with overlapping recruitment periods were excluded, it is possible that overlapping patient groups may also have occurred between registry studies and studies at individual institutions. Fifth, the review as well as the assessment of the bias risk were conducted by only one observer, which may reduce reliability. Sixth, for the systematic review of this meta-analysis, only Medline was searched with Pubmed, so studies that are only listed in other databases may not have been found. Seventh, due to different reporting standards and partially missing information, no demographic data were pooled and included in the meta-analysis. Especially when comparing groups (e.g., patients with malignant disease and without malignant disease), the groups may differ not only in the prevalence of dysphagia but also in demographic characteristics. For the available demographic data of the studies included in the meta-analysis, we refer to Table S1 (column “cohort”) in the Supplementary Materials.

## 5. Conclusions

Dysphagia is common in patients with IIM, with an estimated overall prevalence rate of 36% and a particularly high prevalence in IBM. Factors with increased risk of dysphagia include malignancy and NXP2 autoantibodies. A refined instrumental assessment is more sensitive to detect dysphagia and should be included in the diagnostic work-up of swallowing impairment. Dysphagia in IIM is caused by inflammatory involvement of the swallowing muscles, which can lead to reduced pharyngeal contractility, cricopharyngeal dysfunction, reduced laryngeal elevation and esophageal hypomotility. In IIM, impaired deglutition can lead to life-threatening complications such as aspiration pneumonia and increasing mortality. Standard immunomodulatory therapy can improve swallowing function and dysphagia should, therefore, be included as a therapeutic target. Further positive therapeutic effects may result from the treatment of malignancy or from interventions targeting the cricopharyngeal muscle such as myotomy, dilatation or botulinum toxin injection.

## Figures and Tables

**Table 1:** The estimate of the pooled prevalence, the 95% confidence interval (CI), the number of included studies and subjects, I^2^ as measure for heterogeneity, *p*-value of the Egger’s test and percentage of studies with low risk of bias for all meta-analyses.

Patient Group	*n*, Studies	*n*, Subjects	Prevalence	CI Lower	CI Upper	I-Squared	*p*-Value Egger’s Test	Low Bias Risk
total cohort	109	10382	36%	33%	40%	87%	>0.01 *	6%
PM	21	882	23%	18%	27%	52%	0.03 *	5%
DM	49	3274	31%	26%	35%	80%	>0.01 *	2%
IBM	23	1352	56%	47%	65%	76%	>0.01 *	22%
low bias risk	6	115	82%	65%	98%	0%	0.70	100%
malignancy+	13	271	51%	43%	60%	0%	0.39	0%
malignancy−	13	1120	23%	17%	30%	85%	0.02 *	0%
NXP2+	5	196	56%	45%	66%	0%	0.42	0%
NXP2−	5	1188	33%	28%	37%	42%	0.22	0%
MDA5+	3	89	12%	0%	23%	61%	0.13	0%
MDA5−	3	538	21%	10%	32%	86%	0.22	0%
SEA+	2	17	76%	35%	100%	0%	n.a.	0%
SEA−	2	589	35%	20%	49%	81%	n.a.	0%
SRP+	3	51	62%	40%	84%	0%	0.69	0%
SRP−	3	943	36%	26%	45%	81%	0.15	0%
TIF1y+	3	103	45%	32%	58%	0%	0.67	0%
TIF1y−	3	519	23%	0%	48%	98%	0.12	0%

* Significant *p*-values.

## Supplementary Materials

The following are available online at https://www.mdpi.com/2077-0383/9/7/2150/s1, Table S1: Articles reporting on epidemiology and prevalence, Table S2: Articles reporting on pathophysiology, Table S3: Articles reporting on outcome; Table S4: Articles reporting on therapeutic effects; Document S1: included studies with prevalence and CI in a forest plot and a funnel-plot for all meta-analysis;
