# Supplementary material for: The Impact of Dysphagia in Myositis: A Systematic Review and Meta-Analysis
Source: J Clin Med. 2020 Jul 8;9(7):2150. doi: 10.3390/jcm9072150 (PMC7408750; doi:10.3390/jcm9072150)

**Meta-Analysis IIM total cohort**

| **Fixed and Random Effects** | | | | | | | |
| --- | --- | --- | --- | --- | --- | --- | --- |
|  | | **Q** | | **df** | | **p** | |
| Omnibus test of Model Coefficients |  | 430.394 |  | 1 |  | < .001 |  |
| Test of Residual Heterogeneity |  | 717.558 |  | 108 |  | < .001 |  |
|  | | | | | | | |
| *Note.*   *p* -values are approximate. | | | | | | | |

| **Coefficients** | | | | | | | | | | | | | |
| --- | --- | --- | --- | --- | --- | --- | --- | --- | --- | --- | --- | --- | --- |
|  | | **Estimate** | | **Standard Error** | | **z** | | **p** | | **Lower Bound** | | **Upper Bound** | |
| intrcpt |  | 0.360 |  | 0.017 |  | 20.746 |  | < .001 |  | 0.326 |  | 0.395 |  |
|  | | | | | | | | | | | | | |
| *Note.*  Wald test. | | | | | | | | | | | | | |

| **Residual Heterogeneity Estimates** | | | | | | | |
| --- | --- | --- | --- | --- | --- | --- | --- |
|  | | **Estimate** | | **Lower Bound** | | **Upper Bound** | |
| *τ²* |  | 0.022 |  | 0.016 |  | 0.037 |  |
| *τ* |  | 0.149 |  | 0.127 |  | 0.191 |  |
| *I²* (%) |  | 87.480 |  | 83.501 |  | 92.014 |  |
| *H²* |  | 7.987 |  | 6.061 |  | 12.522 |  |
|  | | | | | | | |

| **Regression test for Funnel plot asymmetry ("Egger's test")** | | | | | |
| --- | --- | --- | --- | --- | --- |
|  | | **z** | | **p** | |
| sei |  | 7.043 |  | < .001 |  |
|  | | | | | |

**Plot**

**Forest plot**


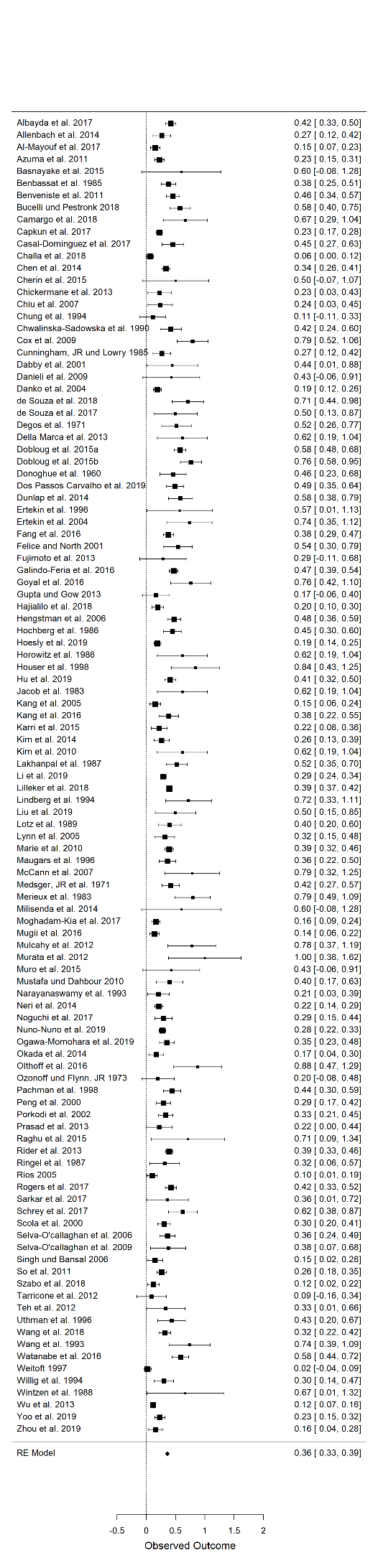


**Funnel plot**


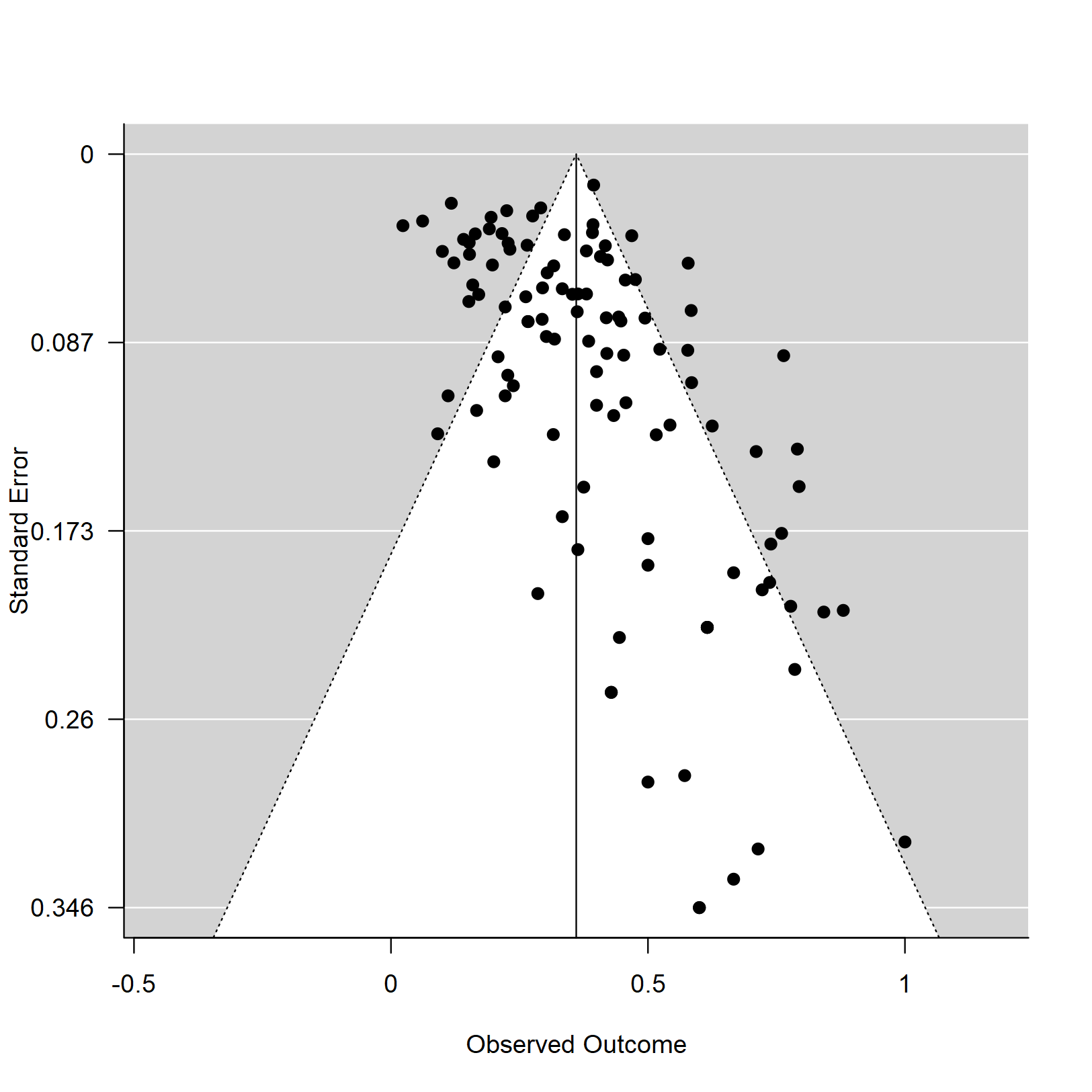


**Meta-Analysis PM**

| **Fixed and Random Effects** | | | | | | | |
| --- | --- | --- | --- | --- | --- | --- | --- |
|  | | **Q** | | **df** | | **p** | |
| Omnibus test of Model Coefficients |  | 85.536 |  | 1 |  | < .001 |  |
| Test of Residual Heterogeneity |  | 42.831 |  | 20 |  | 0.002 |  |
|  | | | | | | | |
| *Note.*   *p* -values are approximate. | | | | | | | |

| **Coefficients** | | | | | | | | | | | | | |
| --- | --- | --- | --- | --- | --- | --- | --- | --- | --- | --- | --- | --- | --- |
|  | | **Estimate** | | **Standard Error** | | **z** | | **p** | | **Lower Bound** | | **Upper Bound** | |
| intrcpt |  | 0.226 |  | 0.024 |  | 9.249 |  | < .001 |  | 0.178 |  | 0.274 |  |
|  | | | | | | | | | | | | | |
| *Note.*  Wald test. | | | | | | | | | | | | | |

| **Residual Heterogeneity Estimates** | | | | | | | |
| --- | --- | --- | --- | --- | --- | --- | --- |
|  | | **Estimate** | | **Lower Bound** | | **Upper Bound** | |
| *τ²* |  | 0.006 |  | 0.001 |  | 0.025 |  |
| *τ* |  | 0.074 |  | 0.032 |  | 0.157 |  |
| *I²* (%) |  | 52.047 |  | 16.859 |  | 82.881 |  |
| *H²* |  | 2.085 |  | 1.203 |  | 5.841 |  |
|  | | | | | | | |

| **Regression test for Funnel plot asymmetry ("Egger's test")** | | | | | |
| --- | --- | --- | --- | --- | --- |
|  | | **z** | | **p** | |
| sei |  | 2.197 |  | 0.028 |  |
|  | | | | | |

**Plot**

**Forest plot**


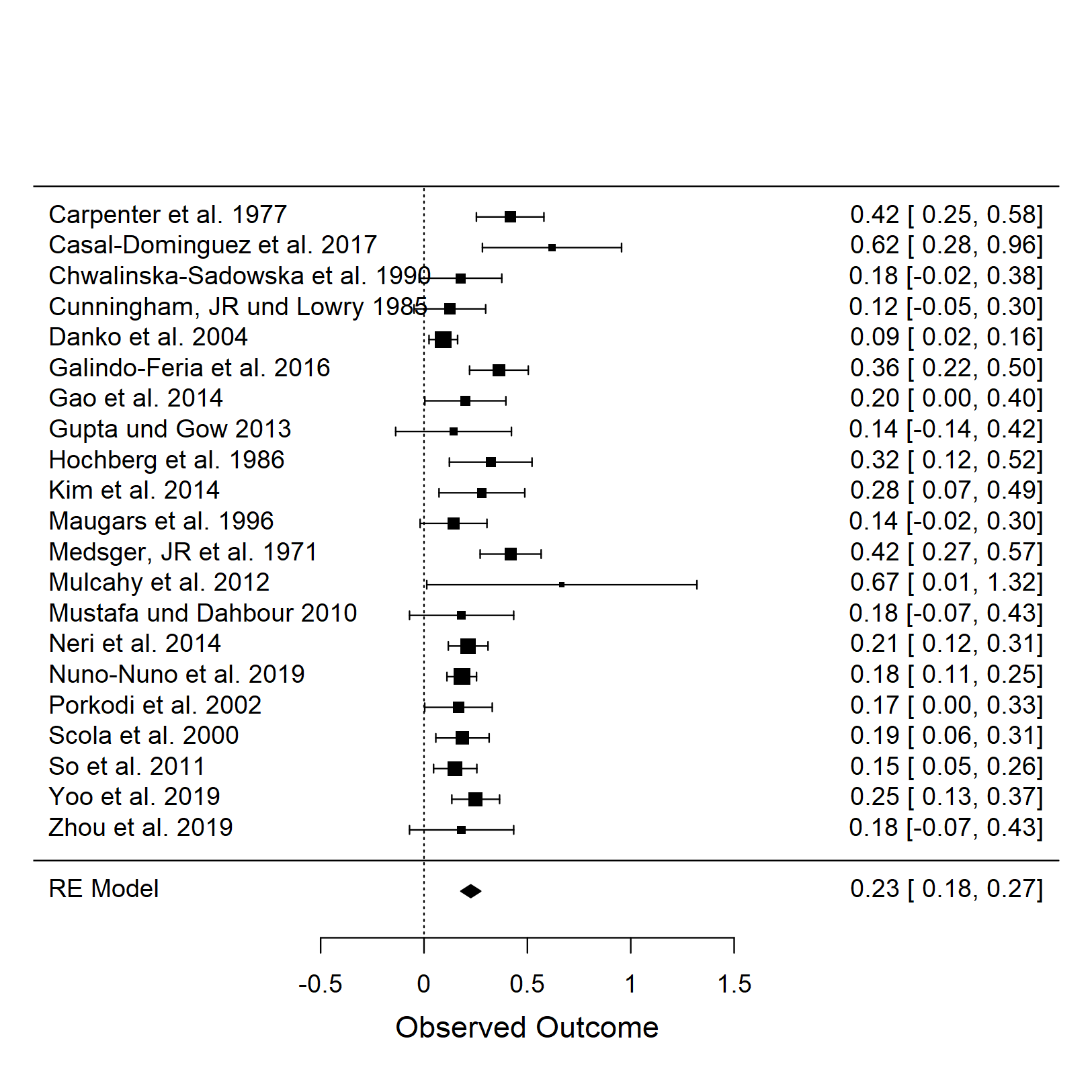


**Funnel plot**


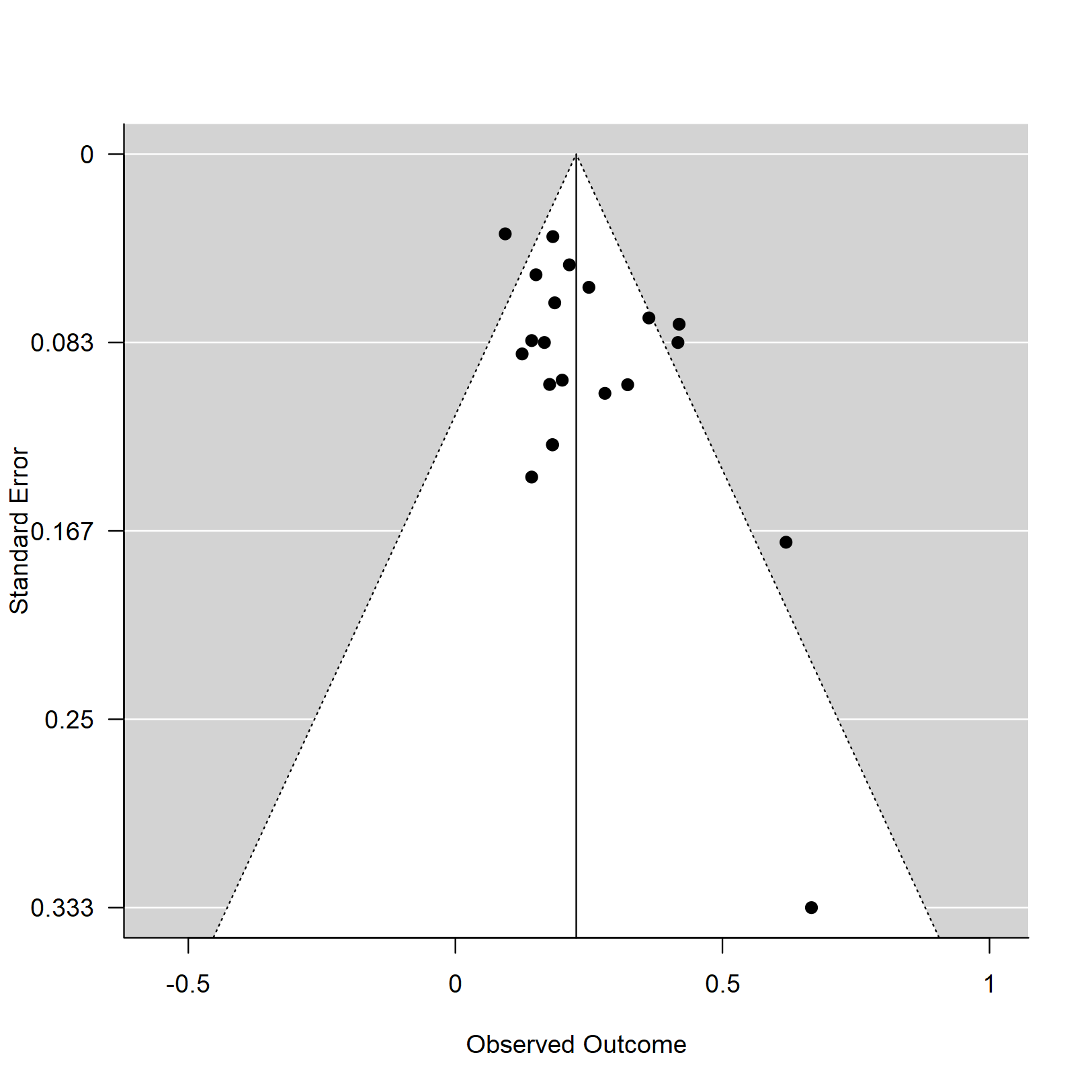


**Meta-Analysis DM**

| **Fixed and Random Effects** | | | | | | | |
| --- | --- | --- | --- | --- | --- | --- | --- |
|  | | **Q** | | **df** | | **p** | |
| Omnibus test of Model Coefficients |  | 191.697 |  | 1 |  | < .001 |  |
| Test of Residual Heterogeneity |  | 258.016 |  | 48 |  | < .001 |  |
|  | | | | | | | |
| *Note.*   *p* -values are approximate. | | | | | | | |

| **Coefficients** | | | | | | | | | | | | | |
| --- | --- | --- | --- | --- | --- | --- | --- | --- | --- | --- | --- | --- | --- |
|  | | **Estimate** | | **Standard Error** | | **z** | | **p** | | **Lower Bound** | | **Upper Bound** | |
| intrcpt |  | 0.307 |  | 0.022 |  | 13.845 |  | < .001 |  | 0.263 |  | 0.350 |  |
|  | | | | | | | | | | | | | |
| *Note.*  Wald test. | | | | | | | | | | | | | |

| **Residual Heterogeneity Estimates** | | | | | | | |
| --- | --- | --- | --- | --- | --- | --- | --- |
|  | | **Estimate** | | **Lower Bound** | | **Upper Bound** | |
| *τ²* |  | 0.015 |  | 0.008 |  | 0.030 |  |
| *τ* |  | 0.123 |  | 0.091 |  | 0.172 |  |
| *I²* (%) |  | 80.018 |  | 68.524 |  | 88.643 |  |
| *H²* |  | 5.005 |  | 3.177 |  | 8.805 |  |
|  | | | | | | | |

| **Regression test for Funnel plot asymmetry ("Egger's test")** | | | | | |
| --- | --- | --- | --- | --- | --- |
|  | | **z** | | **p** | |
| sei |  | 3.841 |  | < .001 |  |
|  | | | | | |

**Plot**

**Forest plot**


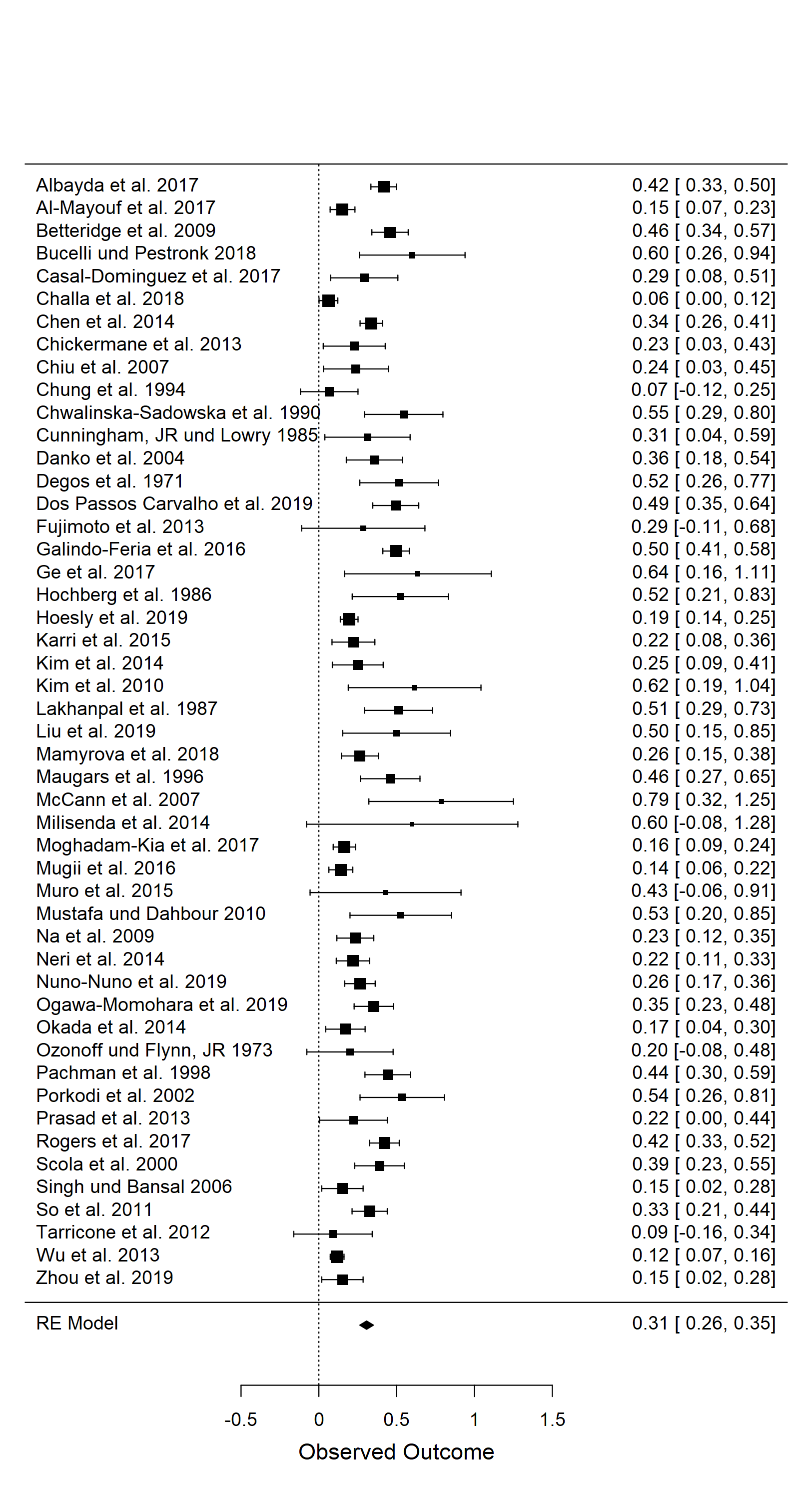


**Funnel plot**


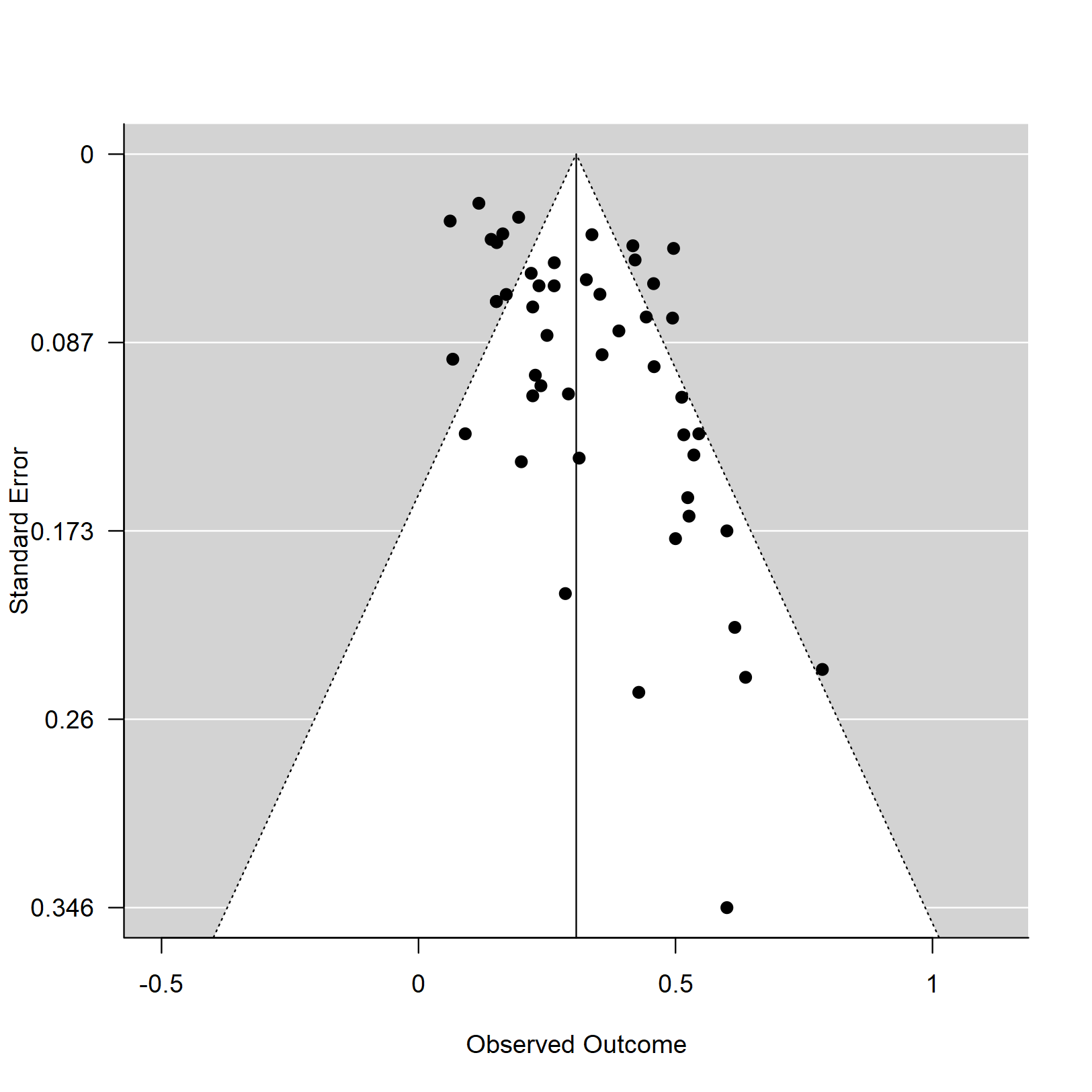


**Meta-Analysis IBM**

| **Fixed and Random Effects** | | | | | | | |
| --- | --- | --- | --- | --- | --- | --- | --- |
|  | | **Q** | | **df** | | **p** | |
| Omnibus test of Model Coefficients |  | 150.441 |  | 1 |  | < .001 |  |
| Test of Residual Heterogeneity |  | 119.322 |  | 22 |  | < .001 |  |
|  | | | | | | | |
| *Note.*   *p* -values are approximate. | | | | | | | |

| **Coefficients** | | | | | | | | | | | | | |
| --- | --- | --- | --- | --- | --- | --- | --- | --- | --- | --- | --- | --- | --- |
|  | | **Estimate** | | **Standard Error** | | **z** | | **p** | | **Lower Bound** | | **Upper Bound** | |
| intrcpt |  | 0.561 |  | 0.046 |  | 12.265 |  | < .001 |  | 0.471 |  | 0.650 |  |
|  | | | | | | | | | | | | | |
| *Note.*  Wald test. | | | | | | | | | | | | | |

| **Residual Heterogeneity Estimates** | | | | | | | |
| --- | --- | --- | --- | --- | --- | --- | --- |
|  | | **Estimate** | | **Lower Bound** | | **Upper Bound** | |
| *τ²* |  | 0.026 |  | 0.008 |  | 0.060 |  |
| *τ* |  | 0.162 |  | 0.090 |  | 0.245 |  |
| *I²* (%) |  | 76.055 |  | 49.395 |  | 87.900 |  |
| *H²* |  | 4.176 |  | 1.976 |  | 8.264 |  |
|  | | | | | | | |

| **Regression test for Funnel plot asymmetry ("Egger's test")** | | | | | |
| --- | --- | --- | --- | --- | --- |
|  | | **z** | | **p** | |
| sei |  | 3.547 |  | < .001 |  |
|  | | | | | |

**Plot**

**Forest plot**


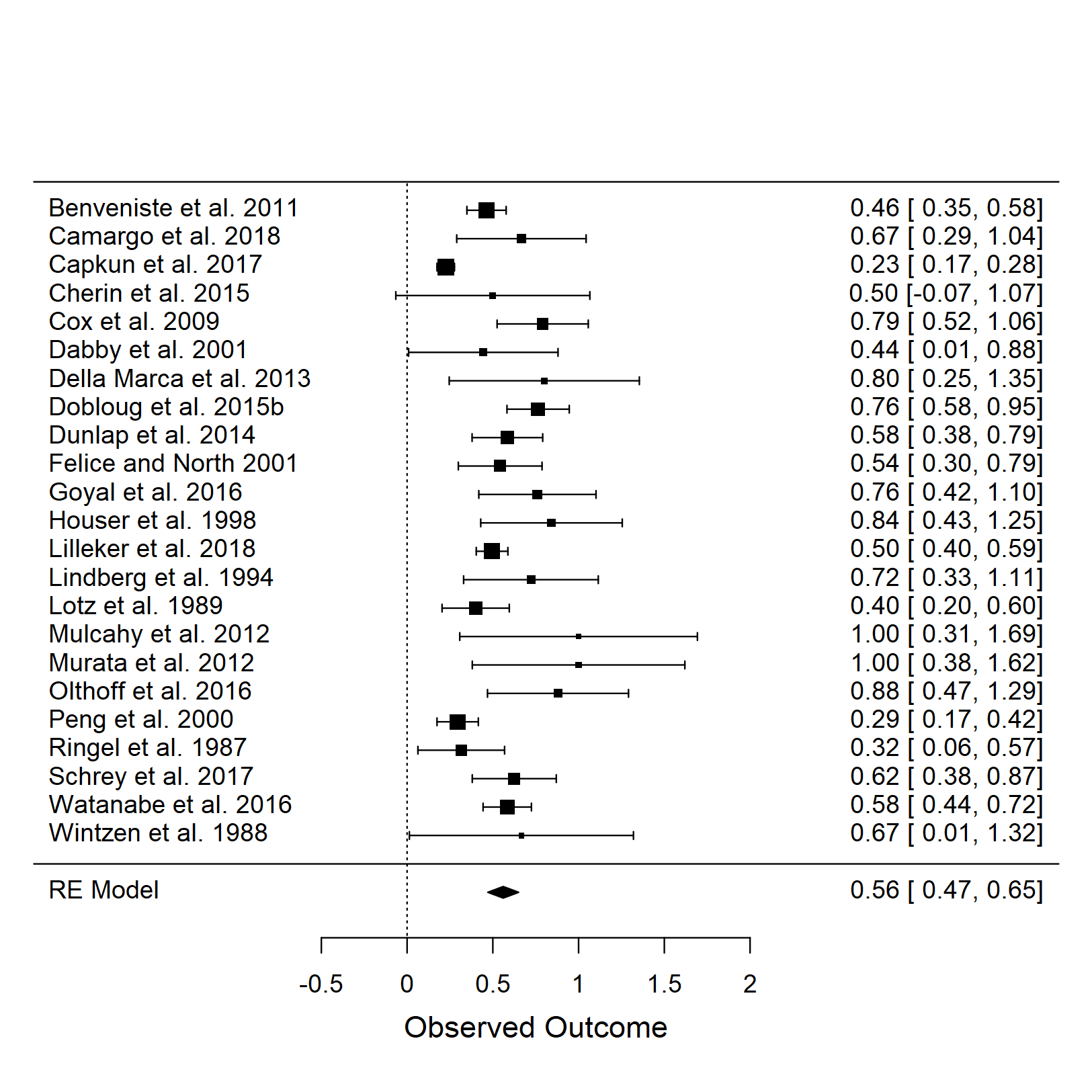


**Funnel plot**


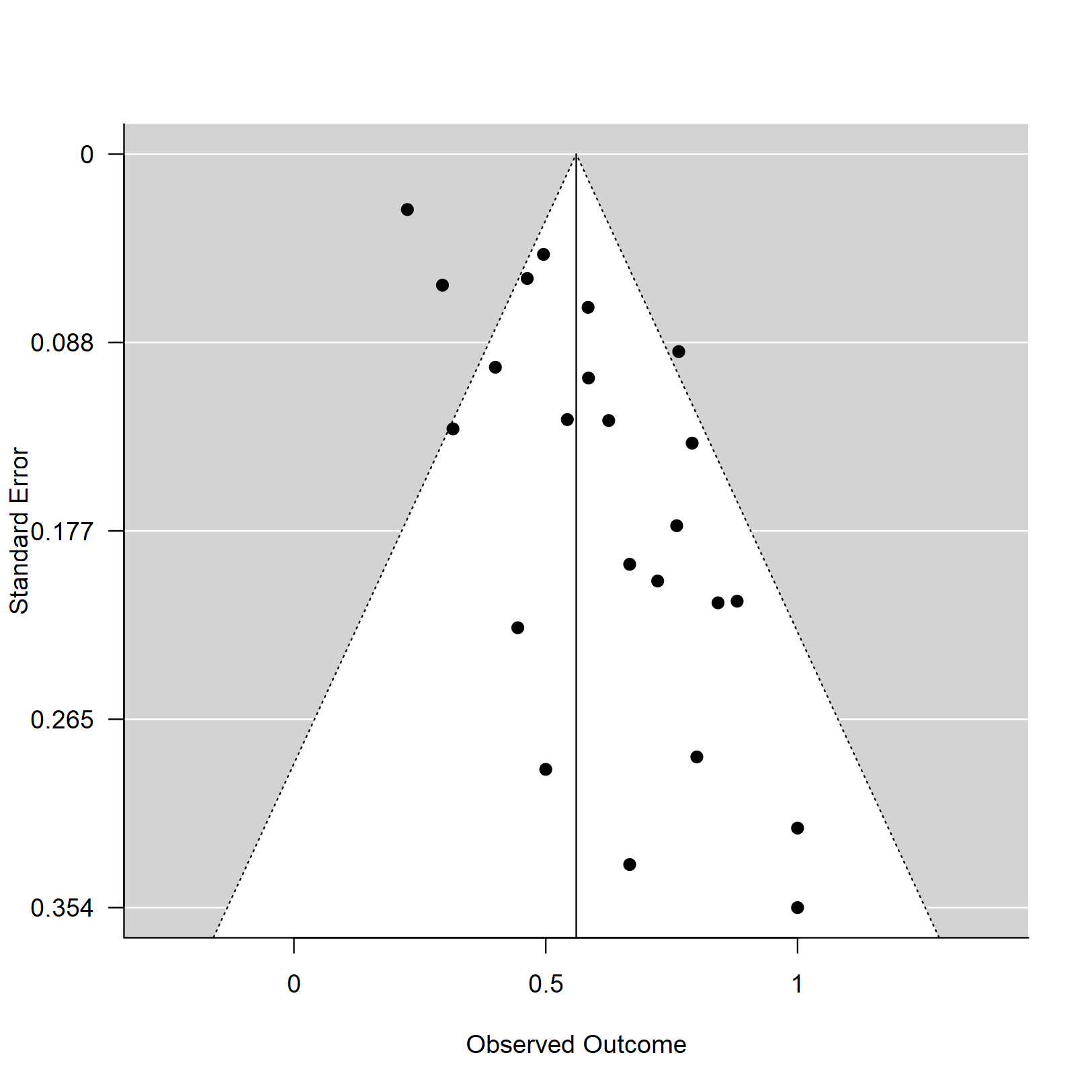


**Meta-Analysis low bias-risk studies**

| **Fixed and Random Effects** | | | | | | | |
| --- | --- | --- | --- | --- | --- | --- | --- |
|  | | **Q** | | **df** | | **p** | |
| Omnibus test of Model Coefficients |  | 94.081 |  | 1 |  | < .001 |  |
| Test of Residual Heterogeneity |  | 0.519 |  | 5 |  | 0.991 |  |
|  | | | | | | | |
| *Note.*   *p* -values are approximate. | | | | | | | |

| **Coefficients** | | | | | | | | | | | | | |
| --- | --- | --- | --- | --- | --- | --- | --- | --- | --- | --- | --- | --- | --- |
|  | | **Estimate** | | **Standard Error** | | **z** | | **p** | | **Lower Bound** | | **Upper Bound** | |
| intrcpt |  | 0.818 |  | 0.084 |  | 9.700 |  | < .001 |  | 0.653 |  | 0.983 |  |
|  | | | | | | | | | | | | | |
| *Note.*  Wald test. | | | | | | | | | | | | | |

| **Residual Heterogeneity Estimates** | | | | | | | |
| --- | --- | --- | --- | --- | --- | --- | --- |
|  | | **Estimate** | | **Lower Bound** | | **Upper Bound** | |
| *τ²* |  | 0.000 |  | 0.000 |  | 0.000 |  |
| *τ* |  | 0.000 |  | 0.000 |  | 0.000 |  |
| *I²* (%) |  | 0.000 |  | 0.000 |  | 0.000 |  |
| *H²* |  | 1.000 |  | 1.000 |  | 1.000 |  |
|  | | | | | | | |

| **Regression test for Funnel plot asymmetry ("Egger's test")** | | | | | |
| --- | --- | --- | --- | --- | --- |
|  | | **z** | | **p** | |
| sei |  | 0.398 |  | 0.691 |  |
|  | | | | | |

**Plot**

**Forest plot**


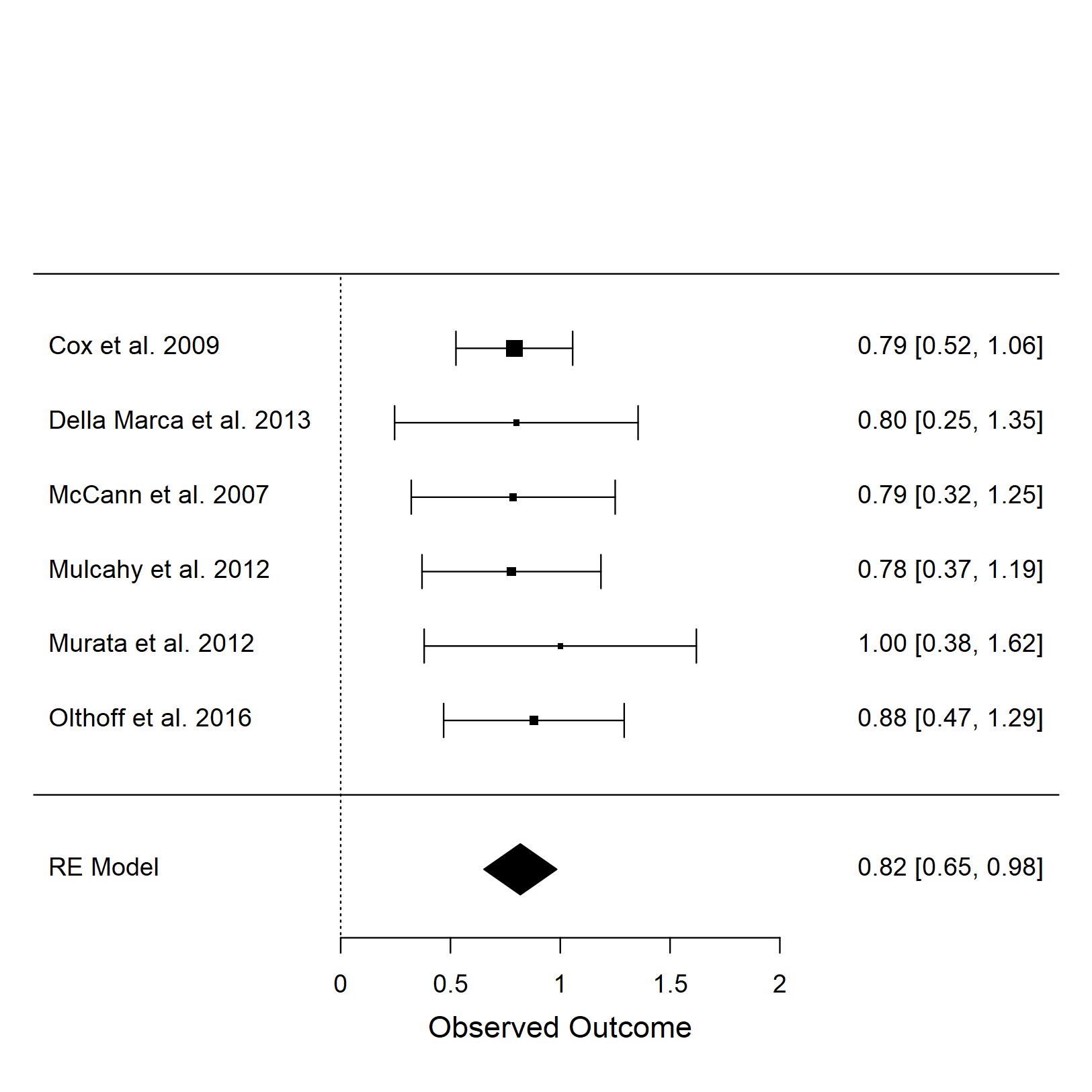


**Funnel plot**


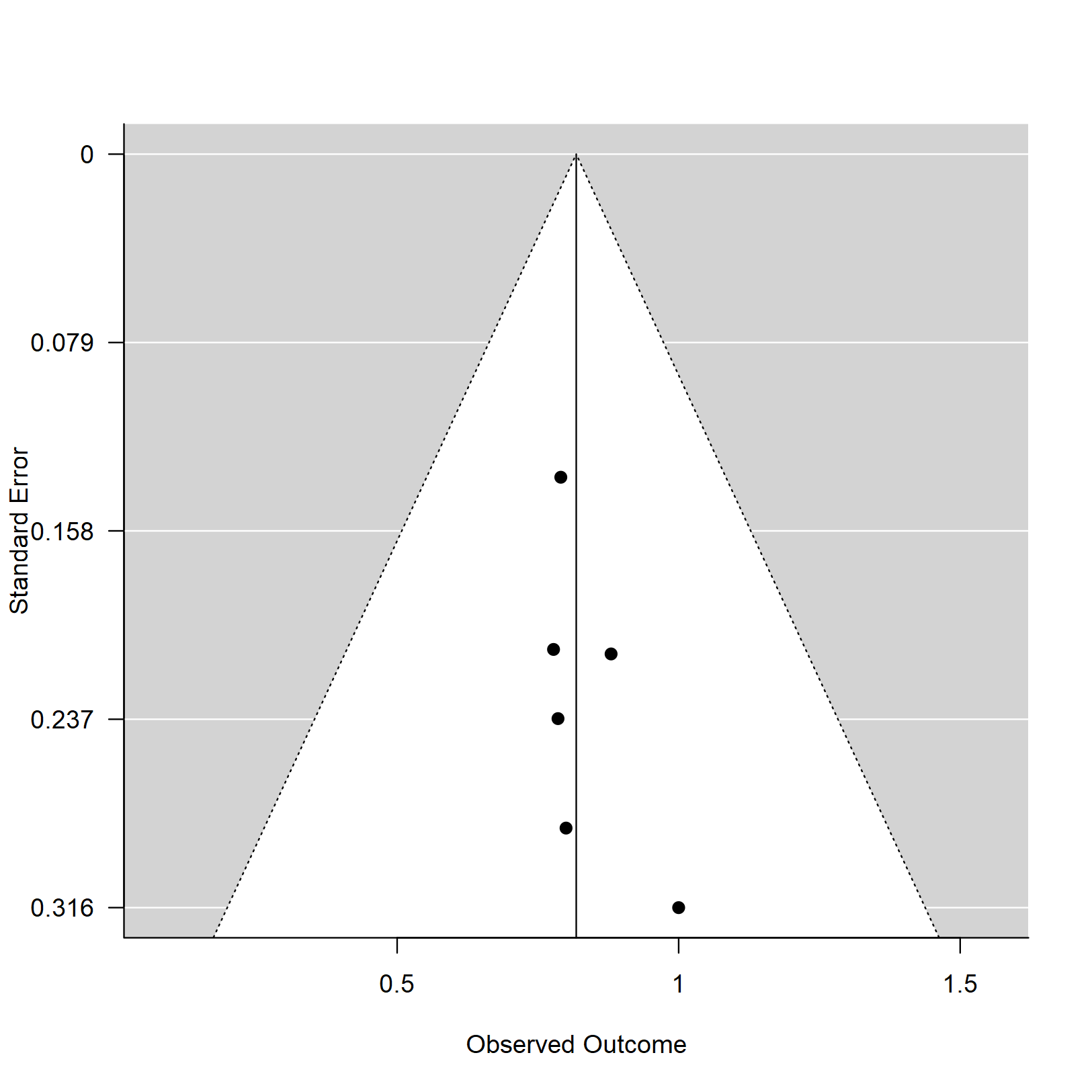


**Meta-Analysis Malignancy +**

| **Fixed and Random Effects** | | | | | | | |
| --- | --- | --- | --- | --- | --- | --- | --- |
|  | | **Q** | | **df** | | **p** | |
| Omnibus test of Model Coefficients |  | 138.869 |  | 1 |  | < .001 |  |
| Test of Residual Heterogeneity |  | 5.431 |  | 12 |  | 0.942 |  |
|  | | | | | | | |
| *Note.*   *p* -values are approximate. | | | | | | | |

| **Coefficients** | | | | | | | | | | | | | |
| --- | --- | --- | --- | --- | --- | --- | --- | --- | --- | --- | --- | --- | --- |
|  | | **Estimate** | | **Standard Error** | | **z** | | **p** | | **Lower Bound** | | **Upper Bound** | |
| intrcpt |  | 0.512 |  | 0.043 |  | 11.784 |  | < .001 |  | 0.427 |  | 0.598 |  |
|  | | | | | | | | | | | | | |
| *Note.*  Wald test. | | | | | | | | | | | | | |

| **Residual Heterogeneity Estimates** | | | | | | | |
| --- | --- | --- | --- | --- | --- | --- | --- |
|  | | **Estimate** | | **Lower Bound** | | **Upper Bound** | |
| *τ²* |  | 0.000 |  | 0.000 |  | 0.006 |  |
| *τ* |  | 0.000 |  | 0.000 |  | 0.076 |  |
| *I²* (%) |  | 0.000 |  | 0.000 |  | 18.113 |  |
| *H²* |  | 1.000 |  | 1.000 |  | 1.221 |  |
|  | | | | | | | |

| **Regression test for Funnel plot asymmetry ("Egger's test")** | | | | | |
| --- | --- | --- | --- | --- | --- |
|  | | **z** | | **p** | |
| sei |  | 0.860 |  | 0.390 |  |
|  | | | | | |

**Plot**

**Forest plot**


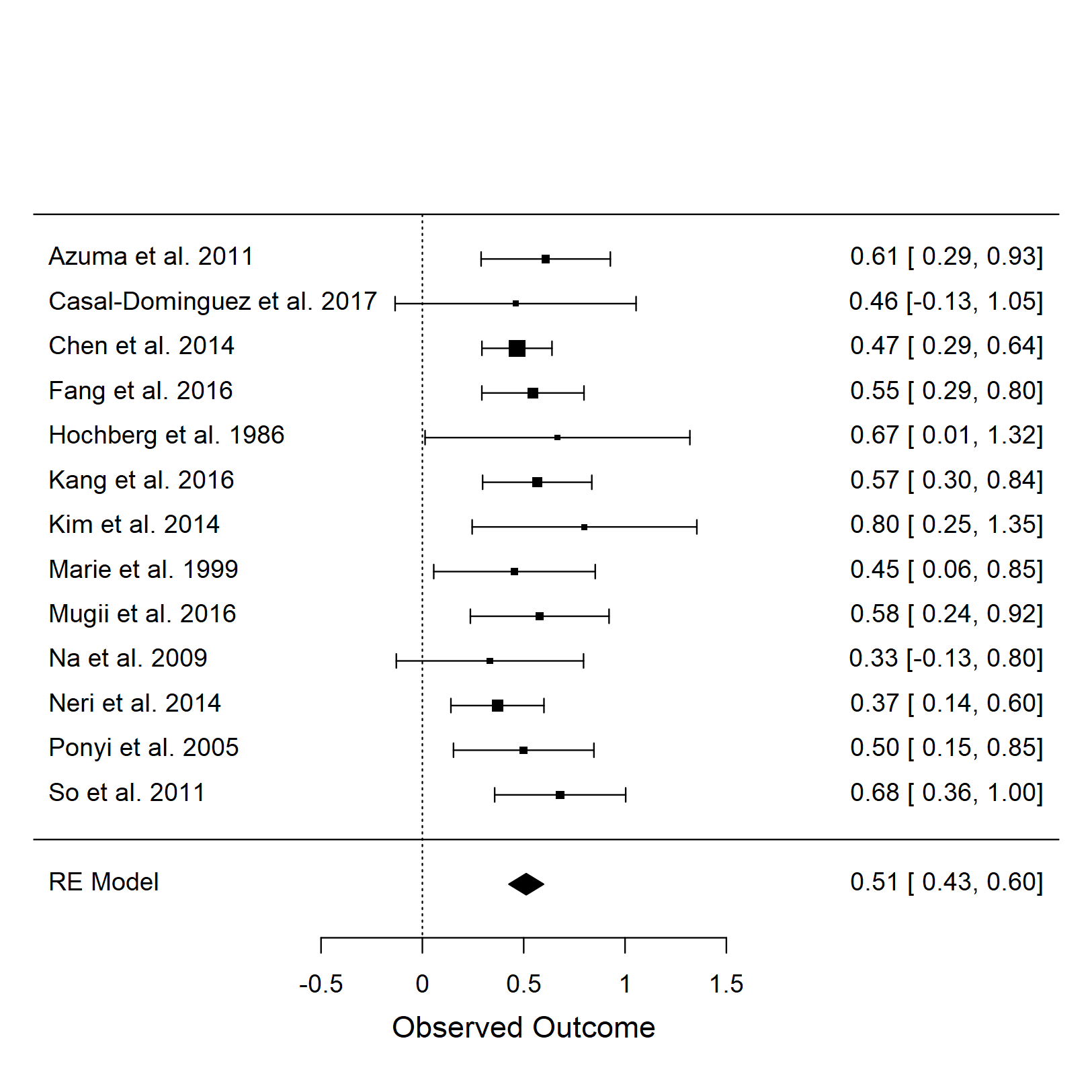


**Funnel plot**


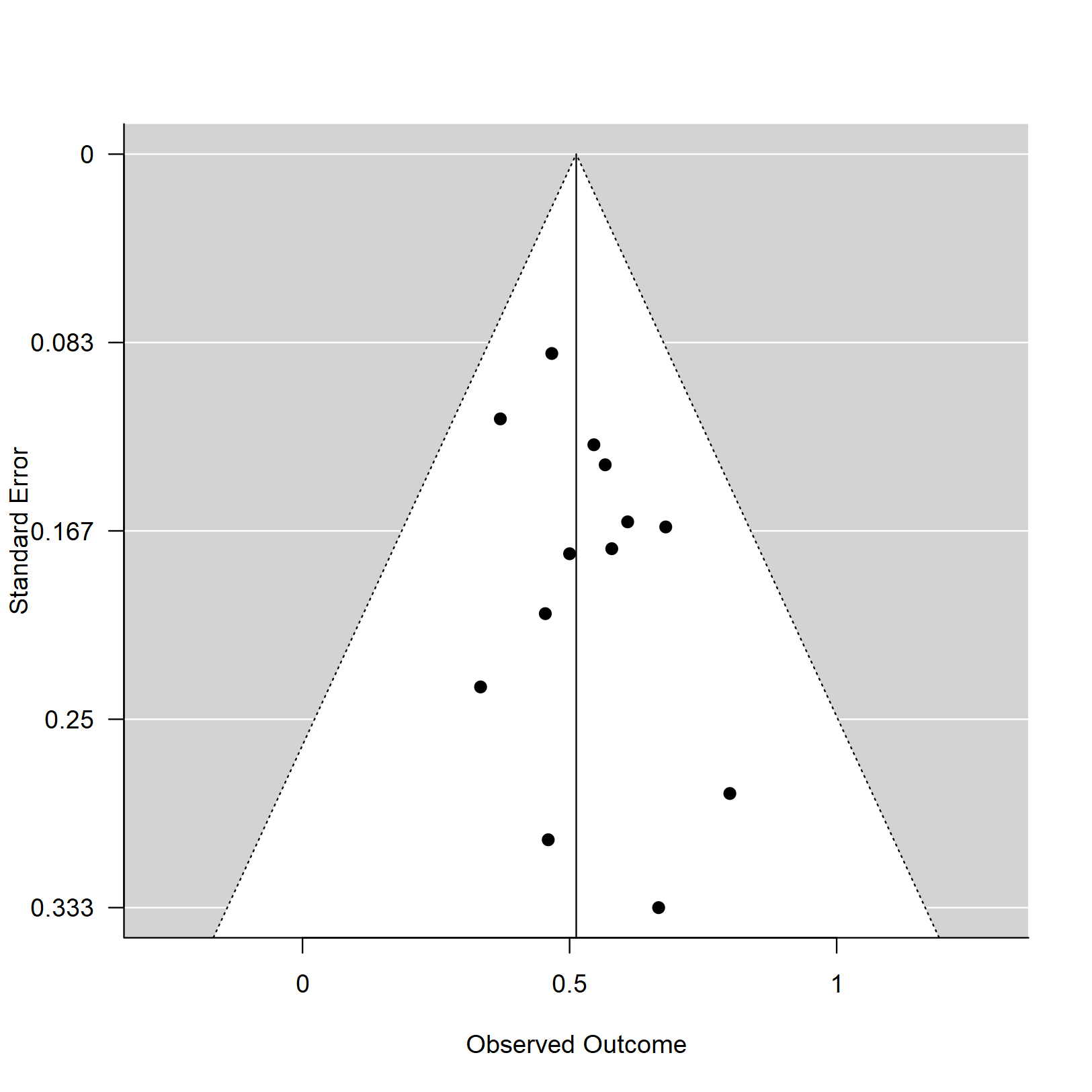


**Meta-Analysis Malignancy -**

| **Fixed and Random Effects** | | | | | | | |
| --- | --- | --- | --- | --- | --- | --- | --- |
|  | | **Q** | | **df** | | **p** | |
| Omnibus test of Model Coefficients |  | 45.044 |  | 1 |  | < .001 |  |
| Test of Residual Heterogeneity |  | 103.448 |  | 12 |  | < .001 |  |
|  | | | | | | | |
| *Note.*   *p* -values are approximate. | | | | | | | |

| **Coefficients** | | | | | | | | | | | | | |
| --- | --- | --- | --- | --- | --- | --- | --- | --- | --- | --- | --- | --- | --- |
|  | | **Estimate** | | **Standard Error** | | **z** | | **p** | | **Lower Bound** | | **Upper Bound** | |
| intrcpt |  | 0.233 |  | 0.035 |  | 6.711 |  | < .001 |  | 0.165 |  | 0.301 |  |
|  | | | | | | | | | | | | | |
| *Note.*  Wald test. | | | | | | | | | | | | | |

| **Residual Heterogeneity Estimates** | | | | | | | |
| --- | --- | --- | --- | --- | --- | --- | --- |
|  | | **Estimate** | | **Lower Bound** | | **Upper Bound** | |
| *τ²* |  | 0.012 |  | 0.005 |  | 0.038 |  |
| *τ* |  | 0.109 |  | 0.067 |  | 0.195 |  |
| *I²* (%) |  | 85.251 |  | 68.558 |  | 94.866 |  |
| *H²* |  | 6.780 |  | 3.181 |  | 19.476 |  |
|  | | | | | | | |

| **Regression test for Funnel plot asymmetry ("Egger's test")** | | | | | |
| --- | --- | --- | --- | --- | --- |
|  | | **z** | | **p** | |
| sei |  | 2.310 |  | 0.021 |  |
|  | | | | | |

**Plot**

**Forest plot**


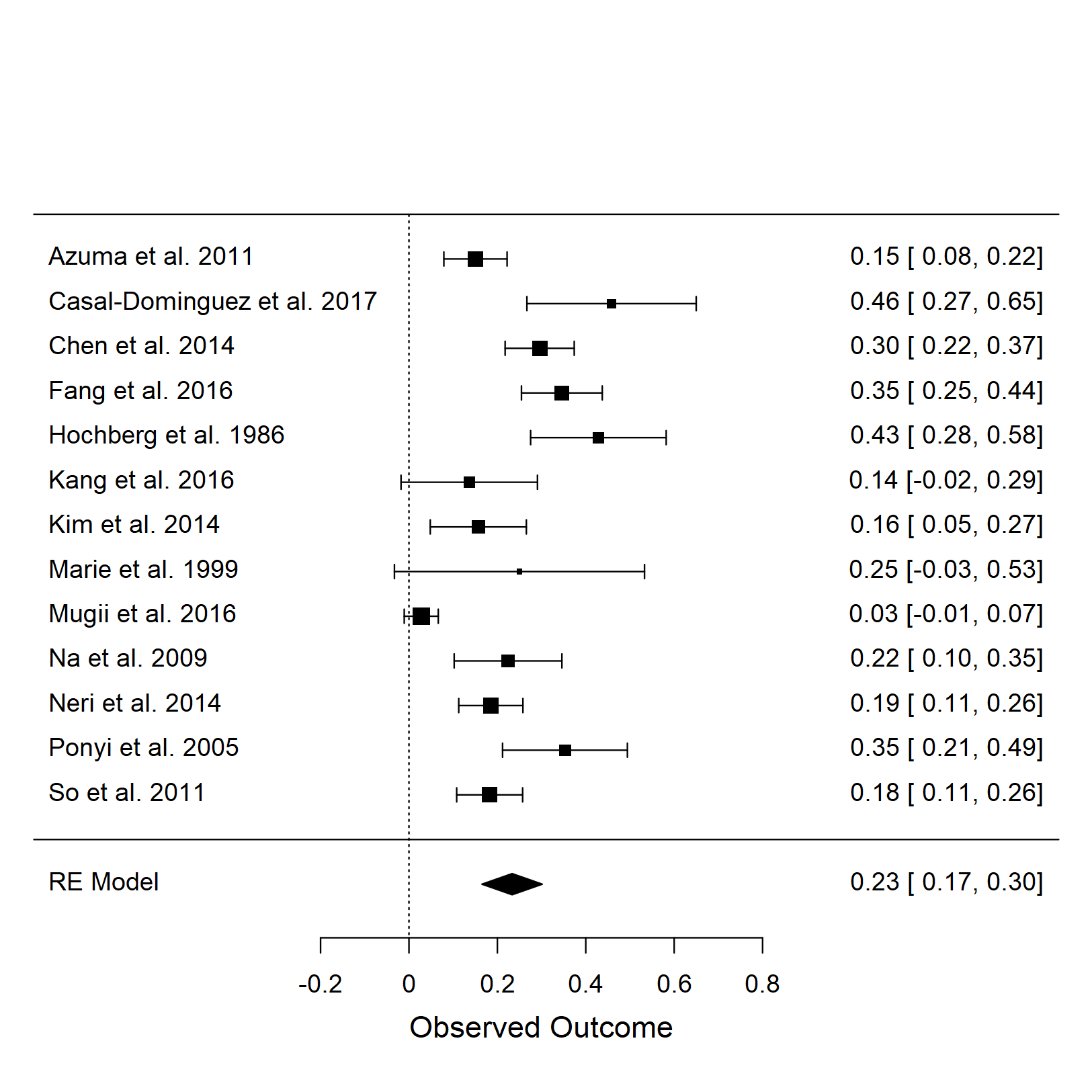


**Funnel plot**


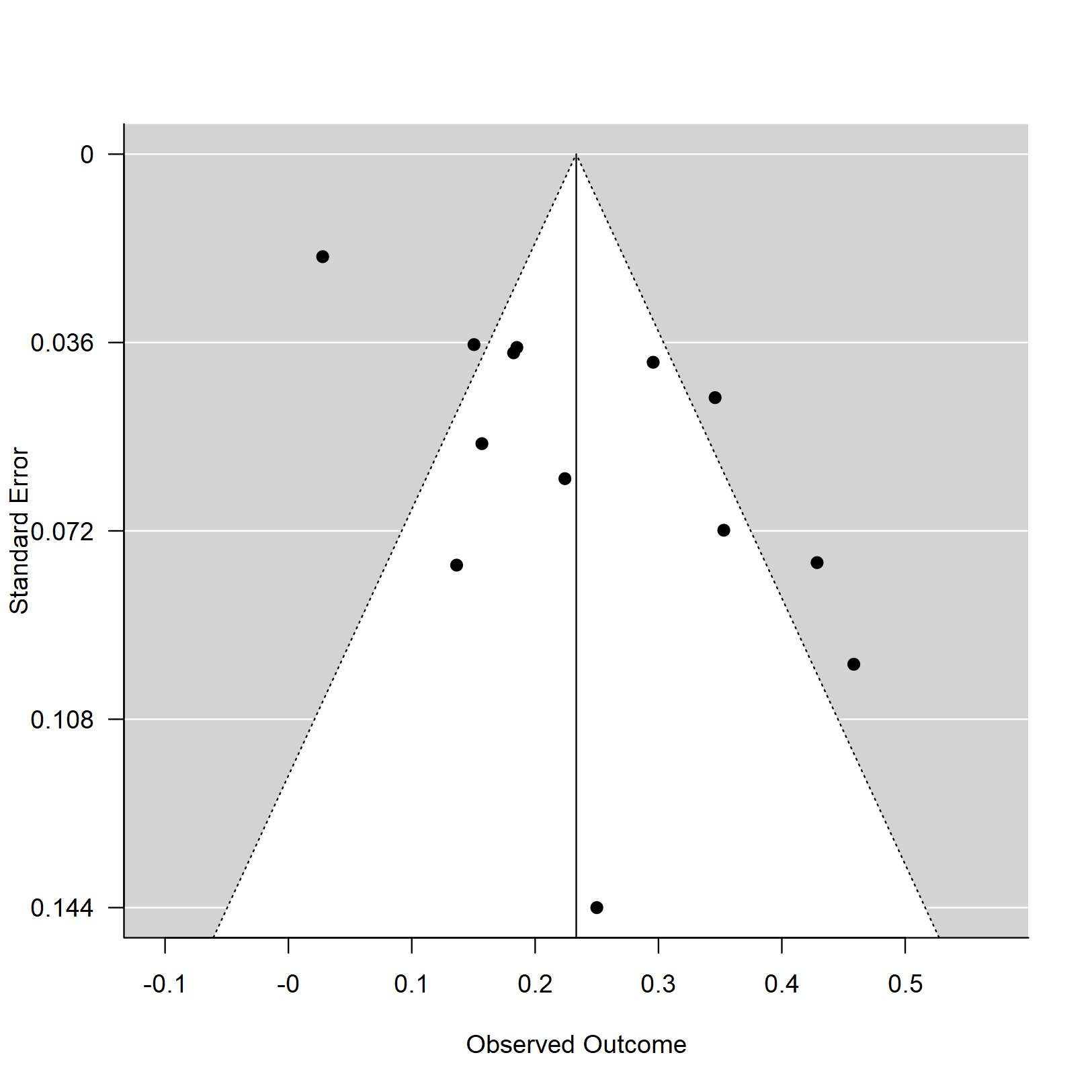


**Meta-Analysis NXP2 +**

| **Fixed and Random Effects** | | | | | | | |
| --- | --- | --- | --- | --- | --- | --- | --- |
|  | | **Q** | | **df** | | **p** | |
| Omnibus test of Model Coefficients |  | 109.402 |  | 1 |  | < .001 |  |
| Test of Residual Heterogeneity |  | 1.598 |  | 4 |  | 0.809 |  |
|  | | | | | | | |
| *Note.*   *p* -values are approximate. | | | | | | | |

| **Coefficients** | | | | | | | | | | | | | |
| --- | --- | --- | --- | --- | --- | --- | --- | --- | --- | --- | --- | --- | --- |
|  | | **Estimate** | | **Standard Error** | | **z** | | **p** | | **Lower Bound** | | **Upper Bound** | |
| intrcpt |  | 0.558 |  | 0.053 |  | 10.460 |  | < .001 |  | 0.454 |  | 0.663 |  |
|  | | | | | | | | | | | | | |
| *Note.*  Wald test. | | | | | | | | | | | | | |

| **Residual Heterogeneity Estimates** | | | | | | | |
| --- | --- | --- | --- | --- | --- | --- | --- |
|  | | **Estimate** | | **Lower Bound** | | **Upper Bound** | |
| *τ²* |  | 0.000 |  | 0.000 |  | 0.030 |  |
| *τ* |  | 0.000 |  | 0.000 |  | 0.172 |  |
| *I²* (%) |  | 0.000 |  | 0.000 |  | 63.620 |  |
| *H²* |  | 1.000 |  | 1.000 |  | 2.749 |  |
|  | | | | | | | |

| **Regression test for Funnel plot asymmetry ("Egger's test")** | | | | | |
| --- | --- | --- | --- | --- | --- |
|  | | **z** | | **p** | |
| sei |  | 0.799 |  | 0.424 |  |
|  | | | | | |

**Plot**

**Forest plot**


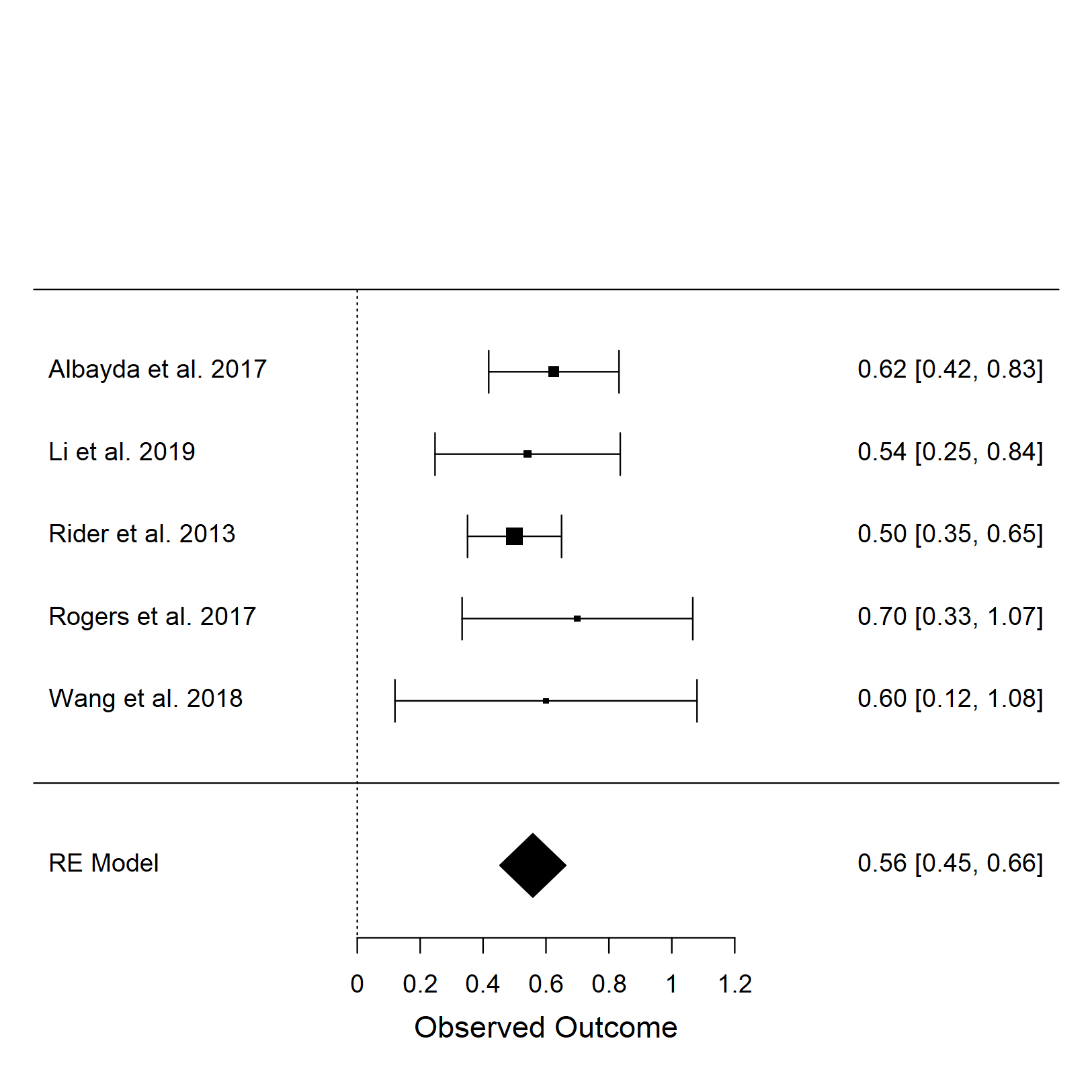


**Funnel plot**


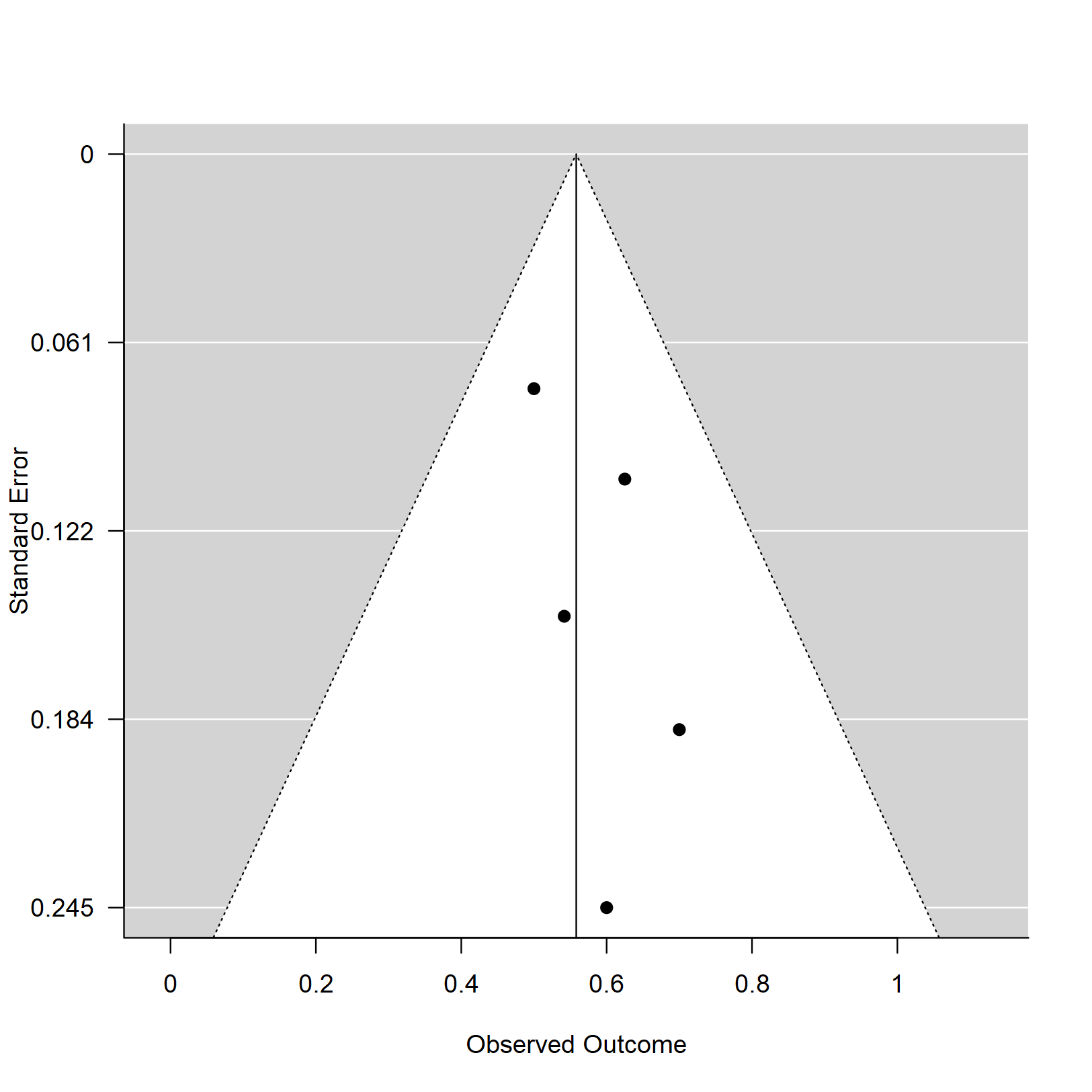


**Meta-Analysis NXP2-**

| **Fixed and Random Effects** | | | | | | | |
| --- | --- | --- | --- | --- | --- | --- | --- |
|  | | **Q** | | **df** | | **p** | |
| Omnibus test of Model Coefficients |  | 202.693 |  | 1 |  | < .001 |  |
| Test of Residual Heterogeneity |  | 6.825 |  | 4 |  | 0.145 |  |
|  | | | | | | | |
| *Note.*   *p* -values are approximate. | | | | | | | |

| **Coefficients** | | | | | | | | | | | | | |
| --- | --- | --- | --- | --- | --- | --- | --- | --- | --- | --- | --- | --- | --- |
|  | | **Estimate** | | **Standard Error** | | **z** | | **p** | | **Lower Bound** | | **Upper Bound** | |
| intrcpt |  | 0.328 |  | 0.023 |  | 14.237 |  | < .001 |  | 0.282 |  | 0.373 |  |
|  | | | | | | | | | | | | | |
| *Note.*  Wald test. | | | | | | | | | | | | | |

| **Residual Heterogeneity Estimates** | | | | | | | |
| --- | --- | --- | --- | --- | --- | --- | --- |
|  | | **Estimate** | | **Lower Bound** | | **Upper Bound** | |
| *τ²* |  | 0.001 |  | 0.000 |  | 0.016 |  |
| *τ* |  | 0.033 |  | 0.000 |  | 0.128 |  |
| *I²* (%) |  | 42.337 |  | 0.000 |  | 91.670 |  |
| *H²* |  | 1.734 |  | 1.000 |  | 12.005 |  |
|  | | | | | | | |

| **Regression test for Funnel plot asymmetry ("Egger's test")** | | | | | |
| --- | --- | --- | --- | --- | --- |
|  | | **z** | | **p** | |
| sei |  | 1.223 |  | 0.221 |  |
|  | | | | | |

**Plot**

**Forest plot**


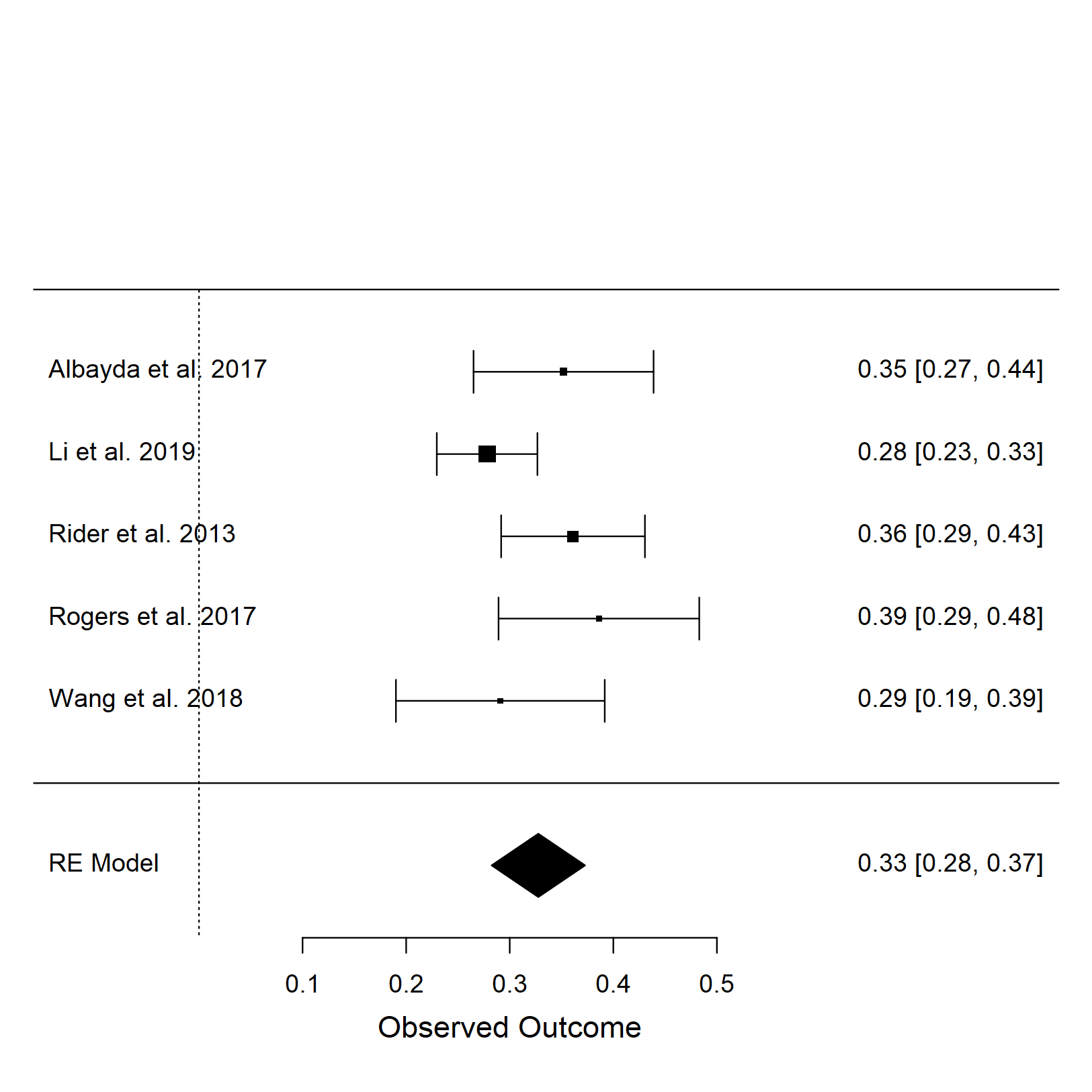


**Funnel plot**


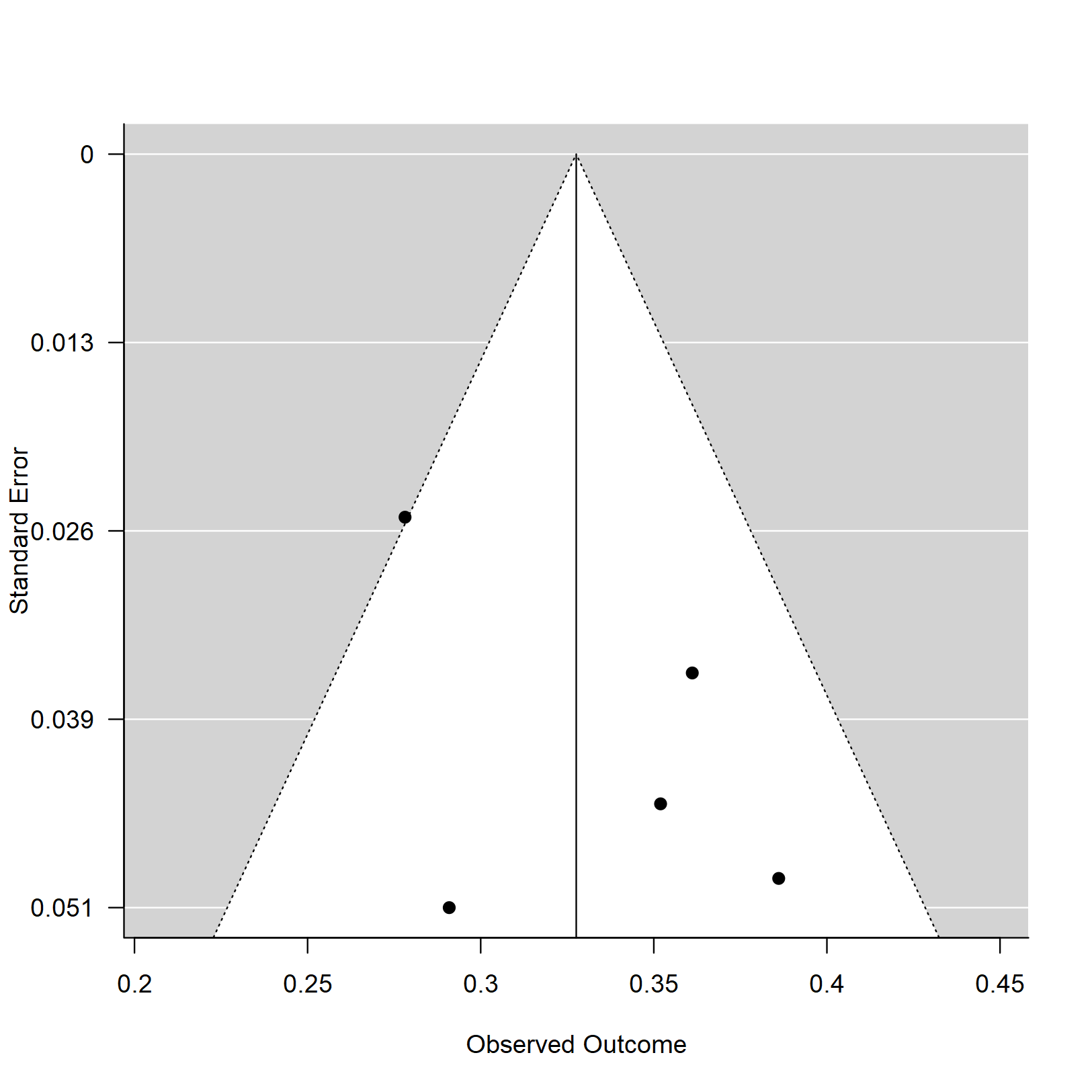


**Meta-Analysis MDA5 +**

| **Fixed and Random Effects** | | | | | | | |
| --- | --- | --- | --- | --- | --- | --- | --- |
|  | | **Q** | | **df** | | **p** | |
| Omnibus test of Model Coefficients |  | 3.762 |  | 1 |  | 0.052 |  |
| Test of Residual Heterogeneity |  | 5.088 |  | 2 |  | 0.079 |  |
|  | | | | | | | |
| *Note.*   *p* -values are approximate. | | | | | | | |

| **Coefficients** | | | | | | | | | | | | | |
| --- | --- | --- | --- | --- | --- | --- | --- | --- | --- | --- | --- | --- | --- |
|  | | **Estimate** | | **Standard Error** | | **z** | | **p** | | **Lower Bound** | | **Upper Bound** | |
| intrcpt |  | 0.116 |  | 0.060 |  | 1.940 |  | 0.052 |  | -0.001 |  | 0.233 |  |
|  | | | | | | | | | | | | | |
| *Note.*  Wald test. | | | | | | | | | | | | | |

| **Residual Heterogeneity Estimates** | | | | | | | |
| --- | --- | --- | --- | --- | --- | --- | --- |
|  | | **Estimate** | | **Lower Bound** | | **Upper Bound** | |
| *τ²* |  | 0.006 |  | 0.000 |  | 0.779 |  |
| *τ* |  | 0.078 |  | 0.000 |  | 0.883 |  |
| *I²* (%) |  | 61.374 |  | 0.000 |  | 99.510 |  |
| *H²* |  | 2.589 |  | 1.000 |  | 204.044 |  |
|  | | | | | | | |

| **Regression test for Funnel plot asymmetry ("Egger's test")** | | | | | |
| --- | --- | --- | --- | --- | --- |
|  | | **z** | | **p** | |
| sei |  | 1.521 |  | 0.128 |  |
|  | | | | | |

**Plot**

**Forest plot**


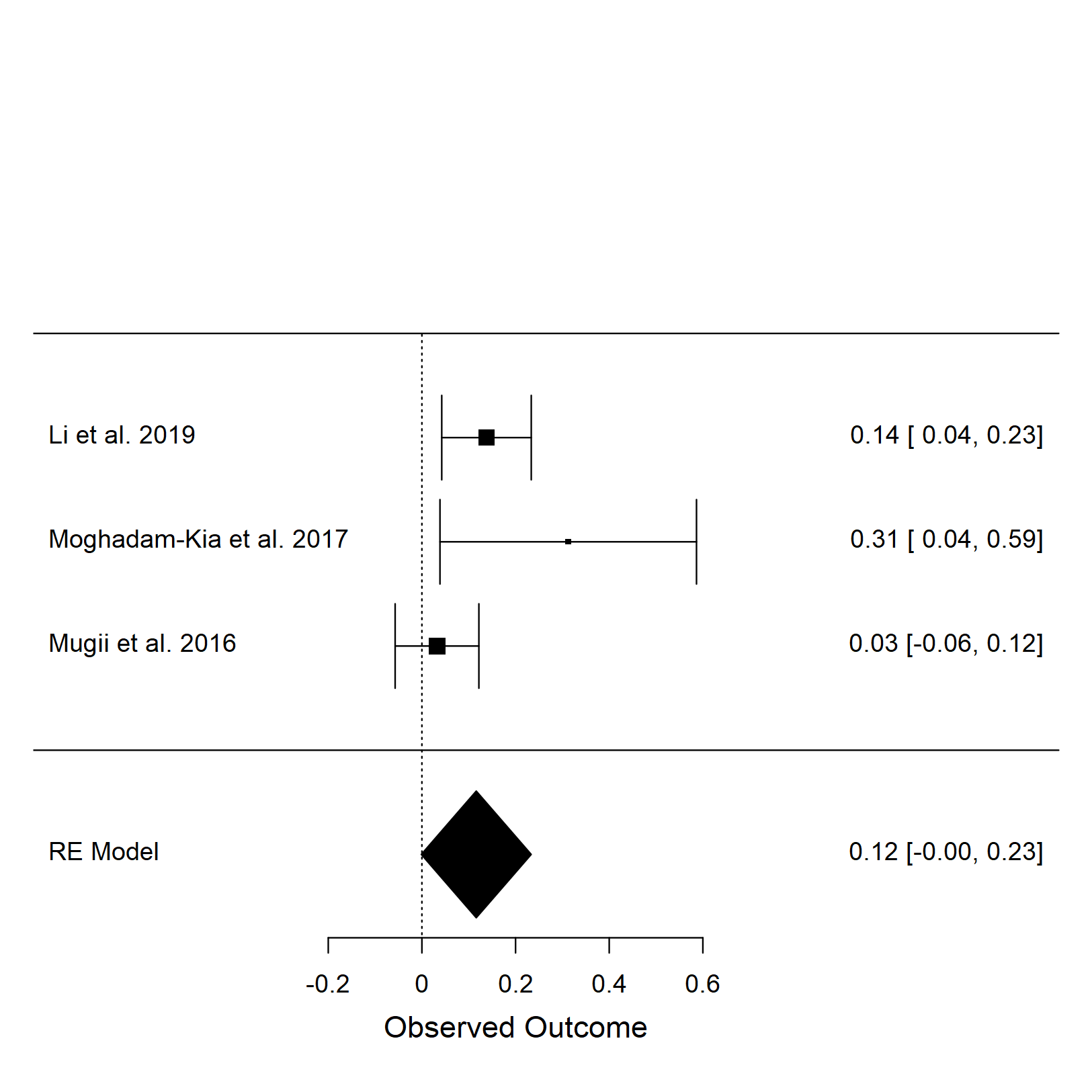


**Funnel plot**


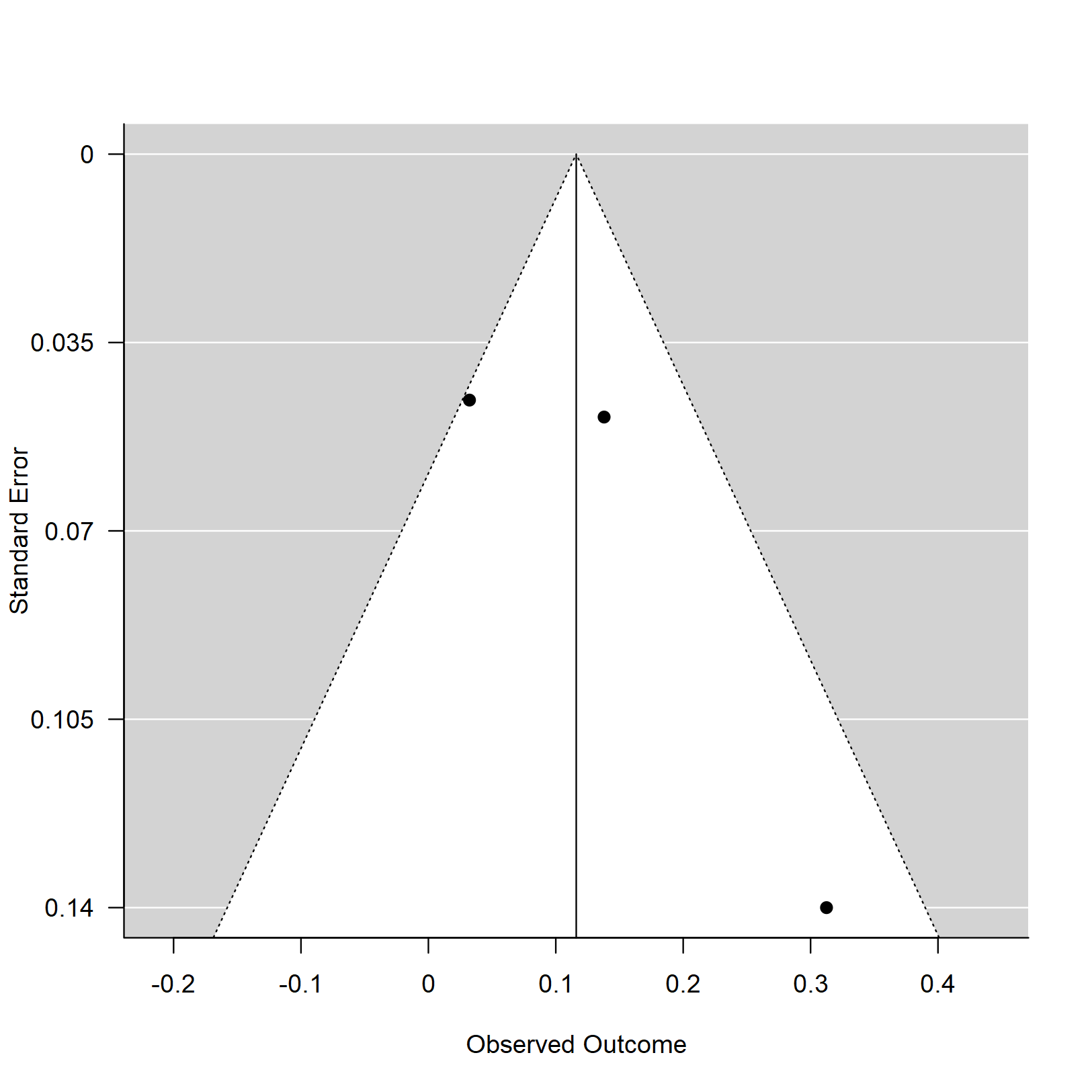


**Meta-Analyisi MAD -**

| **Fixed and Random Effects** | | | | | | | |
| --- | --- | --- | --- | --- | --- | --- | --- |
|  | | **Q** | | **df** | | **p** | |
| Omnibus test of Model Coefficients |  | 14.577 |  | 1 |  | < .001 |  |
| Test of Residual Heterogeneity |  | 16.619 |  | 2 |  | < .001 |  |
|  | | | | | | | |
| *Note.*   *p* -values are approximate. | | | | | | | |

| **Coefficients** | | | | | | | | | | | | | |
| --- | --- | --- | --- | --- | --- | --- | --- | --- | --- | --- | --- | --- | --- |
|  | | **Estimate** | | **Standard Error** | | **z** | | **p** | | **Lower Bound** | | **Upper Bound** | |
| intrcpt |  | 0.211 |  | 0.055 |  | 3.818 |  | < .001 |  | 0.103 |  | 0.320 |  |
|  | | | | | | | | | | | | | |
| *Note.*  Wald test. | | | | | | | | | | | | | |

| **Residual Heterogeneity Estimates** | | | | | | | |
| --- | --- | --- | --- | --- | --- | --- | --- |
|  | | **Estimate** | | **Lower Bound** | | **Upper Bound** | |
| *τ²* |  | 0.008 |  | 0.001 |  | 0.333 |  |
| *τ* |  | 0.088 |  | 0.035 |  | 0.577 |  |
| *I²* (%) |  | 85.697 |  | 49.008 |  | 99.611 |  |
| *H²* |  | 6.992 |  | 1.961 |  | 256.898 |  |
|  | | | | | | | |

| **Regression test for Funnel plot asymmetry ("Egger's test")** | | | | | |
| --- | --- | --- | --- | --- | --- |
|  | | **z** | | **p** | |
| sei |  | -1.225 |  | 0.220 |  |
|  | | | | | |

**Plot**

**Forest plot**


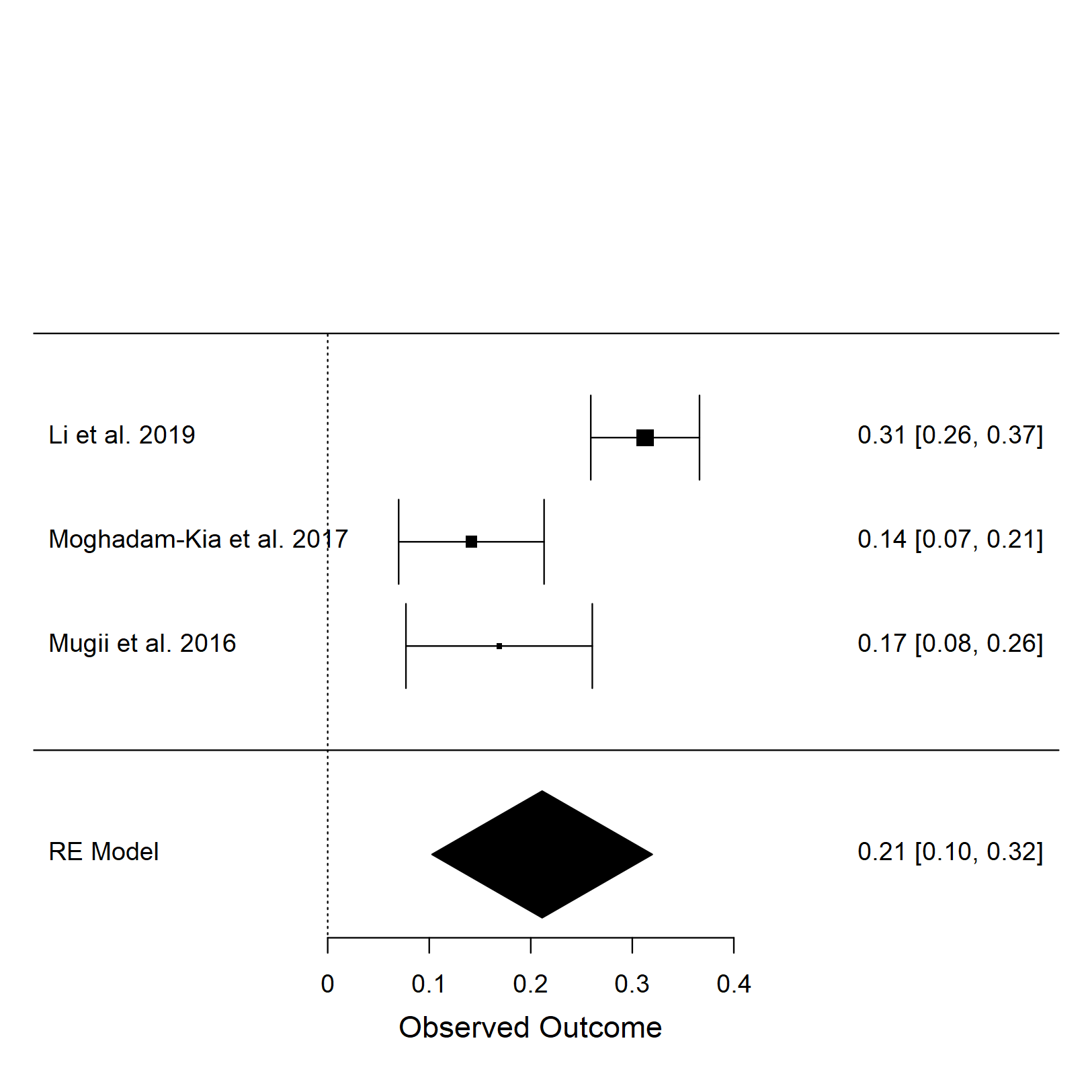


**Funnel plot**


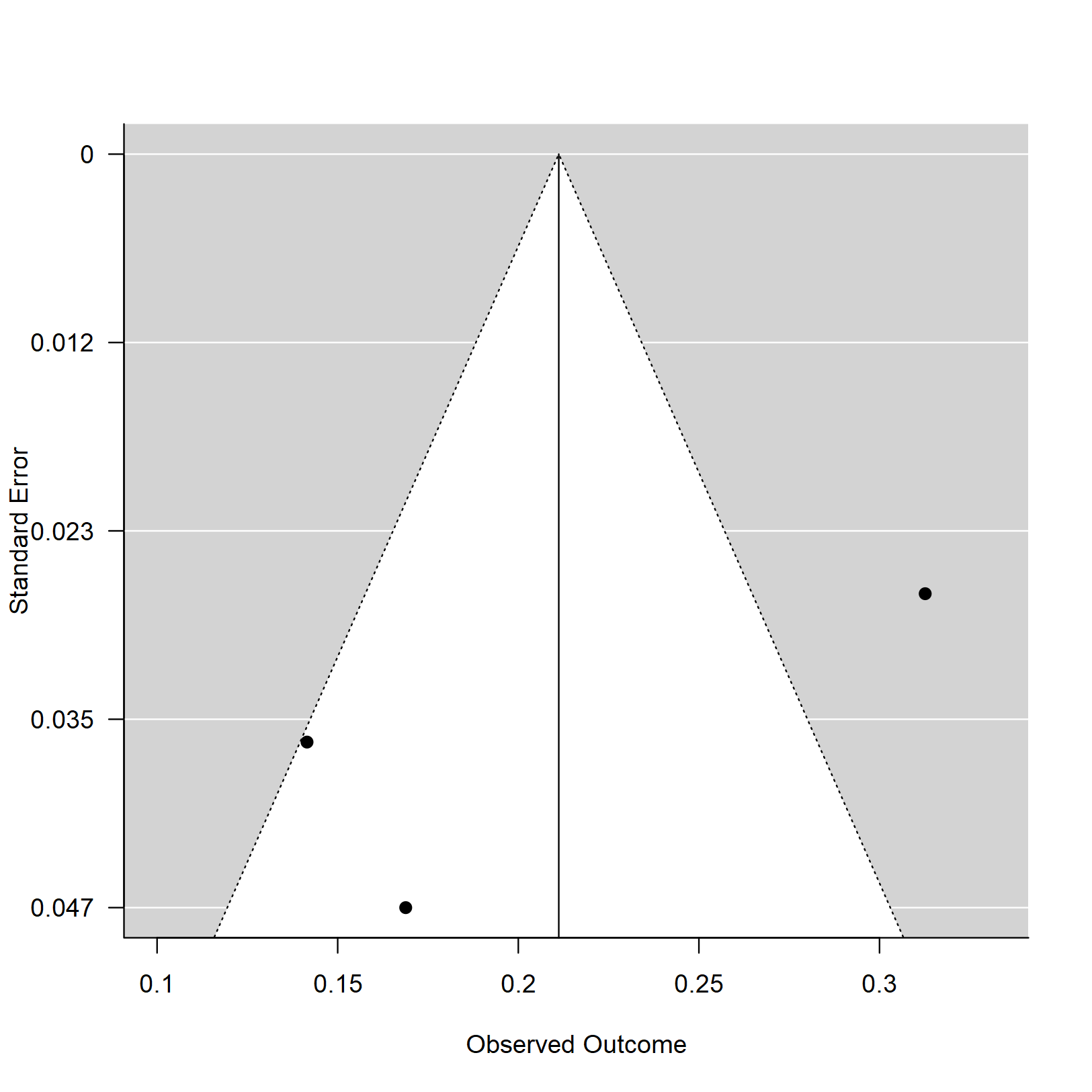


**Meta Analysis SEA +**

| **Fixed and Random Effects** | | | | | | | |
| --- | --- | --- | --- | --- | --- | --- | --- |
|  | | **Q** | | **df** | | **p** | |
| Omnibus test of Model Coefficients |  | 12.996 |  | 1 |  | < .001 |  |
| Test of Residual Heterogeneity |  | 0.004 |  | 1 |  | 0.948 |  |
|  | | | | | | | |
| *Note.*   *p* -values are approximate. | | | | | | | |

| **Coefficients** | | | | | | | | | | | | | |
| --- | --- | --- | --- | --- | --- | --- | --- | --- | --- | --- | --- | --- | --- |
|  | | **Estimate** | | **Standard Error** | | **z** | | **p** | | **Lower Bound** | | **Upper Bound** | |
| intrcpt |  | 0.764 |  | 0.212 |  | 3.605 |  | < .001 |  | 0.349 |  | 1.180 |  |
|  | | | | | | | | | | | | | |
| *Note.*  Wald test. | | | | | | | | | | | | | |

| **Residual Heterogeneity Estimates** | | | | | | | |
| --- | --- | --- | --- | --- | --- | --- | --- |
|  | | **Estimate** | | **Lower Bound** | | **Upper Bound** | |
| *τ²* |  | 0.000 |  | 0.000 |  | 0.303 |  |
| *τ* |  | 0.000 |  | 0.000 |  | 0.550 |  |
| *I²* (%) |  | 0.000 |  | 0.000 |  | 77.069 |  |
| *H²* |  | 1.000 |  | 1.000 |  | 4.361 |  |
|  | | | | | | | |

**Plot**

**Forest plot**


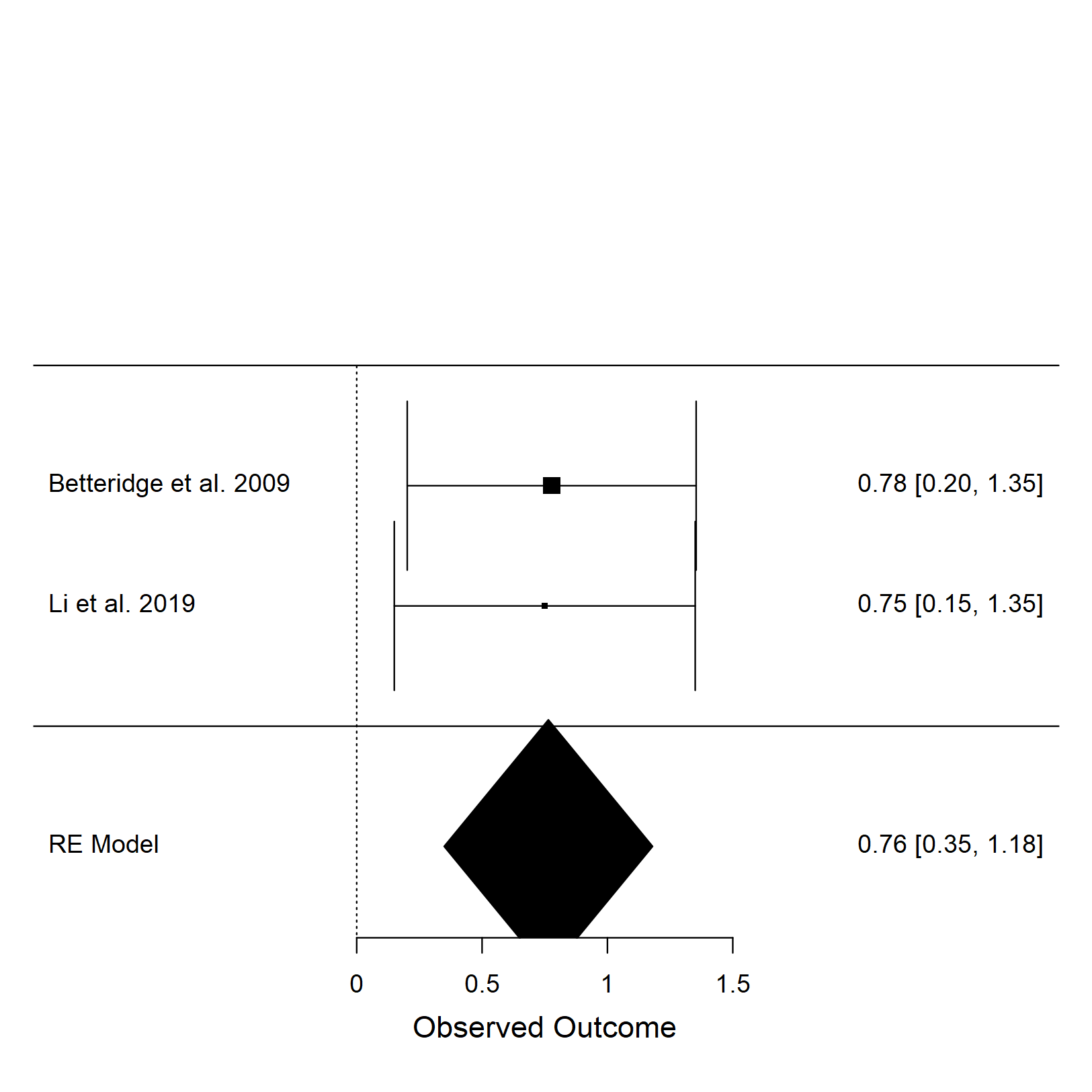


**Funnel plot**


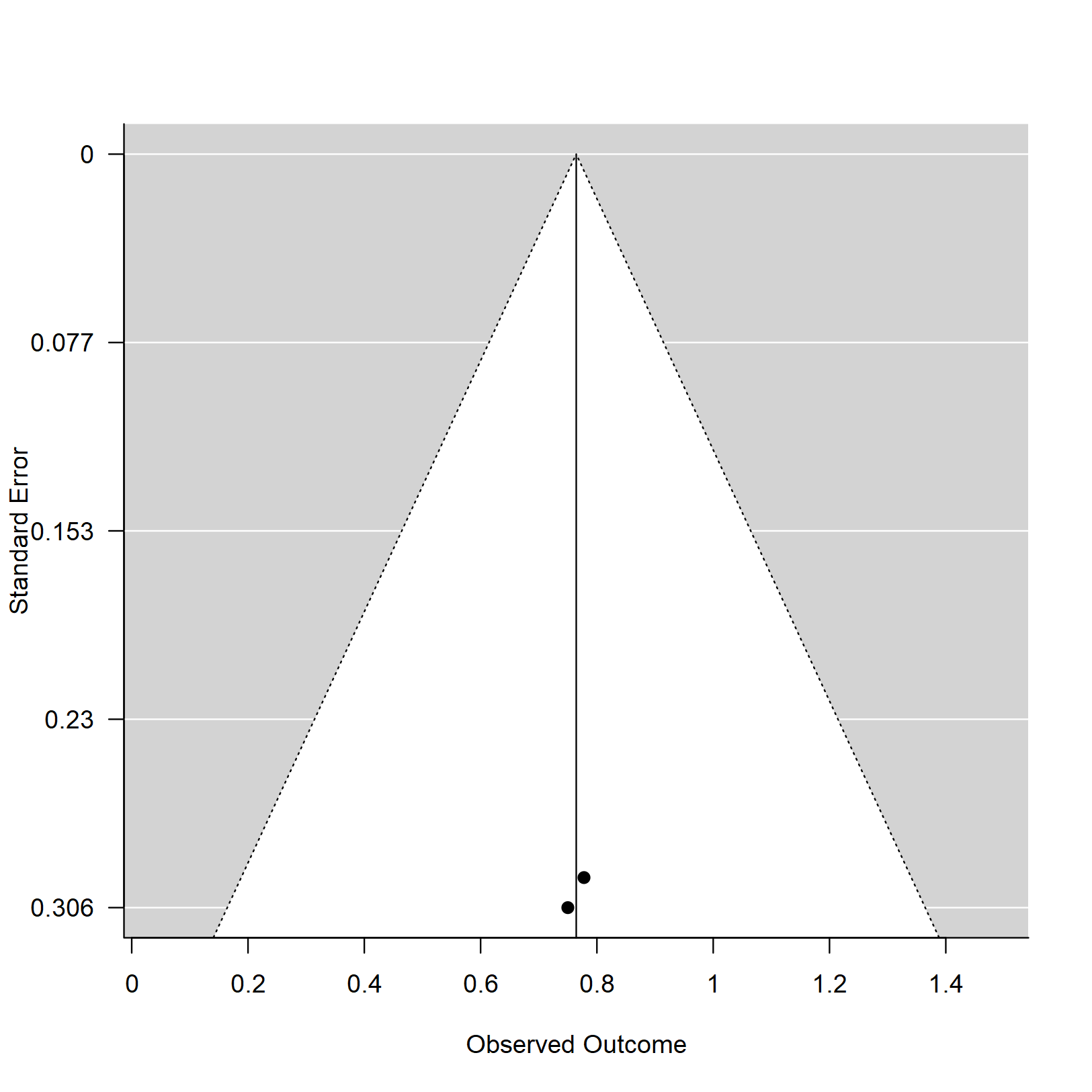


**Meta Analysis SAE -**

| **Fixed and Random Effects** | | | | | | | |
| --- | --- | --- | --- | --- | --- | --- | --- |
|  | | **Q** | | **df** | | **p** | |
| Omnibus test of Model Coefficients |  | 22.050 |  | 1 |  | < .001 |  |
| Test of Residual Heterogeneity |  | 5.319 |  | 1 |  | 0.021 |  |
|  | | | | | | | |
| *Note.*   *p* -values are approximate. | | | | | | | |

| **Coefficients** | | | | | | | | | | | | | |
| --- | --- | --- | --- | --- | --- | --- | --- | --- | --- | --- | --- | --- | --- |
|  | | **Estimate** | | **Standard Error** | | **z** | | **p** | | **Lower Bound** | | **Upper Bound** | |
| intrcpt |  | 0.348 |  | 0.074 |  | 4.696 |  | < .001 |  | 0.203 |  | 0.494 |  |
|  | | | | | | | | | | | | | |
| *Note.*  Wald test. | | | | | | | | | | | | | |

| **Residual Heterogeneity Estimates** | | | | | | | |
| --- | --- | --- | --- | --- | --- | --- | --- |
|  | | **Estimate** | | **Lower Bound** | | **Upper Bound** | |
| *τ²* |  | 0.009 |  | 1.159e -4 |  | 11.415 |  |
| *τ* |  | 0.095 |  | 0.011 |  | 3.379 |  |
| *I²* (%) |  | 81.201 |  | 5.212 |  | 99.982 |  |
| *H²* |  | 5.319 |  | 1.055 |  | 5416.554 |  |
|  | | | | | | | |

**Plot**

**Forest plot**


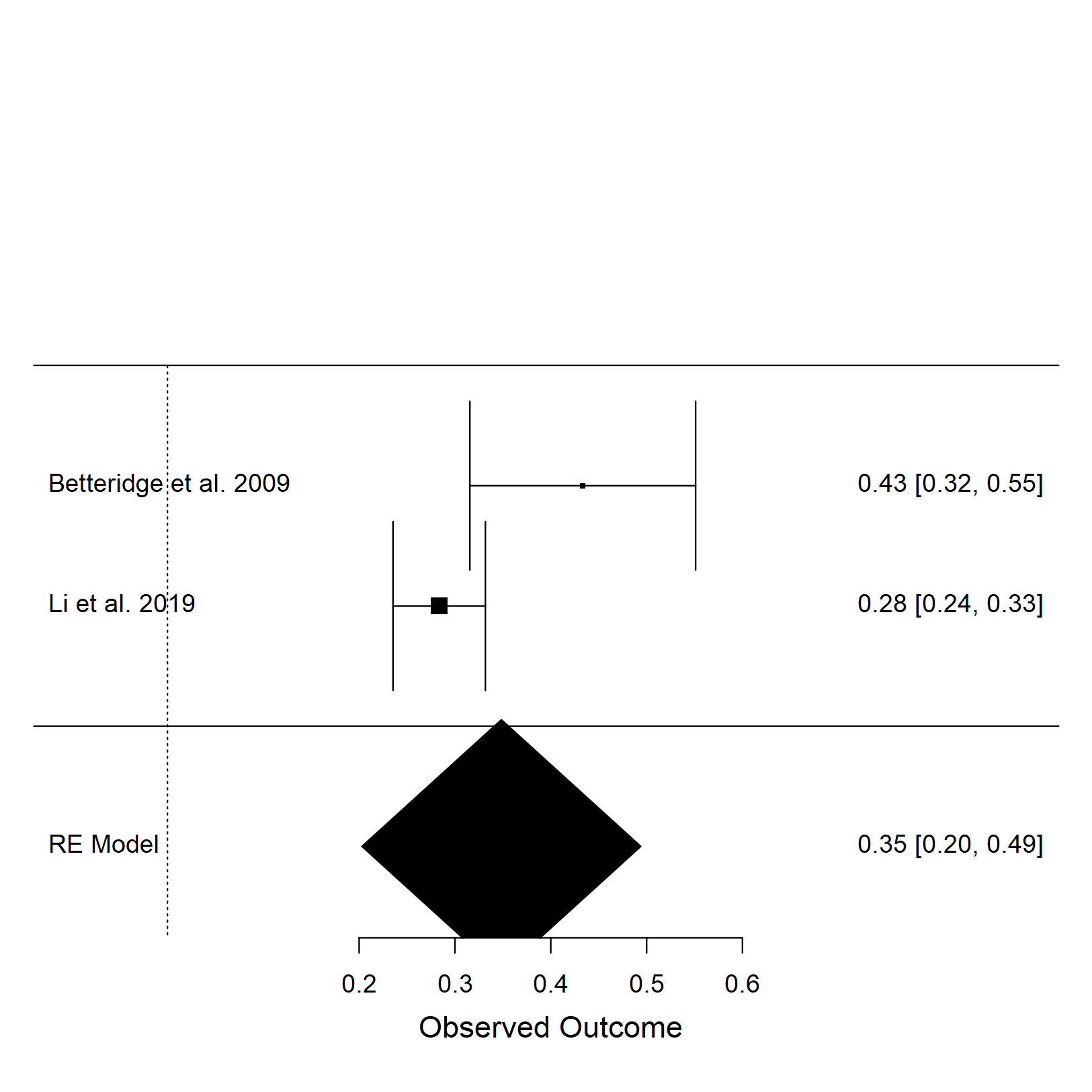


**Funnel plot**


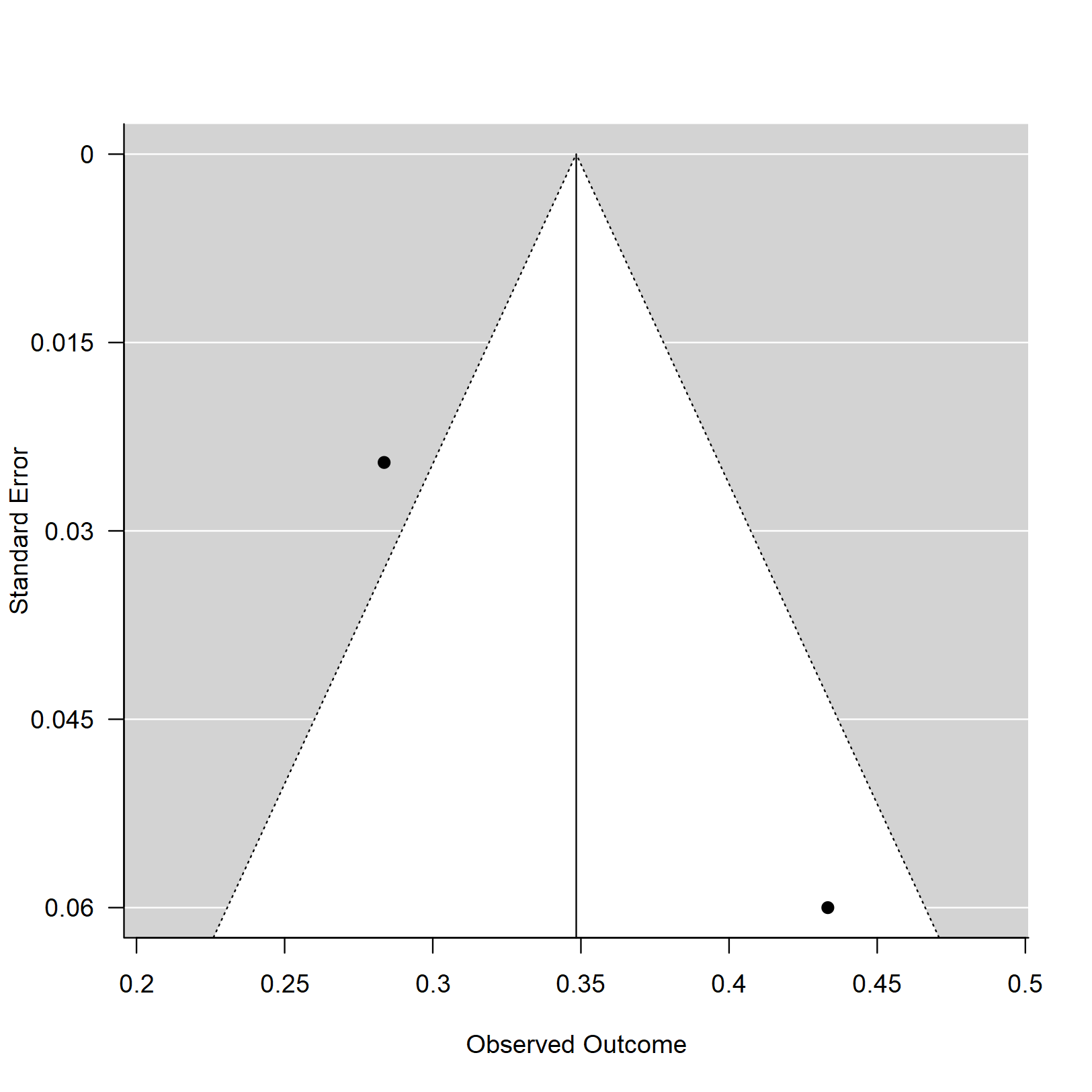


**Meta Analysis SRP +**

| **Fixed and Random Effects** | | | | | | | |
| --- | --- | --- | --- | --- | --- | --- | --- |
|  | | **Q** | | **df** | | **p** | |
| Omnibus test of Model Coefficients |  | 31.606 |  | 1 |  | < .001 |  |
| Test of Residual Heterogeneity |  | 0.394 |  | 2 |  | 0.821 |  |
|  | | | | | | | |
| *Note.*   *p* -values are approximate. | | | | | | | |

| **Coefficients** | | | | | | | | | | | | | |
| --- | --- | --- | --- | --- | --- | --- | --- | --- | --- | --- | --- | --- | --- |
|  | | **Estimate** | | **Standard Error** | | **z** | | **p** | | **Lower Bound** | | **Upper Bound** | |
| intrcpt |  | 0.620 |  | 0.110 |  | 5.622 |  | < .001 |  | 0.404 |  | 0.836 |  |
|  | | | | | | | | | | | | | |
| *Note.*  Wald test. | | | | | | | | | | | | | |

| **Residual Heterogeneity Estimates** | | | | | | | |
| --- | --- | --- | --- | --- | --- | --- | --- |
|  | | **Estimate** | | **Lower Bound** | | **Upper Bound** | |
| *τ²* |  | 0.000 |  | 0.000 |  | 0.324 |  |
| *τ* |  | 0.000 |  | 0.000 |  | 0.569 |  |
| *I²* (%) |  | 0.000 |  | 0.000 |  | 89.087 |  |
| *H²* |  | 1.000 |  | 1.000 |  | 9.164 |  |
|  | | | | | | | |

| **Regression test for Funnel plot asymmetry ("Egger's test")** | | | | | |
| --- | --- | --- | --- | --- | --- |
|  | | **z** | | **p** | |
| sei |  | -0.399 |  | 0.690 |  |
|  | | | | | |

**Plot**

**Forest plot**


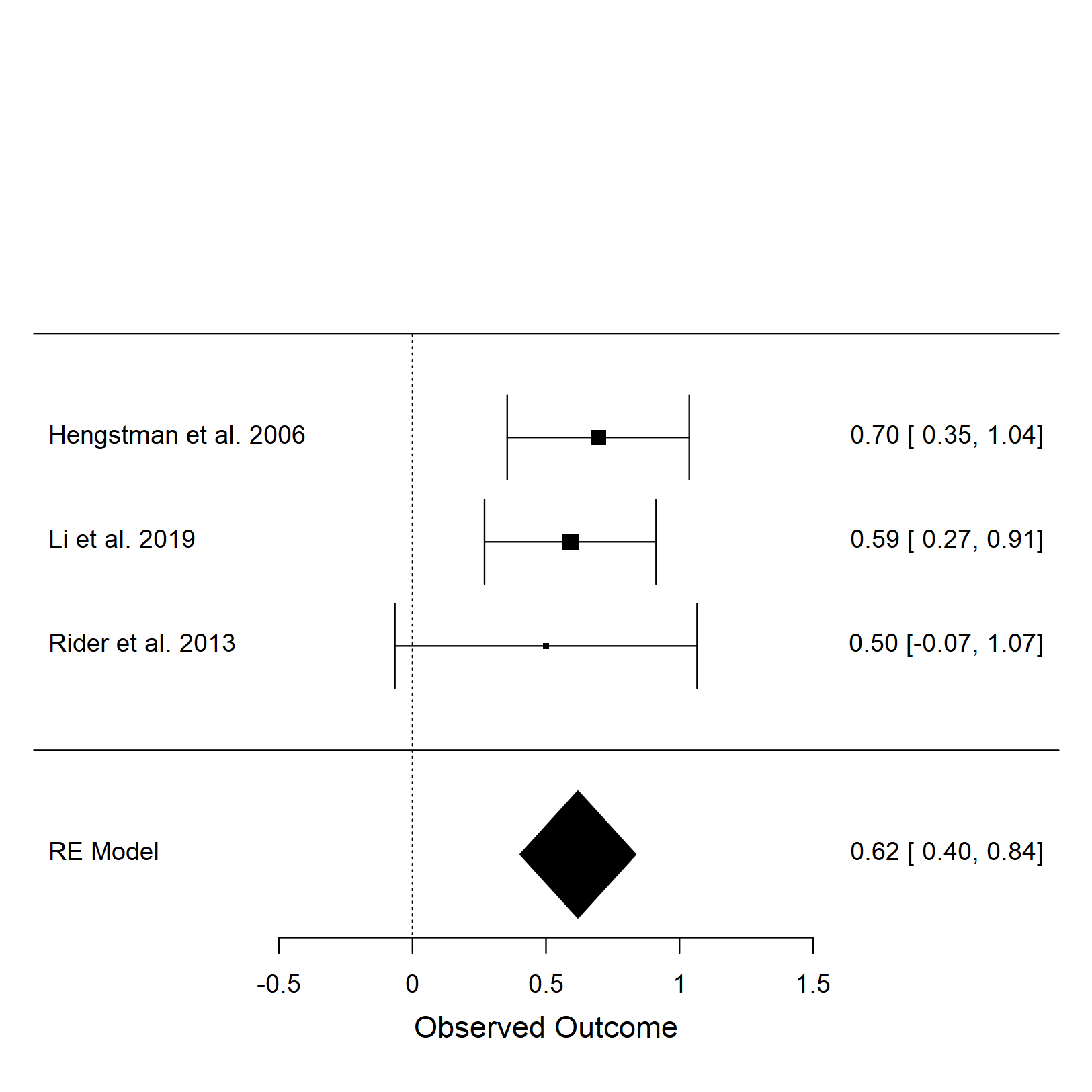


**Funnel plot**


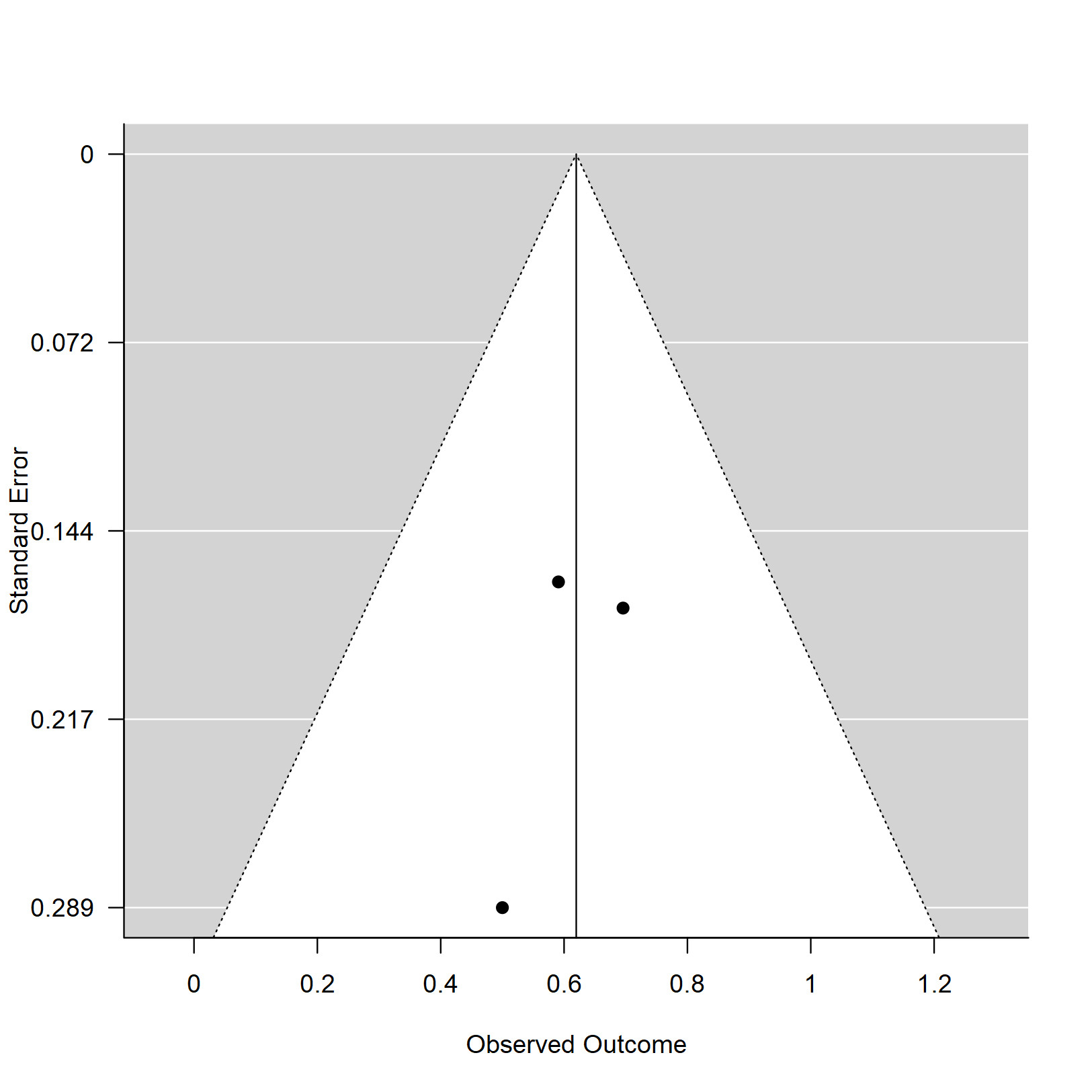


**Meta Analysis SRP -**

| **Fixed and Random Effects** | | | | | | | |
| --- | --- | --- | --- | --- | --- | --- | --- |
|  | | **Q** | | **df** | | **p** | |
| Omnibus test of Model Coefficients |  | 56.410 |  | 1 |  | < .001 |  |
| Test of Residual Heterogeneity |  | 11.120 |  | 2 |  | 0.004 |  |
|  | | | | | | | |
| *Note.*   *p* -values are approximate. | | | | | | | |

| **Coefficients** | | | | | | | | | | | | | |
| --- | --- | --- | --- | --- | --- | --- | --- | --- | --- | --- | --- | --- | --- |
|  | | **Estimate** | | **Standard Error** | | **z** | | **p** | | **Lower Bound** | | **Upper Bound** | |
| intrcpt |  | 0.358 |  | 0.048 |  | 7.511 |  | < .001 |  | 0.264 |  | 0.451 |  |
|  | | | | | | | | | | | | | |
| *Note.*  Wald test. | | | | | | | | | | | | | |

| **Residual Heterogeneity Estimates** | | | | | | | |
| --- | --- | --- | --- | --- | --- | --- | --- |
|  | | **Estimate** | | **Lower Bound** | | **Upper Bound** | |
| *τ²* |  | 0.005 |  | 5.247e -4 |  | 0.257 |  |
| *τ* |  | 0.073 |  | 0.023 |  | 0.507 |  |
| *I²* (%) |  | 80.647 |  | 29.313 |  | 99.510 |  |
| *H²* |  | 5.167 |  | 1.415 |  | 204.253 |  |
|  | | | | | | | |

| **Regression test for Funnel plot asymmetry ("Egger's test")** | | | | | |
| --- | --- | --- | --- | --- | --- |
|  | | **z** | | **p** | |
| sei |  | 1.435 |  | 0.151 |  |
|  | | | | | |

**Plot**

**Forest plot**


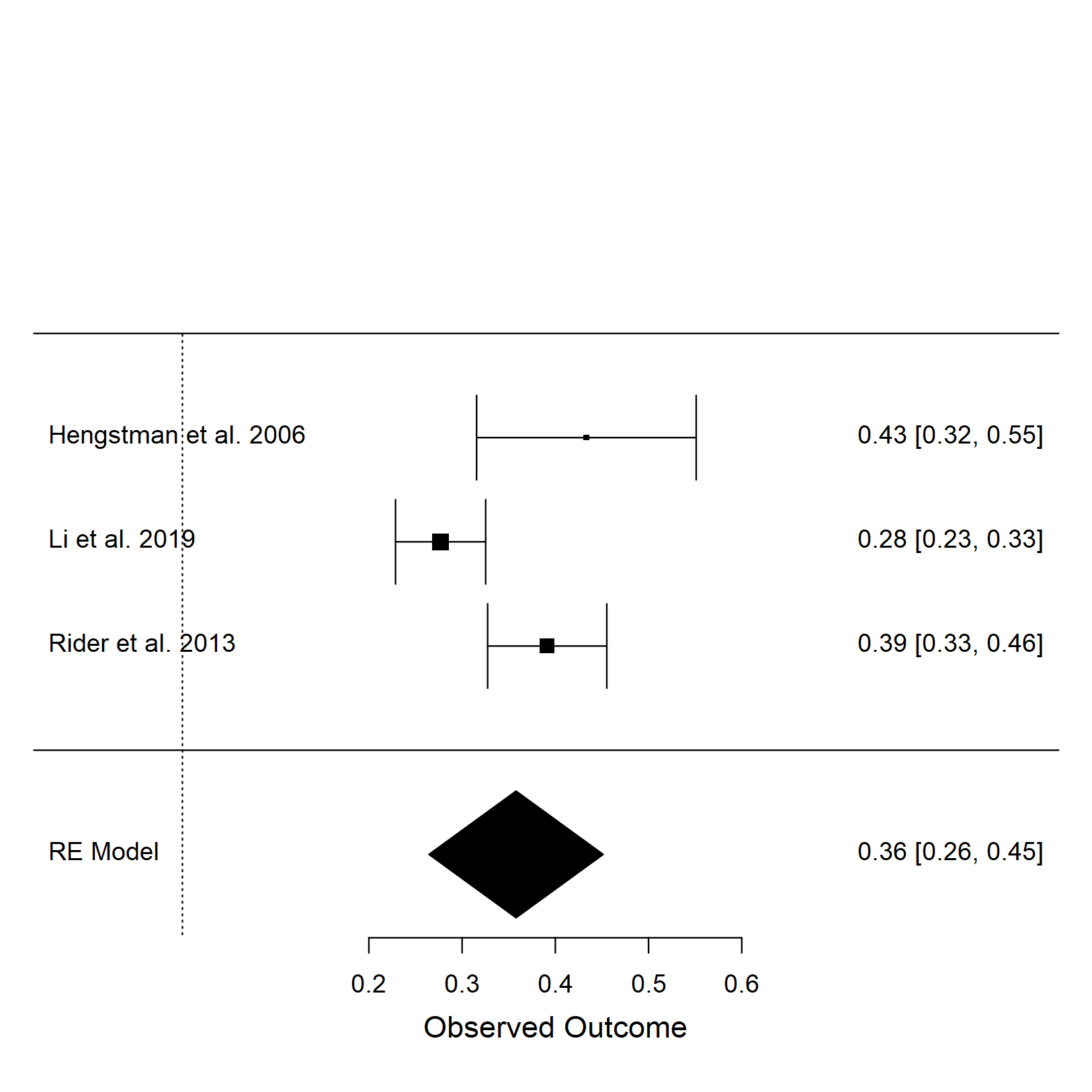


**Funnel plot**


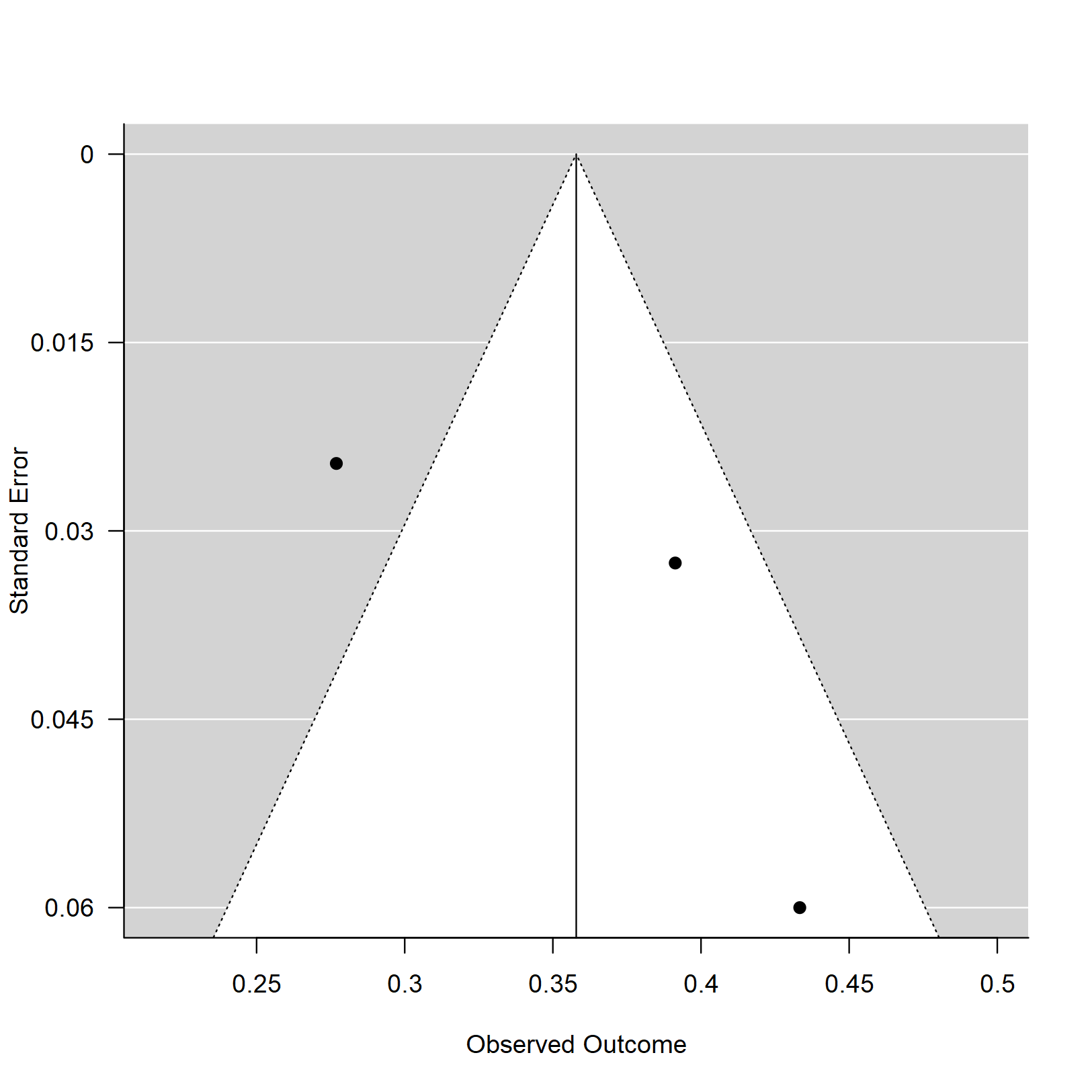


**Meta Analysis TIF +**

| **Fixed and Random Effects** | | | | | | | |
| --- | --- | --- | --- | --- | --- | --- | --- |
|  | | **Q** | | **df** | | **p** | |
| Omnibus test of Model Coefficients |  | 46.790 |  | 1 |  | < .001 |  |
| Test of Residual Heterogeneity |  | 0.210 |  | 2 |  | 0.900 |  |
|  | | | | | | | |
| *Note.*   *p* -values are approximate. | | | | | | | |

| **Coefficients** | | | | | | | | | | | | | |
| --- | --- | --- | --- | --- | --- | --- | --- | --- | --- | --- | --- | --- | --- |
|  | | **Estimate** | | **Standard Error** | | **z** | | **p** | | **Lower Bound** | | **Upper Bound** | |
| intrcpt |  | 0.454 |  | 0.066 |  | 6.840 |  | < .001 |  | 0.324 |  | 0.584 |  |
|  | | | | | | | | | | | | | |
| *Note.*  Wald test. | | | | | | | | | | | | | |

| **Residual Heterogeneity Estimates** | | | | | | | |
| --- | --- | --- | --- | --- | --- | --- | --- |
|  | | **Estimate** | | **Lower Bound** | | **Upper Bound** | |
| *τ²* |  | 0.000 |  | 0.000 |  | 0.045 |  |
| *τ* |  | 0.000 |  | 0.000 |  | 0.212 |  |
| *I²* (%) |  | 0.000 |  | 0.000 |  | 73.051 |  |
| *H²* |  | 1.000 |  | 1.000 |  | 3.711 |  |
|  | | | | | | | |

| **Regression test for Funnel plot asymmetry ("Egger's test")** | | | | | |
| --- | --- | --- | --- | --- | --- |
|  | | **z** | | **p** | |
| sei |  | -0.428 |  | 0.669 |  |
|  | | | | | |

**Plot**

**Forest plot**


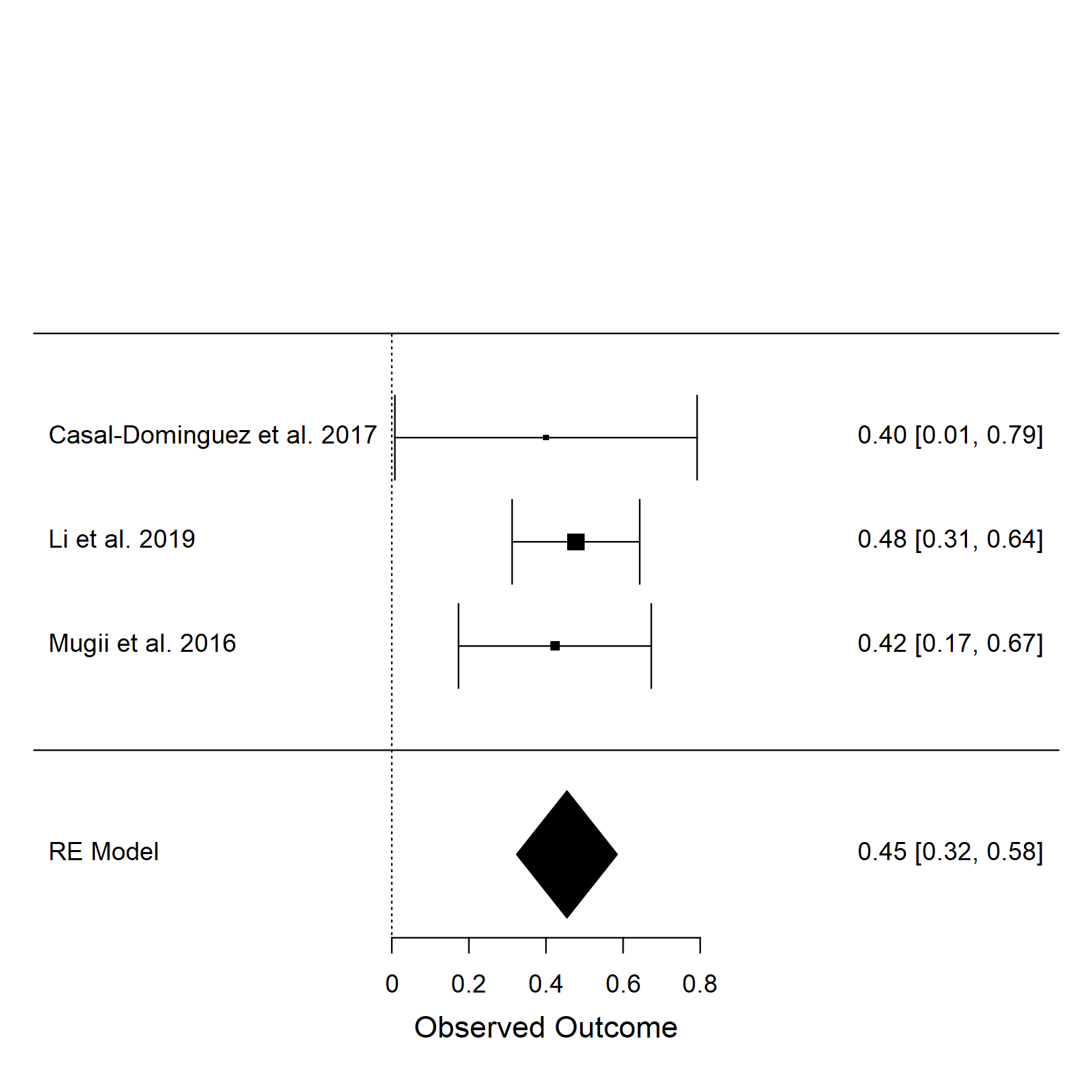


**Funnel plot**


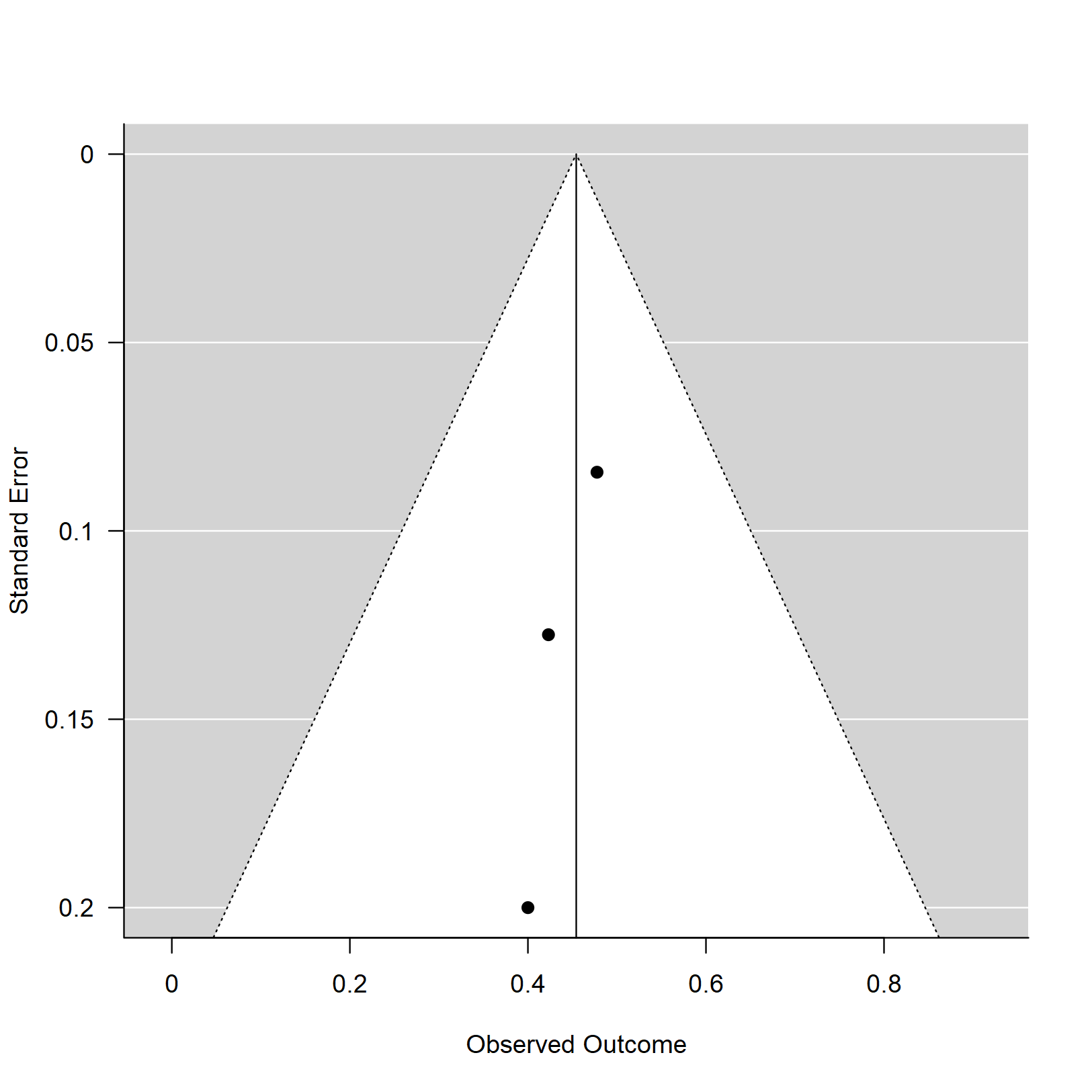


**Meta Analysis TIF -**

| **Fixed and Random Effects** | | | | | | | |
| --- | --- | --- | --- | --- | --- | --- | --- |
|  | | **Q** | | **df** | | **p** | |
| Omnibus test of Model Coefficients |  | 3.798 |  | 1 |  | 0.051 |  |
| Test of Residual Heterogeneity |  | 58.881 |  | 2 |  | < .001 |  |
|  | | | | | | | |
| *Note.*   *p* -values are approximate. | | | | | | | |

| **Coefficients** | | | | | | | | | | | | | |
| --- | --- | --- | --- | --- | --- | --- | --- | --- | --- | --- | --- | --- | --- |
|  | | **Estimate** | | **Standard Error** | | **z** | | **p** | | **Lower Bound** | | **Upper Bound** | |
| intrcpt |  | 0.237 |  | 0.122 |  | 1.949 |  | 0.051 |  | -0.001 |  | 0.475 |  |
|  | | | | | | | | | | | | | |
| *Note.*  Wald test. | | | | | | | | | | | | | |

| **Residual Heterogeneity Estimates** | | | | | | | |
| --- | --- | --- | --- | --- | --- | --- | --- |
|  | | **Estimate** | | **Lower Bound** | | **Upper Bound** | |
| *τ²* |  | 0.041 |  | 0.008 |  | 1.864 |  |
| *τ* |  | 0.202 |  | 0.091 |  | 1.365 |  |
| *I²* (%) |  | 97.564 |  | 89.028 |  | 99.945 |  |
| *H²* |  | 41.045 |  | 9.114 |  | 1829.296 |  |
|  | | | | | | | |

| **Regression test for Funnel plot asymmetry ("Egger's test")** | | | | | |
| --- | --- | --- | --- | --- | --- |
|  | | **z** | | **p** | |
| sei |  | 1.555 |  | 0.120 |  |
|  | | | | | |

**Plot**

**Forest plot**


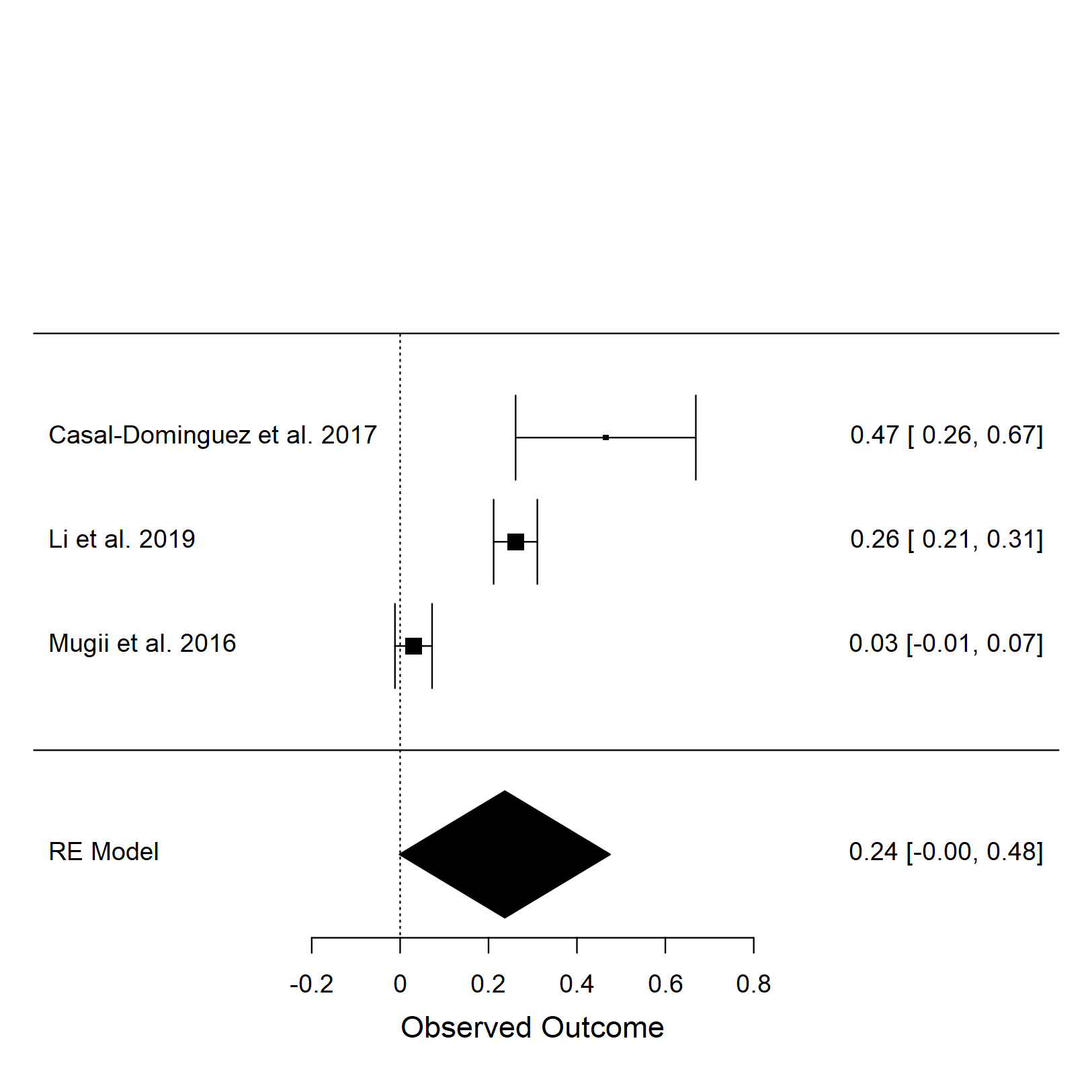


**Funnel plot**


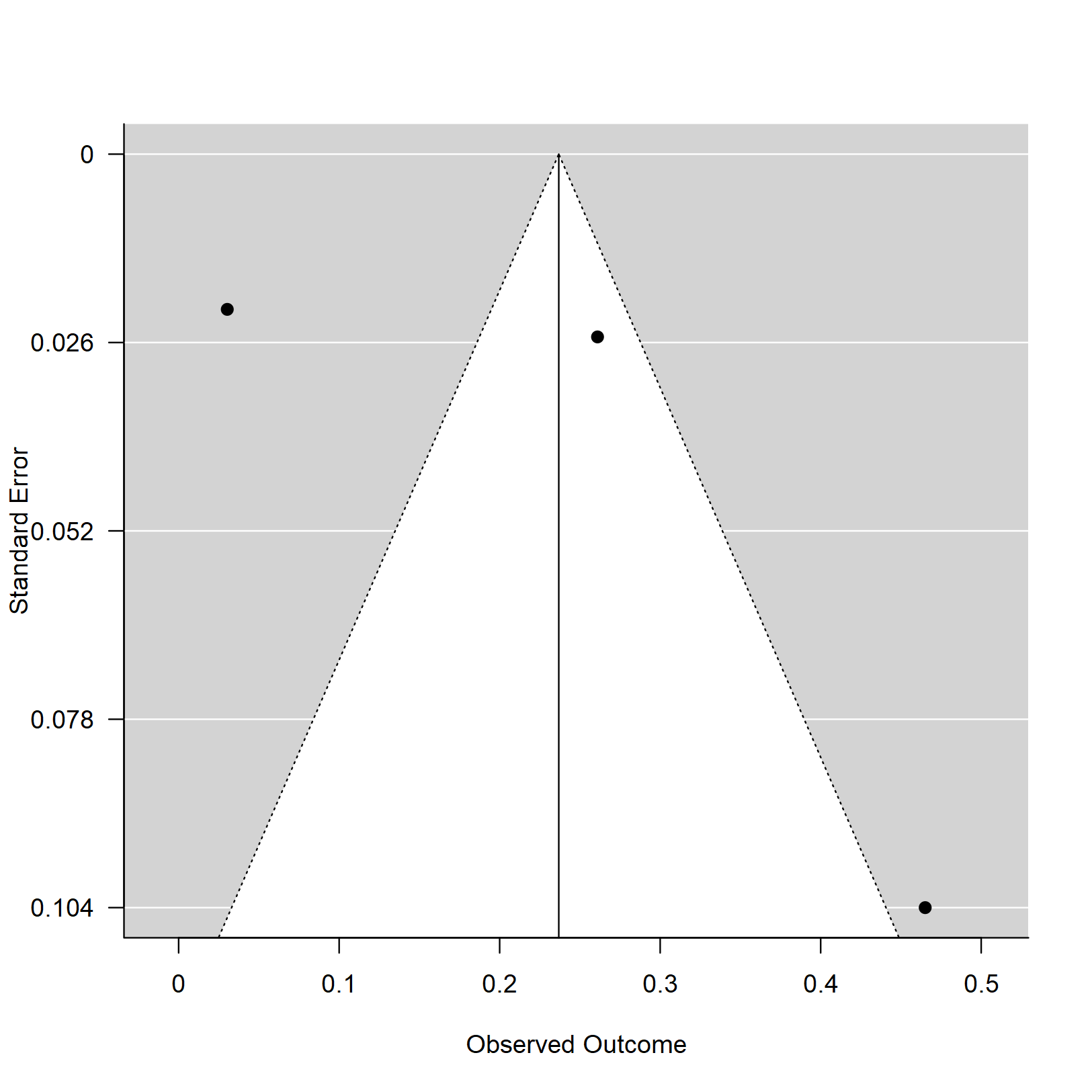

Supplement: Supplementary file 1 [file jcm-09-02150-s001.zip › jcm-843942-SI-conversion/supplements/Document S1.docx]
