# Supplementary material for: The Impact of Dysphagia in Myositis: A Systematic Review and Meta-Analysis
Source: J Clin Med. 2020 Jul 8;9(7):2150. doi: 10.3390/jcm9072150 (PMC7408750; doi:10.3390/jcm9072150)
Supplement: Supplementary file 1 [file jcm-09-02150-s001.zip › jcm-843942-SI-conversion/supplements/Table S1.docx]

**Table S1:** Studies reporting on prevalence and epidemiology of dysphagia in myositis. IIM: idiopathic inflammatory myopathy, DM: dermatomyositis, PM: polymyositis, IBM: inclusion body myositis, JDM: juvenile dermatomyositis, JPM: juvenile polymyositis, CADM: clinically amyopathic dermatomyositis, CAJDM: clinically amyopathic juvenile dermatomyositis, VFSS: videofluoroscopy, FEES: flexible endoscopic evaluation of swallowing, EGD: esophagogastroduodenoscopy; EMG: electromyography, HLA: human leukocyte antigen, MSA: myositis specific antibody, MAA: myositis associated antibody.

| Author | Study design | Cohort | Definition/ assessment of dysphagia | Prevalence and epidemiology of dysphagia |
| --- | --- | --- | --- | --- |
| (Albayda et al. 2017) | retrospective observational study | total cohort, n=235  anti-NXP2-positive DM, n=56 (mean age at onset 46.8 ± 17.7 years; men: 16)  anti-NXP2-negative DM, n=179 (mean age at onset: 44.7 ± 14.8 years; men: 49) | patient report | total cohort: 42%  anti-NXP2-positive DM: 62%  anti-NXP2-negative DM: 35%  significantly higher in anti-NXP2-positive DM |
| (Albrecht et al. 2015) | retrospective observational study | total cohort, n=132 (median age at serum test: 60 years; men: 43)  anti-FHL1-positive IIM, n=33  anti-FHL1-negative IIM, n=96 | not further stated | total cohort: 61%  anti-FHL1-positive IIM: 85%  anti-FHL1-negative IIM: 54%  significantly higher in anti-FHL1-positive IIM |
| (Allenbach et al. 2014) | retrospective observational study | HMGCR-associated necrotizing autoimmune myositis, n=45 (mean age: 48.9 ± 21.9 years; men: 12) | not further stated | 27% |
| (Al-Mayouf et al. 2000) | prospective, non-randomized intervention study | severe JDM, n=12 (mean age at disease onset: 6.5 years; boys: 4) | not further stated | 50% |
| (Al-Mayouf et al. 2017) | retrospective observational study | JDM, n=92 (mean age at diagnosis: 6.6 ± 3.0 years; boys: 34) | not further stated | 15% |
| (Azuma et al. 2011) | retrospective observational study | total cohort, n=136 (mean age: 53 ± 15 years; men: 38)  PM/DM/CADM with malignancy, n=23 (mean age: 61.5 years; men: 10)  PM/DM/CADM without malignancy, n=113 (mean age: 51.1 years; men: 28) | clinically: difficulty in swallowing water or food,  or the requirement for a nasogastric tube | total cohort: 23%  PM/DM/CADM with malignancy: 61%  PM/DM/CADM without malignancy: 15%  significantly higher in patients with malignancy. |
| (Badrising et al. 2005) | prospective observational study | IBM, n=64 (mean age: 67 ± 8 years, men: 43) | standardized questionnaire i.e. a feeling of stasis and experiencing a need to swallow repeatedly, regurgitation or choking more than five times a month | presenting symptom: 9%  current symptom: 66% |
| (Basnayake et al. 2015) | retrospective observational study | anti-SRP-positive IIM, n=5 (mean age: 61.0 ± 11.9 years; men: 5) | clinical symptoms and weight loss | 60% |
| (Benbassat et al. 1985) | retrospective observational study | PM and DM, n=92 (age at diagnosis <20 years: 23; 21-60 years: 46; >61 years: 23; men: 34) | recorded in patient history | 38% |
| (Benveniste et al. 2011) | retrospective observational study | sIBM, n=136 (median age at last visit: 72.5 years; men: 78) | clinical symptoms | dysphagia as only, first symptom: 4%  dysphagia together with muscle weakness as initial symptom: 8%  dysphagia in the further course of the disease: 46% |
| (Betteridge et al. 2009) | retrospective observational study | total cohort, n=129  anti-SAE positive DM, n=9 (mean age: 62.3 ± 11.6; men: 3)  anti-SAE negative DM, n=120 | not further stated | total cohort: 46%  anti-SAE positive DM: 78%  anti-SAE negative DM: 43% |
| (Bodoki et al. 2014) | retrospective observational study | total cohort, n=20 (mean age: 48.8 years; men: 6)  anti-TIF1γ-positive IIM, n=12  anti-NXP2-positive IIM, n=4  anti-SAE-positive IIM, n=4 | not further stated | total cohort: 40%  anti-TIF1γ-positive IIM: 33%  anti-NXP2-positive IIM: 25%  anti-SAE-positive IIM: 75% |
| (Bucelli and Pestronk 2018) | retrospective observational study | total cohort, n=71  immune myopathies with perimysial pathology, n=51 (mean onset age: 47 ± 2 years, men: 17)  DM with vascular pathology, n=20 (mean 45 ± 5 years; men: 4) | VFSS, FEES or expert opinion | total cohort: 58%  immune myopathies with perimysial pathology: 57%  DM with vascular pathology: 60%  no significant difference between the groups |
| (Camargo et al. 2018) | prospective observational study | sIBM, n=18 (mean age of onset: 58.8 ± 10.9; men: 11) | not further stated | 67%  1 patient (6%) as initial symptom |
| (Capkun et al. 2017) | retrospective observational study | sIBM, n=333 (mean age: 69 ± 9.6 years; men: 66%) | not further stated | at diagnosis during the study: 17%  12 months after diagnosis: 23% |
| (Carpenter et al. 1977) | retrospective observational study | PM, n=60 | not further stated | 42% |
| (Casal-Dominguez et al. 2017) | prospective observational study | total Cohort IIM, n=53 (mean age at manometry: 58.3 ± 16.4 years; men: 11)  DM, n=24  (mean age: 62.7 ± 14.3 years; men: 5)  PM, n=21 (mean age: 53 ± 16.1 years; men: 3)  cancer associated myositis, n=5 (mean age: 66.7 ± 20 years)  ASS, n=16 (mean age: 51.9 ± 13.5 years; men: 5)  Anti–TIF1‐γ, n = 10 (mean age: 58.7 ± 22.4 years, men: 0)  Anti‐PM/Scl, n = 8 (mean age: 50.1 ± 17.3 years, men: 2)  Anti‐Ro52, n = 16 (mean age: 57.8 ± 15 years, men: 6) | esophageal symptom survey  HRM | IIM: 45% esophageal symptoms, 45% abnormal esophageal motility  DM: 46% esophageal symptoms, 29% abnormal esophageal motility  PM: 43% esophageal symptoms, 62% abnormal esophageal motility  cancer associated myositis: 80% esophageal symptoms, 46% abnormal esophageal motility  ASS: 38% esophageal symptoms, 38% abnormal esophageal motility  anti–TIF1‐γ: 70% esophageal symptoms, 40% abnormal esophageal motility  anti‐PM/Scl: 38% esophageal symptoms, 38% abnormal esophageal motility  anti‐Ro52: 56% esophageal symptoms, 38% abnormal esophageal motility  differences in esophageal symptoms between the clinical and serologic groups were not statistically significant  abnormal esophageal HRM in PM significantly higher than in DM. |
| (Challa et al. 2018) | retrospective observational register- study | JDM, n=65 (mean age at baseline: 9.3 ± 4.4 years; men: 22) | dysphagia and dysphonia, not further reported | 6% |
| (Chen et al. 2014) | retrospective observational study | DM, n=246 (mean age at diagnosis: 49.2 ± 12.7 years; men: 107)  DM with malignancy, n=60 (mean age: 49.2 ± 12.7 years; men: 41)  DM without malignancy, n=186 (mean age: 44.6 ± 12.1 years; men: 66) | not further stated | 34%  DM with malignancy: 47%  DM without malignancy: 30%  significantly higher in patients with malignancy |
| (Cherin et al. 2015) | case-series | IBM with subcutaneous immunoglobulin therapy, n=6 (mean age: 66.5 years; men: 3) | not further stated | 50% |
| (Chickermane et al. 2013) | retrospective observational study | JDM, n=22 (mean age at diagnosis: 7.5 ± 3.99 years; men: 12) | tube-dependent | 23% |
| (Chiu et al. 2007) | retrospective observational study | JDM, n=21 (mean age at diasease onset: 6.4 ± 3.7 years, men: 4) | not further reported | 24%  19% at disease onset |
| (Chung et al. 1994) | retrospective observational study | JDM, n=7 (mean age: 11.86 years; men: 3)  JPM, n=2 (mean age: 12.5 years, men: 0) | not further stated | total cohort: 11%  JDM: 0%  JPM: 50% |
| (Chwalinska-Sadowska and Maldykowa 1990) | Prospective observational study | total cohort IIM, n=50 (age range at disease onset: 17-64 years; men: 14)  PM, n=17 (men: 4)  DM, n=33 (men: 10) | not further stated | PM as presenting symptom: 0%  DM as presenting symptom: 21%  PM in the course of the disease: 18%  DM in the course of the disease: 55% |
| (Cox et al. 2009) | prospective observational study | IBM, n=57 (67 ± 8 years for men, n = 41, and 71 ± 10 years for women, n = 16) | standard questionnaire regarding dysphagia, VFSS | 7% dysphagia as presenting symptom  65% had symptoms of dysphagia, of these 56% did not spontaneously report the symptoms  79% abnormal VFSS finding |
| (Cunningham, JR and Lowry 1985) | retrospective observational study | total cohort, n=45 (mean age: 44.7 years; men: 10)  PM, n=16 (mean age: 48.6 years; men: 4)  DM, n=16 (mean age: 43.9 years; men: 3)  myositis with calcinosis, n=7  childhood myositis, n=2  overlap syndrome, n=4 | not further stated | total cohort: 27%  PM: 13%  DM: 31%  myositis with calcinosis: 43%  childhood myositis: 0%  overlap syndrome: 50% |
| (Dabby et al. 2001) | retrospective observational study | IBM initially misdiagnosed as motor neuron disease, n=9 (mean age at onset: 61 years; men: 6) | not further stated | 45%  at onset: 0% |
| (Danieli et al. 2009) | prospective interventional study | PM/DM, n=7 (mean age: 49.3 years; men: 0) | not further stated | total cohort: 43% |
| (Danko et al. 2004) | prospective observational study | IIM, n=162 (mean age of diagnosis: 39.2 ± 13.6 years; men to women ratio: 1: 2.1)  PM, n=75 (mean age of diagnosis: 39.3 ± 11.1 years; men to women ratio: 1:1.8)  DM, n=42 (mean age of diagnosis: 43.7 ± 12.4 years; men to women ratio: 1:2.8) | dysphagia-clinical history of disordered swallowing, requirement for nasogastric tube | IIM: 19%  PM: 9%  DM: 36%  significantly higher in DM |
| (Davis and Ahmed 1997) | retrospective observational study | DM and PM associated with ovarian malignancy, n=14 (mean age at diagnosis: 59 years) | not further stated | 36% |
| (de Souza, Fernando Henrique Carlos et al. 2018) | retrospective observational study | IIM, n=38 (mean age: 42.6 ± 10.9 years; men: 6) | not further stated | 71% |
| (de Souza, Fernando Henrique Carlos et al. 2017) | retrospective observational study | anti-SRP positive necrotizing myopathy, n=14 (mean age at disease onset of 40.7 years; men: 2) | not further stated | 50% |
| (Degos et al. 1971) | retrospective observational study | DM, n=31 (mean age: of onset of disease: 49 years; men: 8) | not further stated | 52% |
| (Della Marca et al. 2013) | prospective observational study | sIBM, n=13 (mean age: 66.2 ± 11.1 years, men: 7) | oro-pharyngo-esophageal scintigraphy | 62% |
| (Dobloug et al. 2015a) | retrospective observational study | mixed group of PM (n=100) and DM (n=130) (mean age at diagnosis: 53.5 years; men: 80) | not further stated | 23% at onset, 58% during follow-up |
| (Dobloug et al. 2012) | retrospective observational study | sIBM, n=14; n=6 | total cohort, n=20  subjective report of patients n=6  radiological dynamic studies of the esophagus n=14 | total cohort: 80%  n=6 based on subjective report: 67%  n=14 based on radiological dynamic studies: 86% |
| (Dobloug et al. 2015b) | retrospective observational study | total cohort, n=89 (mean age at diagnosis: 66.9 ± 9.3 years; men to women ratio: 1.5:1)  sIBM female, n=35  sIBM male, n=54 | partly examined by dynamic x-ray of the esophagus, otherwise not further stated | total cohort: 76%  sIBM female: 94%  sIBM male: 65%  significantly higher in sIBM female |
| (DONOGHUE et al. 1960) | retrospective observational study | DM and PM, n=38 | Clinical signs, roentgenography of esophagus (n=35), esophagoscopy (n=6), or motility studies (n=17) | 84% clinical signs for dysphagia  46% abnormal roentgenography  33% in esophagoscopy  47% in motility studies |
| (Dos Passos Carvalho, Maria Isabel Cardoso and Shinjo 2019) | retrospective observational study | total cohort, n=87 (mean age at diagnosis: 42.4 ± 13.8 years; men: 23)  anti-Mi-2-positive DM, n=17 (mean age at diagnosis: 43.1 ± 13.7 years; men: 4)  anti-Mi-2-negative DM, n=70 (mean age at diagnosis: 42.3 ± 14.0; men: 29) | not further stated | total cohort: 49%  anti-Mi-2-positive DM: 65%  anti-Mi-2-negative DM: 46%  no significant difference between the groups |
| (Dunlap et al. 2014) | retrospective observational study | sIBM, n=53 (mean age at onset of symptoms: 60.9 ± 1.34; men: 39) | symptoms of swallowing difficulty | 59%  13% as first symptom  the prevalence of dysphagia increased as patients required more supportive gait devices |
| (Ertekin et al. 1996) | prospective observational study | DM/PM, n=7 | tube-dependent  decreased dysphagia limit in EMG | tube dependent: 71%  decreased dysphagia limit in EMG: 57% |
| (Ertekin et al. 2004) | prospective observational study | DM/PM, n=19 (mean age: 48.7 years, men: 6) | submental EMG  clinical parameters | 63% tube-dependent  74% piecemeal deglutition when swallowing 20ml of water |
| (Fang et al. 2016) | retrospective observational study | total cohort, n=192 (mean age at onset of disease: 47.3 ± 18.5 years; men: 64)  PM and DM with cancer, n=33 (mean age at onset of disease: 55.4 ± 13.1; men: 15)  PM and DM without cancer, n=159 | not further stated | total cohort: 38%  PM and DM with cancer: 55%  PM and DM without cancer: 35%  significantly higher in PM and DM with cancer |
| (Felice and North 2001) | retrospective observational study | sIBM, n=35 (mean age: 70.0 years; men: 23) | Clinical symptoms  Partly VFSS | 54% |
| (Fujimoto et al. 2013) | retrospective observational study | SAE associated DM, n=7 (median age: 67 years; men: 3) | not further stated | 29% |
| (Galindo-Feria et al. 2016) | retrospective observational study | total cohort, n=333  DM without JDM, n=264 (median aga at diagnosis: 40 years; men: 63)  PM, n=69 (median aga at diagnosis: 46 years, men: 18) | not further stated | total cohort: 47%  DM: 50%  PM: 36%  significantly higher in patients with DM |
| (Gao et al. 2014) | retrospective observational study | total cohort, n=91 (median age:51 ± 15.8 years; men: 25)  DM, n=71  PM, n=20 | not further stated | total cohort: 15%  DM: 14%  PM: 20%  the frequency of HLA-DRB1*07 was significantly higher among IIM patients with dysphagia |
| (Ge et al. 2015) | retrospective observational study | total cohort, n=365  anti-HMGCR-positive IIM, n=20  anti-HMGCR-negative IIM, n=345 | for the anti-HMGCR-positive patients: difficulty swallowing solid food, choking or coughing when drinking water, requiring nasogastric tube | total cohort: 22%  anti-HMGCR-positive IIM: 50%  anti-HMGCR-negative IIM: 20%  significantly higher in anti-HMGCR-positive IIM |
| (Ge et al. 2017) | retrospective observational study | total cohort, n=339  anti-SAE-positive DM, n=11 (mean age: 59.1 years, men: 3)  anti-SAE-negative IIM, n=328 (mean age: 48.6 ± 14.0 years; men: 105) | anti-SAE-positive patients: difficulty in digesting solid food; coughing when drinking water or depending on. nasogastric tubes | total cohort: 27%  anti-SAE-positive DM: 64%  anti-SAE-negative IIM: 25%  significantly higher in anti-SAE-positive DM |
| (Goyal et al. 2016) | prospective observational study | total cohort, n=25  anti- NT5c1A- positive sIBM, n=18 (mean age: 67.0 years; men: 13)  antiNT5c1A-negative sIBM, n=7 (mean age: 70.0 years; men: 6) | self-report | total cohort: 76%  anti- NT5c1A- positive sIBM: 89%  anti- NT5c1A- negative sIBM: 43%  significantly higher in patients with anti- NT5c1A- positive sIBM |
| (Gupta and Gow 2013) | retrospective observational study | total cohort, n=12  PM, n=7 (mean age: 49 years, men: 3)  DM, n=4 (mean age: 62 years; men: 3)  IBM, n=1 (mean age: 66 years, men: 0) | not further stated | total cohort: 17%  PM: 14%  DM: 25%  IBM: none |
| (Hajialilo et al. 2018) | retrospective observational study | PM and DM, n=76 (mean age at diagnosis: 45.5 ± 10.9; men-women ratio: 1:5.3) | not further stated | 20% |
| (Hengstman et al. 2006) | retrospective observational study | total cohort, n=143  anti-SRP positive IIM, n=23 (mean age at onset: 47.7 years; men-women-ratio: 1:3.6)  anti-SRP negative IIM, n=120 (mean age at onset: 47.9 years; men-women-ratio: 1:2.4) | not further stated | total cohort: 48%  anti-SRP positive IIM: 69%  anti-SRP negative IIM: 43%  significantly higher in patients with anti-SRP positive IIM |
| (Hochberg et al. 1986) | retrospective observational study | total cohort, n=76 (mean age: 45.3 years; men: 19)  PM, n=31 (mean age: 50.0 years; men: 11)  DM, n=21 (mean age: 38.8 years; men: 4)  cancer-associated IIM, n=6 (mean age: 57.1 years; men: 2)  overlap syndrome, n=18 (mean age: 40.9 years; men: 2) | not further stated | total cohort: 45%  PM: 32%  DM: 52%  cancer-associated IIM: 67%  overlap syndrome, n=50% |
| (Hoesly et al. 2019) | retrospective observational study | total cohort, n=231 (median age: 59 years, men: 46)  ANA-positive DM, n=140 (median age: 39 years; men: 25)  ANA-negative DM, n=91 (median age: 59 years; men: 21) | not further stated | total cohort: 19%  ANA-positive DM: 15%  ANA-negative DM: 26%  significantly higher in ANA-negative patients |
| (Horowitz et al. 1986) | Prospective observational study | PM and DM, n=13 (median age: 53 years; men: 3) | questionnaire on nasal regurgitation, dysphagia (including pre-esophageal dysphagia), heartburn, acid regurgitation, anorexia, nausea, early satiety, upper abdominal discomfort or distention, vomiting, and abdominal pain.  measurement of esophageal emptying using scintigraphy | according to questionnaire: 38%  delayed esophageal emptying according to scintigraphy: 62% |
| (Houser et al. 1998) | retrospective and prospective observational study | IBM, n=22 (men: 13) | chart documentation and telephone interview | according to chart documentation: 36%  according to telephone interview (n=19): 84% |
| (Hu et al. 2019) | prospective observational study | total cohort, n=184 (mean age: 47.9 ± 13.8 years, men: 47)  KL-6-positive IIM, n=84 (mean age: 50.3 ± 12.2 years; men: 23)  KL-6-negative IIM, n=100 (mean age: 45.8 ± 14.8; men: 24) | not further stated | total cohort: 41%  KL-6-positive IIM: 48%  KL-6-negative IIM: 35%  significantly higher in patients with KL-6-positive IIM |
| (Jacob et al. 1983) | prospective observational study | PM or DM, n=13 (mean age: 60.5; men: 0) | questionnaire about dysphagia for solids or liquids, nasal regurgitation, pre-esophageal dysphagia, regurgitation, and heart burn (requiring and relieved by antacids).  esophageal manometry  barium swallow examination  partly VFSS | symptoms of dysphagia: 69 %: 46% had cervical dysphagia; 31% nasal regurgitation; 38% esophageal dysphagia, 8% had aspiration pneumonia  abnormalities in VFSS: 75% (6 of 8) |
| (Johnson et al. 2012) | retrospective observational study | IIM, n=53  IIM, n=118 | dysphagia for solid food by history, esophageal dilation on radiograph, barium swallow or high-resolution computer tomography | 32%  20% |
| (Kang et al. 2005) | retrospective observational study | total cohort, n=72 (mean age at diagnosis: 43.7±14.3; men: 14)  PM/DM with intestinal lung disease, n=29 (mean age at diagnosis: 45.1±13.6; men: 5)  PD/DM without intestinal lung disease, n=43 (mean age at diagnosis: 42.7±14.8; men: 9) | not further stated | total cohort: 15%  PM/DM with intestinal lung disease: 3%  PD/DM without intestinal lung disease: 23%  significantly higher in patients without intestinal lung disease |
| (Kang et al. 2016) | retrospective observational study | total cohort (PM and DM), n=52  cancers concurrent with active myositis, n=30 (mean age: 60.5 ± 11.1 years; men: 13)  cancers non-concurrent with active myositis, n=22 (mean age: 49.3 ± 16.6 years, men: 5) | not further stated | total cohort: 38%  cancers concurrent with active myositis: 57%  cancers non-concurrent with active myositis: 14%  significantly higher in cancers concurrent with active myositis |
| (Karri et al. 2015) | retrospective observational study | DM, n=45 (mean age: 42.0; men: 10) | not further stated | 22% |
| (Kim et al. 2014) | retrospective observational study | total cohort, n=61  DM, n=36 (mean age: 45.3 ± 19.7 years; men: 14)  PM, n=25 (mean age: 49.3 ± 18.1 years; men: 10)  IIM with malignancy, n=10 (mean age: 61.4 ± 10.7 years; men: 5)  IIM without malignancy, n=51 (mean age: 44.1 ± 19.1 years; men: 19) | not further stated | total cohort: 26%  DM: 25%  PM: 28%  IIM with malignancy: 80%  IIM without malignancy: 16% |
| (Kim et al. 2010) | retrospective observational study | DM, n=13 (mean age 37.2 ± 15.9 years; men: 6) | VFSS  American Speech-Language-Hearing Association (ASHA) level | According to ASHA-level < 6: 62%, 31% tube dependent |
| (Lakhanpal et al. 1987) | retrospective observational study | autopsies of patients with PM (n=24) and DM (n=41) (mean age at time of death in the total cohort: 57 years, men: 24) | not further stated | total cohort: 52%  PM: 54%  DM: 51% |
| (Langdon et al. 2012) | prospective observational study | IIM, n=18 (mean age: 58.9 years; men: 8) | VFSS  esophageal manometry | 72% |
| (Li et al. 2019) | retrospective observational study | total cohort, n=477  IIM with MSAs, n=325 (mean age: 49.4 ± 14.9 years; men: 101)  IIM without MSAs, n=172 (44.5 ± 15.5 years, men: 72) | not further stated | total cohort: 29%  IIM with MSAs: 35%  IIM without MSAs: 20%  IIM with anti-Mi-2: 23%  IIM with anti-TIF-1y: 48%  IIM with anti-MAD5: 14%  IIM with anti-NXP2: 54%  IIM with anti-SAE: 75%  IIM with anti-SRP: 49%  IIM with Anti-HMGCR: 50%  IIM with anti-Jo-1: 16%  IIM with anti-PL-7: 35%  IIM with anti-PL12: 11%  IIM with anti-EJ: 23%  IIM with anti-OJ: 0%  significantly higher in patients with MSAs  associated with dysphagia: anti-TIF1-γ, anti-NXP2, anti-SAE1, anti-SRP, and anti-HMGCR  associated with absence of dysphagia: anti-MDA5 |
| (Lilleker et al. 2018) | retrospective observational register-study | IIM, n=1945 (mean age approx. 49 years; men: approx. 665)  DM, n=unclear  PM, n=unclear  IBM, n=224  ASS, n=unclear  JDM, n=unclear  connected tissue disease-overlap, n=258 | not further stated | IIM: 39%  DM: 43%  PM: 35%  IBM: 50%  ASS: 26%  JDM: 16%  connected tissue disease-overlap: 53%  significantly higher in patients with IBM and connected tissue disease-overlap  age at disease onset, the rate of smokers and the number of applied medications was significantly higher in patients with dysphagia. |
| (Lindberg et al. 1994) | retrospective observational study | IBM, n=18 (mean age: 60.4 years; men: 10) | symptoms according to patient history | 73% |
| (Liu et al. 2019) | retrospective observational study | anti-NXP-2-positive DM, n=16 (mean age: 30.8 years; men: 5) | not further stated | 50% |
| (Lotz et al. 1989) | retrospective observational study | IBM, n=40 (mean age at onset of symptoms: 56.1 years; men: 29) | partly barium swallow | at disease onset: 10%  at diagnosis: 40% |
| (Lynn et al. 2005) | retrospective observational study | IIM, n=44 (age at presentation ranging from 36-81 years; men: 20%) | not further stated | dysphagia as presenting symptom: 32% |
| (Mamyrova et al. 2018) | retrospective observational study | CAJDM, n=12 (median age at diagnosis: 4.1 years; boys: 2)  JDM, n=60 (median age at diagnosis: 7.3; boys: 17) | not further stated | CAJDM: 0%  JDM: 32%  significantly higher in JDM |
| (Marie et al. 2002) | retrospective observational study | PM/DM, n=156 | esophageal dysfunction but not further stated how evaluated | esophageal involvement: 38% |
| (Marie et al. 2001) | retrospective observational study | PM/DM, n=77 | manometric esophageal impairment | 29 % esophageal involvement |
| (Marie et al. 1999) | retrospective observational study | DM/PM, n=79 (median age: 52 years; men: 37)  DM/PM > 65 years, n=23 (mean age: 70.7 years; men: 9)  DM/PM < 65 years, n=56  DM/PM with cancer > 65 years, n=11 (mean age: 72.4 years; men: 5)  DM/PM without cancer > 65 years, n=12 (mean age: 69.3 years; men: 4) | esophageal manometry | DM/PM with esophageal hypomotility: 25%  DM/PM > 65 years with esophageal hypomotility: 35%  DM/PM < 65 years with esophageal hypomotility: 16%  DM/PM with cancer > 65 years: 45%  DM/PM without cancer > 65 years: 25%  significantly higher in patients > 65 |
| (Marie et al. 2010) | retrospective observational study | PM/DM, n=301 (median age: 50 years in patients with esophageal impairment; 51 years in patients without impairment) | esophageal impairment defined by clinical manifestation in combination with abnormal finding in esophageal manometry and exclusion of other causes in gastroscopy | 39 % with esophageal impairment  18% with esophageal impairment at onset of the disease |
| (Maugars et al. 1996) | retrospective observational study | IIM, n=69 (mean age at onset: 47.4 ± 20.7 years; men: 20)  PM, n=13  DM, n=27 | not further stated | IIM: 36%  DM: 46%  PM: 14%  dysphagia as presenting symptom: 3% |
| (McCann et al. 2007) | prospective observational study | JDM, n=14 (mean age at onset: 7.2 years; boys: 5) | VFSS | 64% showed clinical symptoms of dysphagia  79% signs of dysphagia in VFSS (including 14% without clinical symptoms)  no correlation between symptoms and VFSS findings |
| (Medsger, JR et al. 1971) | retrospective observational study | PM, n=74 | not further stated | 42% |
| (Merieux et al. 1983) | observational study with prospective and retrospective cohort | PM or DM with incomplete response to cortisone in the prospective cohort, n=16 (average age: 46 years; men: 2)  autopsy of patients with PM or DM, n=18 (mean age: 53 years; men: 9) | clinical symptoms and barium swallow with cine esophagram | symptoms of dysphagia in the prospective cohort: 56%  abnormalities in VFSS in the prospective cohort: 88% (all abnormalities e.g. hiatal hernia are included)  symptoms of dysphagia in the autopsy cohort: 72% |
| (Milisenda et al. 2014) | case-series | DM with subcutaneous edema, n=5 (mean age: 73 years; men: 1) | not further stated | 60% |
| (Moghadam-Kia et al. 2017) | retrospective observational study | total cohort, n=122  CADM, n=61 (mean age: 48.2 ± 16.9 years; men: 22)  DM, n=61 (mean age: 44.8 ± 17.6 years)  MDA5-positive CADM or DM, n=16  MDA5-negative CADM or DM, n=106 | not further stated | total cohort: 39%  CADM: 28%  DM: 5%  MDA5-positive CADM or DM: 31%  MDA5-negative CADM or DM: 14%  significantly higher in CADM compared to DM |
| (Mugii et al. 2016) | prospective observational study | DM, n=92 (mean age: 54.9 ± 16.2 years; men: 20)  ARS-positive, n=23  TIF1y positive, n=26  MDA5 positive, n=15  Ro60 positive, n=5  U1-RNP positive, n=5  internal malignancy, n=19 | clinical observation by otorhinolaryngologists and speech therapists  partly VFSS | 14% due to clinical signs  ARS-positive: 4%  TIF1y positive: 42%  MDA5 positive: 0%  Ro60 positive: 0%  U1-RNP positive 0%  internal malignancy: 58%  significant association of dysphagia with age, male gender, malignancy, anti-TIF-1γ antibody, and reduced MMT scores of sternomastoid and deltoid muscles, absence of intestinal lung disease. |
| (Mulcahy et al. 2012) | prospective observational study | IIM, n=18 (mean age: 58.9 ± 13.3 years; men: 8)  PM, n=6 (men age: 51.8 years; men: 1)  DM, n=4 (mean age: 57 years; men: 1)  IBM, n=8 (men age: 65.1 years; men: 6) | subjective self-report  VFSS | IIM: self-report: 94%, VFSS: 78%  PM: 66% (in VFSS)  DM: 50% (in VFSS)  IBM: 100% (in VFSS) |
| (Murata et al. 2012) | prospective observational study | sIBM, n=10 (mean age: 70.5 ± 7.1 years; men: 5) | VFSS  computed pharyngoesophageal manometry | subjective report: 50%  VFSS: 100% |
| (Muro et al. 2015) | retrospective observational study | SAE associated DM, n=7 (mean age: 65; men: 4) | not further stated | 43% |
| (Mustafa and Dahbour 2010) | retrospective observational study | total cohort, n=30 (mean age at onset: 34.3 ± 9.2 years; men: 11)  PM, n=11  DM, n=19 | not further stated | total cohort: 40%  PM: 18%  DM: 53% |
| (Na et al. 2009) | retrospective observational study | total cohort: n=64 (mean age: 36.4 years; men: 23)  JDM, n=16  adult DM, n=48  DM with malignancy, n=6 (mean age: 54.7 years; men: 2)  DM without malignancy, n=58 (mean age: 34.5 years; men: 21) | not further stated | total cohort: 23%  JDM: 38%  adult DM: 19%  DM with malignancy: 33%  DM without malignancy: 22% |
| (Narayanaswamy et al. 1993) | retrospective observational study | PM/DM, n=24 (age: 20-40 years; men: 18) | not further stated | 21% |
| (Needham et al. 2008) | retrospective and prospective observational study | sIBM, n=57 | not further stated | 58%  2% as initial symptom |
| (Neri et al. 2014) | retrospective observational study | IIM, n=162 (mean age at onset of myositis: approx. 52.3 years; men: 55)  cancer-associated IIM, n=27 (mean age at onset of myositis: approx. 59.0 years; men: 11)  non-cancer IIM, n=135 (mean age at onset of myositis: approx. 51.0 years; men: 44)  PM, n=89 (mean age at onset of myosits: approx. 50.1 years; men: 35)  DM, n=73 (mean age at onset of myositis: approx. 55.1 years; men: 20) | self-report | IIM: 22%  cancer-associated IIM: 37%  Non-cancer IIM: 19%  PM: 21%  DM, n=22%  significantly higher in patients with cancer associated IIM |
| (Noguchi et al. 2017) | retrospective observational study | ASS, n=51(mean age at disease onset 60.2 ± 16.1 years; men: 20)  anti-OJ-positive ASS, n=14 | not further stated | ASS: 29%  anti-OJ-positive ASS: 57%  significantly higher in anti-OJ-positive ASS  significantly lower in patients with ASS compared to patients with anti-SRP-positive IIM |
| (Nuno-Nuno et al. 2019) | retrospective observational study | total cohort, n=341 (median age at diagnosis: 49.1 years, men: 76)  overlap myositis, n=98 (median age at diagnosis: 42.2 years; men: 14)  PM, n=137 (median age at diagnosis: 56.1 years; men: 41)  DM, n=106 (median age at diagnosis: 51.5; men: 21) | not further stated | total cohort: 28%  overlap myositis: 42%  PM: 18%  DM: 26%  significantly higher in patients with overlap myositis |
| (Ogawa-Momohara et al. 2019) | retrospective observational study | DM, n=85 (mean age at diagnosis: 61.0 ± 13.9 years; men: 28) | symptoms according to physician, otolaryngologist or speech language pathologist | 35% |
| (Oh et al. 2008) | retrospective observational study | IBM with Dysphagia, n=26 | partly clinical dysphagia evaluation (n=24), VFSS (n=23), and pharyngoesophageal  manometry (n=12) | not applicable  42% had dysphagia as initial symptom  8% had dysphagia as only symptom |
| (Oh et al. 2007) | retrospective observational study | IIM with dysphagia, n=62 | Partly VFSS (n=38) | not applicable  Dysphagia as presenting symptom: 21% (significantly more often in IBM) |
| (Okada et al. 2014) | retrospective observational study | total cohort, n=41 (men: 16)  DM patients with CC genotype of miR‐146a, n=9 (mean age: 58.7 years)  DM patients with CG or GG genotype of miR‐146a, n=16 (mean age: 54.6 years)  amyopathic DM, n=16 (mean age: approx.45.4 years) | not further stated | total cohort: 17%  DM patients with CC genotype of miR‐146a: 67%  DM patients with CG or GG genotype of miR‐146a: 9%  amyopathic DM: 0%  significantly higher in DM patients with CC genotype of miR‐146a |
| (Olthoff et al. 2016) | prospective observational study | IBM without severe aspiration according to patient history, n=20 (mean age: 72 ± 7 years; men: 12) | Swallowing-Related Quality of Life scale  VFSS  FEES  Real-time-MRI | Swallowing-Related Quality of Life scale: 80%  average percentage of patients with retention according the different instrumental diagnostic approaches and two raters: 88%   - VFSS: 19/19 - MRI: 17/20 - FEES: 15/19 |
| (Ozonoff and Flynn, JR 1973) | retrospective observational study | JDM (“DM of childhood”), n=10 (mean age of onset: 7 years; boys: 3) | not further stated | 20% |
| (Pachman et al. 1998) | retrospective observational study with telephone interview | JDM, n=79 (mean age: 6.9 ± 3.8 years; boys: 24) | not further stated | 44% |
| (Peng et al. 2000) | retrospective observational study | sIBM, n=78 (mean age at disease onset: 57.0 ± 8.8 years; men: 61) | not further stated | 30% |
| (Peng et al. 2014) | prospective observational study | mixed group of PM and DM, n=98 | not further stated | 36%  patients with oropharyngeal dysphagia had significantly higher Fn14 mRNA levels than the patients without oropharyngeal dysphagia |
| (Ponyi et al. 2005) | retrospective observational study | total cohort, n=84  cancer associated DM, N=16 (mean age at onset: 56.0 ± 11.0 years; men: 8)  DM without cancer, n=68 (mean age at onset: 46.2 ± 12.3 years; ratio men/women: 1.0:2.4) | not further stated | total cohort: 38%  cancer associated DM: 50%  DM without cancer: 35% |
| (Porkodi et al. 2002) | observational study (unclear if retrospective or prospective) | IIM, n=87  PM, n=24 (mean age: 33.3 years; men: 9)  DM, n=28 (mean age: 35.0 years; men: 11)  DM with malignancy, n=1 (42 years, woman)  JDM/JPM, n=5 (mean age: 7.4 years; men: 3)  overlap syndrome, n=30 (mean age: 25.5 years; men: 3) | not further stated | IIM: 33%  PM: 17%  DM: 54%  DM with malignancy: 0%  JDM/JPM: 0%  overlap syndrome: 33% |
| (Prasad et al. 2013) | retrospective observational study | JDM, n=18 (median age: 12.5 years; boys: 9) | not further stated | 22% |
| (Price et al. 2016) | retrospective, survey-based observational study among physicians | data based on 585 living and 149 deceased patients with sIBM | not further stated | 60% |
| (Raghu et al. 2015) | retrospective observational study | IIM, N=7 (mean age: 46 years; men: 2) | not further stated | 71% |
| (Rider et al. 2013) | retrospective observational study | JIIM with MSA/MAA-testing, n=374 (mean age at diagnosis: 7.4 years; boys: 103)  anti-synthetase-positive, n=19 (mean age at diagnosis: 14.0 years; boys: 3)  SRP-positive, n=6 (mean age at diagnosis: 15.1 years; boys: 2)  Mi2-positive, n=11 (mean age at diagnosis: 10.8 years; boys: 4)  P155/140 positive, n=131 (mean age at diagnosis: 7.2 years; boys: 29)  MJ-positive, n=86 (mean age at diagnosis: 6.2 years; boys: 26)  MSA/MAA positive, n=253 (mean age at diagnosis: 7.9 years; boys: 39) | not further stated | JIIM: 39%  anti-synthetase-positive: 16%  SRP-positive: 50%  Mi2-positive. 27%  P155/140 positive: 40%  NXP2-positive: 50%  MSA/MAA positive: 41% |
| (Rigolet et al. 2012) | retrospective observational study | anti-Ku-positive IIM, n=11 | not further stated | 36% |
| (Ringel et al. 1987) | retrospective observational study | IBM, n=19 (mean age  at diagnosis: 62.9 years; men: 15) | not further stated | 32% |
| (Rios 2005) | retrospective observational study | DM/PM/JDM, n=50 (mean age at onset: 30.0 ± 11.8 years; men: 24%) | not further stated | 10% at diagnosis |
| (Rodriguez Cruz et al. 2014) | prospective observational study | sIBM, n=16 | Dysphagia Outcome and Severity Scale (DOSS) ≤ 5, partly VFSS | 50% |
| (Rogers et al. 2017) | retrospective observational study | total cohort, n=178 (median age at diagnosis: 48.3 years; 28% men)  anti NXP-2-positive DM, n=20 (mean age at diagnosis: 51.7 ± 17.5 years; men: 10)  NXP-2-negative DM, n=158 (mean age at diagnosis: 47.3 ± 16.6 years; men: 39) | according to patient history, further severe dysphagia was defined as depending on feeding tube | total cohort: 42%  anti NXP-2-positive DM: 74%, 36% severe  anti NXP-2-negative DM: 39%, 10% severe  significantly higher in patients with NXP-2 positive DM |
| (Sarkar et al. 2017) | retrospective observational study | pediatric IIM, n=11 (mean age at diagnosis: 6.4 years; boys: 3) | not further stated | 36% |
| (Schrey et al. 2017) | retrospective observational study | sIBM, n=40 (mean age at diagnosis: 68.0 years; men: 25) | according to patient chart  partly VFSS and EGD | 63% |
| (Scola et al. 2000) | retrospective observational study | total cohort, n=102 (mean age 21 years men: 41)  definitive DM, n=34  mild-early DM, n=25  definitive PM, n=24  probable PM, n=19 | not further stated | total cohort: 30%  definitive DM: 44%  mild-early DM: 32%  definitive PM: 33%  probable PM: 0%  significantly more frequent in DM than in PM |
| (Selva-O'callaghan et al. 2006) | retrospective observational study | IIM, n=88  MSA positive IIM, n=28 (mean age at onset: 46 ± 19 years; men-women ratio: 1:2.5)  MAA positive IIM, n=43 (mean age at onset: 46.2 ± 17 years; men-women ratio: 1:1.8)  MSA and MAA positive IIM, n=12 (mean age at onset: 43.9 ± 17; men-women ratio: 1:3)  MSA or MAA positive, n=59 (mean age at onset: 46.5 ± 18 years; men-women ratio: 1:1.9)  MSA and MAA negative, n=29 (mean age at onset: 48.3 ± 17 years; men-women ratio: 1:3.1)  Jo-1-positive IIM, n=14 (mean age at onset: 44 ± 19 years; men-women ratio: 1:2.5)  Mi-2-positive IIM, n=6 (mean age at onset: 56 ± 24; men-women ratio: 1:5)  PM-Scl-positive IIM, n=10 (mean age at onset: 41 ± 15; men-women ratio: 3:2)  Ro-52-positive IIM, n=18 (mean age at onset: 49 ± 16 years; men-women ratio: 1:2)  Ro-60-positive IIM, n=19 (mean age at onset: 46 ± 17 years; men-women ratio: 8:2)  RNP-positive IIM, n=8 (mean age at onset: 49 ± 18 years; men-women ratio: 1:1) | not further stated | IIM: 36%  MSA positive IIM: 32%  MAA positive IIM: 42%  MSA and MAA positive IIM: 33%  MSA or MAA positive: 39%  MSA and MAA negative: 31%  Jo-1-positive IIM: 21%  Mi-2-positive IIM: 83%  PM-Scl-positive IIM: 50%  Ro-52-positive IIM: 50%  Ro-60-positive IIM: 50%  RNP-positive IIM: 37% |
| (Selva-O'callaghan et al. 2009) | prospective observational study | DM/PM, n=16 (mean age: 57.5 ± 14.5 years; men: 1) | clinical evidence, or findings from esophageal manometry, or upper gastrointestinal barium study | 37% |
| (Singh and Bansal 2006) | retrospective observational study | JDM, n=33 (mean age: 8.7±3.3 years, boys: 21) | clinical signs or tube-dependent | 15% |
| (So et al. 2011) | retrospective observational study | total cohort, n=151 (mean age: 49.5 ± 15.7 years; men: 59)  PM, n=53 (mean age: 53.8 ± 15.4 years; men: 19)  DM, n=98 (mean age: 47.1 ± 15.4; men: 40)  IIM with malignancy, n=25 (mean age: 62.4 ± 11.5; men: 14)  IIM without malignancy, n=126 (mean age: 46.9 ± 15.2; men: 45) | not further stated | total cohort: 26%  PM: 15%  DM: 33%  IIM with malignancy: 68%  IIM without malignancy: 18%  significantly higher in DM and in IIM with malignancy |
| (Suzuki et al. 2015) | retrospective observational study | anti-SRP-positive IIM, n=100 | not further stated | 41% |
| (Szabo et al. 2018) | retrospective observational study | ASS, n=49 (mean age at disease onset: 43.4 ± 13.28 years, men: 7) | not further stated | 12% |
| (Tarricone et al. 2012) | retrospective observational trial | SAE- positive DM, n=5 | not further stated | 0% |
| (Teh et al. 2012) | retrospective observational study | PM/DM, n=12 (mean age in years: 57.8 ± 11.1 years) | not further stated | 33% |
| (Uthman et al. 1996) | retrospective observational study | IIM in French Canadians (PM and DM), n=30 (median age at diagnosis: 44.5 years; men: 10) | not further stated  partly barium swallow | 43% |
| (Wang et al. 2018) | retrospective observational study | total cohort, n=120  NXP-2-postive IIM, n=10 (mean age: 50.5 ± 11.8 years; men: 3)  NXP-2-negative IIM, n=110 (mean age: 51.3 ± 14.0; men: 38) | not further stated | total cohort: 32%  NXP-2-postive IIM: 60%  NXP-2-negative IIM: 29% |
| (Wang et al. 1993) | prospective observational study | IIM, n=23 | increased esophageal transit time in scintigraphy | 74% |
| (Watanabe et al. 2015) | retrospective observational study | HMGCR-associated necrotizing autoimmune myositis, n=8 | not further stated | 0% |
| (Watanabe et al. 2016) | retrospective observational study | total cohort, n=113  anti-SRP-positive IIM without IBM, n=68 (mean age at examination: 55.2 ± 16.1 years, men: 19)  anti-HMGCR- positive IIM without IBM, n=45 (mean age at examination: 56.4 ± 18.8 years, men: 14) | not further stated | total cohort: 58%  anti-SRP-positive IIM without IBM: 68%  anti-HMGCR- positive IIM without IBM: 44%  significantly higher in anti-SRP-positive IIM without IBM |
| (Weitoft 1997) | retrospective observational study | IIM, n=21 (mean age: 54 years; men: 10) | not further stated | At onset of disease: 0% |
| (Williams et al. 2003) | prospective observational study | IIM with oropharyngeal dysphagia, n=13 | oropharyngeal dysphagia was defined as difficulty with the act of swallowing together with one or more of the following deglutitive symptoms: bolus holdup; multiple swallows required to clear the pharynx; deglutitive coughing and/or choking; or postnasal regurgitation  partly VFSS and manometry | general prevalence not applicable  69% dysphagia as presenting symptom |
| (Willig et al. 1994) | retrospective questionnaire-based study | DM and PM, n=43 (mean age: 43.2 ± 23.5 years; men: 3/12) | questionnaire assessing complaints associated with feeding, means of alimentation, orthodontic problems, and oral hygiene. | respondents reporting at least one alimentation problem during the chronic phase of the disease: 30% |
| (Wintzen et al. 1988) | Case-series | IBM, n=6 | partly VFSS | 67% |
| (Wu et al. 2013) | retrospective observational study | DM, n=230 (mean age: 51.97 ± 15.48 years; men: 91)  DM with intestinal lung disease, n=114  DM without intestinal lung disease, n=116 | not further stated | DM: 12%  DM with intestinal lung disease: 20%  DM without intestinal lung disease: 3%  significantly higher in patients with intestinal lung disease |
| (Yin et al. 2016) | retrospective observational study | IIM without IBM, n=104 | not further stated | 16% |
| (Yoo et al. 2019) | retrospective observational study | total cohort, n=121  PM, n=72 (mean age: 60.5 ± 16.0 years; men: 16)  DM, n=49 (mean age: 54.4 ± 15.0; men: 15) | not further stated | total cohort: 23%  PM: 25%  DM: 20% |
| (Zhou et al. 2019) | prospective observational study | total cohort, n=44  PM, n=11 (mean age: 40 years; men: 4)  DM, n=33 (mean age: 43 years; men: 5) | not further stated | total cohort: 16%  PM: 18%  DM: 15%  serum levels of sTRAIL in patients with dysphagia were significantly higher than that in patients without dysphagia |

Publication bibliography

Albayda, Jemima; Pinal-Fernandez, Iago; Huang, Wilson; Parks, Cassie; Paik, Julie; Casciola-Rosen, Livia et al. (2017): Antinuclear Matrix Protein 2 Autoantibodies and Edema, Muscle Disease, and Malignancy Risk in Dermatomyositis Patients. In *Arthritis care & research* 69 (11), pp. 1771–1776. DOI: 10.1002/acr.23188.

Albrecht, Inka; Wick, Cecilia; Hallgren, Asa; Tjarnlund, Anna; Nagaraju, Kanneboyina; Andrade, Felipe et al. (2015): Development of autoantibodies against muscle-specific FHL1 in severe inflammatory myopathies. In *The Journal of clinical investigation* 125 (12), pp. 4612–4624. DOI: 10.1172/JCI81031.

Allenbach, Yves; Drouot, Laurent; Rigolet, Aude; Charuel, Jean Luc; Jouen, Fabienne; Romero, Norma B. et al. (2014): Anti-HMGCR autoantibodies in European patients with autoimmune necrotizing myopathies: inconstant exposure to statin. In *Medicine* 93 (3), pp. 150–157. DOI: 10.1097/MD.0000000000000028.

Al-Mayouf, S.; Al-Mazyed, A.; Bahabri, S. (2000): Efficacy of early treatment of severe juvenile dermatomyositis with intravenous methylprednisolone and methotrexate. In *Clinical rheumatology* 19 (2), pp. 138–141. DOI: 10.1007/s100670050032.

Al-Mayouf, Sulaiman M.; AlMutiari, Nora; Muzaffer, Mohammed; Shehata, Rawiah; Al-Wahadneh, Adel; Abdwani, Reem et al. (2017): Phenotypic characteristics and outcome of juvenile dermatomyositis in Arab children. In *Rheumatology international* 37 (9), pp. 1513–1517. DOI: 10.1007/s00296-017-3770-x.

Azuma, Kohei; Yamada, Hidehiro; Ohkubo, Michiko; Yamasaki, Yoshioki; Yamasaki, Masaomi; Mizushima, Machiko; Ozaki, Shoichi (2011): Incidence and predictive factors for malignancies in 136 Japanese patients with dermatomyositis, polymyositis and clinically amyopathic dermatomyositis. In *Modern rheumatology* 21 (2), pp. 178–183. DOI: 10.1007/s10165-010-0362-y.

Badrising, Umesh A.; Maat-Schieman, Marion L. C.; van Houwelingen, Johannes C.; van Doorn, Peter A.; van Duinen, Sjoerd G.; van Engelen, Baziel G M et al. (2005): Inclusion body myositis. Clinical features and clinical course of the disease in 64 patients. In *Journal of neurology* 252 (12), pp. 1448–1454. DOI: 10.1007/s00415-005-0884-y.

Basnayake, Sajini K.; Blumbergs, Peter; Tan, Ju Ann; Roberts-Thompson, Peter J.; Limaye, Vidya (2015): Inflammatory myopathy with anti-SRP antibodies: case series of a South Australian cohort. In *Clinical rheumatology* 34 (3), pp. 603–608. DOI: 10.1007/s10067-014-2512-7.

Benbassat, J.; Gefel, D.; Larholt, K.; Sukenik, S.; Morgenstern, V.; Zlotnick, A. (1985): Prognostic factors in polymyositis/dermatomyositis. A computer-assisted analysis of ninety-two cases. In *Arthritis and rheumatism* 28 (3), pp. 249–255. DOI: 10.1002/art.1780280303.

Benveniste, Olivier; Guiguet, Marguerite; Freebody, Jane; Dubourg, Odile; Squier, Waney; Maisonobe, Thierry et al. (2011): Long-term observational study of sporadic inclusion body myositis. In *Brain : a journal of neurology* 134 (Pt 11), pp. 3176–3184. DOI: 10.1093/brain/awr213.

Betteridge, Z. E.; Gunawardena, H.; Chinoy, H.; North, J.; Ollier, W. E. R.; Cooper, R. G.; McHugh, N. J. (2009): Clinical and human leucocyte antigen class II haplotype associations of autoantibodies to small ubiquitin-like modifier enzyme, a dermatomyositis-specific autoantigen target, in UK Caucasian adult-onset myositis. In *Annals of the rheumatic diseases* 68 (10), pp. 1621–1625. DOI: 10.1136/ard.2008.097162.

Bodoki, Levente; Nagy-Vincze, Melinda; Griger, Zoltan; Betteridge, Zoe; Szollosi, Laszlone; Danko, Katalin (2014): Four dermatomyositis-specific autoantibodies-anti-TIF1gamma, anti-NXP2, anti-SAE and anti-MDA5-in adult and juvenile patients with idiopathic inflammatory myopathies in a Hungarian cohort. In *Autoimmunity reviews* 13 (12), pp. 1211–1219. DOI: 10.1016/j.autrev.2014.08.011.

Bucelli, Robert C.; Pestronk, Alan (2018): Immune myopathies with perimysial pathology: Clinical and laboratory features. In *Neurology(R) neuroimmunology & neuroinflammation* 5 (2), e434. DOI: 10.1212/NXI.0000000000000434.

Camargo, Leonardo Valente de; Carvalho, Mary Souza de; Shinjo, Samuel Katsuyuki; de Oliveira, Acary Souza Bulle; Zanoteli, Edmar (2018): Clinical, Histological, and Immunohistochemical Findings in Inclusion Body Myositis. In *BioMed research international* 2018, p. 5069042. DOI: 10.1155/2018/5069042.

Capkun, Gorana; Callan, Aoife; Tian, Haijun; Wei, Zhongyuan; Zhao, Changgeng; Agashivala, Neetu; Barghout, Victoria (2017): Burden of illness and healthcare resource use in United States patients with sporadic inclusion body myositis. In *Muscle & nerve* 56 (5), pp. 861–867. DOI: 10.1002/mus.25686.

Carpenter, J. R.; Bunch, T. W.; Engel, A. G.; O'Brien, P. C. (1977): Survival in polymyositis: corticosteroids and risk factors. In *The Journal of rheumatology* 4 (2), pp. 207–214.

Casal-Dominguez, Maria; Pinal-Fernandez, Iago; Mego, Marianela; Accarino, Anna; Jubany, Lluis; Azpiroz, Fernando; Selva-O'callaghan, Albert (2017): High-resolution manometry in patients with idiopathic inflammatory myopathy. Elevated prevalence of esophageal involvement and differences according to autoantibody status and clinical subset. In *Muscle & nerve* 56 (3), pp. 386–392. DOI: 10.1002/mus.25507.

Challa, Divya; Crowson, Cynthia S.; Niewold, Timothy B.; Reed, Ann M. (2018): Predictors of changes in disease activity among children with juvenile dermatomyositis enrolled in the Childhood Arthritis and Rheumatology Research Alliance (CARRA) Legacy Registry. In *Clinical rheumatology* 37 (4), pp. 1011–1015. DOI: 10.1007/s10067-017-3901-5.

Chen, Dongying; Yuan, Shiwen; Wu, Xiangni; Li, Hao; Qiu, Qian; Zhan, Zhongping et al. (2014): Incidence and predictive factors for malignancies with dermatomyositis: a cohort from southern China. In *Clinical and experimental rheumatology* 32 (5), pp. 615–621.

Cherin, Patrick; Delain, Jean-Christophe; Jaeger, Christophe de; Crave, Jean-Charles (2015): Subcutaneous Immunoglobulin Use in Inclusion Body Myositis: A Review of 6 Cases. In *Case reports in neurology* 7 (3), pp. 227–232. DOI: 10.1159/000441490.

Chickermane, Pranav R.; Mankad, Deepali; Khubchandani, Raju P. (2013): Disease patterns of juvenile dermatomyositis from Western India. In *Indian pediatrics* 50 (10), pp. 961–963. DOI: 10.1007/s13312-013-0260-4.

Chiu, Shih Kai; Yang, Yao Hsu; Wang, Li Chieh; Chiang, Bor Luen (2007): Ten-year experience of juvenile dermatomyositis: a retrospective study. In *Journal of microbiology, immunology, and infection = Wei mian yu gan ran za zhi* 40 (1), pp. 68–73.

Chung, H. T.; Huang, J. L.; Wang, H. S.; Hung, P. C.; Chou, M. L. (1994): Dermatomyositis and polymyositis in childhood. In *Zhonghua Minguo xiao er ke yi xue hui za zhi [Journal]. Zhonghua Minguo xiao er ke yi xue hui* 35 (5), pp. 407–414.

Chwalinska-Sadowska, H.; Maldykowa, H. (1990): Polymyositis-dermatomyositis--a 25-year follow-up of 50 patients (analysis of clinical symptoms and signs and results of laboratory tests). In *Materia medica Polona. Polish journal of medicine and pharmacy* 22 (3), pp. 205–212.

Cox, F. M.; Verschuuren, J. J.; Verbist, B. M.; Niks, E. H.; Wintzen, A. R.; Badrising, U. A. (2009): Detecting dysphagia in inclusion body myositis. In *Journal of neurology* 256 (12), pp. 2009–2013. DOI: 10.1007/s00415-009-5229-9.

Cunningham, J. D., JR; Lowry, L. D. (1985): Head and neck manifestations of dermatomyositis-polymyositis. In *Otolaryngology--head and neck surgery : official journal of American Academy of Otolaryngology-Head and Neck Surgery* 93 (5), pp. 673–677.

Dabby, R.; Lange, D. J.; Trojaborg, W.; Hays, A. P.; Lovelace, R. E.; Brannagan, T. H.; Rowland, L. P. (2001): Inclusion body myositis mimicking motor neuron disease. In *Archives of neurology* 58 (8), pp. 1253–1256. DOI: 10.1001/archneur.58.8.1253.

Danieli, Maria Giovanna; Calcabrini, Lucia; Calabrese, Vincenzina; Marchetti, Annalisa; Logullo, Francesco; Gabrielli, Armando (2009): Intravenous immunoglobulin as add on treatment with mycophenolate mofetil in severe myositis. In *Autoimmunity reviews* 9 (2), pp. 124–127. DOI: 10.1016/j.autrev.2009.04.003.

Danko, Katalin; Ponyi, Andrea; Constantin, Tamas; Borgulya, Gabor; Szegedi, Gyula (2004): Long-term survival of patients with idiopathic inflammatory myopathies according to clinical features: a longitudinal study of 162 cases. In *Medicine* 83 (1), pp. 35–42. DOI: 10.1097/01.md.0000109755.65914.5e.

Davis, M. D.; Ahmed, I. (1997): Ovarian malignancy in patients with dermatomyositis and polymyositis: a retrospective analysis of fourteen cases. In *Journal of the American Academy of Dermatology* 37 (5 Pt 1), pp. 730–733. DOI: 10.1016/s0190-9622(97)70109-9.

de Souza, Fernando Henrique Carlos; Miossi, Renata; de Moraes, Julio Cesar Bertacini; Bonfa, Eloisa; Shinjo, Samuel Katsuyuki (2018): Favorable rituximab response in patients with refractory idiopathic inflammatory myopathies. In *Advances in rheumatology (London, England)* 58 (1), p. 31. DOI: 10.1186/s42358-018-0030-z.

de Souza, Fernando Henrique Carlos; Miossi, Renata; Shinjo, Samuel Katsuyuki (2017): Necrotising myopathy associated with anti-signal recognition particle (anti-SRP) antibody. In *Clinical and experimental rheumatology* 35 (5), pp. 766–771.

Degos, R.; Civatte, J.; Belaich, S.; Delarue, A. (1971): The prognosis of adult dermatomyositis. In *Transactions of the St. John's Hospital Dermatological Society* 57 (1), pp. 98–104.

Della Marca, Giacomo; Sancricca, Cristina; Losurdo, Anna; Di Blasi, Chiara; Fino, Chiara de; Morosetti, Roberta et al. (2013): Sleep disordered breathing in a cohort of patients with sporadic inclusion body myositis. In *Clinical neurophysiology : official journal of the International Federation of Clinical Neurophysiology* 124 (8), pp. 1615–1621. DOI: 10.1016/j.clinph.2013.03.002.

Dobloug, Cecilie; Garen, Torhild; Bitter, Helle; Stjarne, Johan; Stenseth, Guri; Grovle, Lars et al. (2015a): Prevalence and clinical characteristics of adult polymyositis and dermatomyositis; data from a large and unselected Norwegian cohort. In *Annals of the rheumatic diseases* 74 (8), pp. 1551–1556. DOI: 10.1136/annrheumdis-2013-205127.

Dobloug, Cecilie; Walle-Hansen, Ragnhild; Gran, Jan Tore; Molberg, Oyvind (2012): Long-term follow-up of sporadic inclusion body myositis treated with intravenous immunoglobulin: a retrospective study of 16 patients. In *Clinical and experimental rheumatology* 30 (6), pp. 838–842.

Dobloug, G. C.; Antal, E. A.; Sveberg, L.; Garen, T.; Bitter, H.; Stjarne, J. et al. (2015b): High prevalence of inclusion body myositis in Norway; a population-based clinical epidemiology study. In *European journal of neurology* 22 (4), 672-e41. DOI: 10.1111/ene.12627.

DONOGHUE, F. E.; WINKELMANN, R. K.; MOERSCH, H. J. (1960): Esophageal defects in dermatomyositis. In *The Annals of otology, rhinology, and laryngology* 69, pp. 1139–1145. DOI: 10.1177/000348946006900422.

Dos Passos Carvalho, Maria Isabel Cardoso; Shinjo, Samuel Katsuyuki (2019): Frequency and clinical relevance of anti-Mi-2 autoantibody in adult Brazilian patients with dermatomyositis. In *Advances in rheumatology (London, England)* 59 (1), p. 27. DOI: 10.1186/s42358-019-0071-y.

Dunlap, Heather V.; Macneil, Lauren G.; Tarnopolsky, Mark A. (2014): Functional impairment in patients with sporadic Inclusion Body Myositis. In *The Canadian journal of neurological sciences. Le journal canadien des sciences neurologiques* 41 (2), pp. 253–259. DOI: 10.1017/s0317167100016668.

Ertekin, C.; Aydogdu, I.; Yuceyar, N. (1996): Piecemeal deglutition and dysphagia limit in normal subjects and in patients with swallowing disorders. In *Journal of neurology, neurosurgery, and psychiatry* 61 (5), pp. 491–496. DOI: 10.1136/jnnp.61.5.491.

Ertekin, Cumhur; Secil, Yaprak; Yuceyar, Nur; Aydogdu, Ibrahim (2004): Oropharyngeal dysphagia in polymyositis/dermatomyositis. In *Clinical neurology and neurosurgery* 107 (1), pp. 32–37. DOI: 10.1016/j.clineuro.2004.02.024.

Fang, Yao-Fan; Wu, Yeong-Jian Jan; Kuo, Chang-Fu; Luo, Shue-Fen; Yu, Kuang-Hui (2016): Malignancy in dermatomyositis and polymyositis: analysis of 192 patients. In *Clinical rheumatology* 35 (8), pp. 1977–1984. DOI: 10.1007/s10067-016-3296-8.

Felice, K. J.; North, W. A. (2001): Inclusion body myositis in Connecticut: observations in 35 patients during an 8-year period. In *Medicine* 80 (5), pp. 320–327. DOI: 10.1097/00005792-200109000-00006.

Fujimoto, Manabu; Matsushita, Takashi; Hamaguchi, Yasuhito; Kaji, Kenzo; Asano, Yoshihide; Ogawa, Fumihide et al. (2013): Autoantibodies to small ubiquitin-like modifier activating enzymes in Japanese patients with dermatomyositis: comparison with a UK Caucasian cohort. In *Annals of the rheumatic diseases* 72 (1), pp. 151–153. DOI: 10.1136/annrheumdis-2012-201736.

Galindo-Feria, Angeles Shunashy; Rojas-Serrano, Jorge; Hinojosa-Azaola, Andrea (2016): Clinical and Prognostic Factors Associated With Survival in Mexican Patients With Idiopathic Inflammatory Myopathies. In *Journal of clinical rheumatology : practical reports on rheumatic & musculoskeletal diseases* 22 (2), pp. 51–56. DOI: 10.1097/RHU.0000000000000365.

Gao, Xiang; Han, Lei; Yuan, Lan; Yang, Yongchen; Gou, Guimei; Sun, Hengjuan et al. (2014): HLA class II alleles may influence susceptibility to adult dermatomyositis and polymyositis in a Han Chinese population. In *BMC dermatology* 14, p. 9. DOI: 10.1186/1471-5945-14-9.

Ge, Yongpeng; Lu, Xin; Peng, Qinglin; Shu, Xiaoming; Wang, Guochun (2015): Clinical Characteristics of Anti-3-Hydroxy-3-Methylglutaryl Coenzyme A Reductase Antibodies in Chinese Patients with Idiopathic Inflammatory Myopathies. In *PloS one* 10 (10), e0141616. DOI: 10.1371/journal.pone.0141616.

Ge, Yongpeng; Lu, Xin; Shu, Xiaoming; Peng, Qinglin; Wang, Guochun (2017): Clinical characteristics of anti-SAE antibodies in Chinese patients with dermatomyositis in comparison with different patient cohorts. In *Scientific reports* 7 (1), p. 188. DOI: 10.1038/s41598-017-00240-6.

Goyal, N. A.; Cash, T. M.; Alam, U.; Enam, S.; Tierney, P.; Araujo, N. et al. (2016): Seropositivity for NT5c1A antibody in sporadic inclusion body myositis predicts more severe motor, bulbar and respiratory involvement. In *Journal of neurology, neurosurgery, and psychiatry* 87 (4), pp. 373–378. DOI: 10.1136/jnnp-2014-310008.

Gupta, Rajiv; Gow, Peter J. (2013): Inflammatory myopathies--a review of newly diagnosed patients (2004-2008) in the Counties Manukau region. In *The New Zealand medical journal* 126 (1370), pp. 89–95.

Hajialilo, Mehrzad; Ghorbanihaghjo, Amir; Khabbazi, Alireza; Kolahi, Sousan; Jafari Nakhjavani, Mohammad Reza; Ebrahimi, Ali Asghar et al. (2018): Long-term follow-up of 76 Iranian patients with idiopathic inflammatory myopathies. In *International journal of rheumatic diseases* 21 (8), pp. 1627–1633. DOI: 10.1111/1756-185X.13352.

Hengstman, G. J. D.; ter Laak, H. J.; Vree Egberts, W T M; Lundberg, I. E.; Moutsopoulos, H. M.; Vencovsky, J. et al. (2006): Anti-signal recognition particle autoantibodies: marker of a necrotising myopathy. In *Annals of the rheumatic diseases* 65 (12), pp. 1635–1638. DOI: 10.1136/ard.2006.052191.

Hochberg, M. C.; Feldman, D.; STEVENS, M. B. (1986): Adult onset polymyositis/dermatomyositis: an analysis of clinical and laboratory features and survival in 76 patients with a review of the literature. In *Seminars in arthritis and rheumatism* 15 (3), pp. 168–178. DOI: 10.1016/0049-0172(86)90014-4.

Hoesly, Paul M.; Sluzevich, Jason C.; Jambusaria-Pahlajani, Anokhi; Lesser, Elizabeth R.; Heckman, Michael G.; Abril, Andy (2019): Association of antinuclear antibody status with clinical features and malignancy risk in adult-onset dermatomyositis. In *Journal of the American Academy of Dermatology* 80 (5), pp. 1364–1370. DOI: 10.1016/j.jaad.2018.11.023.

Horowitz, M.; McNeil, J. D.; Maddern, G. J.; Collins, P. J.; Shearman, D. J. (1986): Abnormalities of gastric and esophageal emptying in polymyositis and dermatomyositis. In *Gastroenterology* 90 (2), pp. 434–439. DOI: 10.1016/0016-5085(86)90944-3.

Houser, S. M.; Calabrese, L. H.; Strome, M. (1998): Dysphagia in patients with inclusion body myositis. In *The Laryngoscope* 108 (7), pp. 1001–1005. DOI: 10.1097/00005537-199807000-00009.

Hu, Chaojun; Wu, Chanyuan; Yang, Enhao; Huang, Hui; Xu, Dong; Hou, Yong et al. (2019): Serum KL-6 is associated with the severity of interstitial lung disease in Chinese patients with polymyositis and dermatomyositis. In *Clinical rheumatology* 38 (8), pp. 2181–2187. DOI: 10.1007/s10067-019-04501-9.

Jacob, H.; Berkowitz, D.; McDonald, E.; Bernstein, L. H.; Beneventano, T. (1983): The esophageal motility disorder of polymyositis. A prospective study. In *Archives of internal medicine* 143 (12), pp. 2262–2264.

Johnson, Sindhu R.; Fransen, Jaap; Khanna, Dinesh; Baron, Murray; van den Hoogen, Frank; Medsger, Thomas A., JR et al. (2012): Validation of potential classification criteria for systemic sclerosis. In *Arthritis care & research* 64 (3), pp. 358–367. DOI: 10.1002/acr.20684.

Kang, E. H.; Lee, E. B.; Shin, K. C.; Im, C. H.; Chung, D. H.; Han, S. K.; Song, Y. W. (2005): Interstitial lung disease in patients with polymyositis, dermatomyositis and amyopathic dermatomyositis. In *Rheumatology (Oxford, England)* 44 (10), pp. 1282–1286. DOI: 10.1093/rheumatology/keh723.

Kang, Eun Ha; Lee, Sang Jin; Ascherman, Dana P.; Lee, Yun Jong; Lee, Eun Young; Lee, Eun Bong; Song, Yeong Wook (2016): Temporal relationship between cancer and myositis identifies two distinctive subgroups of cancers: impact on cancer risk and survival in patients with myositis. In *Rheumatology (Oxford, England)* 55 (9), pp. 1631–1641. DOI: 10.1093/rheumatology/kew215.

Karri, Sudhir Babu; Kannan, Meena Anga Muthu; Rajashekhar, Liza; Uppin, Megha S.; Challa, Sundaram (2015): Clinico pathological study of adult dermatomyositis: Importance of muscle histology in the diagnosis. In *Annals of Indian Academy of Neurology* 18 (2), pp. 194–199. DOI: 10.4103/0972-2327.150603.

Kim, Na Ri; Nam, Eon Jeong; Kang, Jong Wan; Song, Hyun Seok; Im, Churl Hyun; Kang, Young Mo (2014): Complex repetitive discharge on electromyography as a risk factor for malignancy in idiopathic inflammatory myopathy. In *The Korean journal of internal medicine* 29 (6), pp. 814–821. DOI: 10.3904/kjim.2014.29.6.814.

Kim, S. J.; Han, T. R.; Jeong, S. J.; Beom, J. W. (2010): Comparison between swallowing-related and limb muscle involvement in dermatomyositis patients. In *Scandinavian journal of rheumatology* 39 (4), pp. 336–340. DOI: 10.3109/03009740903555366.

Lakhanpal, S.; Lie, J. T.; Conn, D. L.; Martin, W. J. 2nd (1987): Pulmonary disease in polymyositis/dermatomyositis: a clinicopathological analysis of 65 autopsy cases. In *Annals of the rheumatic diseases* 46 (1), pp. 23–29. DOI: 10.1136/ard.46.1.23.

Langdon, P. Claire; Mulcahy, Kylie; Shepherd, Kelly L.; Low, Vincent H.; Mastaglia, Frank L. (2012): Pharyngeal dysphagia in inflammatory muscle diseases resulting from impaired suprahyoid musculature. In *Dysphagia* 27 (3), pp. 408–417. DOI: 10.1007/s00455-011-9384-7.

Li, Shanshan; Ge, Yongpeng; Yang, Hanbo; Wang, Tao; Zheng, Xiaoxiao; Peng, Qinglin et al. (2019): The spectrum and clinical significance of myositis-specific autoantibodies in Chinese patients with idiopathic inflammatory myopathies. In *Clinical rheumatology* 38 (8), pp. 2171–2179. DOI: 10.1007/s10067-019-04503-7.

Lilleker, James B.; Vencovsky, Jiri; Wang, Guochun; Wedderburn, Lucy R.; Diederichsen, Louise Pyndt; Schmidt, Jens et al. (2018): The EuroMyositis registry: an international collaborative tool to facilitate myositis research. In *Annals of the rheumatic diseases* 77 (1), pp. 30–39. DOI: 10.1136/annrheumdis-2017-211868.

Lindberg, C.; Persson, L. I.; Bjorkander, J.; Oldfors, A. (1994): Inclusion body myositis: clinical, morphological, physiological and laboratory findings in 18 cases. In *Acta neurologica Scandinavica* 89 (2), pp. 123–131. DOI: 10.1111/j.1600-0404.1994.tb01647.x.

Liu, Y.; Zheng, Y.; Gang, Q.; Xie, Z.; Jin, Y.; Zhang, X. et al. (2019): Perimysial microarteriopathy in dermatomyositis with anti-nuclear matrix protein-2 antibodies. In *European journal of neurology*. DOI: 10.1111/ene.14097.

Lotz, B. P.; Engel, A. G.; Nishino, H.; Stevens, J. C.; Litchy, W. J. (1989): Inclusion body myositis. Observations in 40 patients. In *Brain : a journal of neurology* 112 (Pt 3), pp. 727–747. DOI: 10.1093/brain/112.3.727.

Lynn, S. J.; Sawyers, S. M.; Moller, P. W.; O'Donnell, J. L.; Chapman, P. T. (2005): Adult-onset inflammatory myopathy. North Canterbury experience 1989-2001. In *Internal medicine journal* 35 (3), pp. 170–173. DOI: 10.1111/j.1445-5994.2004.00764.x.

Mamyrova, Gulnara; Kishi, Takayuki; Targoff, Ira N.; Ehrlich, Alison; Curiel, Rodolfo V.; Rider, Lisa G. (2018): Features distinguishing clinically amyopathic juvenile dermatomyositis from juvenile dermatomyositis. In *Rheumatology (Oxford, England)* 57 (11), pp. 1956–1963. DOI: 10.1093/rheumatology/key190.

Marie, I.; Hachulla, E.; Cherin, P.; Dominique, S.; Hatron, P-Y; Hellot, M-F et al. (2002): Interstitial lung disease in polymyositis and dermatomyositis. In *Arthritis and rheumatism* 47 (6), pp. 614–622. DOI: 10.1002/art.10794.

Marie, I.; Hachulla, E.; Hatron, P. Y.; Hellot, M. F.; Levesque, H.; Devulder, B.; Courtois, H. (2001): Polymyositis and dermatomyositis: short term and longterm outcome, and predictive factors of prognosis. In *The Journal of rheumatology* 28 (10), pp. 2230–2237.

Marie, I.; Hatron, P. Y.; Levesque, H.; Hachulla, E.; Hellot, M. F.; Michon-Pasturel, U. et al. (1999): Influence of age on characteristics of polymyositis and dermatomyositis in adults. In *Medicine* 78 (3), pp. 139–147. DOI: 10.1097/00005792-199905000-00001.

Marie, I.; Menard, J-F; Hatron, P. Y.; Hachulla, E.; Mouthon, L.; Tiev, K. et al. (2010): Intravenous immunoglobulins for steroid-refractory esophageal involvement related to polymyositis and dermatomyositis: a series of 73 patients. In *Arthritis care & research* 62 (12), pp. 1748–1755. DOI: 10.1002/acr.20325.

Maugars, Y. M.; Berthelot, J. M.; Abbas, A. A.; Mussini, J. M.; Nguyen, J. M.; Prost, A. M. (1996): Long-term prognosis of 69 patients with dermatomyositis or polymyositis. In *Clinical and experimental rheumatology* 14 (3), pp. 263–274.

McCann, L. J.; Garay, S. M.; Ryan, M. M.; Harris, R.; Riley, P.; Pilkington, C. A. (2007): Oropharyngeal dysphagia in juvenile dermatomyositis (JDM): an evaluation of videofluoroscopy swallow study (VFSS) changes in relation to clinical symptoms and objective muscle scores. In *Rheumatology (Oxford, England)* 46 (8), pp. 1363–1366. DOI: 10.1093/rheumatology/kem131.

Medsger, T. A., JR; Robinson, H.; Masi, A. T. (1971): Factors affecting survivorship in polymyositis. A life-table study of 124 patients. In *Arthritis and rheumatism* 14 (2), pp. 249–258. DOI: 10.1002/art.1780140210.

Merieux, P. de; Verity, M. A.; Clements, P. J.; Paulus, H. E. (1983): Esophageal abnormalities and dysphagia in polymyositis and dermatomyositis. In *Arthritis and rheumatism* 26 (8), pp. 961–968. DOI: 10.1002/art.1780260804.

Milisenda, Jose C.; Doti, Pamela I.; Prieto-Gonzalez, Sergio; Grau, Josep M. (2014): Dermatomyositis presenting with severe subcutaneous edema: five additional cases and review of the literature. In *Seminars in arthritis and rheumatism* 44 (2), pp. 228–233. DOI: 10.1016/j.semarthrit.2014.04.004.

Moghadam-Kia, Siamak; Oddis, Chester V.; Sato, Shinji; Kuwana, Masataka; Aggarwal, Rohit (2017): Antimelanoma Differentiation-associated Gene 5 Antibody: Expanding the Clinical Spectrum in North American Patients with Dermatomyositis. In *The Journal of rheumatology* 44 (3), pp. 319–325. DOI: 10.3899/jrheum.160682.

Mugii, Naoki; Hasegawa, Minoru; Matsushita, Takashi; Hamaguchi, Yasuhito; Oohata, Sacihe; Okita, Hirokazu et al. (2016): Oropharyngeal Dysphagia in Dermatomyositis: Associations with Clinical and Laboratory Features Including Autoantibodies. In *PloS one* 11 (5), e0154746. DOI: 10.1371/journal.pone.0154746.

Mulcahy, Kylie Patricia; Langdon, Patricia Claire; Mastaglia, Francis (2012): Dysphagia in inflammatory myopathy. Self-report, incidence, and prevalence. In *Dysphagia* 27 (1), pp. 64–69. DOI: 10.1007/s00455-011-9338-0.

Murata, Ken-Ya; Kouda, Ken; Tajima, Fumihiro; Kondo, Tomoyoshi (2012): A dysphagia study in patients with sporadic inclusion body myositis (s-IBM). In *Neurological sciences : official journal of the Italian Neurological Society and of the Italian Society of Clinical Neurophysiology* 33 (4), pp. 765–770. DOI: 10.1007/s10072-011-0814-y.

Muro, Yoshinao; Sugiura, Kazumitsu; Nara, Mizuho; Sakamoto, Izumi; Suzuki, Noriyuki; Akiyama, Masashi (2015): High incidence of cancer in anti-small ubiquitin-like modifier activating enzyme antibody-positive dermatomyositis. In *Rheumatology (Oxford, England)* 54 (9), pp. 1745–1747. DOI: 10.1093/rheumatology/kev247.

Mustafa, Khader N.; Dahbour, Said S. (2010): Clinical characteristics and outcomes of patients with idiopathic inflammatory myopathies from Jordan 1996-2009. In *Clinical rheumatology* 29 (12), pp. 1381–1385. DOI: 10.1007/s10067-010-1465-8.

Na, Sang-Jun; Kim, Seung Min; Sunwoo, Il Nam; Choi, Young-Chul (2009): Clinical characteristics and outcomes of juvenile and adult dermatomyositis. In *Journal of Korean medical science* 24 (4), pp. 715–721. DOI: 10.3346/jkms.2009.24.4.715.

Narayanaswamy, A. S.; Akhtar, M.; Kumar, N.; Lazar, A. I. (1993): Polymyositis--a review and follow up study of 24 cases. In *The Journal of the Association of Physicians of India* 41 (6), pp. 354–356.

Needham, M.; James, I.; Corbett, A.; Day, T.; Christiansen, F.; Phillips, B.; Mastaglia, F. L. (2008): Sporadic inclusion body myositis: phenotypic variability and influence of HLA-DR3 in a cohort of 57 Australian cases. In *Journal of neurology, neurosurgery, and psychiatry* 79 (9), pp. 1056–1060. DOI: 10.1136/jnnp.2007.138891.

Neri, Rossella; Barsotti, Simone; Iacopetti, Valentina; Iacopetti, Giacomo; Pepe, Pasquale; d'Ascanio, Anna et al. (2014): Cancer-associated myositis: a 35-year retrospective study of a monocentric cohort. In *Rheumatology international* 34 (4), pp. 565–569. DOI: 10.1007/s00296-013-2910-1.

Noguchi, Eri; Uruha, Akinori; Suzuki, Shigeaki; Hamanaka, Kohei; Ohnuki, Yuko; Tsugawa, Jun et al. (2017): Skeletal Muscle Involvement in Antisynthetase Syndrome. In *JAMA neurology* 74 (8), pp. 992–999. DOI: 10.1001/jamaneurol.2017.0934.

Nuno-Nuno, Laura; Joven, Beatriz Esther; Carreira, Patricia E.; Maldonado-Romero, Valentina; Larena-Grijalba, Carmen; Llorente Cubas, Irene et al. (2019): Overlap myositis, a distinct entity beyond primary inflammatory myositis: A retrospective analysis of a large cohort from the REMICAM registry. In *International journal of rheumatic diseases* 22 (8), pp. 1393–1401. DOI: 10.1111/1756-185X.13559.

Ogawa-Momohara, Mariko; Muro, Yoshinao; Kono, Michihiro; Akiyama, Masashi (2019): Prognosis of dysphagia in dermatomyositis. In *Clinical and experimental rheumatology* 37 (1), p. 165.

Oh, Terry H.; Brumfield, Kathlyn A.; Hoskin, Tanya L.; Kasperbauer, Jan L.; Basford, Jeffrey R. (2008): Dysphagia in inclusion body myositis: clinical features, management, and clinical outcome. In *American journal of physical medicine & rehabilitation* 87 (11), pp. 883–889. DOI: 10.1097/PHM.0b013e31818a50e2.

Oh, Terry H.; Brumfield, Kathlyn A.; Hoskin, Tanya L.; Stolp, Kathryn A.; Murray, Joseph A.; Bassford, Jeffrey R. (2007): Dysphagia in inflammatory myopathy. Clinical characteristics, treatment strategies, and outcome in 62 patients. In *Mayo Clinic proceedings* 82 (4), pp. 441–447.

Okada, Yoshifumi; Jinnin, Masatoshi; Makino, Takamitsu; Kajihara, Ikko; Makino, Katsunari; Honda, Noritoshi et al. (2014): MIRSNP rs2910164 of miR-146a is associated with the muscle involvement in polymyositis/dermatomyositis. In *International journal of dermatology* 53 (3), pp. 300–304. DOI: 10.1111/j.1365-4632.2012.05739.x.

Olthoff, Arno; Carstens, Per-Ole; Zhang, Shuo; Fintel, Eva von; Friede, Tim; Lotz, Joachim et al. (2016): Evaluation of dysphagia by novel real-time MRI. In *Neurology* 87 (20), pp. 2132–2138. DOI: 10.1212/WNL.0000000000003337.

Ozonoff, M. B.; Flynn, F. J., JR (1973): Roentgenologic features of dermatomyositis of childhood. In *The American journal of roentgenology, radium therapy, and nuclear medicine* 118 (1), pp. 206–212. DOI: 10.2214/ajr.118.1.206.

Pachman, L. M.; Hayford, J. R.; Chung, A.; Daugherty, C. A.; Pallansch, M. A.; Fink, C. W. et al. (1998): Juvenile dermatomyositis at diagnosis: clinical characteristics of 79 children. In *The Journal of rheumatology* 25 (6), pp. 1198–1204.

Peng, A.; Koffman, B. M.; Malley, J. D.; Dalakas, M. C. (2000): Disease progression in sporadic inclusion body myositis: observations in 78 patients. In *Neurology* 55 (2), pp. 296–298. DOI: 10.1212/wnl.55.2.296.

Peng, Qing-Lin; Shu, Xiao-Ming; Tian, Xiao-Lan; Lu, Xin; Wang, Guo-Chun (2014): Expression of tumor necrosis factor-like weak inducer of apoptosis and fibroblast growth factor-inducible 14 in patients with polymyositis and dermatomyositis. In *Arthritis research & therapy* 16 (1), R26. DOI: 10.1186/ar4454.

Ponyi, Andrea; Constantin, Tamas; Garami, Miklos; Andras, Csilla; Tallai, Bela; Vancsa, Andrea et al. (2005): Cancer-associated myositis: clinical features and prognostic signs. In *Annals of the New York Academy of Sciences* 1051, pp. 64–71. DOI: 10.1196/annals.1361.047.

Porkodi, R.; Shanmuganandan, K.; Parthiban, M.; Madhavan, Radha; Rajendran, P. (2002): Clinical spectrum of inflammatory myositis in South India--a ten year study. In *The Journal of the Association of Physicians of India* 50, pp. 1255–1258.

Prasad, Shiva; Misra, Ramnath; Agarwal, Vikas; Lawrence, Able; Aggarwal, Amita (2013): Juvenile dermatomyositis at a tertiary care hospital: is there any change in the last decade? In *International journal of rheumatic diseases* 16 (5), pp. 556–560. DOI: 10.1111/1756-185X.12053.

Price, Mark A.; Barghout, Victoria; Benveniste, Olivier; Christopher-Stine, Lisa; Corbett, Alastair; Visser, Marianne de et al. (2016): Mortality and Causes of Death in Patients with Sporadic Inclusion Body Myositis: Survey Study Based on the Clinical Experience of Specialists in Australia, Europe and the USA. In *Journal of neuromuscular diseases* 3 (1), pp. 67–75. DOI: 10.3233/JND-150138.

Raghu, Padmanabhan; Manadan, Augustine M.; Schmukler, Juan; Mathur, Tanisha; Block, Joel A. (2015): Pulse Dose Methylprednisolone Therapy for Adult Idiopathic Inflammatory Myopathy. In *American journal of therapeutics* 22 (4), pp. 244–247. DOI: 10.1097/MJT.0000000000000175.

Rider, Lisa G.; Shah, Mona; Mamyrova, Gulnara; Huber, Adam M.; Rice, Madeline Murguia; Targoff, Ira N.; Miller, Frederick W. (2013): The myositis autoantibody phenotypes of the juvenile idiopathic inflammatory myopathies. In *Medicine* 92 (4), pp. 223–243. DOI: 10.1097/MD.0b013e31829d08f9.

Rigolet, Aude; Musset, Lucile; Dubourg, Odile; Maisonobe, Thierry; Grenier, Philippe; Charuel, Jean-Luc et al. (2012): Inflammatory myopathies with anti-Ku antibodies: a prognosis dependent on associated lung disease. In *Medicine* 91 (2), pp. 95–102. DOI: 10.1097/MD.0b013e31824d9cec.

Ringel, S. P.; Kenny, C. E.; Neville, H. E.; Giorno, R.; Carry, M. R. (1987): Spectrum of inclusion body myositis. In *Archives of neurology* 44 (11), pp. 1154–1157. DOI: 10.1001/archneur.1987.00520230042011.

Rios, Grissel (2005): Retrospective review of the clinical manifestations and outcomes in Puerto Ricans with idiopathic inflammatory myopathies. In *Journal of clinical rheumatology : practical reports on rheumatic & musculoskeletal diseases* 11 (3), pp. 153–156. DOI: 10.1097/01.rhu.0000164820.46979.52.

Rodriguez Cruz, Pedro M.; Needham, Merrilee; Hollingsworth, Peter; Mastaglia, Frank L.; Hillman, David R. (2014): Sleep disordered breathing and subclinical impairment of respiratory function are common in sporadic inclusion body myositis. In *Neuromuscular disorders : NMD* 24 (12), pp. 1036–1041. DOI: 10.1016/j.nmd.2014.08.003.

Rogers, Anna; Chung, Lorinda; Li, Shufeng; Casciola-Rosen, Livia; Fiorentino, David F. (2017): Cutaneous and Systemic Findings Associated With Nuclear Matrix Protein 2 Antibodies in Adult Dermatomyositis Patients. In *Arthritis care & research* 69 (12), pp. 1909–1914. DOI: 10.1002/acr.23210.

Sarkar, Sumantra; Mondal, Tanushree; Saha, Arpan; Mondal, Rakesh; Datta, Supratim (2017): Profile of Pediatric Idiopathic Inflammatory Myopathies from a Tertiary Care Center of Eastern India. In *Indian journal of pediatrics* 84 (4), pp. 299–306. DOI: 10.1007/s12098-017-2302-8.

Schrey, Aleksi; Airas, Laura; Jokela, Manu; Pulkkinen, Jaakko (2017): Botulinum toxin alleviates dysphagia of patients with inclusion body myositis. In *Journal of the neurological sciences* 380, pp. 142–147. DOI: 10.1016/j.jns.2017.07.031.

Scola, R. H.; Werneck, L. C.; Prevedello, D. M.; Toderke, E. L.; Iwamoto, F. M. (2000): Diagnosis of dermatomyositis and polymyositis: a study of 102 cases. In *Arquivos de neuro-psiquiatria* 58 (3B), pp. 789–799. DOI: 10.1590/s0004-282x2000000500001.

Selva-O'callaghan, Albert; Labrador-Horrillo, Moises; Solans-Laque, Roser; Simeon-Aznar, Carmen Pilar; Martinez-Gomez, Xavier; Vilardell-Tarres, Miquel (2006): Myositis-specific and myositis-associated antibodies in a series of eighty-eight Mediterranean patients with idiopathic inflammatory myopathy. In *Arthritis and rheumatism* 55 (5), pp. 791–798. DOI: 10.1002/art.22237.

Selva-O'callaghan, Albert; Sampol, Gabriel; Romero, Odile; Lloberes, Patricia; Trallero-Araguas, Ernesto; Vilardell-Tarres, Miquel (2009): Obstructive sleep apnea in patients with inflammatory myopathies. In *Muscle & nerve* 39 (2), pp. 144–149. DOI: 10.1002/mus.21204.

Singh, Surjit; Bansal, Arun (2006): Twelve years experience of juvenile dermatomyositis in North India. In *Rheumatology international* 26 (6), pp. 510–515. DOI: 10.1007/s00296-005-0030-2.

So, Min Wook; Koo, Bon San; Kim, Yong-Gil; Lee, Chang-Keun; Yoo, Bin (2011): Idiopathic inflammatory myopathy associated with malignancy: a retrospective cohort of 151 Korean patients with dermatomyositis and polymyositis. In *The Journal of rheumatology* 38 (11), pp. 2432–2435. DOI: 10.3899/jrheum.110320.

Suzuki, Shigeaki; Nishikawa, Atsuko; Kuwana, Masataka; Nishimura, Hiroaki; Watanabe, Yurika; Nakahara, Jin et al. (2015): Inflammatory myopathy with anti-signal recognition particle antibodies: case series of 100 patients. In *Orphanet journal of rare diseases* 10, p. 61. DOI: 10.1186/s13023-015-0277-y.

Szabo, Katalin; Bodoki, Levente; Nagy-Vincze, Melinda; Vincze, Anett; Zilahi, Erika; Szodoray, Peter et al. (2018): Effect of Genetic and Laboratory Findings on Clinical Course of Antisynthetase Syndrome in a Hungarian Cohort. In *BioMed research international* 2018, p. 6416378. DOI: 10.1155/2018/6416378.

Tarricone, Elena; Ghirardello, Anna; Rampudda, Mariaelisa; Bassi, Nicola; Punzi, Leonardo; Doria, Andrea (2012): Anti-SAE antibodies in autoimmune myositis: identification by unlabelled protein immunoprecipitation in an Italian patient cohort. In *Journal of immunological methods* 384 (1-2), pp. 128–134. DOI: 10.1016/j.jim.2012.07.019.

Teh, C. L.; Wong, J. S.; Soo, H. H. (2012): Polymyositis and dermatomyositis in Sarawak: a profile of patients treated in the Sarawak General Hospital. In *Rheumatology international* 32 (1), pp. 265–268. DOI: 10.1007/s00296-010-1745-2.

Uthman, I.; Vazquez-Abad, D.; Senecal, J. L. (1996): Distinctive features of idiopathic inflammatory myopathies in French Canadians. In *Seminars in arthritis and rheumatism* 26 (1), pp. 447–458. DOI: 10.1016/s0049-0172(96)80025-4.

Wang, Li; Huang, Li; Yang, Yang; Chen, Huan; Liu, Yanjuan; Liu, Ke et al. (2018): Calcinosis and malignancy are rare in Chinese adult patients with myositis and nuclear matrix protein 2 antibodies identified by an unlabeled immunoprecipitation assay. In *Clinical rheumatology* 37 (10), pp. 2731–2739. DOI: 10.1007/s10067-018-4216-x.

Wang, S. J.; Lin, W. Y.; Hsu, C. Y.; Kao, C. H.; Chang, C. P.; Lan, J. L. (1993): Solid phase radionuclide esophageal motility in polymyositis and dermatomyositis. In *Gaoxiong yi xue ke xue za zhi = The Kaohsiung journal of medical sciences* 9 (6), pp. 338–342.

Watanabe, Yurika; Suzuki, Shigeaki; Nishimura, Hiroaki; Murata, Ken-Ya; Kurashige, Takashi; Ikawa, Masamichi et al. (2015): Statins and myotoxic effects associated with anti-3-hydroxy-3-methylglutaryl-coenzyme A reductase autoantibodies: an observational study in Japan. In *Medicine* 94 (4), e416. DOI: 10.1097/MD.0000000000000416.

Watanabe, Yurika; Uruha, Akinori; Suzuki, Shigeaki; Nakahara, Jin; Hamanaka, Kohei; Takayama, Kazuko et al. (2016): Clinical features and prognosis in anti-SRP and anti-HMGCR necrotising myopathy. In *Journal of neurology, neurosurgery, and psychiatry* 87 (10), pp. 1038–1044. DOI: 10.1136/jnnp-2016-313166.

Weitoft, T. (1997): Occurrence of polymyositis in the county of Gavleborg, Sweden. In *Scandinavian journal of rheumatology* 26 (2), pp. 104–106. DOI: 10.3109/03009749709115827.

Williams, R. B.; Grehan, M. J.; Hersch, M.; Andre, J.; Cook, I. J. (2003): Biomechanics, diagnosis, and treatment outcome in inflammatory myopathy presenting as oropharyngeal dysphagia. In *Gut* 52 (4), pp. 471–478.

Willig, T. N.; Paulus, J.; Lacau Saint Guily, J.; Beon, C.; Navarro, J. (1994): Swallowing problems in neuromuscular disorders. In *Archives of physical medicine and rehabilitation* 75 (11), pp. 1175–1181. DOI: 10.1016/0003-9993(94)90001-9.

Wintzen, A. R.; Bots, G. T.; Bakker, H. M. de; Hulshof, J. H.; Padberg, G. W. (1988): Dysphagia in inclusion body myositis. In *Journal of neurology, neurosurgery, and psychiatry* 51 (12), pp. 1542–1545.

Wu, Hao; Geng, Daoying; Xu, Jinhua (2013): An approach to the development of interstitial lung disease in dermatomyositis: a study of 230 cases in China. In *The Journal of international medical research* 41 (2), pp. 493–501. DOI: 10.1177/0300060513476435.

Yin, Liguo; Ge, Yongpeng; Yang, Hanbo; Peng, Qinglin; Lu, Xin; Zhang, Yamei; Wang, Guochun (2016): The clinical utility of serum IL-35 in patients with polymyositis and dermatomyositis. In *Clinical rheumatology* 35 (11), pp. 2715–2721. DOI: 10.1007/s10067-016-3347-1.

Yoo, Juyoung; Ahn, Sung Soo; Jung, Seung Min; Song, Jason Jungsik; Park, Yong-Beom; Lee, Sang-Won (2019): Reclassification of Korean patients with polymyositis and dermatomyositis based on the Bohan and Peter criteria by the 2017 European League Against Rheumatism/American College of Rheumatology classification criteria for adult and juvenile idiopathic inflammatory myopathies. In *The Korean journal of internal medicine*. DOI: 10.3904/kjim.2019.149.

Zhou, Hang; Wang, Yunchao; Bi, Kuo; Qi, Haiyu; Song, Shuju; Zhou, Mingzhu et al. (2019): Serum-soluble TRAIL: a potential biomarker for disease activity in myositis patients. In *Clinical rheumatology* 38 (5), pp. 1425–1431. DOI: 10.1007/s10067-018-04418-9.
