# Supplementary material for: The Impact of Dysphagia in Myositis: A Systematic Review and Meta-Analysis
Source: J Clin Med. 2020 Jul 8;9(7):2150. doi: 10.3390/jcm9072150 (PMC7408750; doi:10.3390/jcm9072150)
Supplement: Supplementary file 1 [file jcm-09-02150-s001.zip › jcm-843942-SI-conversion/supplements/Table S2.docx]

**Table S2:** Studies reporting on pathophysiology of dysphagia in myositis. IIM: idiopathic inflammatory myopathy, DM: dermatomyositis, PM: polymyositis, IBM: inclusion body myositis, ASS: antisynthetase syndrome, JDM: juvenile dermatomyositis, JPM: juvenile polymyositis, VFSS: videofluoroscopy, FEES: flexible endoscopic evaluation of swallowing, HRM: high resolution manometry, EGD: esophagogastroduodenoscopy, EMG: electromyography, LES: lower esophagus sphincter, UES: upper esophagus sphincter, PEG: percutaneous endoscopic gastrostomy, CNS: central nervous system, ASHA: American Speech-Language Hearing Association

| Author | Study design | Cohort | Definition/ assessment of dysphagia | Pathophysiology of dysphagia |
| --- | --- | --- | --- | --- |
| (Almodovar et al. 2012) | case-report | DM, n=1 | barium study of the esophagus, stomach, and duodenum | decreased motility in the proximal esophagus |
| (Azola et al. 2019) | retrospective observational study | IIM with dysphagia; n=23 | VFSS | decreased duration of upper esophageal sphincter opening and laryngeal vestibule closure, higher scoring on the Modified Barium Swallow Impairment Profile with impairments in tongue control and motion, oral and pharyngeal residue, laryngeal elevation and closure, hyoid excursion, and epiglottic movement, penetration aspiration score (PAS) of >6 were observed in 26% of patients with myositis, there was no difference in upper esophageal sphincter diameter in myositis compared to healthy controls |
| (Bachmann et al. 2001) | case-report | IIM, n=1 | laryngoscopy  VFSS  manometry | pooling of saliva in the piriform sinus, stenosis of the UES caused by cricopharyngeal hypertrophy, hypertonic upper esophageal sphincter, postdeglutitive aspiration, signs for cricopharyngeal IIM in biopsy |
| (Badrising et al. 2005) | retrospective observational study | IBM, n=64 | symptoms of dysphagia | not reported  onset of symptoms with quadriceps weakness predicted an earlier onset of dysphagia in older patients (> or = 56 years) compared with younger ones (< 56 years) |
| (Barrera et al. 1998) | case-report | PM associated with systemic sclerosis, n=1 | gastrointestinal series (probably VFSS) | distal esophageal hypomotility, cricopharyngeus muscle spasm, aspiration |
| (Butt et al. 2017) | case-report | NXP-2-positive dermatomyositis, n=1 | barium-swallow | severe swallowing weakness with aspiration |
| (Caramaschi et al. 1997) | case-report | DM, n=1 | esophageal radiographic examination  endoscopic examination of the upper gastrointestinal tract  biopsy of the esophageal mucosa  esophageal manometry | dilation of the esophageal lumen, mild and non-specific chronic inflammation in the mucosa of the esophagus, aperistalsis of esophageal body and reduced pressure at the upper esophageal sphincter. |
| (Casal-Dominguez et al. 2017) | prospective observational study | total cohort IIM, n=53  DM, n=24  PM, n=21  cancer associated myositis, n=5  ASS, n=16  Anti–TIF1‐γ, n = 10  Anti‐PM/Scl, n = 8  Anti‐Ro52, n = 16 | esophageal symptom survey  HRM | most common HRM-findings: ineffective esophageal motility, absent contractility, jackhammer esophagus.  PM patients had an increased percentage of failed waves and significantly decreased UES pressure. Anti–TIF1‐γ patients showed more frequently Jackhammer esophagus, had a higher distal contractile integral, higher intrabolus pressure and more fragmented waves. ASS patients had decreased LES pressure and an increased percentage of low LES pressure values |
| (Cherin et al. 2002) | case-series | IBM, n=4 | tube dependent  esophageal manometry | esophageal manometry: low pressure in the upper esophageal sphincter and reduced peristalsis |
| (Chiang et al. 1997) | case-report | IIM, n=1 | esophagography | narrowing of the esophagus below the piriform sinus, signs for IIM in muscle biopsy |
| (Cochicho et al. 2016) | case-report | PM, n=1 | barium swallow, esophageal manometry | weak esophageal peristalsis, and large peristaltic defects, deficient propulsion of tongue, residue in valleculae, laryngeal penetration. |
| (Cox et al. 2009) | prospective observational study | IBM, n=57 | standard questionnaire regarding dysphagia, VFSS | impaired propulsion: the feeling of food getting stuck in the throat, repetitive swallowing (56%), residue in the valleculae (37%) or piriform sinus (44%) and cricopharyngeal sphincter dysfunction (37%).  aspiration-related dysphagia: choking for more than five times a month, coughing related to eating, drinking or lying down, aspiration (2%), fluid entering the larynx, inadequate epiglottal downward tilting (43%), residues in the valleculae and/or piriform sinus  two questions had high positive predictive value for impaired propulsion: “Is food getting stuck in the throat?” (91%) and “are repeated swallows needed?” (92%), otherwise rather low, especially for aspiration related symptoms |
| (Cunningham, JR and Lowry 1985) | retrospective observational study | PM, n=16  DM, n=16  myositis with calcinosis, n=7  childhood myositis, n=2  overlap syndrome, n=4 | not further stated | aspiration |
| (Curiel et al. 2005) | case-report | DM, n=1 | not further stated | oropharyngeal dysphagia: Increased T2 signal in MRI in the tongue, masticator and pharyngeal muscles. |
| (Dagan et al. 2013) | case-report | DM, n=1 | VFSS | pharyngeal dysphagia with decreased laryngeal elevation, residue in the valleculae and piriform sinus, intradeglutitive aspiration |
| (Dakovic et al. 2009) | case-report | DM, n=1 | barium-swallow  PEG-dependent | regurgitation, aspiration, pharyngeal residue |
| (Danon and Friedman 1989) | case-report | IBM, n=1 | oropharyngeal swallow study | diminished peristalsis in the pharynx and upper esophagus suggestive of a motility disorder, typical signs for chronic inflammation in the cricopharyngeal and omohyoid muscle |
| (Darrow et al. 1992) | case-report | IBM, n=1 | VFSS | cricopharyngeal dysfunction: Hyperreflexive UES with no relaxation, impaired oral bolus transit, decreased laryngeal elevation, increased pharyngeal transit time |
| (Degos et al. 1971) | retrospective observational study | DM, n=31 | not further stated | weakness of pharyngeal muscles, involvement of the buccal mucosa, hypotonia of the esophagus, nasal regurgitation |
| (Della Marca et al. 2013) | prospective observational study | sIBM, n=13 | oro-pharyngo-esophageal scintigraphy | oropharyngeal dysphagia in all cases |
| (Di Pede et al. 2016) | case-series | IBM, n=4 | VFSS, FEES, HRM | cricopharyngeal dysfunction |
| (Dietz et al. 1980) | case-report | PM, n=1 | VFSS | pharyngeal residue, obstruction of the cricopharyngeus muscle, reduced pharyngeal contractility, increased pharyngeal transit time, fibrosis and little evidence of inflammation in cricopharyngeal muscle biopsy |
| (Dobloug et al. 2012) | retrospective observational study | sIBM, n=14; n=6 | subjective report of patients n=6  radiological dynamic studies of the esophagus in the n=14 | esophageal dysmotility |
| (Dobloug et al. 2015) | retrospective observational study | sIBM female, n=40  sIBM male, n=60 | partly examined by dynamic x-ray of the esophagus, otherwise not further stated | esophageal dysmotility was present in 83% of those patients who were examined by x-ray of the esophagus |
| (Donoghue et al. 1960) | retrospective observational study | DM and PM with investi-gation of the esophagus, n=38 | investigation of the esophagus, either by roentgenography, esophagoscopy, or motility studies | diminished or absent esophageal peristalsis, laryngeal aspiration, diaphragmatic hernia, atonic esophagus |
| (Ertekin et al. 1996) | prospective observational study | DM/PM, n=7 | tube-dependent  decreased dysphagia limit in EMG | decreased dysphagia limit: Piecemeal deglutition at volumes below 20ml |
| (Ertekin et al. 2004) | prospective observational study | DM/PM, n=19 | submental EMG  clinical parameters | pharyngeal dysphagia: Piecemeal deglutition when swallowing larger volumes (74%), cricopharyngeal dysfunction (n=5, 50%) with the m. cricopharyngeus showing both hyporeflexive and hyperreflexive states, prolongation of the pharyngeal phase of swallowing |
| (Eura et al. 2015) | case-report | DM, n=1 | VFSS | nasopharyngeal regurgitation, residue in the piriform sinus, edema in oropharyngeal muscles in MRI |
| (Felice and North 2001) | retrospective observational study | sIBM, n=35 | Clinical symptoms  partly VFSS | prominent cricopharyngeus, reduced pharyngeal movement, residue in the piriform sinus, achalasia of UES, Aspiration |
| (Gameiro et al. 2018) | case-report | U1-RNP positive IIM with mixed connective tissue disease, N=1 | tube-dependent  HRM | absent peristalsis in the whole esophagus |
| (Giannini et al. 2018) | case-report | anti-PL12-ASS-and sIBM overlap, n=1 | FEES | lack of oropharyngeal muscle propulsion, stasis of saliva, liquid and solid bolus stagnation in the valleculae, glosso-epiglottic folds, and piriform sinus |
| (Gibson et al. 1991) | case-report | IBM with mixed connected tissue disease, n=1 | not further stated | tongue atrophy |
| (Gorelik et al. 2001) | case-series | IIM with subcutaneous edema, n=1 | barium swallow | aspiration |
| (Gyorffy et al. 2018) | case-report | mixed connective tissue disease, n=1 | barium swallow, EGD, HRM | ineffective esophageal motility |
| (Hafejee and Coulson 2005) | case-report | DM associated with transitional cell carcinoma of the bladder, n=1 | VFSS | postdeglutitive aspiration and impaired peristalsis in the proximal oesophagus |
| (Horowitz et al. 1994) | case-report | DM associated with lyme disease, n=1 | tube dependent  manometry  histology of LES | decreased contractility in the pharynx, chronic inflammation in the LES |
| (Horowitz et al. 1986) | prospective observational study | PM and DM, n=13 | questionnaire on symptoms  measurement of esophageal emptying using scintigraphy | significantly delayed esophageal emptying in comparison with healthy individuals. In 8 of 13 patients, esophageal emptying rates were outside the normal range. All 5 patients with dysphagia had delayed esophageal emptying. Two asymptomatic patients had delayed esophageal emptying outside the normal range.  there was a direct relationship between the clinical grading of peripheral muscle weakness and rates of esophageal emptying (r = 0.83; p < 0.01). None of the 5 patients with minimal or no weakness had delayed esophageal emptying. |
| (Houser et al. 1998) | retrospective and prospective observational study | IBM, n=22 | chart documentation and telephone interview | partly nasal regurgitation and drooling |
| (Iannone et al. 2015) | case-report | DM, n=1 | barium swallow | barium retention in the valleculae and piriform sinus due to  poor hypopharynx emptying and esophageal dyskinesia |
| (Jacob et al. 1983) | prospective observational study | PM or DM, n=13 | questionnaire about symptoms  esophageal manometry  barium swallow examination  partly VFSS | abnormalities in the smooth as well as the striated muscle portion of the esophagus in manometry: impaired distal esophageal peristalsis,  significantly lower mean amplitude of lower esophageal contractions compared to healthy controls, absent proximal esophageal contraction, decreased UES pressure  barium swallow: proximal or distal esophagus dilatation  VFSS: nasal reflux, aspiration, Zenker’s diverticulum |
| (Johnson and McKenzie 1993) | prospective observational study | PM, n=3 | VFSS | pharyngeal residue, aspiration, impaired esophageal peristalsis, cricopharyngeal bar, prolonged "spasm" of the cricopharyngeus muscle, abnormal kinematic pharyngeal transit times |
| (Joshi et al. 2008) | case-report | DM, n=1 | FEES | velo-pharyngeal dysfunction, inadequate pharyngeal bolus clearance, residue in the valleculae and piriform sinus, esophageal dysmotility |
| (Kagen et al. 1985) | case-series | PM and DM, n=3 | VFSS  manometry | cricopharyngeal sphincter hypertrophy, proximal pharyngeal patulous dilatation, aspiration, nasopharyngeal regurgitation, cricopharyngeal achalasia, chronic inflammation in cricopharyngeal muscle biopsy |
| (Kim et al. 2010) | retrospective observational study | DM, n=13 | VFSS  American Speech-Language-Hearing Association (ASHA) level | dysphagia and swallowing muscle involvement had no correlation to motor involvement |
| (Kleine-Natrop and Engel 1967) | case-report | DM, n=1 | x-ray of swallowing | residue in the piriform sinus, diverticula in the esophagus |
| (Ko and Rubin 2014) | case-report | IBM, n=1 | FEES | reduced pharyngeal squeeze bilaterally, elevated sensory thresholds, pharyngeal residue in the valleculae and hypopharynx, which cleared slowly with multiple swallows. |
| (Korn and Mauiyyedi 2001) | case-report | PM, n=1 | barium-swallow examination | lingual and pharyngeal weakness, incomplete velopharyngeal closure, nasopharyngeal reflux, esophageal dysmotility, tertiary contractions and relaxation and opening of the LES |
| (Kwon et al. 2018) | case-report | DM, n=1 | VFSS | inadequate pharyngeal contraction, decreased upper esophageal sphincter opening, pharyngeal residue in the valleculae and piriform sinus, silent aspiration |
| (Labeit et al. 2019) | case-report | Anti-Jo-1-positive IIM, n=1 | FEES, VFSS, HRM | pre-, intra- and postdeglutitive aspiration, residue in the pyriform sinus and valleculae, impairment of the UES |
| (Langdon et al. 2012) | prospective observational study | IIM, n=18 | VFSS  esophageal manometry | mainly pharyngeal dysphagia in VFSS:  delayed swallow initiation (24%), decreased hyolaryngeal excursion (22%), pyriform residue (17%), penetration (13%), vallecular residue (9%), aspiration events (4%). Percentage refers to the number of swallows investigated. Manometry findings: failed EUS relaxation was seen in 11% of patients.  conclusion: dysphagia in IIM patients appears to be more due to impaired muscle contraction and reduced hyolaryngeal excursion than the often-held belief of failed UES relaxation. |
| (Laurikainen et al. 1992) | prospective observational study | IIM, n=1 | VFSS  fiberoptic esophagoscopy  manometry | inflammation in the cricopharyngeus muscle |
| (Liu et al. 2004) | case-series | IBM, n=2 | VFSS  esophageal manometry | patient 1: Premature bolus spillage, pharyngeal residue, aspiration  patient 2: cricopharyngeus muscle hypertrophy and spasm, poor contraction of the pharynx, elevated UES pressure |
| (Lotz et al. 1989) | retrospective observational study | IBM, n=40 | partly barium swallow | decreased or absent peristalsis (42%), prominent cricopharyngeus muscle (42%), proximal stricture (25%), Zenker’s diverticulum (17%), distal diverticulum (8%) |
| (Malandraki et al. 2012) | case-report | IBM, n=1 | VFSS | reduced UES opening and base of tongue retraction, pharyngeal residue, aspiration and penetration, delayed pharyngeal swallow |
| (Margulis and Koehler 1976) | case-report | DM, n=1 | x-ray of swallowing | residue in the piriform sinus, diverticula in the esophagus |
| (Marie et al. 2001) | retrospective observational study | PM/DM, n=77 | manometric esophageal impairment | esophageal involvement in manometry |
| (Marie et al. 1999a) | case-series | PM/DM, n=3 | Esophageal manometry | low pressure in the UES and LES, hypomotility in the esophagus |
| (Marie et al. 1999b) | retrospective observational study | DM/PM, n=79  DM/PM > 65 years, n=23  DM/PM < 65 years, n= | esophageal manometry | esophageal hypomotility |
| (Marie et al. 2010) | retrospective observational study | PM/DM, n=301 | clinical manifestation,  esophageal manometry | low pressure in the UES, decreased peristalsis in the upper third of the esophageal body, absence of peristalsis in the upper third of the esophageal body, decreased peristalsis within the lower two‐thirds of the esophageal body, impaired cricopharyngeal muscle relaxation |
| (McCann et al. 2007) | prospective observational study | JDM, n=14 | VFSS | 29% aspiration, 14% pharyngeal residue, 14% pharyngeal residue in the piriform sinus, 7% nasal regurgitation, 7% pharyngeal wall weakness, 7% delay in swallow  there was no correlation between swallow score and objective measures of muscle strength and function or general disease activity and function |
| (Merieux et al. 1983) | prospective and retrospective observational study | PM or DM in the prospective cohort, n=16  autopsy of patients with PM or DM, n=18 | partly cineradiography | radiological findings: abnormal motility during deglutition, abnormal motility in the proximal esophagus, abnormal motility in the distal esophagus, dilatation, hiatal hernia, structure, prominent cricopharyngeus muscle  autopsy finding: ulcerative lesions involving the mucosa with accompanying edema and/or hemorrhage, mucosal atrophy, atrophy of esophageal smoot muscle, abnormal fibrosis of the esophageal wall  no correlation of autopsy findings with previous radiological findings, no lymphatic infiltrations in the smooth muscles |
| (Metheny 1978) | case-series | DM, n=2 | laryngoscopy, indirect laryngoscopy in 1 patient and barium swallow in 1 patient | pooling of saliva in the piriform sinus, dysfunction of the esophagus, signs for DM in cricopharyngeal muscle biopsy, signs for DM in esophageal muscle biopsy |
| (Mii et al. 2006) | case-report | DM, n=1 | swallow x-ray film  tube-dependent | obstruction in the upper esophagus |
| (Mugii et al. 2016) | prospective observational study | DM, n=92 | clinical observation by otorhinolaryngologists and speech therapists  partly VFSS | only pharyngeal dysphagia, no oral or esophageal involvement was observed: pharyngeal pooling, nasal regurgitation |
| (Murata et al. 2012) | prospective observational study | sIBM, n=10 | VFSS,  computed pharyngoesophageal manometry | VFSS: pharyngeal muscle propulsion between C3 and C7 (100%), pharyngeal residue in the epiglottic valleculae (80%), insufficiency of UES opening (50%), pharyngeal residue in the piriform sinus (50%), penetration (50%);  manometry: no or incomplete UES relaxation (80%), decreased oro-hypopharyngeal pressure (60%), decreased deglutitive UES pressure (60%); percentage in relation to number of patients.  Additional information: IBM patients were affected more severely, symptomatic patients were more severely affected. |
| (Murata et al. 2013) | case-series | sIBM, n=3 | VFSS  computed pharyngo-esophageal manometry | cricopharyngeal dysfunction: The UES opening was impaired and aspiration was confirmed due to an influx of remaining food from the piriform sinus |
| (Nagano et al. 2009) | case-report | PM, n=1 | VFSS | saliva pooling in the piriform sinus, cricopharyngeal dysfunction: difficulty with dilatation of the esophagus orifice, pharyngeal residue, velopharyngeal insufficiency |
| (Ofori et al. 2017) | case-report | DM, n=1 | EGD  PEG-tube-dependent | pooling of saliva in the piriform sinus |
| (Oh et al. 2008) | retrospective observational study | IBM with dysphagia, n=26 | partly clinical dysphagia evaluation (n=24), VFSS (n=23), and pharyngo-esophageal  manometry (n=12) | VFSS findings: impairment in tongue control (39%),  bolus control (52%), tongue base retraction (74%), laryngeal elevation (43%), pharyngeal constrictor contraction (57%), pharyngeal residue (91%), cricopharyngeal dysfunction (57%),  penetration (70%), aspiration (35%).  manometry: low amplitude pharyngeal constrictor contraction (75%), normal resting tone and relaxation of the UES (82%), and diminished inferior esophageal sphincter pressure  (42%)  cricopharyngeal biopsy (n=1): cricopharyngeal myositis. |
| (Oh et al. 2007) | retrospective observational study | IIM with dysphagia, n=62 | partly VFSS (n=38) | symptoms: “food sticking in throat” (85%), “coughing while eating” (75%), and “difficulty with solid and dry foods“ (96%)  VFSS:  pharyngeal pooling (85%), impaired tongue base retraction (76%), impaired laryngeal elevation (50%), abnormal cricopharyngeal function (47%), aspiration (24%) |
| (O'Hara et al. 1967) | case-report | DM, n=1 | VFSS | abnormalities in barium swallow examination: nasal reflux during  deglutition, aspiration, pharyngeal residue, generalized loss of motility, moderate dilatation, and significant diminution of peristalsis in the esophagus  abnormality in the autopsy: irregular defects involving the esophageal mucosa and edges of the esophageal wall, irregular elongated shallow diverticula in the esophagus, absence of longitudinal mucosal folds in the area of the lesions, submucosal fibrosis, atrophy of the muscularis mucosa and occasional small mononuclear inflammatory cells in the smooth muscle of the esophagus |
| (Olthoff et al. 2016) | prospective observational study | IBM without severe aspiration according to patient history, n=20 | VFSS  FEES  real-time-MRI | cricopharyngeal propulsion, prolonged pharyngeal transit time, pharyngeal residue (retention), penetration |
| (Otao et al. 2007) | case-report | DM, n=1 | not further stated | high intensity lesions in the mylohyoid and geniohyoid muscle in T2-weighted fat-saturated images of MRI |
| (Paik et al. 2008) | prospective observational study | DM/PM with aspiration in VFSS, n=3 | VFSS | mean functional dysphagia scale: 30.3 ± 17.5.  horizontal excursion of the hyoid bone and rotation of the epiglottis were significantly reduced compared to healthy controls and stroke patients with aspiration |
| (Palace et al. 1993) | case-report | PM, n=1 | VFSS | nasal regurgitation, reduced laryngeal elevation, reduced pharyngeal peristalsis, residue in the piriform sinus, aspiration |
| (Peng et al. 2014) | prospective observational study | mixed group of PM and DM, n=98 | not further stated | patients with oropharyngeal dysphagia had significantly higher Fn14 mRNA levels than the patients without oropharyngeal dysphagia |
| (Porkodi et al. 2002) | observational study (unclear if retrospective or prospective) | PM, n=24  DM, n=27  DM with malignancy, n=1  JDM/JPM, n=5  overlap syndrome, n=30 | not further stated | partly nasal regurgitation |
| (Porubsky et al. 1973) | case-report | DM, n=1 | VFSS | aspiration, obstruction of the cricopharyngeus muscle |
| (Ramachandran and Swash 2004) | case-series | DM, n=2 | VFSS | oropharyngeal dysphagia: increased oral and pharyngeal transit time, reduced laryngeal elevation, pharyngeal residue in the valleculae, signs of aspiration |
| (Riminton et al. 1993) | case-series | IBM, n=2 | VFSS, manometry, cine barium swallowing study | nasal regurgitation, pharyngeal hypomotility, residue in the piriform sinus, narrowing at the level of the cricopharyngeus muscle, aspiration, elevation of cricopharyngeal pressure in manometry, signs for cricopharyngeal IBM in biopsy |
| (Ryan et al. 2003) | case-report | PM, n=1 | VFSS | oropharyngeal weakness, pooling of secretions, silent aspiration |
| (Schrey et al. 2017) | retrospective observational study | sIBM, n=40  sIBM with neurotoxin A injection-therapy (BoNT-A), n=12 | VFSS  EGD | cricopharyngeal dysfunction i.e. either obstruction to thick or solid barium at the UES or a cricopharyngeal bar that obstructs 50% or more of the lumen throughout the swallow |
| (Shapiro et al. 1996) | case-series | isolated oropharyngeal IIM, n=3 | FEES  VFSS | pharyngeal dysphagia: pooling of saliva in the pharyngeal recesses, prominent cricopharyngeus muscle, prominent proximal portion of the inferior constrictor muscle, decreased epiglottic tilt, aspiration, reverse pharyngeal clearance as posterior movement of the tongue and anterior movement of the posterior pharyngeal wall, cricopharyngeal inflammatory myopathy |
| (Shibata et al. 2017) | case-report | sIBM, n=1 | VFSS | UES dysfunction, residue in the piriform sinus, decreased laryngeal elevation |
| (St Guily et al. 1994) | retrospective observational study | PM, n=3 | clinical symptoms  FEES  partly manometry  partly VFSS | decreased pharyngeal propulsion, disturbed UES |
| (Stevens et al. 1964) | prospective observational study | IIM, n=6  PM, n=2  DM, n=4 | esophageal manometry | pharyngeal weakness |
| (Takamiya et al. 2019) | case-report | anti-NT5C1A-positive IBM, n=1 | VFSS | residue in the piriform sinus and valleculae |
| (Tang et al. 2018) | case-report | IIM, n=1 | barium radiography, HRM, EGD | EGD: corkscrew like appearance of esophagus  barium radiography: spasmodic contraction of distal esophagus, and narrow of esophageal cavity  HRM: high amplitude distal esophageal contraction  esophageal biopsy: smooth muscle layer showed eosinophilic infiltration into the muscularis propria |
| (Thomas et al. 1972) | case-report | PM, n=1 | VFSS | narrowing and rigidity of the distal, cervical esophagus, pharyngeal residue in the valleculae and piriform sinus, signs for inflammation in muscle biopsy of distal cervical esophagus |
| (Tierney and Jirjis 1997) | case-report | DM, n=1 | VFSS | decreased tongue control, penetration |
| (Uthman et al. 1996) | retrospective observational study | IIM in French Canadians (PM and DM), n=30 | not further stated  partly barium swallow | barium swallow: abnormal motility  of the esophagus both upper third and middle |
| (Vencovsky et al. 1988) | case-report | PM, n=1 | VFSS  esophagoscopy | residue of saliva, cricopharyngeal obstruction with aspiration, muscle biopsy: cricopharyngeal inflammatory myopathy with intermysial fibrosis |
| (Verma et al. 1991) | case-report | IBM, n=1 | upper gastrointestinal radiographic study  esophagoscopy  barium-swallow-video | cricopharyngeal achalasia with aspiration, cricopharyngeal narrowing without fixed stricture, cricopharyngeal inflammation |
| (Wanamaker et al. 1992) | case-report | PM, n=1 | not further stated | enlargement of the entire left sternocleidomastoid muscle, from  its origin to its insertion with preservation of fat planes |
| (Wang et al. 1993) | prospective observational study | IIM, n=23 | increased esophageal transit time in scintigraphy | increased mean esophageal transit time, increased esophageal residue fraction |
| (Wenzel et al. 2001) | case-report | IBM associated with subacute cutaneous lupus erythematosus, n=1 | scintigraphy | reduced esophageal motility |
| (Williams et al. 2003) | retrospective observational study | IIM with oropharyngeal dysphagia, n=13 | oropharyngeal dysphagia was defined as difficulty with the act of swallowing together with one or more of the following deglutitive symptoms: bolus holdup; multiple swallows required to clear the pharynx; deglutitive coughing and/or choking; or postnasal regurgitation  partly VFSS and manometry | VFSS: restrictive cricopharyngeal abnormalities (69%), aspiration (61%), pharyngo-oesophageal stenosis (38%), cricopharyngeal bar (31%), Zenker’s diverticula (23%)  manometry: decreased peak pharyngeal pressure (46%)  biopsy: inflammatory changes in the cricopharyngeal muscle  restrictive pharyngo-oesophageal segment abnormalities were significantly more often in in patients with IIM than in controls with central nervous system (CNS) pathology. In none of the IIM patients was the pharyngeal swallow response absent compared to 21% in controls with CNS pathology. Maximal sagittal UOS opening diameter was significantly reduced in IIM patients compared to controls with CNS pathology (the extent of sphincter opening was reduced in IIM). IIM patients demonstrated a bolus volume dependent increase in hypopharyngeal intrabolus pressure which was significantly greater than that seen in controls with CNS pathology. Temporal coordination seemed to be less disrupted in IIM compared to controls with CNS pathology |
| (Wintzen et al. 1988) | case-series | IBM, n=6 | VFSS | paretic widening of the hypopharynx, prominent posterior indentation as a result of contraction of the cricopharyngeus muscle, pharyngeal residue, Zenker's pulsion diverticulum, aspiration, signs for chronic inflammation in the omohyoid muscle biopsy |
| (Wolman et al. 1965) | retrospective observational study | rheumatoid arthritis and autopsy of the larynx, n=8 | clinical symptoms | 4 patients showed signs for PM in the laryngeal muscles, 1 of whom had clinical signs for dysphagia, the other patients had signs for stridor |
| (Yin et al. 2016) | retrospective observational study | IIM without IBM, n=104 | not further stated | patients with dysphagia had significantly higher serum IL-35 |
| (Zuber et al. 2013) | case-report | DM, n=1 | PEG-tube dependent | edema of oropharyngeal muscles in MRI |

Publication bibliography

Almodovar, Raquel; Lindo, Daniel Paul; Martin, Helena; Mazzuchelli, Ramon; Pardo, Javier; Quiros, Francisco Javier; Zarco, Pedro (2012): Dermatomyositis and meningioma in the same patient. In *Reumatologia clinica* 8 (2), pp. 87–89. DOI: 10.1016/j.reuma.2011.06.009.

Azola, Alba; Mulheren, Rachel; Mckeon, Genevieve; Lloyd, Thomas; Christopher-Stine, Lisa; Palmer, Jeffrey; Chung, Tae Hwan (2019): "Dysphagia in myositis: A study of the structural and physiologic changes resulting in disordered swallowing.". In *American journal of physical medicine & rehabilitation*. DOI: 10.1097/PHM.0000000000001354.

Bachmann, G.; Streppel, M.; Krug, B.; Neuen-Jacob, E. (2001): Cricopharyngeal muscle hypertrophy associated with florid myositis. In *Dysphagia* 16 (4), pp. 244–248. DOI: 10.1007/s00455-001-0082-8.

Badrising, Umesh A.; Maat-Schieman, Marion L. C.; van Houwelingen, Johannes C.; van Doorn, Peter A.; van Duinen, Sjoerd G.; van Engelen, Baziel G M et al. (2005): Inclusion body myositis. Clinical features and clinical course of the disease in 64 patients. In *Journal of neurology* 252 (12), pp. 1448–1454. DOI: 10.1007/s00415-005-0884-y.

Barrera, P.; den Broeder, A. A.; van den Hoogen, F H; van Engelen, B. G.; van de Putte, L B (1998): Postural changes, dysphagia, and systemic sclerosis. In *Annals of the rheumatic diseases* 57 (6), pp. 331–338. DOI: 10.1136/ard.57.6.331.

Butt, Zeeshan; Patel, Leeza; Das, Manash K.; Mecoli, Christopher A.; Ramji, Alim (2017): NXP-2 Positive Dermatomyositis: A Unique Clinical Presentation. In *Case reports in rheumatology* 2017, p. 4817275. DOI: 10.1155/2017/4817275.

Caramaschi, P.; Blasi, D.; Carletto, A.; Randon, M.; Bambara, L. M. (1997): Megaoesophagus in a patient affected by dermatomyositis. In *Clinical rheumatology* 16 (1), pp. 106–107. DOI: 10.1007/bf02238774.

Casal-Dominguez, Maria; Pinal-Fernandez, Iago; Mego, Marianela; Accarino, Anna; Jubany, Lluis; Azpiroz, Fernando; Selva-O'callaghan, Albert (2017): High-resolution manometry in patients with idiopathic inflammatory myopathy. Elevated prevalence of esophageal involvement and differences according to autoantibody status and clinical subset. In *Muscle & nerve* 56 (3), pp. 386–392. DOI: 10.1002/mus.25507.

Cherin, P.; Pelletier, S.; Teixeira, A.; Laforet, P.; Simon, A.; Herson, S.; Eymard, B. (2002): Intravenous immunoglobulin for dysphagia of inclusion body myositis. In *Neurology* 58 (2), p. 326.

Chiang, I. P.; Wang, J.; Tsang, Y. M.; Hsiao, C. H. (1997): Focal myositis of esophagus: a distinct inflammatory pseudotumor mimicking esophageal malignancy. In *The American journal of gastroenterology* 92 (1), pp. 174–175.

Cochicho, Joana; Madaleno, Joao; Louro, Emilia; Simao, Adelia; Carvalho, Armando (2016): Polymyositis and the Spectrum of Scleroderma Disorders. In *European journal of case reports in internal medicine* 3 (1), p. 346. DOI: 10.12890/2015_000346.

Cox, F. M.; Verschuuren, J. J.; Verbist, B. M.; Niks, E. H.; Wintzen, A. R.; Badrising, U. A. (2009): Detecting dysphagia in inclusion body myositis. In *Journal of neurology* 256 (12), pp. 2009–2013. DOI: 10.1007/s00415-009-5229-9.

Cunningham, J. D., JR; Lowry, L. D. (1985): Head and neck manifestations of dermatomyositis-polymyositis. In *Otolaryngology--head and neck surgery : official journal of American Academy of Otolaryngology-Head and Neck Surgery* 93 (5), pp. 673–677.

Curiel, Rodolfo V.; Brindle, Kathleen A.; Kressel, Bruce R.; Katz, James D. (2005): Dysphagia after testicular cancer. In *Arthritis and rheumatism* 52 (12), p. 3712. DOI: 10.1002/art.21614.

Dagan, Amir; Markovits, Doron; Braun-Moscovici, Yolanda; Rozin, Alexander; Toledano, Kohava; Balbir-Gurman, Alexandra (2013): Life-threatening oropharyngeal aphagia as the major manifestation of dermatomyositis. In *The Israel Medical Association journal : IMAJ* 15 (8), pp. 453–455.

Dakovic, Zorana; Vesic, Sonja; Tomovic, Maja; Vukovic, Jelena (2009): Oropharyngeal dysphagia as dominant and life-threatening symptom in dermatomyositis. In *Vojnosanitetski pregled* 66 (8), pp. 671–674. DOI: 10.2298/vsp0908671d.

Danon, M. J.; Friedman, M. (1989): Inclusion body myositis associated with progressive dysphagia: treatment with cricopharyngeal myotomy. In *The Canadian journal of neurological sciences. Le journal canadien des sciences neurologiques* 16 (4), pp. 436–438. DOI: 10.1017/s031716710002953x.

Darrow, D. H.; Hoffman, H. T.; Barnes, G. J.; Wiley, C. A. (1992): Management of dysphagia in inclusion body myositis. In *Archives of otolaryngology--head & neck surgery* 118 (3), pp. 313–317. DOI: 10.1001/archotol.1992.01880030103021.

Degos, R.; Civatte, J.; Belaich, S.; Delarue, A. (1971): The prognosis of adult dermatomyositis. In *Transactions of the St. John's Hospital Dermatological Society* 57 (1), pp. 98–104.

Della Marca, Giacomo; Sancricca, Cristina; Losurdo, Anna; Di Blasi, Chiara; Fino, Chiara de; Morosetti, Roberta et al. (2013): Sleep disordered breathing in a cohort of patients with sporadic inclusion body myositis. In *Clinical neurophysiology : official journal of the International Federation of Clinical Neurophysiology* 124 (8), pp. 1615–1621. DOI: 10.1016/j.clinph.2013.03.002.

Di Pede, Chiara; Masiero, Stefano; Bonsangue, Valentina; Ragona, Rosario Marchese; Del Felice, Alessandra (2016): Botulinum toxin and rehabilitation treatment in inclusion body myositis for severe oropharyngeal dysphagia. In *Neurological sciences : official journal of the Italian Neurological Society and of the Italian Society of Clinical Neurophysiology* 37 (10), pp. 1743–1745. DOI: 10.1007/s10072-016-2586-x.

Dietz, F.; Logeman, J. A.; Sahgal, V.; Schmid, F. R. (1980): Cricopharyngeal muscle dysfunction in the differential diagnosis of dysphagia in polymyositis. In *Arthritis and rheumatism* 23 (4), pp. 491–495. DOI: 10.1002/art.1780230412.

Dobloug, Cecilie; Walle-Hansen, Ragnhild; Gran, Jan Tore; Molberg, Oyvind (2012): Long-term follow-up of sporadic inclusion body myositis treated with intravenous immunoglobulin: a retrospective study of 16 patients. In *Clinical and experimental rheumatology* 30 (6), pp. 838–842.

Dobloug, G. C.; Antal, E. A.; Sveberg, L.; Garen, T.; Bitter, H.; Stjarne, J. et al. (2015): High prevalence of inclusion body myositis in Norway; a population-based clinical epidemiology study. In *European journal of neurology* 22 (4), 672-e41. DOI: 10.1111/ene.12627.

DONOGHUE, F. E.; WINKELMANN, R. K.; MOERSCH, H. J. (1960): Esophageal defects in dermatomyositis. In *The Annals of otology, rhinology, and laryngology* 69, pp. 1139–1145. DOI: 10.1177/000348946006900422.

Ertekin, C.; Aydogdu, I.; Yuceyar, N. (1996): Piecemeal deglutition and dysphagia limit in normal subjects and in patients with swallowing disorders. In *Journal of neurology, neurosurgery, and psychiatry* 61 (5), pp. 491–496. DOI: 10.1136/jnnp.61.5.491.

Ertekin, Cumhur; Secil, Yaprak; Yuceyar, Nur; Aydogdu, Ibrahim (2004): Oropharyngeal dysphagia in polymyositis/dermatomyositis. In *Clinical neurology and neurosurgery* 107 (1), pp. 32–37. DOI: 10.1016/j.clineuro.2004.02.024.

Eura, Nobuyuki; Sugie, Kazuma; Kiriyama, Takao; Ueno, Satoshi (2015): Characteristic dysphagia as a manifestation of dermatomyositis on oropharyngeal muscle imaging. In *Journal of clinical rheumatology : practical reports on rheumatic & musculoskeletal diseases* 21 (2), pp. 105–106. DOI: 10.1097/RHU.0000000000000225.

Felice, K. J.; North, W. A. (2001): Inclusion body myositis in Connecticut: observations in 35 patients during an 8-year period. In *Medicine* 80 (5), pp. 320–327. DOI: 10.1097/00005792-200109000-00006.

Gameiro, Rita de Sousa; Reis, Ana Isabel Alves; Grilo, Ana Cristina; Noronha, Carla (2018): Following leads: connecting dysphagia to mixed connective tissue disease. In *BMJ case reports* 2018. DOI: 10.1136/bcr-2017-223699.

Giannini, M.; Macchia, L.; Amati, A.; Lia, A.; Girolamo, F.; D'Abbicco, D. et al. (2018): A rare association of anti-alanine-transfer RNA synthetase (anti-PL12) syndrome and sporadic inclusion body myositis. In *Scandinavian journal of rheumatology* 47 (4), pp. 336–337. DOI: 10.1080/03009742.2017.1350747.

Gibson, J.; Lamey, P. J.; Zoma, A.; Ballantyne, J. (1991): Tongue atrophy in mixed connective tissue disease. In *Oral surgery, oral medicine, and oral pathology* 71 (3), pp. 294–296. DOI: 10.1016/0030-4220(91)90302-s.

Gorelik, O.; Almoznino-Sarafian, D.; Alon, I.; Rapoport, M. J.; Goltsman, G.; Herbert, M. et al. (2001): Acute inflammatory myopathy with severe subcutaneous edema, a new variant? Report of two cases and review of the literature. In *Rheumatology international* 20 (4), pp. 163–166. DOI: 10.1007/s002960000093.

Gyorffy, Janelle B.; Marowske, Johanna; Gancayco, John (2018): A Rare Cause of Dysphagia and Weight Loss. In *Case reports in gastroenterology* 12 (3), pp. 640–645. DOI: 10.1159/000493919.

Hafejee, A.; Coulson, I. H. (2005): Dysphagia in dermatomyositis secondary to bladder cancer: rapid response to combined immunoglobulin and methylprednisolone. In *Clinical and experimental dermatology* 30 (1), pp. 93–94. DOI: 10.1111/j.1365-2230.2004.01671.x.

Horowitz, H. W.; Sanghera, K.; Goldberg, N.; Pechman, D.; Kamer, R.; Duray, P.; Weinstein, A. (1994): Dermatomyositis associated with Lyme disease: case report and review of Lyme myositis. In *Clinical infectious diseases : an official publication of the Infectious Diseases Society of America* 18 (2), pp. 166–171. DOI: 10.1093/clinids/18.2.166.

Horowitz, M.; McNeil, J. D.; Maddern, G. J.; Collins, P. J.; Shearman, D. J. (1986): Abnormalities of gastric and esophageal emptying in polymyositis and dermatomyositis. In *Gastroenterology* 90 (2), pp. 434–439. DOI: 10.1016/0016-5085(86)90944-3.

Houser, S. M.; Calabrese, L. H.; Strome, M. (1998): Dysphagia in patients with inclusion body myositis. In *The Laryngoscope* 108 (7), pp. 1001–1005. DOI: 10.1097/00005537-199807000-00009.

Iannone, Florenzo; Giannini, Margherita; Lapadula, Giovanni (2015): Recovery of barium swallow radiographic abnormalities in a patient with dermatomyositis and severe dysphagia after high-dose intravenous immunoglobulins. In *Journal of clinical rheumatology : practical reports on rheumatic & musculoskeletal diseases* 21 (4), p. 227. DOI: 10.1097/RHU.0000000000000252.

Jacob, H.; Berkowitz, D.; McDonald, E.; Bernstein, L. H.; Beneventano, T. (1983): The esophageal motility disorder of polymyositis. A prospective study. In *Archives of internal medicine* 143 (12), pp. 2262–2264.

Johnson, E. R.; McKenzie, S. W. (1993): Kinematic pharyngeal transit times in myopathy: evaluation for dysphagia. In *Dysphagia* 8 (1), pp. 35–40. DOI: 10.1007/bf01351476.

Joshi, Deepak; Mahmood, Rizwan; Williams, Peter; Kitchen, Paul (2008): Dysphagia secondary to dermatomyositis treated successfully with intravenous immunoglobulin: a case report. In *International archives of medicine* 1 (1), p. 12. DOI: 10.1186/1755-7682-1-12.

Kagen, L. J.; Hochman, R. B.; Strong, E. W. (1985): Cricopharyngeal obstruction in inflammatory myopathy (polymyositis/dermatomyositis). Report of three cases and review of the literature. In *Arthritis and rheumatism* 28 (6), pp. 630–636. DOI: 10.1002/art.1780280606.

Kim, S. J.; Han, T. R.; Jeong, S. J.; Beom, J. W. (2010): Comparison between swallowing-related and limb muscle involvement in dermatomyositis patients. In *Scandinavian journal of rheumatology* 39 (4), pp. 336–340. DOI: 10.3109/03009740903555366.

Kleine-Natrop, H. E.; Engel, S. (1967): Participation of smooth muscles in dermatomyositis. I. In *Archiv fur klinische und experimentelle Dermatologie* 228 (4), pp. 353–363.

Ko, Ellen H.; Rubin, Adam D. (2014): Dysphagia due to inclusion body myositis: case presentation and review of the literature. In *The Annals of otology, rhinology, and laryngology* 123 (9), pp. 605–608. DOI: 10.1177/0003489414525588.

Korn, Joseph H.; Mauiyyedi, Shamila (2001): Case 26-2001. In *The New England journal of medicine* 345 (8), pp. 596–605. DOI: 10.1056/NEJMcpc010026.

Kwon, Kyoung Min; Lee, Jung Soo; Kim, Yeo Hyung (2018): A case report of life-threatening acute dysphagia in dermatomyositis: Challenges in diagnosis and treatment. In *Medicine* 97 (17), e0508. DOI: 10.1097/MD.0000000000010508.

Labeit, Bendix; Muhle, Paul; Suntrup-Krueger, Sonja; Ahring, Sigrid; Ruck, Tobias; Dziewas, Rainer; Warnecke, Tobias (2019): Dysphagia as Isolated Manifestation of Jo-1 Associated Myositis? In *Frontiers in neurology* 10, p. 739. DOI: 10.3389/fneur.2019.00739.

Langdon, P. Claire; Mulcahy, Kylie; Shepherd, Kelly L.; Low, Vincent H.; Mastaglia, Frank L. (2012): Pharyngeal dysphagia in inflammatory muscle diseases resulting from impaired suprahyoid musculature. In *Dysphagia* 27 (3), pp. 408–417. DOI: 10.1007/s00455-011-9384-7.

Laurikainen, E.; Aitasalo, K.; Halonen, P.; Falck, B.; Kalimo, H. (1992): Muscle pathology in idiopathic cricopharyngeal dysphagia. Enzyme histochemical and electron microscopic findings. In *European archives of oto-rhino-laryngology : official journal of the European Federation of Oto-Rhino-Laryngological Societies (EUFOS) : affiliated with the German Society for Oto-Rhino-Laryngology - Head and Neck Surgery* 249 (4), pp. 216–223. DOI: 10.1007/bf00178473.

Liu, Louis W. C.; Tarnopolsky, Mark; Armstrong, David (2004): Injection of botulinum toxin A to the upper esophageal sphincter for oropharyngeal dysphagia in two patients with inclusion body myositis. In *Canadian journal of gastroenterology = Journal canadien de gastroenterologie* 18 (6), pp. 397–399. DOI: 10.1155/2004/360537.

Lotz, B. P.; Engel, A. G.; Nishino, H.; Stevens, J. C.; Litchy, W. J. (1989): Inclusion body myositis. Observations in 40 patients. In *Brain : a journal of neurology* 112 (Pt 3), pp. 727–747. DOI: 10.1093/brain/112.3.727.

Malandraki, Georgia A.; Kaufman, Andrew; Hind, Jacqueline; Ennis, Stephanie; Gangnon, Ronald; Waclawik, Andrew; Robbins, Joanne (2012): The effects of lingual intervention in a patient with inclusion body myositis and Sjogren's syndrome: a longitudinal case study. In *Archives of physical medicine and rehabilitation* 93 (8), pp. 1469–1475. DOI: 10.1016/j.apmr.2012.02.010.

Margulis, A. R.; Koehler, R. E. (1976): Radiologic diagnosis of disordered esophageal motility: a unified physiologic approach. In *Radiologic clinics of North America* 14 (3), pp. 429–439.

Marie, I.; Hachulla, E.; Hatron, P. Y.; Hellot, M. F.; Levesque, H.; Devulder, B.; Courtois, H. (2001): Polymyositis and dermatomyositis: short term and longterm outcome, and predictive factors of prognosis. In *The Journal of rheumatology* 28 (10), pp. 2230–2237.

Marie, I.; Hachulla, E.; Levesque, H.; Reumont, G.; Ducrotte, P.; Cailleux, N. et al. (1999a): Intravenous immunoglobulins as treatment of life threatening esophageal involvement in polymyositis and dermatomyositis. In *The Journal of rheumatology* 26 (12), pp. 2706–2709.

Marie, I.; Hatron, P. Y.; Levesque, H.; Hachulla, E.; Hellot, M. F.; Michon-Pasturel, U. et al. (1999b): Influence of age on characteristics of polymyositis and dermatomyositis in adults. In *Medicine* 78 (3), pp. 139–147. DOI: 10.1097/00005792-199905000-00001.

Marie, I.; Menard, J-F; Hatron, P. Y.; Hachulla, E.; Mouthon, L.; Tiev, K. et al. (2010): Intravenous immunoglobulins for steroid-refractory esophageal involvement related to polymyositis and dermatomyositis: a series of 73 patients. In *Arthritis care & research* 62 (12), pp. 1748–1755. DOI: 10.1002/acr.20325.

McCann, L. J.; Garay, S. M.; Ryan, M. M.; Harris, R.; Riley, P.; Pilkington, C. A. (2007): Oropharyngeal dysphagia in juvenile dermatomyositis (JDM): an evaluation of videofluoroscopy swallow study (VFSS) changes in relation to clinical symptoms and objective muscle scores. In *Rheumatology (Oxford, England)* 46 (8), pp. 1363–1366. DOI: 10.1093/rheumatology/kem131.

Merieux, P. de; Verity, M. A.; Clements, P. J.; Paulus, H. E. (1983): Esophageal abnormalities and dysphagia in polymyositis and dermatomyositis. In *Arthritis and rheumatism* 26 (8), pp. 961–968. DOI: 10.1002/art.1780260804.

Metheny, J. A. (1978): Dermatomyositis: a vocal and swallowing disease entity. In *The Laryngoscope* 88 (1 Pt 1), pp. 147–161. DOI: 10.1002/lary.1978.88.1.147.

Mii, Sumiyuki; Niiyama, Shiro; Kusunoki, Mai; Arai, Satoru; Katsuoka, Kensei (2006): Cyclosporine A as treatment of esophageal involvement in dermatomyositis. In *Rheumatology international* 27 (2), pp. 183–185. DOI: 10.1007/s00296-006-0168-6.

Mugii, Naoki; Hasegawa, Minoru; Matsushita, Takashi; Hamaguchi, Yasuhito; Oohata, Sacihe; Okita, Hirokazu et al. (2016): Oropharyngeal Dysphagia in Dermatomyositis: Associations with Clinical and Laboratory Features Including Autoantibodies. In *PloS one* 11 (5), e0154746. DOI: 10.1371/journal.pone.0154746.

Murata, Ken-Ya; Kouda, Ken; Tajima, Fumihiro; Kondo, Tomoyoshi (2012): A dysphagia study in patients with sporadic inclusion body myositis (s-IBM). In *Neurological sciences : official journal of the Italian Neurological Society and of the Italian Society of Clinical Neurophysiology* 33 (4), pp. 765–770. DOI: 10.1007/s10072-011-0814-y.

Murata, Ken-Ya; Kouda, Ken; Tajima, Fumihiro; Kondo, Tomoyoshi (2013): Balloon dilation in sporadic inclusion body myositis patients with Dysphagia. In *Clinical medicine insights. Case reports* 6, pp. 1–7. DOI: 10.4137/CCRep.S10200.

Nagano, Hiromi; Yoshifuku, Kousuke; Kurono, Yuichi (2009): Polymyositis with dysphagia treated with endoscopic balloon dilatation. In *Auris, nasus, larynx* 36 (6), pp. 705–708. DOI: 10.1016/j.anl.2009.04.007.

Ofori, Emmanuel; Ramai, Daryl; Ona, Mel; Reddy, Madhavi (2017): Paraneoplastic Dermatomyositis Syndrome Presenting as Dysphagia. In *Gastroenterology research* 10 (4), pp. 251–254. DOI: 10.14740/gr841w.

Oh, Terry H.; Brumfield, Kathlyn A.; Hoskin, Tanya L.; Kasperbauer, Jan L.; Basford, Jeffrey R. (2008): Dysphagia in inclusion body myositis: clinical features, management, and clinical outcome. In *American journal of physical medicine & rehabilitation* 87 (11), pp. 883–889. DOI: 10.1097/PHM.0b013e31818a50e2.

Oh, Terry H.; Brumfield, Kathlyn A.; Hoskin, Tanya L.; Stolp, Kathryn A.; Murray, Joseph A.; Bassford, Jeffrey R. (2007): Dysphagia in inflammatory myopathy. Clinical characteristics, treatment strategies, and outcome in 62 patients. In *Mayo Clinic proceedings* 82 (4), pp. 441–447.

O'Hara, J. M.; Szemes, G.; Lowman, R. M. (1967): The esophageal lesions in dermatomyositis. A correlation of radiologic and pathologic findings. In *Radiology* 89 (1), pp. 27–31. DOI: 10.1148/89.1.27.

Olthoff, Arno; Carstens, Per-Ole; Zhang, Shuo; Fintel, Eva von; Friede, Tim; Lotz, Joachim et al. (2016): Evaluation of dysphagia by novel real-time MRI. In *Neurology* 87 (20), pp. 2132–2138. DOI: 10.1212/WNL.0000000000003337.

Otao, Goh; Yamashita, Shu-ichi; Kyoraku, Itaru; Shiomi, Kazutaka; Nakazato, Masamitsu (2007): Dysphagia due to inflammation of oral muscles as the first symptom of dermatomyositis. In *Internal medicine (Tokyo, Japan)* 46 (12), pp. 923–924. DOI: 10.2169/internalmedicine.46.0068.

Paik, Nam-Jong; Kim, Sang Jun; Lee, Ho Jun; Jeon, Jae Yong; Lim, Jae-Young; Han, Tai Ryoon (2008): Movement of the hyoid bone and the epiglottis during swallowing in patients with dysphagia from different etiologies. In *Journal of electromyography and kinesiology : official journal of the International Society of Electrophysiological Kinesiology* 18 (2), pp. 329–335. DOI: 10.1016/j.jelekin.2006.09.011.

Palace, J.; Losseff, N.; Clough, C. (1993): Isolated dysphagia due to polymyositis. In *Muscle & nerve* 16 (6), pp. 680–681.

Peng, Qing-Lin; Shu, Xiao-Ming; Tian, Xiao-Lan; Lu, Xin; Wang, Guo-Chun (2014): Expression of tumor necrosis factor-like weak inducer of apoptosis and fibroblast growth factor-inducible 14 in patients with polymyositis and dermatomyositis. In *Arthritis research & therapy* 16 (1), R26. DOI: 10.1186/ar4454.

Porkodi, R.; Shanmuganandan, K.; Parthiban, M.; Madhavan, Radha; Rajendran, P. (2002): Clinical spectrum of inflammatory myositis in South India--a ten year study. In *The Journal of the Association of Physicians of India* 50, pp. 1255–1258.

Porubsky, E. S.; Murray, J. P.; Pratt, L. L. (1973): Cricopharyngeal achalasia in dermatomyositis. In *Archives of otolaryngology (Chicago, Ill. : 1960)* 98 (6), pp. 428–429. DOI: 10.1001/archotol.1973.00780020442018.

Ramachandran, Ramesh B.; Swash, Michael (2004): Pharyngeal Dysphagia in dermatomyositis: responsive to cyclophosphamide. In *Journal of clinical neuromuscular disease* 5 (3), pp. 166–167. DOI: 10.1097/00131402-200403000-00008.

Riminton, D. S.; Chambers, S. T.; Parkin, P. J.; Pollock, M.; Donaldson, I. M. (1993): Inclusion body myositis presenting solely as dysphagia. In *Neurology* 43 (6), pp. 1241–1243.

Ryan, Aisling; Nor, Azli M.; Costigan, Donal; Foley-Nolan, Daragh; El-Rafie, Ahmed; Farrell, Michael A.; Hardiman, Orla (2003): Polymyositis masquerading as motor neuron disease. In *Archives of neurology* 60 (7), pp. 1001–1003. DOI: 10.1001/archneur.60.7.1001.

Schrey, Aleksi; Airas, Laura; Jokela, Manu; Pulkkinen, Jaakko (2017): Botulinum toxin alleviates dysphagia of patients with inclusion body myositis. In *Journal of the neurological sciences* 380, pp. 142–147. DOI: 10.1016/j.jns.2017.07.031.

Shapiro, J.; Martin, S.; DeGirolami, U.; Goyal, R. (1996): Inflammatory myopathy causing pharyngeal dysphagia. A new entity. In *The Annals of otology, rhinology, and laryngology* 105 (5), pp. 331–335. DOI: 10.1177/000348949610500501.

Shibata, Saori; Izumi, Rumiko; Hara, Tomonori; Ohshima, Ryuji; Nakamura, Naoko; Suzuki, Naoki et al. (2017): Five-year history of dysphagia as a sole initial symptom in inclusion body myositis. In *Journal of the neurological sciences* 381, pp. 325–327. DOI: 10.1016/j.jns.2017.09.014.

St Guily, J. L.; Perie, S.; Willig, T. N.; Chaussade, S.; Eymard, B.; Angelard, B. (1994): Swallowing disorders in muscular diseases: functional assessment and indications of cricopharyngeal myotomy. In *Ear, nose, & throat journal* 73 (1), pp. 34–40.

STEVENS, M. B.; HOOKMAN, P.; SIEGEL, C. I.; ESTERLY, J. R.; SHULMAN, L. E.; HENDRIX, T. R. (1964): APERISTALSIS OF THE ESOPHAGUS IN PATIENTS WITH CONNECTIVE-TISSUE DISORDERS AND RAYNAUD'S PHENOMENON. In *The New England journal of medicine* 270, pp. 1218–1222. DOI: 10.1056/NEJM196406042702303.

Takamiya, Motonori; Takahashi, Yoshiaki; Morimoto, Mizuki; Morimoto, Nobutoshi; Yamashita, Satoshi; Abe, Koji (2019): Effect of intravenous immunoglobulin therapy on anti-NT5C1A antibody-positive inclusion body myositis after successful treatment of hepatitis C: A case report. In *eNeurologicalSci* 16, p. 100204. DOI: 10.1016/j.ensci.2019.100204.

Tang, Yurong; Xiong, Wenjie; Yu, Ting; Wang, Meifeng; Zhang, Guoxin; Lin, Lin (2018): Eosinophilic Esophageal Myositis a Plausible Cause of Histological Changes of Primary Jackhammer Esophagus: A Case Report. In *The American journal of gastroenterology* 113 (1), pp. 150–152. DOI: 10.1038/ajg.2017.433.

Thomas, F. B.; LeBauer, S.; Greenberger, N. J. (1972): Polymyositis masquerading as carcinoma of the cervical esophagus. In *Archives of internal medicine* 129 (6), pp. 984–986.

Tierney, D.; Jirjis, J. N. (1997): A woman with difficulty swallowing. In *Tennessee medicine : journal of the Tennessee Medical Association* 90 (11), pp. 462–463.

Uthman, I.; Vazquez-Abad, D.; Senecal, J. L. (1996): Distinctive features of idiopathic inflammatory myopathies in French Canadians. In *Seminars in arthritis and rheumatism* 26 (1), pp. 447–458. DOI: 10.1016/s0049-0172(96)80025-4.

Vencovsky, J.; Rehak, F.; Pafko, P.; Jirasek, A.; Valesova, M.; Alusik, S.; Trnavsky, K. (1988): Acute cricopharyngeal obstruction in dermatomyositis. In *The Journal of rheumatology* 15 (6), pp. 1016–1018.

Verma, A.; Bradley, W. G.; Adesina, A. M.; Sofferman, R.; Pendlebury, W. W. (1991): Inclusion body myositis with cricopharyngeus muscle involvement and severe dysphagia. In *Muscle & nerve* 14 (5), pp. 470–473. DOI: 10.1002/mus.880140514.

Wanamaker, J. R.; Wanamaker, H. H.; Lavertu, P. (1992): Polymyositis presenting as a neck mass. In *Archives of otolaryngology--head & neck surgery* 118 (3), pp. 318–320. DOI: 10.1001/archotol.1992.01880030108022.

Wang, S. J.; Lin, W. Y.; Hsu, C. Y.; Kao, C. H.; Chang, C. P.; Lan, J. L. (1993): Solid phase radionuclide esophageal motility in polymyositis and dermatomyositis. In *Gaoxiong yi xue ke xue za zhi = The Kaohsiung journal of medical sciences* 9 (6), pp. 338–342.

Wenzel, J.; Uerlich, M.; Gerdsen, R.; Bieber, T.; Boehm, I. (2001): Association of inclusion body myositis with subacute cutaneous lupus erythematosus. In *Rheumatology international* 21 (2), pp. 75–77. DOI: 10.1007/s002960100137.

Williams, R. B.; Grehan, M. J.; Hersch, M.; Andre, J.; Cook, I. J. (2003): Biomechanics, diagnosis, and treatment outcome in inflammatory myopathy presenting as oropharyngeal dysphagia. In *Gut* 52 (4), pp. 471–478.

Wintzen, A. R.; Bots, G. T.; Bakker, H. M. de; Hulshof, J. H.; Padberg, G. W. (1988): Dysphagia in inclusion body myositis. In *Journal of neurology, neurosurgery, and psychiatry* 51 (12), pp. 1542–1545.

WOLMAN, L.; DARKE, C. S.; YOUNG, A. (1965): THE LARYNX IN RHEUMATOID ARTHRITIS. In *The Journal of laryngology and otology* 79, pp. 403–434. DOI: 10.1017/s0022215100063866.

Yin, Liguo; Ge, Yongpeng; Yang, Hanbo; Peng, Qinglin; Lu, Xin; Zhang, Yamei; Wang, Guochun (2016): The clinical utility of serum IL-35 in patients with polymyositis and dermatomyositis. In *Clinical rheumatology* 35 (11), pp. 2715–2721. DOI: 10.1007/s10067-016-3347-1.

Zuber, M. A.; Kouba, M.; Rudolph, S. E.; Weller, M.; Hrdlicka, P. (2013): Severe dysphagia and erythrodermia in a 59-year-old man. In *Der Internist* 54 (3), pp. 359–365. DOI: 10.1007/s00108-012-3225-0.
