# Supplementary material for: The Impact of Dysphagia in Myositis: A Systematic Review and Meta-Analysis
Source: J Clin Med. 2020 Jul 8;9(7):2150. doi: 10.3390/jcm9072150 (PMC7408750; doi:10.3390/jcm9072150)
Supplement: Supplementary file 1 [file jcm-09-02150-s001.zip › jcm-843942-SI-conversion/supplements/Table S3.docx]

**Table S3:** Studies reporting on outcome of dysphagia in myositis. IIM: idiopathic inflammatory myopathy, DM: dermatomyositis, PM: polymyositis, IBM: inclusion body myositis, JDM: juvenile dermatomyositis, CADM: clinically amyopathic dermatomyositis, VFSS: videofluoroscopy, FEES: flexible endoscopic evaluation of swallowing, EGD: esophagogastroduodenoscopy, PEG: percutaneous endoscopic gastrostomy, VAS: Visual Analog Scale, MMT-8: Manual Muscle Testing 8-Scale, HAQ-Di: Health assessment questionnaire disability index, IVIG: intravenous immunoglobulin

| Author | Study design | Cohort | Definition/ assessment of dysphagia | Outcome of dysphagia |
| --- | --- | --- | --- | --- |
| (Azuma et al. 2011) | retrospective observational study | PM/DM/CADM with malignancy, n=23  PM/DM/CADM without malignancy, n=113 | clinically: difficulty in swallowing water or food,  or the requirement for a nasogastric tube | dysphagia was independent predictive factors for developing malignancies |
| (Benbassat et al. 1985) | retrospective observational study | PM and DM, n=92 | recorded in patient history | dysphagia was associated with a significantly reduced actuarial survival  dysphagia was identified as independent risk factor for mortality |
| (Capkun et al. 2017) | retrospective observational study | sIBM, n=333 | not further stated | rate of aspiration pneumonia at diagnosis during the study: 1%  rate of aspiration pneumonia 12 months after diagnosis: 2% |
| (Carpenter et al. 1977) | retrospective observational study | PM, n=60 | not further stated | 32 % of patients with dysphagia vs. 9% of patients without dysphagia died within one year, this difference was statistically significant  36% of the patients who died, died of bronchopneumonia, all of these patients had dysphagia |
| (Challa et al. 2018) | retrospective observational study | JDM, n=65 | not further stated | dysphagia/dysphonia predicted worsening (or less improvement) in the Childhood Myositis Assessment Scale |
| (Chwalinska-Sadowska and Maldykowa 1990a) | retrospective observational study | PM, n=17  DM, n=33 | not further stated | 4 patients died (in the total cohort with and without dysphagia), one patient died of bronchopneumonia.  prevalence of dysphagia in good and poor prognostic group was not significantly different. |
| (Chwalinska-Sadowska and Maldykowa 1990b) | retrospective observational study | PM, n=17  DM, n=33 | not further stated | signs for bronchopneumonia in x-ray in 12 % of patients in the total cohort with and without dysphagia. |
| (Danko et al. 2004) | prospective observational study | PM, n=75  DM, n=42 | clinical history of disordered swallowing, requirement for nasogastric tube | PM patients with dysphagia had significantly worse cumulative survival probability than PM patients without dysphagia |
| (Degos et al. 1971) | retrospective observational study | DM, n=31 | not further stated | 6% (1/16) had inhalation pneumonia |
| (Galindo-Feria et al. 2016) | retrospective observational study | DM without JDM, n=264  PM, n=69 | not further stated | prevalence of dysphagia was not significantly different between patients who died and patients who survived in DM and PM |
| (Hajialilo et al. 2018) | retrospective observational study | PM and DM, n=76 | not further stated | prevalence of dysphagia was significantly higher in the patients not responding to therapy compared to patients responding to therapy |
| (Hochberg et al. 1986) | retrospective observational study | PM, n=31  DM, n=21  Cancer-associated IIM, n=6  overlap syndrome, n=18 | not further stated | overall case fatality ratios and cumulative survival rates did not differ between groups with and without dysphagia. |
| (Houser et al. 1998) | retrospective and prospective observational study | IBM, n=22 | chart documentation and telephone interview | patients with progressive dysphagia had a significantly worse functional class rating than their non-progressing counterparts (measure of the ability to provide self-care and to engage in normal day-to-day activities) |
| (Kobayashi et al. 1997) | retrospective observational study | JDM, n=102 | not further stated | the incidence of dysphagia was higher in the poor prognosis group than in the good prognosis group of JDM.  the incidence of dysphagia was significantly higher in the patients with Brunsting-type JDM who died (100%) than in the surviving patients with Brunsting-type JDM (8%) |
| (Lakhanpal et al. 1987) | retrospective observational study | Autopsies of patients with PM (n=24) and DM (n=41) | not further stated | 54% of patients with pneumonia in autopsy had a history of dysphagia, vs. 50% of patients without pneumonia  aspiration pneumonia was identified in 6 patients in autopsy, 5 of whom had a history of dysphagia |
| (Lilleker et al. 2018) | retrospective observational register-study | IIM, n=3067  DM, n=949  PM, n=813  IBM, n=240  ASS, n=512  JDM, n=90  connected tissue disease-overlap, n=358 | not further stated | MMT-8-score was significantly lower, global disease activity VAS was significantly higher, HAQ-Di was significantly higher, extramuscular disease activity VAS was significantly higher and myositis damage VAS was significantly higher in patients with dysphagia |
| (Marie et al. 2002) | retrospective observational study | PM/DM, n=156 | esophageal dysfunction but not further stated how evaluated | aspiration pneumonia: 17% (but unclear if in all cases dysphagia was confirmed) |
| (Marie et al. 2001) | retrospective observational study | PM/DM, n=77 | manometric esophageal impairment | 17% of all patients had aspiration pneumonia  30% of the patients who died, died of aspiration pneumonia (second most common cause of death after malignancy)  aspiration pneumonia was significantly more frequent in the group with deterioration and death compared to the group without deterioration and death  esophageal dysfunction was not significantly different in the group with deterioration and death compared to the group without deterioration and death |
| (Marie et al. 1999) | retrospective observational study | DM/PM, n=79  DM/PM > 65 years, n=23  DM/PM < 65 years, n= | esophageal manometry | mortality in patients > 65 was 48%, 36% of these cases were attributed to bacterial pneumonia  mortality in patients < 65 was 7%, 25% of these cases were attributed to bacterial pneumonia |
| (Marie et al. 2010) | retrospective observational study | PM/DM, n=301 | esophageal impairment defined by clinical manifestation in combination with abnormal finding in esophageal manometry and exclusion of other causes in gastroscopy | patients with esophageal impairment compared to those without significantly more frequently exhibited muscle weakness (95% vs. 83%), ventilatory insufficiency (35% vs. 10%), and aspiration pneumonia (36% vs. 9%)  among the 8 patients who deteriorated under IVIG therapy, 6 died within one month from aspiration pneumonia |
| (Maugars et al. 1996) | retrospective observational study | IIM, n=69  PM, n=21  DM, n=48 | not further stated | 47% of patients who died had dysphagia, 28% of patients who survived had dysphagia, this difference was not significant.  The absence of dysphagia was a negative prognostic factor after the role of dysphonia was discerned (probably paradox effect as dysphonia was strongly correlated with pulmonary interstitial fibrosis, and dysphagia was correlated with dysphonia) |
| (McCann et al. 2007) | prospective observational study | JDM, n=14 | VFSS | 23% of patients had weight loss (not clear if all of whom had dysphagia) |
| (Medsger, JR et al. 1971) | retrospective observational study | PM, n=124  N=74 with report on dysphagia | not further stated | cumulative survival rate of patients with pneumonitis on x-ray on admission (n=14) was significantly lower compared to patients without pneumonitis, the most common etiology of pneumonitis was aspiration (n=8), cumulative survival rate of patients with dysphagia on admission was significantly lower compared to patients without dysphagia, all patients with aspiration pneumonia had moderate or severe dysphagia. |
| (Merieux et al. 1983) | prospective and retrospective observational study | PM or DM in the prospective cohort, n=16  autopsy of patients with PM or DM, n=18 | clinical symptoms and VFSS  barium swallow with cine esogram | 22% aspiration pneumonia in the patients with clinical dysphagia  14% aspiration pneumonia in the patients with abnormal VFSS (all abnormalities included) |
| (Ogawa-Momohara et al. 2019) | retrospective observational study | DM, n=85 | symptoms according to physician, otolaryngologist or speech language pathologist | 23% of the dysphagic patients who died, died of aspiration pneumonia  survival rate was associated with dysphagia recovery (94% in patients with recovery, 8% in patients without recovery) |
| (Oh et al. 2008) | retrospective observational study | IBM with Dysphagia, n=26 | partly clinical dysphagia evaluation (n=24), VFSS (n=23), and pharyngoesophageal  manometry (n=12) | 13 patients died during follow-up. The cause of death was identified in 8 patients: in all cases complication of aspiration |
| (Oh et al. 2007) | retrospective observational study | IIM with dysphagia, n=62 | partly VFSS (n=38) | 16% history of pneumonia  15% history of weight loss  patients with IBM had least improvement in dysphagia  respiratory failure in association with aspiration pneumonia was the most common cause of death (45%)  mortality was high in patients who required PEG (64%) |
| (Olthoff et al. 2016) | prospective observational study | IBM without severe aspiration according to patient history, n=20 | VFSS  FEES  Real-time-MRI | correlation of pharyngeal retention with swallowing quality of life questionnaire |
| (Peng et al. 2000) | retrospective observational study | sIBM, n=78 | not further stated | aspiration pneumonia was the most common cause of death (in 33% of the patients who died) |
| (Porkodi et al. 2002) | observational study (unclear if retrospective or prospective) | IIM, n=87  PM, n=24  DM, n=28  DM with malignancy, n=1  JDM/JPM, n=5  Overlap syndrome, n=30 | not further stated | aspiration pneumonia in the total cohort: 2% |
| (Price et al. 2016) | retrospective, survey based observational study among physicians | data based on 585 living and 149 deceased patients with sIBM | not further stated | 23% of patients were reported to have experienced aspiration pneumonia  12 of the 13 physicians agreed that patients with bulbar dysfunction (abnormal swallowing and speech), dysphagia and oropharyngeal involvement had a shortened lifespan.  10 of the 13 physicians reported that patients with sIBM had a shortened lifespan compared with the general population, a majority of whom (8/10) thought this was only in a subset of patients, primarily those experiencing severe dysphagia.  physicians ranked oropharyngeal muscle dysfunction as the most common factor that could have contributed to the death of the deceased patients |
| (Schrey et al. 2017) | retrospective observational study | sIBM, n=40  sIBM with neurotoxin A injection-therapy (BoNT-A), n=12 | VFSS  EGD | Before BoNT-A, 58% of patients had aspiration and 25% had aspiration pneumonia. After BoNT-A, none of the patients experienced aspiration or aspiration pneumonia for at least six months |
| (Suzuki et al. 2015) | retrospective observational study | anti-SRP-positive IIM, n=100 | not further stated | prevalence of dysphagia in patients with a poor outcome (modified Rankin Scale scores 3–5) was 63%. Prevalence of dysphagia in patients with good outcome was 34%. Significantly higher in patients with poor outcome |
| (Williams et al. 2003) | retrospective observational study | IIM with oropharyngeal dysphagia, n=13 | oropharyngeal dysphagia was defined as difficulty with the act of swallowing together with one or more of the following deglutitive symptoms: bolus holdup; multiple swallows required to clear the pharynx; deglutitive coughing and/or choking; or postnasal regurgitation  partly VFSS and manometry | overall 12 month mortality: 31%  aspiration pneumonia: 23% (whereas 67% of these patients died, was the leading cause of death) |

Publication bibliography

Azuma, Kohei; Yamada, Hidehiro; Ohkubo, Michiko; Yamasaki, Yoshioki; Yamasaki, Masaomi; Mizushima, Machiko; Ozaki, Shoichi (2011): Incidence and predictive factors for malignancies in 136 Japanese patients with dermatomyositis, polymyositis and clinically amyopathic dermatomyositis. In *Modern rheumatology* 21 (2), pp. 178–183. DOI: 10.1007/s10165-010-0362-y.

Benbassat, J.; Gefel, D.; Larholt, K.; Sukenik, S.; Morgenstern, V.; Zlotnick, A. (1985): Prognostic factors in polymyositis/dermatomyositis. A computer-assisted analysis of ninety-two cases. In *Arthritis and rheumatism* 28 (3), pp. 249–255. DOI: 10.1002/art.1780280303.

Capkun, Gorana; Callan, Aoife; Tian, Haijun; Wei, Zhongyuan; Zhao, Changgeng; Agashivala, Neetu; Barghout, Victoria (2017): Burden of illness and healthcare resource use in United States patients with sporadic inclusion body myositis. In *Muscle & nerve* 56 (5), pp. 861–867. DOI: 10.1002/mus.25686.

Carpenter, J. R.; Bunch, T. W.; Engel, A. G.; O'Brien, P. C. (1977): Survival in polymyositis: corticosteroids and risk factors. In *The Journal of rheumatology* 4 (2), pp. 207–214.

Challa, Divya; Crowson, Cynthia S.; Niewold, Timothy B.; Reed, Ann M. (2018): Predictors of changes in disease activity among children with juvenile dermatomyositis enrolled in the Childhood Arthritis and Rheumatology Research Alliance (CARRA) Legacy Registry. In *Clinical rheumatology* 37 (4), pp. 1011–1015. DOI: 10.1007/s10067-017-3901-5.

Chwalinska-Sadowska, H.; Maldykowa, H. (1990a): Polymyositis-dermatomyositis:25 years of follow-up of 50 patients disease course, treatment, prognostic factors. In *Materia medica Polona. Polish journal of medicine and pharmacy* 22 (3), pp. 213–218.

Chwalinska-Sadowska, H.; Maldykowa, H. (1990b): Polymyositis-dermatomyositis--a 25-year follow-up of 50 patients (analysis of clinical symptoms and signs and results of laboratory tests). In *Materia medica Polona. Polish journal of medicine and pharmacy* 22 (3), pp. 205–212.

Danko, Katalin; Ponyi, Andrea; Constantin, Tamas; Borgulya, Gabor; Szegedi, Gyula (2004): Long-term survival of patients with idiopathic inflammatory myopathies according to clinical features: a longitudinal study of 162 cases. In *Medicine* 83 (1), pp. 35–42. DOI: 10.1097/01.md.0000109755.65914.5e.

Degos, R.; Civatte, J.; Belaich, S.; Delarue, A. (1971): The prognosis of adult dermatomyositis. In *Transactions of the St. John's Hospital Dermatological Society* 57 (1), pp. 98–104.

Galindo-Feria, Angeles Shunashy; Rojas-Serrano, Jorge; Hinojosa-Azaola, Andrea (2016): Clinical and Prognostic Factors Associated With Survival in Mexican Patients With Idiopathic Inflammatory Myopathies. In *Journal of clinical rheumatology : practical reports on rheumatic & musculoskeletal diseases* 22 (2), pp. 51–56. DOI: 10.1097/RHU.0000000000000365.

Hajialilo, Mehrzad; Ghorbanihaghjo, Amir; Khabbazi, Alireza; Kolahi, Sousan; Jafari Nakhjavani, Mohammad Reza; Ebrahimi, Ali Asghar et al. (2018): Long-term follow-up of 76 Iranian patients with idiopathic inflammatory myopathies. In *International journal of rheumatic diseases* 21 (8), pp. 1627–1633. DOI: 10.1111/1756-185X.13352.

Hochberg, M. C.; Feldman, D.; STEVENS, M. B. (1986): Adult onset polymyositis/dermatomyositis: an analysis of clinical and laboratory features and survival in 76 patients with a review of the literature. In *Seminars in arthritis and rheumatism* 15 (3), pp. 168–178. DOI: 10.1016/0049-0172(86)90014-4.

Houser, S. M.; Calabrese, L. H.; Strome, M. (1998): Dysphagia in patients with inclusion body myositis. In *The Laryngoscope* 108 (7), pp. 1001–1005. DOI: 10.1097/00005537-199807000-00009.

Kobayashi, S.; Higuchi, K.; Tamaki, H.; Wada, Y.; Wada, N.; Kubo, M. et al. (1997): Characteristics of juvenile dermatomyositis in Japan. In *Acta paediatrica Japonica : Overseas edition* 39 (2), pp. 257–262. DOI: 10.1111/j.1442-200x.1997.tb03595.x.

Lakhanpal, S.; Lie, J. T.; Conn, D. L.; Martin, W. J. 2nd (1987): Pulmonary disease in polymyositis/dermatomyositis: a clinicopathological analysis of 65 autopsy cases. In *Annals of the rheumatic diseases* 46 (1), pp. 23–29. DOI: 10.1136/ard.46.1.23.

Lilleker, James B.; Vencovsky, Jiri; Wang, Guochun; Wedderburn, Lucy R.; Diederichsen, Louise Pyndt; Schmidt, Jens et al. (2018): The EuroMyositis registry: an international collaborative tool to facilitate myositis research. In *Annals of the rheumatic diseases* 77 (1), pp. 30–39. DOI: 10.1136/annrheumdis-2017-211868.

Marie, I.; Hachulla, E.; Cherin, P.; Dominique, S.; Hatron, P-Y; Hellot, M-F et al. (2002): Interstitial lung disease in polymyositis and dermatomyositis. In *Arthritis and rheumatism* 47 (6), pp. 614–622. DOI: 10.1002/art.10794.

Marie, I.; Hachulla, E.; Hatron, P. Y.; Hellot, M. F.; Levesque, H.; Devulder, B.; Courtois, H. (2001): Polymyositis and dermatomyositis: short term and longterm outcome, and predictive factors of prognosis. In *The Journal of rheumatology* 28 (10), pp. 2230–2237.

Marie, I.; Hatron, P. Y.; Levesque, H.; Hachulla, E.; Hellot, M. F.; Michon-Pasturel, U. et al. (1999): Influence of age on characteristics of polymyositis and dermatomyositis in adults. In *Medicine* 78 (3), pp. 139–147. DOI: 10.1097/00005792-199905000-00001.

Marie, I.; Menard, J-F; Hatron, P. Y.; Hachulla, E.; Mouthon, L.; Tiev, K. et al. (2010): Intravenous immunoglobulins for steroid-refractory esophageal involvement related to polymyositis and dermatomyositis: a series of 73 patients. In *Arthritis care & research* 62 (12), pp. 1748–1755. DOI: 10.1002/acr.20325.

Maugars, Y. M.; Berthelot, J. M.; Abbas, A. A.; Mussini, J. M.; Nguyen, J. M.; Prost, A. M. (1996): Long-term prognosis of 69 patients with dermatomyositis or polymyositis. In *Clinical and experimental rheumatology* 14 (3), pp. 263–274.

McCann, L. J.; Garay, S. M.; Ryan, M. M.; Harris, R.; Riley, P.; Pilkington, C. A. (2007): Oropharyngeal dysphagia in juvenile dermatomyositis (JDM): an evaluation of videofluoroscopy swallow study (VFSS) changes in relation to clinical symptoms and objective muscle scores. In *Rheumatology (Oxford, England)* 46 (8), pp. 1363–1366. DOI: 10.1093/rheumatology/kem131.

Medsger, T. A., JR; Robinson, H.; Masi, A. T. (1971): Factors affecting survivorship in polymyositis. A life-table study of 124 patients. In *Arthritis and rheumatism* 14 (2), pp. 249–258. DOI: 10.1002/art.1780140210.

Merieux, P. de; Verity, M. A.; Clements, P. J.; Paulus, H. E. (1983): Esophageal abnormalities and dysphagia in polymyositis and dermatomyositis. In *Arthritis and rheumatism* 26 (8), pp. 961–968. DOI: 10.1002/art.1780260804.

Ogawa-Momohara, Mariko; Muro, Yoshinao; Kono, Michihiro; Akiyama, Masashi (2019): Prognosis of dysphagia in dermatomyositis. In *Clinical and experimental rheumatology* 37 (1), p. 165.

Oh, Terry H.; Brumfield, Kathlyn A.; Hoskin, Tanya L.; Kasperbauer, Jan L.; Basford, Jeffrey R. (2008): Dysphagia in inclusion body myositis: clinical features, management, and clinical outcome. In *American journal of physical medicine & rehabilitation* 87 (11), pp. 883–889. DOI: 10.1097/PHM.0b013e31818a50e2.

Oh, Terry H.; Brumfield, Kathlyn A.; Hoskin, Tanya L.; Stolp, Kathryn A.; Murray, Joseph A.; Bassford, Jeffrey R. (2007): Dysphagia in inflammatory myopathy. Clinical characteristics, treatment strategies, and outcome in 62 patients. In *Mayo Clinic proceedings* 82 (4), pp. 441–447.

Olthoff, Arno; Carstens, Per-Ole; Zhang, Shuo; Fintel, Eva von; Friede, Tim; Lotz, Joachim et al. (2016): Evaluation of dysphagia by novel real-time MRI. In *Neurology* 87 (20), pp. 2132–2138. DOI: 10.1212/WNL.0000000000003337.

Peng, A.; Koffman, B. M.; Malley, J. D.; Dalakas, M. C. (2000): Disease progression in sporadic inclusion body myositis: observations in 78 patients. In *Neurology* 55 (2), pp. 296–298. DOI: 10.1212/wnl.55.2.296.

Porkodi, R.; Shanmuganandan, K.; Parthiban, M.; Madhavan, Radha; Rajendran, P. (2002): Clinical spectrum of inflammatory myositis in South India--a ten year study. In *The Journal of the Association of Physicians of India* 50, pp. 1255–1258.

Price, Mark A.; Barghout, Victoria; Benveniste, Olivier; Christopher-Stine, Lisa; Corbett, Alastair; Visser, Marianne de et al. (2016): Mortality and Causes of Death in Patients with Sporadic Inclusion Body Myositis: Survey Study Based on the Clinical Experience of Specialists in Australia, Europe and the USA. In *Journal of neuromuscular diseases* 3 (1), pp. 67–75. DOI: 10.3233/JND-150138.

Schrey, Aleksi; Airas, Laura; Jokela, Manu; Pulkkinen, Jaakko (2017): Botulinum toxin alleviates dysphagia of patients with inclusion body myositis. In *Journal of the neurological sciences* 380, pp. 142–147. DOI: 10.1016/j.jns.2017.07.031.

Suzuki, Shigeaki; Nishikawa, Atsuko; Kuwana, Masataka; Nishimura, Hiroaki; Watanabe, Yurika; Nakahara, Jin et al. (2015): Inflammatory myopathy with anti-signal recognition particle antibodies: case series of 100 patients. In *Orphanet journal of rare diseases* 10, p. 61. DOI: 10.1186/s13023-015-0277-y.

Williams, R. B.; Grehan, M. J.; Hersch, M.; Andre, J.; Cook, I. J. (2003): Biomechanics, diagnosis, and treatment outcome in inflammatory myopathy presenting as oropharyngeal dysphagia. In *Gut* 52 (4), pp. 471–478.
