# Supplementary material for: The Impact of Dysphagia in Myositis: A Systematic Review and Meta-Analysis
Source: J Clin Med. 2020 Jul 8;9(7):2150. doi: 10.3390/jcm9072150 (PMC7408750; doi:10.3390/jcm9072150)
Supplement: Supplementary file 1 [file jcm-09-02150-s001.zip › jcm-843942-SI-conversion/supplements/Table S4.docx]

**Table S4:** Studies reporting on therapy of dysphagia in myositis. IIM: idiopathic inflammatory myopathy, DM: dermatomyositis, PM: polymyositis, IBM: inclusion body myositis, JDM: juvenile dermatomyositis, VFSS: videofluoroscopy, FEES: flexible endoscopic evaluation of swallowing, HRM: high resolution manometry, EGD: esophagogastroduodenoscopy, PEG: percutaneous endoscopic gastrostomy, IVIG: intravenous immunoglobulin, EMG: electromyography, PAS: penetration aspiration scale, UES: upper esophageal sphincter, POEM: peroral endoscopic myotomy

| Author | Study design | Cohort | Definition/ assessment of dysphagia | Therapy response of dysphagia |
| --- | --- | --- | --- | --- |
| (Al-Mayouf et al. 2000) | prospective, non-randomized intervention study | severe JDM, n=12 | not further stated | dysphagia resolved in 3 patients after intravenous methylprednisolone, oral prednisolone and methotrexate |
| (Almodovar et al. 2012) | case-report | DM, n=1 | barium study of the esophagus, stomach, and duodenum | Subjective improvement of symptoms after treatment with prednisone |
| (Alqatari et al. 2018) | case-report | MDA-5-positive DM, n=1 | not reported | IVIG and cyclophosphamide led to a short-term improvement of symptoms |
| (Andonopoulos et al. 1993) | case-report | PM, n=1 | not further stated | improvement of symptoms after antibiotics and parenteral fluids and methylprednisolone |
| (Bachmann et al. 2001) | case-report | IIM, n=1 | laryngoscopy  VFSS  manometry | botulinum toxin-a-injection into the cricopharyngeus muscle reduced the maximal and resting pressure of the cricopharyngeus muscle but did not improve symptoms. Symptoms and VFSS findings improved after myotomy of the cricopharyngeus muscle |
| (Barrera et al. 1998) | case-report | PM associated with systemic sclerosis, n=1 | gastrointes-tinal series (probably VFSS) | improvement of symptoms after therapy with oral prednisone and methotrexate |
| (Basnayake et al. 2015) | retrospective observational study | anti-SRP-positive IIM, n=5 | clinical symptoms and weight loss | dysphagia resolved after oral prednisolone, methotrexate and subsequently intravenous IVIG therapy in one patient |
| (Black and Marshman 2011) | case-report | DM, n=1 | not further stated | improvement of symptoms after oral prednisolone |
| (Bonin et al. 2010) | case-report | PM with mixed connective tissue disease, n=1 | not further stated | intravenous therapy with methylprednisolone resulted in clinical improvement |
| (Butt et al. 2017) | case-report | NXP-2-positive DM, n=1 | barium-swallow | IVIG, pulse steroids and prednisone led to an improvement of symptoms |
| (Camargo et al. 2018) | prospective observational study | sIBM, n=18 | not further stated | symptomatic improvement after intravenous methylprednisolone (lasting less than 12 month) |
| (Caramaschi et al. 1997) | case-report | DM, n=1 | esophageal radiographic examination  endoscopic examination of the upper gastro-intestinal tract  biopsy of the esophageal mucosa  esophageal manometry | resolution of symptoms after treatment with omeprazole |
| (Chen et al. 2014) | case-report | IIM, n=1 | clinically e.g. choking when drinking | oral prednisolone improved clinical symptoms |
| (Cherin et al. 2002) | case-series | IBM, n=4 | tube dependent  esophageal manometry | resolution of symptoms and abnormality in manometry after administration of IVIG alone or in association with corticosteroids |
| (Cherin et al. 2015) | case-series | IBM, n=6 | not further stated | dysphagia resolved in all of the cases after subcutaneous immunoglobulin therapy |
| (Chinniah and Mody 2017) | case-report | IIM with systemic sclerosis overlap, n=1 | PEG-tube dependent | methylprednisolone, azathioprine, two courses of IVIG and rituximab led to remission of symptoms (>20 months) |
| (Cnop et al. 2019) | case-report | DM, n=1 | not further stated | improvement after methylprednisolone pulse, methotrexate, hydroxychloroquine and prednisone |
| (Cochicho et al. 2016) | case-report | PM, n=1 | barium swallow, esophageal manometry | prednisolone therapy led to clinical long-term improvement |
| (Dagan et al. 2013) | case-report | DM, n=1 | VFSS | after therapy with intravenous methylprednisolone, intramuscular methotrexate, intravenous IVIG and oral prednisolone dysphagia resolved |
| (Dalakas et al. 1997) | double-blind, placebo-controlled study with cross-over design | IBM, n=19 | ultrasound of swallowing | in the placebo randomized group, the duration of both the dry and wet swallows increased but decreased significantly when crossed to IVIG. In the IVIG-randomized patients the duration of the swallows significantly decreased compared with baseline. |
| (Danon and Friedman 1989) | case-report | IBM, n=1 | oropharyn-geal swallow study | symptoms improved after myotomy |
| (Darrow et al. 1992) | case-report | IBM, n=1 | VFSS | improvement of symptoms and VFSS findings after myotomy |
| (Di Pede et al. 2016) | case-series | IBM, n=4 | VFSS, FEES, HRM | decrease of mean PAS from 6.25 to 3.25, 6 months after percutaneous botulinum neuro toxin type A (BoNT/A) injection in the cricopharyngeal muscle, this improvement remained in the long-term with repetitive treatment. |
| (Dias et al. 2015) | case-report | DM associated with breast cancer, n=1 | not further stated | dysphagia resolved after surgical dissection of the malignant tumor |
| (Dietz et al. 1980) | case-report | PM, n=1 | VFSS | improvement of symptoms and VFSS findings after myotomy |
| (Dobloug et al. 2012) | retrospective observational study | sIBM, n=14; n=6 | subjective report of patients n=6  radiological dynamic studies of the esophagus n=14 | 3 of the IVIG treated patients reported subjective improvement  on swallowing function during follow-up compared to none in the control  group with different medications. |
| (Ertekin et al. 1996) | prospective observational study | DM/PM, n=7 | tube-dependent  decreased dysphagia limit in EMG | increase in dysphagia limit reported in one patient after corticosteroid treatment |
| (Eura et al. 2015) | case-report | DM, n=1 | VFSS | oral prednisolone improved clinical symptoms and VFSS-findings |
| (Gameiro et al. 2018) | case-report | U1-RNP positive IIM with mixed connective tissue disease, N=1 | tube-dependent  HRM | IVIG led to long term improvement in HRM (>9 months) |
| (Giannini et al. 2018) | case-report | anti-PL12-ASS-and sIBM overlap, n=1 | FEES | IVIG and Rituximab: Improvement of FEES-findings. |
| (Gibson et al. 1991) | case-report | IBM with mixed connected tissue disease, n=1 | not further stated | symptoms improved after oral prednisolone therapy |
| (Gyorffy et al. 2018) | case-report | mixed connective tissue disease, n=1 | barium swallow, EGD, HRM | prednisolone, IVIG, mycophenolate mofetil and hydroxychloroquine led to a long-term improvement (>6months, symptoms and HRM) |
| (Hafejee and Coulson 2005) | case-report | DM associated with transitional cell carcinoma of the bladder, n=1 | VFSS | dysphagia in VFSS improved (safe swallowing without aspiration) after intravenous methylprednisolone, IVIG and oral prednisolone |
| (Hirano et al. 1993) | case-report | PM, n=1 | not further stated | improvement of symptoms after intravenous pulse cyclophosphamide and low-dose oral methotrexate therapy |
| (Houser et al. 1998) | retrospective and prospective observational study | IBM, n=22 | chart documen-tation and telephone interview | improvement of symptoms after myotomy in a single case reported |
| (Iannone et al. 2015) | case-report | DM, n=1 | barium swallow | high-dose IVIG therapy led to the resolving of dysphagia (clinical symptoms and on barium swallow) |
| (Joshi et al. 2008) | case-report | DM, n=1 | FEES | additional to prednisolone and methotrexate, intravenous IVIG therapy led to a significant improvement of symptoms and dysphagia resolved in the long-term |
| (Kagen et al. 1985) | case-series | PM and DM, n=3 | VFSS  manometry | improvement of symptoms in 2 out of 2 patients and improvement in VFSS findings in 1 out of 1 patient after myotomy |
| (Kwon et al. 2018) | case-report | DM, n=1 | VFSS | oral prednisolone, intravenous methylprednisolone, azathioprine, and IVIG led to a long-term improvement (>3years) |
| (Kwon et al. 2017) | case-report | anti-EJ-positive DM, n=1 | not further stated | prednisone and mycophenolate mofetil led to a resolvement of dysphagia symptoms |
| (Labeit et al. 2019) | case-report | anti-Jo-1-positive IIM, n=1 | FEES, VFSS, HRM | Long-term improvement (> 1 year) of symptoms and FEES findings after intravenous methylprednisolone, prednisolone and azathioprine |
| (Laurikainen et al. 1992) | prospective observational study | IIM, n=1 | VFSS  fiberoptic esopha-goscopy  manometry | improvement of symptoms after myotomy |
| (Lee et al. 2008) | case-report | DM, n=1 | not further stated | improvement of symptoms after IVIG therapy |
| (Liu et al. 2004) | case-series | IBM, n=2 | VFSS  esophageal manometry | improvement of symptoms and decrease of UES pressure in manometry after botulinum toxin A injection into the cricopharyngeus muscle |
| (MacFarlane et al. 2016) | case-report | DM, n=1 | not further stated | dysphagia resolved after subcutaneous injections of methotrexate at a dose of 20 mg weekly (following methylprednisolone pulse and oral prednisolone therapy) |
| (Malandraki et al. 2012) | case-report | IBM, n=1 | VFSS | isometric lingual strengthening was effective in maintaining posterior tongue lingual pressure and penetration-aspiration Scale scores during the treatment periods. Residue scale scores did not significantly change during treatment |
| (Marie et al. 1999) | case-series | PM/DM, n=3 | esophageal manometry | improvement of symptoms and manometry findings after IVIG therapy |
| (Marie et al. 2010) | retrospective observational study | PM/DM, n=301 | esophageal impairment defined by clinical manifes-tation in combination with abnormal finding in esophageal manometry and exclusion of other causes in gastroscopy | 73 patients with steroid-refractory esophageal involvement received IVIG-therapy: 1 gm/kg daily for 2 days each month; the median IVIG therapy duration was 7 months (range 3–14 months).  82% exhibited resolution of their esophageal clinical manifestations. This resulted in the return to normal oral feeding, with normal food consistency and ablation of feeding enteral tubes in all cases. 7% exhibited improvement of the clinical manifestation. 11% exhibited deterioration of esophageal clinical symptoms.  1 patient with impaired cricopharyngeal muscle relaxation additionally received myotomy after which dysphagia resolved. |
| (Matsuoka et al. 2019) | case-report | DM, n=1 | not further stated | dysphagia resolved after therapy with IVIG, intravenous-cyclophosphamide and tacrolimus |
| (Metheny 1978) | case-series | DM, n=2 | indirect laryngoscopy in 1 patient and barium swallow in 1 patient | improvement of symptoms after oral prednisolone |
| (Mii et al. 2006) | case-report | DM, n=1 | swallow x-ray film  tube-dependent | dysphagia resolved after oral cyclosporine A |
| (Milisenda et al. 2014) | case-series | DM with subcutaneous edema, n=5 | not further stated | dysphagia resolved after IVIG therapy in 1 patient |
| (Mugii et al. 2016) | prospective observational study | DM, n=92 | clinical observation by otorhino-laryngologists and speech therapists  partly VFSS | all patients showed clinical improvement of dysphagia after treatment of malignancy (n = 10) and immunomodulatory therapy (corticosteroid n = 13 and IVIG; n = 2) after 7.3±5.5 months follow up. Swallowing rehabilitation and balloon dilatation were concurrently used. |
| (Murata et al. 2013) | case-series | sIBM, n=3 | VFSS  computed pharyngo-esophageal manometry | after intravenous IVIG therapy, the amount of barium paste passing through the entrance of the UES increased. Following both balloon dilatation and IVIG therapy, the amount of barium passing through the entrance of the UES increased. As a consequence, food residues in the piriform sinus were reduced, and aspiration of food residues was alleviated by both therapies. This effect lasted for at least on year. |
| (Nagano et al. 2009) | case-report | PM, n=1 | VFSS | improvement of symptoms and VFSS findings after endoscopic balloon dilation (repeated twice) for 6 months |
| (Nagano et al. 2015) | case-report | DM associated with rectum cancer, n=1 | not further stated | improvement of dysphagia after chemotherapy with modified FOLFOX6 plus bevacizumab (oxaliplatin was removed after the first cycle of modified FOLFOX6 due to thrombocytopenia) |
| (Noda et al. 1988) | case-report | PM, n=1 | tube-dependent | improvement of symptoms after therapy with oral prednisone |
| (Ocampo et al. 2014) | case-report | DM, n=1 | not further stated | improvement of symptoms after intravenous methylprednisolone, oral prednisolone and methotrexate, and IVIG, however the causality of therapy remains unclear. |
| (Ogawa-Momohara et al. 2019) | retrospective observational study | DM, n=85 | symptoms according to physician, otolaryngologist or speech language pathologist | 55% showed an improvement of dysphagia (various therapies), improvement was associated with initial dose of prednisolone |
| (Oh et al. 2008) | retrospective observational study | IBM with Dysphagia, n=26 | partly clinical dysphagia evaluation (n=24), VFSS (n=23), and pharyngo-esophageal  manometry (n=12) | immunosuppressive therapy (n=20):  corticosteroids (n=19), azathioprine (n=10), methotrexate (n=9), mycophenolate mofetil (n=2), IVIG (n=1). 1 patient with definite IBM reported a subjective functional benefit from prednisone and azathioprine over a 6-year period.  behavioral therapy (n=23):  diet modification (n=23), feeding strategies (n=13), compensatory techniques (n=13), and exercises (n=8).  Mendelsohn maneuver (n=3): maintaining oral intake 1–5 years without aspiration related illness or weight loss (no follow-up VFSS).  patients with follow-up VFSS after behavioral therapy (n=3): persistent or increased residual pharyngeal pooling, penetration, and aspiration.  interventional procedure (n=18):  cricopharyngeal myotomy (n=10), follow-up (n=8) revealed that 7 patients noted symptomatic improvement (however no relevant improvement in those with VFSS follow-up).  pharyngoesophageal dilatation (n=6), follow-up (n=6) revealed that 2 patients reported benefit  botulinum injection (n=2), follow up revealed that no patient reported benefit. |
| (Oh et al. 2007) | retrospective observational study | IIM with dysphagia, n=62 | partly VFSS (n=38) | immunosuppressive therapy: agents reported to be beneficial included prednisone in 29 (56%) of 52 patients, methotrexate in 10  (40%) of 25, azathioprine in 7 (24%) of 29, IVIG in 3 (38%) of 8, hydroxychloroquine in 2 (50%) of 4, and other less frequently used agents in 5  (56%) of 9 patients.  interventions: cricopharyngeal myotomy in patients with prominent cricopharyngeal musculature was described as beneficial in 6 of the 7 available follow up reports.  cricopharyngeal dilatation was described as beneficial in 2 of the 7 available follow up reports. |
| (Otao et al. 2007) | case-report | DM, n=1 | not further stated | improvement of symptoms and decrease of high intensity lesions of pharyngeal muscles in MRI after intravenous methylprednisolone  followed by oral prednisolone |
| (Palace et al. 1993) | case-report | PM, n=1 | VFSS | improvement of symptoms after oral prednisolone |
| (Papakonstantinou et al. 2016) | case-report | DM associated with lung adenocarcinoma, n=1 | not further stated | partial improvement after surgical resection of the carcinoma |
| (Pars et al. 2013) | case-report | IBM, n=1 | unintended weight loss | IVIG improved and stabilized dysphagia over a period of two years. Treatment was then changed to subcutaneous applications of immunoglobulines and swallowing function remained stable. |
| (Patrick et al. 2014) | case-report | PM, n=1 | weight loss | dysphagia resolved after recurrent subcutaneous immunoglobulin therapy and behavioral therapy |
| (Porubsky et al. 1973) | case-report | DM, n=1 | VFSS | short term improvement after cricopharyngeal dilatation, long-term improvement after cricopharyngeal myotomy |
| (Prossliner et al. 2016) | case-report | DM, n=1 | not further stated | IVIG (2 g/kg body weight given cumulatively over two to four days at four-week intervals), in combination with intensive physical and speech language therapy improved dysphagia |
| (Raghu et al. 2015) | retrospective observational study | IIM, N=7 | not further stated | pulse dose intravenous methylprednisolone therapy resolved dysphagia in 2 patients and improved symptoms in 3 patients |
| (Ramachandran and Swash 2004) | case-series | DM, n=2 | VFSS | improvement of dysphagia in both cases after intravenous cyclophosphamide therapy (500mg per day pulses) in addition to prednisolone and azathioprine. |
| (Riminton et al. 1993) | case-series | IBM, n=2 | VFSS, manometry, cine barium swallowing study | improvement of symptoms after myotomy |
| (Ryan et al. 2003) | case-report | PM, n=1 | VFSS | long-term resolution (>1year) of dysphagia after high dose corticosteroids and azathioprine |
| (Schrey et al. 2017) | retrospective observational study | sIBM, n=40  sIBM with Neurotoxin A injection-therapy (BoNT-A), n=12 | VFSS  EGD | BoNT-A to the cricopharyngeus muscle led to an improvement of dysphagia in 100% of the patients (5 patients based on VFSS, 5 patients based on questionnaire, 2 patients based on interview) |
| (Shapiro et al. 1996) | case-series | isolated oropharyngeal IIM, n=3 | FEES  VFSS | of the three patients who underwent cricopharyngeal myotomy, one did not note any swallowing improvement, and a follow-up VFSS showed a persistent, albeit diminished, prominence of the cricopharyngeal muscle. The other two patients noted marked improvement in swallowing function with return to a full diet. In postoperative VFSS in one patient, cricopharyngeal dysfunction had resolved |
| (Sitzia et al. 2019) | case-report | PM, n=1 | FEES, EGD but not specific finding | IVIG therapy led to a mild improvement of symptoms |
| (St Guily et al. 1994) | retrospective observational study | PM, n=3 | clinical symptoms  FEES  partly manometry  partly VFSS | symptoms of dysphagia improved in two patients after steroid therapy (follow-up 18 and 8 months) |
| (Takamiya et al. 2019) | case-report | anti-NT5C1A-positive IBM, n=1 | VFSS | long-term improvement (> 1 year) after IVIG therapy |
| (Tang et al. 2018) | case-report | IIM, n=1 | barium radiography, HRM, EGD | POEM surgery  led to remission of symptoms (>23 months) |
| (Tateyama et al. 2003) | case-series | familial IBM, n=2 | not further stated | improvement of symptoms in one patient after intravenous methylprednisolone pulse therapy followed by oral administration of prednisolone and azathioprine. |
| (Thomas et al. 1972) | case-report | PM, n=1 | VFSS | improvement of symptoms and VFSS finding after oral prednisolone |
| (Tierney and Jirjis 1997) | case-report | DM, n=1 | VFSS | improvement of symptoms after oral prednisone |
| (Vencovsky et al. 1988) | case-report | PM, n=1 | VFSS  esophago-scopy | improvement of symptoms after myotomy |
| (Venhuizen et al. 2006) | case-report | DM associated with ovarian cancer, n=1 | tube-dependent | dysphagia resolved after ovarectomy and chemotherapy with paclitaxel/ carboplatin (and after initial intravenous prednisolone) |
| (Verma et al. 1991) | case-report | IBM, n=1 | upper gastroin-testinal radiographic study  esophago-scopy  barium-swallow-video | improvement of symptoms after myotomy (assessed after 8 months) |
| (Wanamaker et al. 1992) | case-report | PM, n=1 | not further stated | improvement of symptoms and swelling of the sternocleidomastoid muscle after steroid therapy |
| (Wang et al. 2011) | case-report | PM, n=1 | not further stated | dysphagia resolved after oral treatment with prednisolone |
| (Watanabe et al. 2016) | case-report | DM with subcutaneous edema, n=1 | not further stated | improvement of symptoms after tacrolimus and oral prednisolone |
| (Wenzel et al. 2001) | case-report | IBM associated with subacute cutaneous lupus erythematosus, n=1 | scintigraphy | improvement of symptoms after therapy with prednisolone and methotrexate |
| (Williams et al. 2003) | retrospective observational study | IIM with oropharyngeal dysphagia, n=13 | one or more of the following symptoms: bolus holdup; multiple swallows required to clear the pharynx; deglutitive coughing and/or choking; or postnasal regurgitation  Partly VFSS and manometry | the overall short- and long-term response rates to dilatation (n=8) were 50% and 25%, respectively.  two long-term responders, a patient that had an unsustained response and a patient who did not respond to dilatation, subsequently underwent cricopharyngeal myotomy, with further subjective and objective responses in the two that had previously responded to dilatation.  overall, an objective long-term response to cricopharyngeal disruption was 38%. |
| (Wintzen et al. 1988) | case-series | IBM, n=6 | VFSS | improvement of symptoms and disappearance of posterior indentation by the cricopharyngeus muscle in VFSS in 3 out of 4 patients after myotomy |
| (Yoshie et al. 2016) | case-report | DM associated with seminoma, n=1 | not further stated | chemotherapy of seminoma led to remission of dysphagia |
| (Zedan et al. 2008) | case-report | JDM with Anasarca, n=1 | tube dependent | improvement after oral prednisolone and azathioprine |
| (Zuber et al. 2013) | case-report | DM, n=1 | PEG-tube dependent | dysphagia resolved after IVIG, methotrexate and prednisolone. |

Publication bibliography

Al-Mayouf, S.; Al-Mazyed, A.; Bahabri, S. (2000): Efficacy of early treatment of severe juvenile dermatomyositis with intravenous methylprednisolone and methotrexate. In *Clinical rheumatology* 19 (2), pp. 138–141. DOI: 10.1007/s100670050032.

Almodovar, Raquel; Lindo, Daniel Paul; Martin, Helena; Mazzuchelli, Ramon; Pardo, Javier; Quiros, Francisco Javier; Zarco, Pedro (2012): Dermatomyositis and meningioma in the same patient. In *Reumatologia clinica* 8 (2), pp. 87–89. DOI: 10.1016/j.reuma.2011.06.009.

Alqatari, Safi; Riddell, Peter; Harney, Sinead; Henry, Michael; Murphy, Grainne (2018): MDA-5 associated rapidly progressive interstitial lung disease with recurrent Pneumothoraces: a case report. In *BMC pulmonary medicine* 18 (1), p. 59. DOI: 10.1186/s12890-018-0622-8.

Andonopoulos, A. P.; Gogos, C. A.; Tzanakakis, G. (1993): Subcutaneous edema: an "unrecognized" feature of acute polymyositis. In *Rheumatology international* 13 (4), pp. 159–161. DOI: 10.1007/bf00301264.

Bachmann, G.; Streppel, M.; Krug, B.; Neuen-Jacob, E. (2001): Cricopharyngeal muscle hypertrophy associated with florid myositis. In *Dysphagia* 16 (4), pp. 244–248. DOI: 10.1007/s00455-001-0082-8.

Barrera, P.; den Broeder, A. A.; van den Hoogen, F H; van Engelen, B. G.; van de Putte, L B (1998): Postural changes, dysphagia, and systemic sclerosis. In *Annals of the rheumatic diseases* 57 (6), pp. 331–338. DOI: 10.1136/ard.57.6.331.

Basnayake, Sajini K.; Blumbergs, Peter; Tan, Ju Ann; Roberts-Thompson, Peter J.; Limaye, Vidya (2015): Inflammatory myopathy with anti-SRP antibodies: case series of a South Australian cohort. In *Clinical rheumatology* 34 (3), pp. 603–608. DOI: 10.1007/s10067-014-2512-7.

Black, Michael; Marshman, Gillian (2011): Dermatomyositis and pemphigus vulgaris: association or coincidence? In *The Australasian journal of dermatology* 52 (2), e11-4. DOI: 10.1111/j.1440-0960.2010.00646.x.

Bonin, C. C.; da Silva, B Santos Pires; Mota, L. M.; Carvalho, J. F. de (2010): Severe and refractory myositis in mixed connective tissue disease: a description of a rare case. In *Lupus* 19 (14), pp. 1659–1661. DOI: 10.1177/0961203310376637.

Butt, Zeeshan; Patel, Leeza; Das, Manash K.; Mecoli, Christopher A.; Ramji, Alim (2017): NXP-2 Positive Dermatomyositis: A Unique Clinical Presentation. In *Case reports in rheumatology* 2017, p. 4817275. DOI: 10.1155/2017/4817275.

Camargo, Leonardo Valente de; Carvalho, Mary Souza de; Shinjo, Samuel Katsuyuki; de Oliveira, Acary Souza Bulle; Zanoteli, Edmar (2018): Clinical, Histological, and Immunohistochemical Findings in Inclusion Body Myositis. In *BioMed research international* 2018, p. 5069042. DOI: 10.1155/2018/5069042.

Caramaschi, P.; Blasi, D.; Carletto, A.; Randon, M.; Bambara, L. M. (1997): Megaoesophagus in a patient affected by dermatomyositis. In *Clinical rheumatology* 16 (1), pp. 106–107. DOI: 10.1007/bf02238774.

Chen, Ting; Pu, Chuanqiang; Shi, Qiang; Wang, Qian; Cong, Lu; Liu, Jiexiao et al. (2014): Chronic progressive external ophthalmoplegia with inflammatory myopathy. In *International journal of clinical and experimental pathology* 7 (12), pp. 8887–8892.

Cherin, P.; Pelletier, S.; Teixeira, A.; Laforet, P.; Simon, A.; Herson, S.; Eymard, B. (2002): Intravenous immunoglobulin for dysphagia of inclusion body myositis. In *Neurology* 58 (2), p. 326.

Cherin, Patrick; Delain, Jean-Christophe; Jaeger, Christophe de; Crave, Jean-Charles (2015): Subcutaneous Immunoglobulin Use in Inclusion Body Myositis: A Review of 6 Cases. In *Case reports in neurology* 7 (3), pp. 227–232. DOI: 10.1159/000441490.

Chinniah, Keith J.; Mody, Girish M. (2017): Recovery from severe dysphagia in systemic sclerosis - myositis overlap: a case report. In *African health sciences* 17 (2), pp. 593–596. DOI: 10.4314/ahs.v17i2.38.

Cnop, Katia; Martinez, Boris; Austad, Kirsten E. (2019): Resistant dermatomyositis in a rural indigenous Maya woman. In *BMJ case reports* 12 (2). DOI: 10.1136/bcr-2017-223886.

Cochicho, Joana; Madaleno, Joao; Louro, Emilia; Simao, Adelia; Carvalho, Armando (2016): Polymyositis and the Spectrum of Scleroderma Disorders. In *European journal of case reports in internal medicine* 3 (1), p. 346. DOI: 10.12890/2015_000346.

Dagan, Amir; Markovits, Doron; Braun-Moscovici, Yolanda; Rozin, Alexander; Toledano, Kohava; Balbir-Gurman, Alexandra (2013): Life-threatening oropharyngeal aphagia as the major manifestation of dermatomyositis. In *The Israel Medical Association journal : IMAJ* 15 (8), pp. 453–455.

Dalakas, M. C.; Sonies, B.; Dambrosia, J.; Sekul, E.; Cupler, E.; Sivakumar, K. (1997): Treatment of inclusion-body myositis with IVIg: a double-blind, placebo-controlled study. In *Neurology* 48 (3), pp. 712–716. DOI: 10.1212/wnl.48.3.712.

Danon, M. J.; Friedman, M. (1989): Inclusion body myositis associated with progressive dysphagia: treatment with cricopharyngeal myotomy. In *The Canadian journal of neurological sciences. Le journal canadien des sciences neurologiques* 16 (4), pp. 436–438. DOI: 10.1017/s031716710002953x.

Darrow, D. H.; Hoffman, H. T.; Barnes, G. J.; Wiley, C. A. (1992): Management of dysphagia in inclusion body myositis. In *Archives of otolaryngology--head & neck surgery* 118 (3), pp. 313–317. DOI: 10.1001/archotol.1992.01880030103021.

Di Pede, Chiara; Masiero, Stefano; Bonsangue, Valentina; Ragona, Rosario Marchese; Del Felice, Alessandra (2016): Botulinum toxin and rehabilitation treatment in inclusion body myositis for severe oropharyngeal dysphagia. In *Neurological sciences : official journal of the Italian Neurological Society and of the Italian Society of Clinical Neurophysiology* 37 (10), pp. 1743–1745. DOI: 10.1007/s10072-016-2586-x.

Dias, Leonardo Pires Novais; Faria, Ana Luiza Antunes; Scandiuzzi, Maissa Marcola; Inhaia, Claudia Luci dos Santos; Shida, Jorge Yoshinori; Gebrim, Luiz Henrique (2015): A rare case of severe myositis as paraneoplastic syndrome on breast cancer. In *World journal of surgical oncology* 13, p. 134. DOI: 10.1186/s12957-015-0534-5.

Dietz, F.; Logeman, J. A.; Sahgal, V.; Schmid, F. R. (1980): Cricopharyngeal muscle dysfunction in the differential diagnosis of dysphagia in polymyositis. In *Arthritis and rheumatism* 23 (4), pp. 491–495. DOI: 10.1002/art.1780230412.

Dobloug, Cecilie; Walle-Hansen, Ragnhild; Gran, Jan Tore; Molberg, Oyvind (2012): Long-term follow-up of sporadic inclusion body myositis treated with intravenous immunoglobulin: a retrospective study of 16 patients. In *Clinical and experimental rheumatology* 30 (6), pp. 838–842.

Ertekin, C.; Aydogdu, I.; Yuceyar, N. (1996): Piecemeal deglutition and dysphagia limit in normal subjects and in patients with swallowing disorders. In *Journal of neurology, neurosurgery, and psychiatry* 61 (5), pp. 491–496. DOI: 10.1136/jnnp.61.5.491.

Eura, Nobuyuki; Sugie, Kazuma; Kiriyama, Takao; Ueno, Satoshi (2015): Characteristic dysphagia as a manifestation of dermatomyositis on oropharyngeal muscle imaging. In *Journal of clinical rheumatology : practical reports on rheumatic & musculoskeletal diseases* 21 (2), pp. 105–106. DOI: 10.1097/RHU.0000000000000225.

Gameiro, Rita de Sousa; Reis, Ana Isabel Alves; Grilo, Ana Cristina; Noronha, Carla (2018): Following leads: connecting dysphagia to mixed connective tissue disease. In *BMJ case reports* 2018. DOI: 10.1136/bcr-2017-223699.

Giannini, M.; Macchia, L.; Amati, A.; Lia, A.; Girolamo, F.; D'Abbicco, D. et al. (2018): A rare association of anti-alanine-transfer RNA synthetase (anti-PL12) syndrome and sporadic inclusion body myositis. In *Scandinavian journal of rheumatology* 47 (4), pp. 336–337. DOI: 10.1080/03009742.2017.1350747.

Gibson, J.; Lamey, P. J.; Zoma, A.; Ballantyne, J. (1991): Tongue atrophy in mixed connective tissue disease. In *Oral surgery, oral medicine, and oral pathology* 71 (3), pp. 294–296. DOI: 10.1016/0030-4220(91)90302-s.

Gyorffy, Janelle B.; Marowske, Johanna; Gancayco, John (2018): A Rare Cause of Dysphagia and Weight Loss. In *Case reports in gastroenterology* 12 (3), pp. 640–645. DOI: 10.1159/000493919.

Hafejee, A.; Coulson, I. H. (2005): Dysphagia in dermatomyositis secondary to bladder cancer: rapid response to combined immunoglobulin and methylprednisolone. In *Clinical and experimental dermatology* 30 (1), pp. 93–94. DOI: 10.1111/j.1365-2230.2004.01671.x.

Hirano, F.; Tanaka, H.; Nomura, Y.; Matsui, T.; Makino, Y.; Fukawa, E. et al. (1993): Successful treatment of refractory polymyositis with pulse intravenous cyclophosphamide and low-dose weekly oral methotrexate therapy. In *Internal medicine (Tokyo, Japan)* 32 (9), pp. 749–752. DOI: 10.2169/internalmedicine.32.749.

Houser, S. M.; Calabrese, L. H.; Strome, M. (1998): Dysphagia in patients with inclusion body myositis. In *The Laryngoscope* 108 (7), pp. 1001–1005. DOI: 10.1097/00005537-199807000-00009.

Iannone, Florenzo; Giannini, Margherita; Lapadula, Giovanni (2015): Recovery of barium swallow radiographic abnormalities in a patient with dermatomyositis and severe dysphagia after high-dose intravenous immunoglobulins. In *Journal of clinical rheumatology : practical reports on rheumatic & musculoskeletal diseases* 21 (4), p. 227. DOI: 10.1097/RHU.0000000000000252.

Joshi, Deepak; Mahmood, Rizwan; Williams, Peter; Kitchen, Paul (2008): Dysphagia secondary to dermatomyositis treated successfully with intravenous immunoglobulin: a case report. In *International archives of medicine* 1 (1), p. 12. DOI: 10.1186/1755-7682-1-12.

Kagen, L. J.; Hochman, R. B.; Strong, E. W. (1985): Cricopharyngeal obstruction in inflammatory myopathy (polymyositis/dermatomyositis). Report of three cases and review of the literature. In *Arthritis and rheumatism* 28 (6), pp. 630–636. DOI: 10.1002/art.1780280606.

Kwon, Kyoung Min; Lee, Jung Soo; Kim, Yeo Hyung (2018): A case report of life-threatening acute dysphagia in dermatomyositis: Challenges in diagnosis and treatment. In *Medicine* 97 (17), e0508. DOI: 10.1097/MD.0000000000010508.

Kwon, Patrick M.; Zhou, Lan; Motiwala, Rajeev; Kerr, Leslie D.; Shin, Susan C. (2017): Immune Myopathy With Perimysial Pathology Associated With Interstitial Lung Disease and Anti-EJ Antibodies. In *Journal of clinical neuromuscular disease* 18 (4), pp. 223–227. DOI: 10.1097/CND.0000000000000148.

Labeit, Bendix; Muhle, Paul; Suntrup-Krueger, Sonja; Ahring, Sigrid; Ruck, Tobias; Dziewas, Rainer; Warnecke, Tobias (2019): Dysphagia as Isolated Manifestation of Jo-1 Associated Myositis? In *Frontiers in neurology* 10, p. 739. DOI: 10.3389/fneur.2019.00739.

Laurikainen, E.; Aitasalo, K.; Halonen, P.; Falck, B.; Kalimo, H. (1992): Muscle pathology in idiopathic cricopharyngeal dysphagia. Enzyme histochemical and electron microscopic findings. In *European archives of oto-rhino-laryngology : official journal of the European Federation of Oto-Rhino-Laryngological Societies (EUFOS) : affiliated with the German Society for Oto-Rhino-Laryngology - Head and Neck Surgery* 249 (4), pp. 216–223. DOI: 10.1007/bf00178473.

Lee, Ki-Hong; Lim, Sung-Ryoun; Kim, Yeon-Joo; Lee, Kyung-Ju; Myung, Dae-Seong; Jeong, Hae-Chang et al. (2008): Acute dermatomyositis associated with generalized subcutaneous edema. In *Rheumatology international* 28 (8), pp. 797–800. DOI: 10.1007/s00296-008-0520-0.

Liu, Louis W. C.; Tarnopolsky, Mark; Armstrong, David (2004): Injection of botulinum toxin A to the upper esophageal sphincter for oropharyngeal dysphagia in two patients with inclusion body myositis. In *Canadian journal of gastroenterology = Journal canadien de gastroenterologie* 18 (6), pp. 397–399. DOI: 10.1155/2004/360537.

MacFarlane, Lindsey; Osman, Nora; Ritter, Susan; Miller, Amy L.; Loscalzo, Joseph (2016): CLINICAL PROBLEM-SOLVING. Eye of the Beholder. In *The New England journal of medicine* 374 (18), pp. 1774–1779. DOI: 10.1056/NEJMcps1509470.

Malandraki, Georgia A.; Kaufman, Andrew; Hind, Jacqueline; Ennis, Stephanie; Gangnon, Ronald; Waclawik, Andrew; Robbins, Joanne (2012): The effects of lingual intervention in a patient with inclusion body myositis and Sjogren's syndrome: a longitudinal case study. In *Archives of physical medicine and rehabilitation* 93 (8), pp. 1469–1475. DOI: 10.1016/j.apmr.2012.02.010.

Marie, I.; Hachulla, E.; Levesque, H.; Reumont, G.; Ducrotte, P.; Cailleux, N. et al. (1999): Intravenous immunoglobulins as treatment of life threatening esophageal involvement in polymyositis and dermatomyositis. In *The Journal of rheumatology* 26 (12), pp. 2706–2709.

Marie, I.; Menard, J-F; Hatron, P. Y.; Hachulla, E.; Mouthon, L.; Tiev, K. et al. (2010): Intravenous immunoglobulins for steroid-refractory esophageal involvement related to polymyositis and dermatomyositis: a series of 73 patients. In *Arthritis care & research* 62 (12), pp. 1748–1755. DOI: 10.1002/acr.20325.

Matsuoka, Naoki; Asano, Tomoyuki; Sato, Shuzo; Sasajima, Tomomi; Fujita, Yuya; Temmoku, Jumpei et al. (2019): A case of dermatomyositis complicated with pleural effusion and massive ascites. In *Fukushima journal of medical science*. DOI: 10.5387/fms.2019-09.

Metheny, J. A. (1978): Dermatomyositis: a vocal and swallowing disease entity. In *The Laryngoscope* 88 (1 Pt 1), pp. 147–161. DOI: 10.1002/lary.1978.88.1.147.

Mii, Sumiyuki; Niiyama, Shiro; Kusunoki, Mai; Arai, Satoru; Katsuoka, Kensei (2006): Cyclosporine A as treatment of esophageal involvement in dermatomyositis. In *Rheumatology international* 27 (2), pp. 183–185. DOI: 10.1007/s00296-006-0168-6.

Milisenda, Jose C.; Doti, Pamela I.; Prieto-Gonzalez, Sergio; Grau, Josep M. (2014): Dermatomyositis presenting with severe subcutaneous edema: five additional cases and review of the literature. In *Seminars in arthritis and rheumatism* 44 (2), pp. 228–233. DOI: 10.1016/j.semarthrit.2014.04.004.

Mugii, Naoki; Hasegawa, Minoru; Matsushita, Takashi; Hamaguchi, Yasuhito; Oohata, Sacihe; Okita, Hirokazu et al. (2016): Oropharyngeal Dysphagia in Dermatomyositis: Associations with Clinical and Laboratory Features Including Autoantibodies. In *PloS one* 11 (5), e0154746. DOI: 10.1371/journal.pone.0154746.

Murata, Ken-Ya; Kouda, Ken; Tajima, Fumihiro; Kondo, Tomoyoshi (2013): Balloon dilation in sporadic inclusion body myositis patients with Dysphagia. In *Clinical medicine insights. Case reports* 6, pp. 1–7. DOI: 10.4137/CCRep.S10200.

Nagano, Hiromi; Yoshifuku, Kousuke; Kurono, Yuichi (2009): Polymyositis with dysphagia treated with endoscopic balloon dilatation. In *Auris, nasus, larynx* 36 (6), pp. 705–708. DOI: 10.1016/j.anl.2009.04.007.

Nagano, Yuka; Inoue, Yasuhiro; Shimura, Tadanobu; Fujikawa, Hiroyuki; Okugawa, Yoshinaga; Hiro, Junichiro et al. (2015): Exacerbation of Dermatomyositis with Recurrence of Rectal Cancer: A Case Report. In *Case reports in oncology* 8 (3), pp. 482–486. DOI: 10.1159/000439519.

Noda, S.; Umezaki, H.; Itoh, H.; Hiromatsu, K.; Yamamoto, T. (1988): Chronic focal polymyositis. In *Journal of neurology, neurosurgery, and psychiatry* 51 (5), p. 728. DOI: 10.1136/jnnp.51.5.728.

Ocampo, Cassandra; Segwagwe, Molebedi L.; Deonarain, Julian; Cainelli, Francesca; Vento, Sandro (2014): A case of severe dermatomyositis in an African woman. In *The Israel Medical Association journal : IMAJ* 16 (5), pp. 329–330.

Ogawa-Momohara, Mariko; Muro, Yoshinao; Kono, Michihiro; Akiyama, Masashi (2019): Prognosis of dysphagia in dermatomyositis. In *Clinical and experimental rheumatology* 37 (1), p. 165.

Oh, Terry H.; Brumfield, Kathlyn A.; Hoskin, Tanya L.; Kasperbauer, Jan L.; Basford, Jeffrey R. (2008): Dysphagia in inclusion body myositis: clinical features, management, and clinical outcome. In *American journal of physical medicine & rehabilitation* 87 (11), pp. 883–889. DOI: 10.1097/PHM.0b013e31818a50e2.

Oh, Terry H.; Brumfield, Kathlyn A.; Hoskin, Tanya L.; Stolp, Kathryn A.; Murray, Joseph A.; Bassford, Jeffrey R. (2007): Dysphagia in inflammatory myopathy. Clinical characteristics, treatment strategies, and outcome in 62 patients. In *Mayo Clinic proceedings* 82 (4), pp. 441–447.

Otao, Goh; Yamashita, Shu-ichi; Kyoraku, Itaru; Shiomi, Kazutaka; Nakazato, Masamitsu (2007): Dysphagia due to inflammation of oral muscles as the first symptom of dermatomyositis. In *Internal medicine (Tokyo, Japan)* 46 (12), pp. 923–924. DOI: 10.2169/internalmedicine.46.0068.

Palace, J.; Losseff, N.; Clough, C. (1993): Isolated dysphagia due to polymyositis. In *Muscle & nerve* 16 (6), pp. 680–681.

Papakonstantinou, Eleni; Kapp, Alexander; Raap, Ulrike (2016): A mild form of dermatomyositis as a prodromal sign of lung adenocarcinoma: a case report. In *Journal of medical case reports* 10, p. 34. DOI: 10.1186/s13256-016-0816-8.

Pars, Kaweh; Garde, Niklas; Skripuletz, Thomas; Pul, Refik; Dengler, Reinhard; Stangel, Martin (2013): Subcutaneous immunoglobulin treatment of inclusion-body myositis stabilizes dysphagia. In *Muscle & nerve* 48 (5), pp. 838–839. DOI: 10.1002/mus.23895.

Patrick, Cherin; Jean-Christophe, Delain; Jean-Charles, Crave; Odile, Cartry (2014): High-dose subcutaneous immunoglobulins for the treatment of severe treatment-resistant polymyositis. In *Case reports in rheumatology* 2014, p. 458231. DOI: 10.1155/2014/458231.

Porubsky, E. S.; Murray, J. P.; Pratt, L. L. (1973): Cricopharyngeal achalasia in dermatomyositis. In *Archives of otolaryngology (Chicago, Ill. : 1960)* 98 (6), pp. 428–429. DOI: 10.1001/archotol.1973.00780020442018.

Prossliner, Veronika; Philipp, Magdalena; Moosbrugger, Verena; Poewe, Werner; Zangerle, Robert; Wanschitz, Julia; Schmuth, Matthias (2016): Severe dysphagia, myalgia, and rash in a 55-year-old man. In *Journal der Deutschen Dermatologischen Gesellschaft = Journal of the German Society of Dermatology : JDDG* 14 (4), pp. 438–441. DOI: 10.1111/ddg.12863.

Raghu, Padmanabhan; Manadan, Augustine M.; Schmukler, Juan; Mathur, Tanisha; Block, Joel A. (2015): Pulse Dose Methylprednisolone Therapy for Adult Idiopathic Inflammatory Myopathy. In *American journal of therapeutics* 22 (4), pp. 244–247. DOI: 10.1097/MJT.0000000000000175.

Ramachandran, Ramesh B.; Swash, Michael (2004): Pharyngeal Dysphagia in dermatomyositis: responsive to cyclophosphamide. In *Journal of clinical neuromuscular disease* 5 (3), pp. 166–167. DOI: 10.1097/00131402-200403000-00008.

Riminton, D. S.; Chambers, S. T.; Parkin, P. J.; Pollock, M.; Donaldson, I. M. (1993): Inclusion body myositis presenting solely as dysphagia. In *Neurology* 43 (6), pp. 1241–1243.

Ryan, Aisling; Nor, Azli M.; Costigan, Donal; Foley-Nolan, Daragh; El-Rafie, Ahmed; Farrell, Michael A.; Hardiman, Orla (2003): Polymyositis masquerading as motor neuron disease. In *Archives of neurology* 60 (7), pp. 1001–1003. DOI: 10.1001/archneur.60.7.1001.

Schrey, Aleksi; Airas, Laura; Jokela, Manu; Pulkkinen, Jaakko (2017): Botulinum toxin alleviates dysphagia of patients with inclusion body myositis. In *Journal of the neurological sciences* 380, pp. 142–147. DOI: 10.1016/j.jns.2017.07.031.

Shapiro, J.; Martin, S.; DeGirolami, U.; Goyal, R. (1996): Inflammatory myopathy causing pharyngeal dysphagia. A new entity. In *The Annals of otology, rhinology, and laryngology* 105 (5), pp. 331–335. DOI: 10.1177/000348949610500501.

Sitzia, Clementina; Sansone, Valeria Ada; Corsi Romanelli, Massimiliano Marco (2019): Creatine kinase elevation: a neglected clue to the diagnosis of polymyositis. A case report. In *Clinical chemistry and laboratory medicine* 57 (7), e149-e151. DOI: 10.1515/cclm-2018-0928.

St Guily, J. L.; Perie, S.; Willig, T. N.; Chaussade, S.; Eymard, B.; Angelard, B. (1994): Swallowing disorders in muscular diseases: functional assessment and indications of cricopharyngeal myotomy. In *Ear, nose, & throat journal* 73 (1), pp. 34–40.

Takamiya, Motonori; Takahashi, Yoshiaki; Morimoto, Mizuki; Morimoto, Nobutoshi; Yamashita, Satoshi; Abe, Koji (2019): Effect of intravenous immunoglobulin therapy on anti-NT5C1A antibody-positive inclusion body myositis after successful treatment of hepatitis C: A case report. In *eNeurologicalSci* 16, p. 100204. DOI: 10.1016/j.ensci.2019.100204.

Tang, Yurong; Xiong, Wenjie; Yu, Ting; Wang, Meifeng; Zhang, Guoxin; Lin, Lin (2018): Eosinophilic Esophageal Myositis a Plausible Cause of Histological Changes of Primary Jackhammer Esophagus: A Case Report. In *The American journal of gastroenterology* 113 (1), pp. 150–152. DOI: 10.1038/ajg.2017.433.

Tateyama, Maki; Saito, Naohiro; Fujihara, Kazuo; Shiga, Yusei; Takeda, Atsushi; Narikawa, Koichi et al. (2003): Familial inclusion body myositis: a report on two Japanese sisters. In *Internal medicine (Tokyo, Japan)* 42 (10), pp. 1035–1038. DOI: 10.2169/internalmedicine.42.1035.

Thomas, F. B.; LeBauer, S.; Greenberger, N. J. (1972): Polymyositis masquerading as carcinoma of the cervical esophagus. In *Archives of internal medicine* 129 (6), pp. 984–986.

Tierney, D.; Jirjis, J. N. (1997): A woman with difficulty swallowing. In *Tennessee medicine : journal of the Tennessee Medical Association* 90 (11), pp. 462–463.

Vencovsky, J.; Rehak, F.; Pafko, P.; Jirasek, A.; Valesova, M.; Alusik, S.; Trnavsky, K. (1988): Acute cricopharyngeal obstruction in dermatomyositis. In *The Journal of rheumatology* 15 (6), pp. 1016–1018.

Venhuizen, Annemarie C.; Martens, Jolise E.; van der Linden, Paul J Q (2006): Dermatomyositis as first presentation of ovarian cancer. In *Acta obstetricia et gynecologica Scandinavica* 85 (10), pp. 1271–1272. DOI: 10.1080/00016340600935235.

Verma, A.; Bradley, W. G.; Adesina, A. M.; Sofferman, R.; Pendlebury, W. W. (1991): Inclusion body myositis with cricopharyngeus muscle involvement and severe dysphagia. In *Muscle & nerve* 14 (5), pp. 470–473. DOI: 10.1002/mus.880140514.

Wanamaker, J. R.; Wanamaker, H. H.; Lavertu, P. (1992): Polymyositis presenting as a neck mass. In *Archives of otolaryngology--head & neck surgery* 118 (3), pp. 318–320. DOI: 10.1001/archotol.1992.01880030108022.

Wang, Han; Li, Hong; Kai, Cui; Deng, Juelin (2011): Polymyositis associated with hypothyroidism or hyperthyroidism: two cases and review of the literature. In *Clinical rheumatology* 30 (4), pp. 449–458. DOI: 10.1007/s10067-010-1570-8.

Watanabe, M.; Natsuga, K.; Arita, K.; Abe, R.; Shimizu, H. (2016): Generalized acute subcutaneous edema as a rare cutaneous manifestation of severe dermatomyositis. In *Journal of the European Academy of Dermatology and Venereology : JEADV* 30 (11), e151-e152. DOI: 10.1111/jdv.13460.

Wenzel, J.; Uerlich, M.; Gerdsen, R.; Bieber, T.; Boehm, I. (2001): Association of inclusion body myositis with subacute cutaneous lupus erythematosus. In *Rheumatology international* 21 (2), pp. 75–77. DOI: 10.1007/s002960100137.

Williams, R. B.; Grehan, M. J.; Hersch, M.; Andre, J.; Cook, I. J. (2003): Biomechanics, diagnosis, and treatment outcome in inflammatory myopathy presenting as oropharyngeal dysphagia. In *Gut* 52 (4), pp. 471–478.

Wintzen, A. R.; Bots, G. T.; Bakker, H. M. de; Hulshof, J. H.; Padberg, G. W. (1988): Dysphagia in inclusion body myositis. In *Journal of neurology, neurosurgery, and psychiatry* 51 (12), pp. 1542–1545.

Yoshie, Hidekazu; Nakazawa, Ryuto; Usuba, Wataru; Kudo, Hiroya; Sato, Yuichi; Sasaki, Hideo; Chikaraishi, Tatsuya (2016): Paraneoplastic Dermatomyositis Associated with Metastatic Seminoma. In *Case reports in urology* 2016, p. 7050981. DOI: 10.1155/2016/7050981.

Zedan, Magdy; El-Ayouty, Moustafa; Abdel-Hady, Hesham; Shouman, Basma; El-Assmy, Mohamed; Fouda, Ashraf (2008): Anasarca: not a nephrotic syndrome but dermatomyositis. In *European journal of pediatrics* 167 (7), pp. 831–834. DOI: 10.1007/s00431-008-0716-z.

Zuber, M. A.; Kouba, M.; Rudolph, S. E.; Weller, M.; Hrdlicka, P. (2013): Severe dysphagia and erythrodermia in a 59-year-old man. In *Der Internist* 54 (3), pp. 359–365. DOI: 10.1007/s00108-012-3225-0.
